# Economic Instruments for Population Diet and Physical Activity Behaviour Change: A Systematic Scoping Review

**DOI:** 10.1371/journal.pone.0075070

**Published:** 2013-09-24

**Authors:** Ian Shemilt, Gareth J. Hollands, Theresa M. Marteau, Ryota Nakamura, Susan A. Jebb, Michael P. Kelly, Marc Suhrcke, David Ogilvie

**Affiliations:** Behaviour and Health Research Unit, University of Cambridge, Cambridge, United Kingdom; Paris Institute of Technology for Life, Food and Environmental Sciences, France

## Abstract

**Background:**

Unhealthy diet and low levels of physical activity are common behavioural factors in the aetiology of many non-communicable diseases. Recent years have witnessed an upsurge of policy and research interest in the use of taxes and other economic instruments to improve population health.

**Objective:**

To assemble, configure and analyse empirical research studies available to inform the public health case for using economic instruments to promote dietary and physical activity behaviour change.

**Methods:**

We conducted a systematic scoping review of evidence for the effects of specific interventions to change, or general exposure to variations in, prices or income on dietary and physical activity behaviours and corollary outcomes. Systematic electronic searches and parallel snowball searches retrieved >1 million study records. Text mining technologies were used to prioritise title-abstract records for screening. Eligible studies were selected, classified and analysed in terms of key characteristics and principal findings, using a narrative, configuring synthesis focused on implications for policy and further research.

**Results:**

We identified 880 eligible studies, including 192 intervention studies and 768 studies that incorporated evidence for prices or income as correlates or determinants of target outcomes. Current evidence for the effects of economic instruments and exposures on diet and physical activity is limited in quality and equivocal in terms of its policy implications. Direct evidence for the effects of economic instruments is heavily skewed towards impacts on diet, with a relative lack of evidence for impacts on physical activity.

**Conclusions:**

The evidence-based case for using economic instruments to promote dietary and physical activity behaviour change may be less compelling than some proponents have claimed. Future research should include measurement of people’s actual behavioural responses using study designs capable of generating reliable causal inferences regarding intervention effects. Policy implementation needs to be carefully aligned with evaluation planning and design.

## Introduction

In 2008, non-communicable diseases (NCDs) caused 36 million (63%) of global deaths and 2.8 million people died as a consequence of being overweight or obese [1]. A large proportion of NCD deaths occur prematurely [1], imposing large and avoidable costs in human, social and economic terms [2,3]. Alongside smoking and harmful use of alcohol, unhealthy diets and low levels of physical activity are common behavioural factors in the aetiology of the most prevalent and preventable NCDs [4].

The term ‘economic instruments’ encompasses fiscal or legislative government policies designed to change the relative prices of goods or services or people’s disposable income, and promotional practices used by retailers to change the relative prices of goods and services. Recent years have witnessed an upsurge of policy and research interest in the potential use of economic instruments to shape markets for specific goods and services in ways that incentivise healthier behaviours [5-8]. This can be seen as one dimension of a broader policy context in which national and local governments aspire to influence population health behaviours by altering the environments within which people make choices [9-11].

Extant research evidence largely corroborates the public health case for using economic instruments to discourage purchasing and use of tobacco and alcohol products [12-16]. However, the corresponding case for the use of these instruments to encourage healthier eating and physical activity remains controversial from both an evidence and a policy perspective. We therefore conducted a systematic scoping review to configure emprical evidence that is currently available to inform the case for or against the use of economic instruments to promote health-enhancing dietary and physical activity behaviour change.

### Objectives

The specific objectives of this scoping review were to assemble and conduct a configuring synthesis of empirical research evidence for the effects of economic instruments to change, or general exposure to changes in, prices or income on diet- and physical activity-related behaviours and corollary health-related outcomes. Evidence from intervention studies of the effects of economic instruments can be regarded as providing direct evidence, whilst evidence from studies that include assessment of the effects of general exposure to changes in prices or income can be regarded as providing supplementary, indirect evidence for the potential effects of economic instruments that are intended to operate via such price or income mechanisms.

## Methods

### Nature of the review

The methods applied in this systematic scoping review, including provisional study eligibility criteria, were pre-specified and documented in a protocol developed by all members of the review team (IS, GJH, TMM, RN, SAJ, MPK, MS and DO) (Protocol S1). Whilst scoping reviews follow a similar research process to systematic reviews [17] they differ from systematic reviews in terms of their objectives and key characteristics [18-21]. Scoping reviews explore, delimit and describe a broad evidence base whose boundaries are unclear at the outset, often as a preliminary stage to systematic reviews. Pre-specified eligibility criteria are therefore provisional and it is accepted that these may be refined and re-applied iteratively during the review, based on emergent knowledge of the studies and evidence encountered.

In parallel, scoping reviews are characterised by evidence synthesis strategies that focus on configuring or mapping evidence for effects [22,23], rather than on aggregating such evidence as exemplified by the use of meta-analysis to estimate pooled effect-sizes [24]. In these circumstances, refined conceptual understanding of interventions and their proposed mechanisms of action becomes an intended output of the scoping process rather than its starting point. However, in conjunction with this more iterative, formative approach, scoping reviews can still be conducted using systematic, explicit methods. They can therefore be reported in compliance with established reporting guidelines for conventional systematic reviews of interventions [25,26] (Checklist S1).

### Criteria for considering studies for this review

Final eligibility criteria for selecting studies into this scoping review were

#### Types of studies

Empirical primary studies and reviews of any design (encompassing experimental, quasi-experimental, non-experimental and modelling studies; systematic and non-systematic reviews). Reviews were included if at least one of their included primary studies met other eligibility criteria. No restrictions for publication status, year or language were applied in searches.

#### Types of participants

Human participants of any age, with no restrictions for demographic, socio-economic, or clinical characteristics. Animal studies were excluded.

#### Types of intervention or exposure

Economic instruments (direct evidence) – Price promotions, taxes (including tax exemptions), supply-side subsidies (including subsidy removal), direct unit pricing legislation, or transfer payments (see Interventions S1 for a definition of each instrument). These interventions alter remunerative incentives by changing prices, except for transfer payments, which alter people’s budget constraints by changing their income. No restrictions were applied for type of comparator. Studies of personal financial incentives – defined as rewards or penalties with a monetary value, provided directly to individuals contingent on performance of specific behaviours or achievement of specific outcomes – were excluded due to our focus on population- rather than individual-level interventions. Exposures (indirect evidence) – Exposure to variation in prices of final consumer products or services and/or individual or household income (i.e. studies of prices or income as correlates or determinants of target outcomes).

#### Types of outcome measures

i. Purchasing of food, non-alcoholic beverages or physical activity-related products or services; ii. other diet- or physical activity-related behaviours (e.g. food preparation, mealtime, snacking, physical activity or sedentary behaviours); iii. proximal consequences of such behaviours (i.e. those closer to the point of intervention or exposure in proposed causal pathways linking an intervention or exposure with final health outcomes), such as food, energy or nutrient intake, energy expenditure and physical fitness; and iv. distal consequences of such behaviours (i.e. those further from the point of intervention or exposure in proposed causal pathways linking an intervention or exposure with final health outcomes), namely modifiable physiological or metabolic risk factors for NCDs, such as body weight, blood cholesterol, blood glucose and blood pressure. Studies that did not report outcomes in at least one of these categories (i-iv) were excluded. For example, studies reporting measures of morbidity or mortality associated with NCDs (e.g. cardiovascular diseases, diabetes, cancers and chronic respiratory diseases) were excluded if they did not also report outcomes in at least one eligible category (i-iv). No restrictions were applied for type of measurement instrument or scale.

### Location and selection of studies

Methods for locating and selecting eligible studies are described in detail elsewhere [27,28] and summarised here. We pre-specified provisional eligibility criteria, developed a coding guide to inform assessments of eligibility and used this to document any revisions made to provisional eligibility criteria during the study selection stage of the review. In practice, no substantive revisions were made to eligibility criteria. We conducted snowball searches in parallel with systematic searches of electronic literature databases. Snowball searches (i.e. scanning reference lists and forward citation tracking) [29] were conducted by one reviewer (IS) using PubMed and Google Scholar.

We designed systematic search strategies for MEDLINE (Ovid SP), EMBASE (Ovid SP) and PsycINFO (Ovid SP). These search strategies were necessarily highly sensitive, due to the following factors: our intention to progressively refine pre-specified, provisional eligibility criteria; our decision to impose few eligibility restrictions for types of participants, comparators, study designs or publication characteristics; the fact that search terms based on target intervention concepts were unlikely to be specific to titles, abstracts or indexing of eligible study reports; target outcomes encompassed multiple sets of health behaviours, proximal consequences of such behaviours, and the full range of physiological and metabolic risk factors for NCDs. We tested draft search strategies for their sensitivity to retrieve a reference set of 45 records of potentially eligible study reports, assembled using non-systematic internet searches and contacts with topic experts in the author team. We refined search strategies until they retrieved 100% of the reference set records indexed in each database, with a concurrent aim to minimise search yields so far as possible. The process of testing and refining draft search strategies confirmed that we could not achieve greater specificity without sacrificing sensitivity to retrieve eligible reference set records. We then adapted final versions for eight other electronic literature databases, based on close inspection of thesauri, scope notes and search syntax for each database.

Final systematic search strategies were executed between 11 July and 11 August 2011 in 11 relevant electronic literature databases from inception to present: MEDLINE (Ovid SP), EMBASE (Ovid SP), PsycINFO (Ovid SP), EconLit (EBSCO), SPORTDiscus with Full Text (EBSCO), Applied Social Sciences Index and Abstracts (CSA Illumina), Cochrane Database of Systematic Reviews (Wiley Online Library), Database of Abstracts of Reviews of Effects (Wiley Online Library), Health Technology Assessment Database (Wiley Online Library), NHS Economic Evaluation Database (Wiley Online Library), and Database of Promoting Health Effectiveness Reviews (EPPI Centre). Full details of systematic search strategies, search dates and yields are provided as Supporting Information files (Search Strategies S1 and Table S1).

All retrieved study records (title-abstract records) were exported to Endnote X4 reference management software and imported into EPPI Reviewer 4 systematic review software [30]. We next ran EPPI Reviewer 4’s integrated automatic de-duplication software. We used text mining technologies to prioritise records for manual screening [31,32]. Text mining is “an automated process that can assist with the identification and structuring of patterns in the text of individual documents and across multiple documents” [33]. In this scoping review, text mining technologies were applied to automatically analyse text in the growing pool of screened title-abstract records to identify patterns of terms likely to distinguish eligible from ineligible records, search for those unscreened records most likely to be judged eligible and prioritise these to be manually screened next. Our use of text mining technologies to support title-abstract screening was justified by the extremely large number of records retrieved by systematic searches, which made application of conventional screening methods [31,34] impractical within available resources.

A preceding stage to application of text mining technologies involved manual screening of a random sample of title-abstract records to estimate a baseline inclusion rate (BIR). The BIR is an a priori estimate of the proportion of retrieved title-abstract records expected to be assessed as provisionally eligible. Our estimated BIR was 0.00348, indicating that ≈0.3%, or ≈3,669 of >1 million, retrieved records could be expected to be assessed as provisionally eligible. This estimate was used as a target to monitor the overall progress of title-abstract screening. Sequential sets of title-abstract records were then prioritised using text mining and assigned for manual screening. One reviewer (IS) undertook all screening assignments prioritised by text mining. Final eligibility criteria were confirmed at the end of the title-abstract screening stage, in consultation with the multi-disciplinary review team and with reference to a purposive sample of provisionally eligible, or borderline eligible, full-text study reports. Provisional eligibility decisions were reviewed against final eligibility criteria.

Corresponding full-text reports were sought for all title-abstract records assessed as provisionally eligible via electronic library resources of three universities (University of Cambridge, University of East Anglia and King’s College, London). Full-text screening and eligibility assessment was performed by one reviewer (IS). Full-text reports located using snowball searches were also assessed against final eligibility criteria by one reviewer (IS). Further duplicates were identified manually and removed at this stage. Multiple reports of the same study were linked into a single study.

### Data collection and analysis

All included studies were classified by behavioural domain (diet, physical activity, both), study type (intervention study, exposure study, both; primary study or review) and population (including a focus on high income country (HIC) [35] populations or population subgroups, including a focus on low and middle income country (LMIC) [35] populations or population subgroups, both), and types of outcomes (i. purchasing, ii. other behaviours, iii. proximal consequences, iv. distal consequences) by one reviewer (IS).

Due to the large number of included studies, the review team decided at this stage to prioritise studies of the effects of economic instruments (i.e. direct evidence for intervention effects) that included a focus on HIC populations or population subgroups for further, more detailed data collection and analysis. Data were collected from the latter set of studies using a pre-specified data extraction form, based in Microsoft Excel, which had been pilot-tested on a random sample of ten included studies. In addition to study characteristics collected from all included studies, we collected further information relating to primary study design (experimental, quasi-experimental, non-experimental, modelling), interventions (specific sub-types for each broad type of economic instrument), outcomes (details of specific measures within each broad category i-iv), and relevant principal findings and conclusions, the latter two being transcribed verbatim from full-text study reports. All data collection was undertaken and checked by one reviewer (IS). We did not plan or conduct assessments of risk of bias in included studies [36].

We did not plan or conduct statistical meta-analysis of the results of included studies. Summary statistics (frequencies and percentages) were calculated to inform development of a configuring synthesis of included studies in terms of their key characteristics and findings. This included developing narrative statements to summarise the evidence base for each broad type of intervention, based on the study characteristics data and transcribed principal findings and conclusions collected from the relevant subset of primary studies. It was important to base these summary statements on evidence from primary studies, rather than review articles, to avoid the risk of ‘multiple-counting’ of evidence derived from sets of primary studies that overlapped between included reviews. The cumulative evidence base was analysed and interpreted iteratively as the review proceeded through discussions involving all members of the multi-disciplinary review team (IS, GJH, TMM, RN, SAJ, MPK, MS and DO).

## Results

### Search results

**Figure 1:**
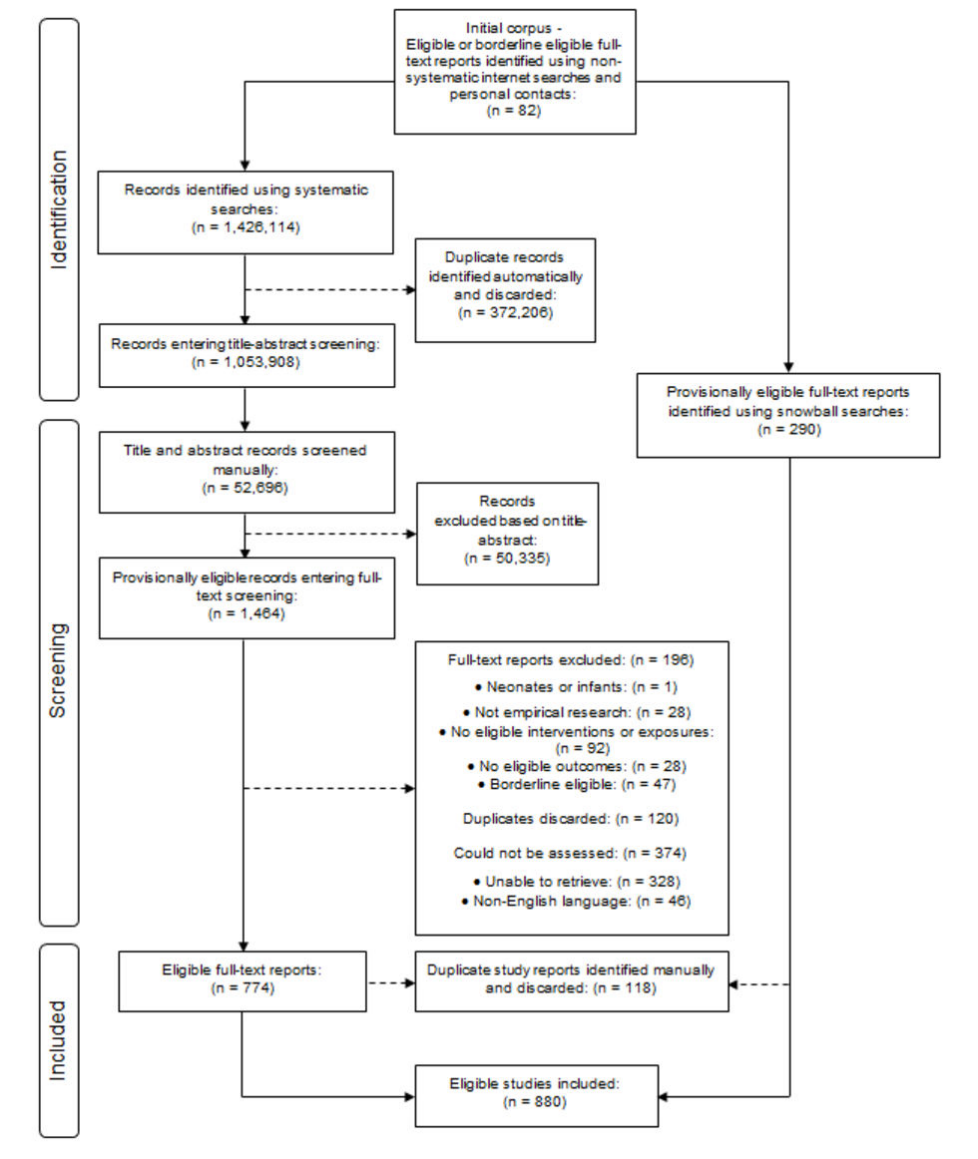
PRISMA flow diagram.

Figure 1 shows the flow of studies though each stage of the systematic scoping review process [26]. Systematic searches retrieved 1,426,032 title-abstract records. Following automatic de-duplication, 1,053,908 records remained. We manually screened a total of 52,695 title-abstract records (of which 46,580 were prioritised using text mining) and selected 1,464 records as provisionally eligible. Corresponding full-text reports were assessed for 970 provisionally eligible records. Of the remaining records assessed as provisionally eligible, 120 were identified as further duplicates and discarded, whilst 374 corresponding full-text reports could not be assessed either because they could not be retrieved (328 study reports) or because they were published in a language other than English (46 study reports); translation was beyond the scope of this review.

Based on full-text screening, 774 study reports met final eligibility criteria and were selected for inclusion in the review, whilst the remaining 196 were excluded for reasons shown in Figure 1. Parallel snowball searches identified 290 full-text study reports that met final eligibility criteria, of which 118 were discarded because they duplicated study reports identified via systematic searches and text mining prioritised screening. Once multiple reports of the same study had been linked, 880 eligible studies were included in the review (comprising 946 study reports). Bibliographies of all included studies, study reports excluded based on full-text screening and those for which the full-text could not be assessed are provided as Supporting Information files (Bibliographies S1).

The final set of 880 eligible studies comprised 192 studies of the effects of economic instruments (i.e. intervention studies, of which 181 included a focus on HICs) and 768 studies (including 86 intervention studies) that included evidence for prices or income as correlates or determinants of target outcomes (i.e. studies of exposure to general variations in prices or income, of which 610 included a focus on HICs). In this article, our predominant focus is on summarising evidence from the 181 intervention studies that included a focus on HICs and were prioritised for detailed data collection and analysis (intervention studies – direct evidence). We also briefly describe the body of 610 studies that included evidence for prices or income as correlates or determinants of target outcomes (studies of exposures – indirect evidence) and included a focus on HICs.

### Evidence from intervention studies

Table S2 (a Supporting Information file) summarises key characteristics of the 181 studies of the effects of economic instruments that included a focus on HIC populations or population subgroups. Table 1 and Table 2 show the overall distributions of evidence for the effects of broad categories and specific sub-types of economic instruments on (respectively) diet-related and physical activity-related outcomes across those 181 studies. Four broad categories of interventions were encountered across these 181 studies: price promotions, taxes, supply-side subsidies and transfer payments. No eligible studies of direct unit pricing legislation were identified. For some intervention types, the identified evidence base comprised more reviews than primary studies. There was much more evidence concerning diet-related outcomes than physical activity. The large majority of intervention studies (87%, 158 of 181) included a focus on United States (US) populations or population subgroups, whilst six per cent (11 of 181) included a focus on United Kingdom (UK) populations or population subgroups.

**Table 1:** Studies of interventions to promote diet.

Intervention category	Subsidiary intervention type	Primary studies (n)	Reviews (n)
Price promotions (n=34)	Simple discounts - price restructuring in discrete settings	13	17
	Multi-buy deals	2	1
	Price-pack deals	0	1
	Price deals	0	0
	Introductory pricing	0	0
	Couponing	1	1
	Rebates	0	0
Taxes (n=56)	Agricultural commodity tax	0	0
	Fat tax	8	5
	Snack tax	5	7
	Calorie tax	9	11
	Soft drinks tax	18	12
	Sugar tax	3	2
	Tax exemptions	3	1
Supply-side subsidies (n=47)	Agricultural commodities	0	3
	Agricultural commodities – subsidy removal	1	2
	School meals	20	4
	Other meals	1	0
	Healthy foods	9	7
	Healthy non-alcoholic beverages	3	6
	Specific nutrients	3	1
	Food transportation or delivery	1	1
Direct unit pricing legislation (n=0)	Minimum pricing legislation	0	0
	Maximum pricing legislation	0	0
Transfer payments (n=80)	Restricted income transfers, welfare benefits or welfare assistance programs	62	16
	Unrestricted income transfers, welfare benefits or welfare assistance programs	3	1
	Tax credits	0	0

**Table 2:** Studies of interventions to promote physical activity.

Intervention category	Subsidiary intervention type	Primary studies (n)	Reviews (n)
Price promotions (n=3)	Any type of price promotion	0	3
	Simple discounts - price restructuring in discrete settings	0	0
	Multi-buy deals	0	0
	Price-pack deals	0	0
	Price deals	0	0
	Introductory pricing	0	0
	Couponing	0	0
	Rebates	0	0
Taxes (n=3)	Tax exemptions	0	1
	Congestion tax	2	1
Supply-side subsidies (n=1)	Physical activity products	0	0
	Physical activity services (programs)	0	1
Direct unit pricing legislation (n=0)	Minimum pricing legislation	0	0
	Maximum pricing legislation	0	0
Transfer payments (n=5)	Restricted income transfers, welfare benefits or welfare assistance programs	2	0
	Unrestricted income transfers, welfare benefits or welfare assistance programs	0	0
	Tax credits	1	2

Table S3 (a Supporting Information file) summarises evidence for the reported effects of each broad category of intervention, based on analysis of primary study characteristics (see Table **S2**) combined with examination of transcribed principal findings and conclusions across the relevant subset of primary studies. Overall, evidence for associations between each type of economic instrument and target diet-related outcomes was largely equivocal. By equivocal we mean that few consistent patterns of reported findings could be identified between heterogenous sets of primary studies of each type of economic instrument with respect to the directions of their associations with (or effects on) diet-related behaviours or corollary outcomes. An exception was evidence from a small, relatively homogenous cluster of prospective quasi-experimental studies conducted in US populations, which found that simple discounts to reduce the unit retail prices of healthy foods and drinks in school or workplace vending machines or cafeterias was associated with increased purchasing of those foods; see, for example [37-39]. We identified few studies of other types of price promotion such as multi-buy deals, price-pack deals, price deals, introductory pricing, couponing or rebates (three primary studies and three reviews).

Although a substantial body of evidence for the effects of taxes or supply-side subsidies on specific foods, drinks or nutrients (food-related taxes or subsidies) was identified, few studies measured behavioural responses to real interventions using experimental or robust quasi-experimental study designs. This is largely attributable to the fact that relatively few governments or legislatures outside the US have implemented such interventions to date [5]. Instead, the prevailing approach involved simulating predicted effects of hypothetical tax or subsidy policy scenarios based on demand elasticities derived from purchasing data. For example, Andreyeva and colleagues estimated the predicted effects of a US national one cent-per-ounce tax on sugar contained in sugar sweetened beverages (SSBs) by constructing a model that projected future beverage consumption based on recent regional beverage consumption data, historic trends in beverage consumption and estimates of the price elasticity of SSB demand [40].

Studies of food-related taxes or subsidies collectively assessed a large and diverse set of specific outcomes. For example, the impacts of such taxes have been modelled in terms of ≈800 specific measures across included primary studies, only ≈5% of which were assessed in more than one study. Modelled tax rates were small compared with those typically applied to tobacco or alcohol products and were generally predicted to have small-to-moderate beneficial effects on purchasing, or negligible effects on body weight. Several authors concluded that higher tax or subsidy rates than those considered in their studies might need to be imposed to have a meaningful impact on target outcomes. Although most studies of food-related taxes modelled compensatory purchasing, whereby consumers substitute within or between taxed and untaxed sets of products, the range of substitute or complementary products incorporated into analyses was inevitably limited relative to the vast array of potential alternative food and beverage products available to consumers. For example, Andreyeva and colleagues highlighted that their prediction that estimated tax-induced reductions in SSB purchasing “could translate into losses in average body weight [of] up to 5 lbs/year... is certainly an upper bound given potential substitution to other caloric beverages and foods” and that “reliable estimates of the cross-price elasticities necessary to quantify the extent of possible substitution and the net impact on caloric intake are not available.” [40]. Furthermore, few studies modelled potential supply-side responses, such as product reformulation to avoid taxes, or adjustment of retail prices to limit or amplify the extent to which the price effects of taxes or subsidies are passed through to consumers in the form of changes in relative unit prices at the checkout. Several authors highlighted, but had rarely demonstrated, the potentially regressive nature of food-related taxes.

The largest subset of studies of transfer payments involved analyses of large, observational datasets to investigate the impacts of US nutrition and federal food assistance programs (63 primary studies and 13 reviews), principally the Supplemental Nutrition Assistance Program (SNAP – formerly known as the Food Stamp Program). SNAP provides financial assistance for food purchasing to people and families on low or no incomes by income transfer via an Electronic Benefit Transfer (debit card) system. Use of SNAP benefits is generally restricted to the purchase of foods or food products for consumption at home, such as breads and cereals, fruits and vegetables, meats, fish and poultry and dairy products [41]. Studies of transfer payments assessed large numbers of outcomes and typically reported mixed patterns of results. For example, Fox and colleagues conducted a secondary analysis of observational data collected using the Third National Health and Nutrition Examination Survey (NHANES-III) to investigate differences between participants in the US federal SNAP and two comparison groups of non-participants (income-eligible and higher income non-participants). Between-group differences were assessed in terms of (inter alia) frequency of meal consumption, seven measures of physical activity, dietary intake of nine key nutrients and dietary components, overall dietary quality (based on Healthy Eating Index total and component scores), and eight measures of physiological or metabolic risk factors for non-communicable disease (or related biomarkers), and the study found a mixed pattern of results across these outcomes in terms of both the existence and the direction of statistically significant differences between groups [42]. Two other US programs studied were the National School Lunch Program (a federally assisted meal program that pays subsidies to public and non-profit private schools and residential child care institutions to support the provision of reduced price or free lunches to children each school day [43]) and the School Breakfast Program (a federally assisted meal program that provides subsidies to participant States to operate non-profit breakfast programs, in which breakfasts are provided at a reduced price in schools and residential childcare institutions [44]). Primary studies that assessed the impacts of participation in these programs – classified as studies of supply-side subsidies, alongside the smaller group of modelling studies that simulated the predicted effects of supply-side subsidies applied to healthier foods, non-alcoholic beverages or nutrients (described above and in Table S3) – were also typically characterised by large numbers of outcome measures and mixed patterns of results.

In summary, our analysis indicates that the public health case for using economic instruments to promote dietary behaviour change depends largely on evidence from US studies; from studies that vary in the degree to which they support causal inferences; and from studies modelling predicted (rather than actual) behavioural responses, which inevitably rely on simplifications and a variety of more or less credible assumptions. Moreover, there is a general lack of evidence to inform the case for or against the use of economic instruments to promote physical activity (see Tables 1, 2, S2 and S3). Overall, we identified two primary studies that assessed congestion charges (taxes) [45,46]; two that assessed employer-sponsored benefit schemes (transfer payments) to promote employees’ use of, respectively, health clubs and public transport [47,48]; and one that evaluated tax credits (transfer payments) provided to parents who had enrolled their children in organised physical activity programmes [49]. This finding is consistent with, and adds little to, those of the six published reviews we identified that included coverage of the use of economic instruments to promote physical activity [50-55].

Further specific types of eligible interventions were encountered in three studies that included investigation of the effects of cigarette taxes [56-58] (of which one also considered a gasoline tax [58] and one also considered a tax on beer [56]), and two studies of the effects of housing [59] and childcare [60] subsidies. Outcomes assessed in these five studies comprised various measures of physical activity [56] and measures of body weight or body weight status [57-60]. These specific interventions are not included in Table 1 or Table 2 because the pathways by which they may influence these outcomes are likely to differ considerably from those of the other specific intervention types within the corresponding broader category (taxes and supply-side subsidies, respectively). For example, Mellor and colleagues propose a causal pathway by which cigarette taxes increase cigarette prices and costs, reduce smoking and increase food expenditures and consumption in the household, leading to increased likelihood of childhood obesity [57].

Across diet and physical activity, few studies of economic instruments included assessment of long-term effects and few incorporated formal economic evaluation. Whilst several studies of some specific types of intervention (e.g. food-related taxes and subsidies, subsidised school meals, or transfer payments restricted for use to purchase foods) incorporated assessments of distributions of effects by population sub-groups, such assessments were less frequently conducted for other intervention types. In addition, many included studies of economic instruments that are reliant on behaviour change as the pathway for modifying physiological or metabolic risk factors did not assess intermediate behavioural endpoints at all.

### Evidence from studies of general exposure to variations in prices or income

610 studies conducted in HICs incorporated evidence for price or income levels as correlates or determinants of target outcomes. This body of evidence bears indirectly on the public health case for using economic instruments because such instruments are proposed to operate via price and income mechanisms to influence the target behaviours (e.g. price promotions change the relative unit retail prices of foods, and prices are, in turn, a determinant of food choices [61]). This evidence base was characterised by heterogeneity of study designs and of the populations, exposures, outcomes, and covariates assessed. However, a clear inference from reviewing this set of studies is that the relationships between prices or income and target outcomes may often be non-linear, moderated by a wide range of modifiable and non-modifiable factors and mediated by complex mechanisms of action. Also, many studies reported null associations or statistically significant associations in the opposite direction to that anticipated. Whilst further synthesis of evidence drawn from subsets of studies within this large body of literature may provide some useful pointers for policy design, it primarily serves to highlight that hasty generalisation about the causal pathways linking changes in prices of diet- and physical activity-related products or services and income to behavioural and health outcomes is not warranted.

## Discussion

### Implications for policy and research

Current evidence for the effects of economic instruments on diet- and physical activity-related outcomes is limited in terms of the potential for causal inference and yields equivocal findings. The relative lack of direct evidence for the effects of economic instruments on physical activity is striking. Whilst direct evidence for effects on diet is much more prevalent, the dearth of experimental studies and inconsistency in findings between studies of the same types of interventions in terms of the specific outcomes they have assessed are key limitations of this cumulative evidence base.

Whilst economic instruments have been suggested in several studies to hold promise, mixed patterns of findings for most intervention types are likely to reflect the heterogeneous evidence base, as well as the complexity of behavioural responses to economic stimuli and of the causal pathways involved. This suggests a need for caution in developing policy based on limited evidence and overly simple assumptions about how people will respond to changes in prices and income. It does not necessarily imply that underlying economic theory, which holds that people respond rationally to incentives, or behavioural economic theory, which holds that rationality of choice is moderated by heuristics and biases attributable to various social, cognitive, and emotional factors [62], are flawed. Rather, it is likely that people’s responses to, say, a tobacco control tax are relatively predictable, whereas their responses to, say, a tax-stimulated increase in the prices of specific foods, relative to the vast array of alternative foods available, are less predictable and more complex in their relationships to health behaviours and corollary outcomes.

Crucially, our findings highlight the need to implement interventions in order to subject the logic models and programme theories involved to much closer scrutiny than they have been afforded in intervention research in these fields to date. In particular, people’s actual behavioural responses to interventions should be measured using prospective or ex-post evaluation studies capable of generating reliable causal inferences that increase our understanding of the conditions under which economic incentives work, as well as the reasons they may sometimes produce counter-intuitive behavioural responses [63]. So far as recent policy interest translates into implementation of interventions designed to change prices or income, this represents an important opportunity for evaluations of public health impacts to be conducted. However, to maximise these opportunities, implementation strategies will need to be carefully aligned with evaluation planning and design.

In order to support causal inferences about the effects of interventions, there is a further need for future intervention studies to include reliable measures of impacts at different stages of proposed causal pathways between interventions and intermediate or final health outcomes [64]. Where possible, for example, studies of economic instruments intended to alter people’s body weight, by changing first their food purchasing behaviour and then their food and nutrient intake, should include measures of both purchasing and intake alongside measures of body weight. However, we also recognise that intervention studies alone are unlikely to generate all the evidence needed to unpack all of the links in causal chains of effects. The limited feasibility of conducting intervention studies with sufficient length of follow-up to allow for measurement of long-term effects makes it likely that quantification of relationships between behavioural and proximal endpoints and final health outcomes (principally, mortality and morbidity associated with NCDs) will remain largely within the purview of observational epidemiology. Therefore, when the ultimate goal of intervention is to improve population health, the study of interventions that work via price or income mechanisms will usually require synthesis of evidence from both intervention and epidemiological studies and use of modelling to extrapolate from intermediate to final health outcomes. Given the current inconsistency between studies in the specific outcomes they assess, development of the cumulative evidence base for the health-related effects of economic instruments (and other population-level interventions) would also benefit from international consensus on a core set of outcome measures to be routinely included in future surveys, primary studies and systematic reviews.

Our findings have identified a general lack of economic evaluation of interventions that may need to be addressed in future research if policy is to be informed by considerations of how to maximise population health within available resources. Future primary studies and systematic reviews would be further strengthened by explicit assessments of distributional effects between population subgroups and of potential trade-offs or synergies between improving diet and physical activity ‘on average’ and reducing health and economic inequalities [65]. Finally, the capacity of economic instruments to bring about sustained population-level shifts in diet and physical activity needs to be evaluated, given the potential for any beneficial effects to weaken as consumers become accustomed to altered relative prices or altered budget constraints.

### Strengths and limitations of the review

A major strength of this systematic scoping review is its broad scope. Eligibility criteria adopted by previous systematic reviews in this area are not as inclusive across all components of the PICOS framework, and none have adopted such a sensitive, broad-based approach to locating and selecting eligible studies. Our development and application of innovative text mining methods to support study selection from an initially broad set of >1 million retrieved records enabled us to assemble, configure and describe a large, multi-disciplinary evidence base for relevant interventions and exposures on a scale that few reviews have managed previously. This is important, because the design and implementation of behaviour change programmes have been hampered in the past by their being located in single disciplines. In parallel, we have configured typologies of interventions, delimited sets of outcomes and identified study-level limitations in ways that can support future primary and secondary research.

However, our inclusive, broad-based approach also engendered limitations that need to be considered when interpreting our findings. First, based on the baseline inclusion rate, we estimate that only ≈40% of title-abstract records expected to be selected for full-text screening were identified within available resources. It is acknowledged that the obligation to identify every eligible study may be relaxed to some extent in scoping reviews, since they typically prioritise conceptual breadth (the aim to assemble a range and distribution of eligible studies that is representative of the target evidence base in terms of key study characteristics) over depth (the aim to assemble all eligible studies) [66]. Nevertheless, the validity of our configuring synthesis is reliant on the range and distribution of included studies being representative of the unknown full set of eligible studies, which encompasses those represented in the ≈60% (2,205 of 3,669) title-abstract records that were expected to be selected as provisionally eligible but were not screened or analysed (as well as those 374 full-text study reports that could not be assessed because inter-library loans and translation of non-English language studies were beyond the scope of the review). Our parallel use of snowball searches, coupled with the observation that included reviews did not identify any further eligible types of study designs, interventions or exposures not captured in this review, may ameliorate concern about this limitation. Moreover, had we used conventional screening methods, we would have identified only ≈5% of provisionally eligible study records within available resources, compared with the ≈40% we were able to identify in practice, assisted by the use of text mining.

Second, the large absolute number of included studies (N=880) necessitated prioritisation of studies that included a focus on HIC populations for data collection and analysis (N=791), with the subset of intervention studies in HIC populations (N=181) prioritised for further, more detailed data collection and analysis. With limited scope to collect and analyse data from studies of general exposure to changes in prices or income in HIC populations, results derived from these studies should be viewed with caution. Furthermore, we did not generate any results pertaining to the effects of interventions or exposures on diet- or physical activity-related behaviours and corollary outcomes in LMIC populations. We have, however, assembled a large body of studies that comprise this evidence base for LMIC populations, which could, in principle, be synthesised in future systematic reviews.

In this scoping review, we did not plan to conduct formal risk-of-bias assessments, nor aggregative synthesis (such as meta-analysis) of the results of included studies. The scope for conducting formal risk-of-bias assessments was, in any case, limited by a lack of established methods for conducting such assessments on quasi-experimental and non-experimental study designs. The lack of formal aggregative synthesis of the results of included studies, which means we do not (and did not set out to) present pooled effect-sizes for economic instruments in terms of target outcomes, reflects differences between the objectives and characteristics of scoping reviews and those of systematic reviews, outlined in the introduction to this article. Clearly, reliable estimates of intervention effect-sizes are an important component of the basis for policy formulation, and meta-analysis of evidence for the effects of economic instruments will therefore form an important component of the design of subsequent systematic reviews that follow the preliminary, configuring synthesis performed in this systematic scoping review.

Finally, a further limitation of this scoping review was that a single reviewer completed the large majority of screening at the study selection stage. Whilst initial development of the scoping review protocol and judgements concerning potential revisions to provisional eligibility criteria and application of final eligibility criteria were based on discussions involving the wider review team and collective examination of examples drawn from the emerging body of eligible and borderline eligible studies, this limitation should be acknowledged. The ‘gold standard’ approach involves assessments of study eligibility being undertaken by at least two reviewers working independently, in order to reduce the possibility that eligible study reports will be discarded due to human error [34].

## Conclusion

Our findings have exposed a complex, limited and largely equivocal evidence base, suggesting that the public health case for using economic instruments to promote dietary and physical activity behaviour change may be less compelling than some proponents have claimed. This conclusion provides an important counterpoint to what are, in our view, overly optimistic claims made by some authors of individual primary studies and reviews for the use of economic instruments to improve population health behaviour. It implies a need for caution in the development of public health policies intended to alter economic environmental stimuli to incentivise health-enhancing dietary and physical activity behaviour change at population level. In particular, policy implementation needs to be carefully aligned with evaluation planning and design.

## Supporting Information

Bibliographies S1 **Bibliographic details of included studies, excluded studies, and studies not assessed.**
(DOCX)

Checklist S1 **PRISMA checklist.**
(DOCX)

Interventions S1 **Defintions of interventions.**
(DOCX)

Protocol S1 **Protocol for the systematic scoping review.**
(DOCX)

Search Strategies S1 **Search strategies used in electronic searches.**
(DOCX)

Table S1 **Electronic search dates and yields, by database.**
(DOCX)

Table S2 **Characteristics of included studies.**
(DOCX)

Table S3 **Brief summaries of evidence by intervention type.**
(DOCX)
