# Supplementary material for: Economic Instruments for Population Diet and Physical Activity Behaviour Change: A Systematic Scoping Review
Source: PLoS One. 2013 Sep 24;8(9):e75070. doi: 10.1371/journal.pone.0075070 (PMC3782495; doi:10.1371/journal.pone.0075070)
Supplement: Bibliographies S1 — Bibliographic details of included studies, excluded studies, and studies not assessed. (DOCX) [file pone.0075070.s001.docx]

**Bibliographies S1**

This Supporting Information file presents complete bibliographies of: included studies; study reports excluded based on full-text screening; and those for which the full-text could not be assessed.

**Included studies**

**Aasness 2003**Aasness J, Larsen ER (2003) Distributional effects of environmental taxes on transportation. Journal of Consumer Policy 26: 279-300.

**Abdulai 2004**
Abdulai A, Aubert D (2004) A cross-section analysis of household demand for food and nutrients in Tanzania. Agricultural Economics 31: 67-79.

**Abdulai 1999**Abdulai A, Jain DK, Sharma AK (1999) Household food demand analysis in India. Journal of Agricultural Economics 50: 316-327.

**Abdul-Rahman 2008**Abdul-Rahman MF (2008) The demand for physical activity: An application of Grossman's health demand model to the elderly population [Doctoral dissertation]. Columbus, OH, United States: Ohio State University.

**Abdus 2008**Abdus S, Cawley J (2008) Simulating the impact of a “fat tax” on body weight (Job market paper). Ithaca, NY: Cornell University.

**Adelaja 1997**Adelaja AO, Nayga RMJ, Lauderbach TC (1997) Income and racial differentials in selected nutrient intakes. American Journal of Agricultural Economics 79: 1452-1460.

**Adrian 1976**
Adrian J, Daniel R (1976) Impact of socioeconomic factors on consumption of selected food nutrients in the United States. American Journal of Agricultural Economics 58: 31-38.

**Agbola 2003**Agbola FW (2003) Estimation of food demand patterns in South Africa based on a survey of households. Journal of Agricultural and Applied Economics 35: 663-670.

**Agrawal 2007**Agrawal AW, Schimek P (2007) Extent and correlates of walking in the USA. Transportation Research: Part D: Transport and Environment 12: 548-563.

**Ahmed 1993**Ahmed F, Barua S, Mohiduzzaman M, Shaheen N, Bhuyan MAH, et al. (1993) Interactions between growth and nutrient status in school-age children of urban Bangladesh. American Journal of Clinical Nutrition 58: 334-338.

**Ahn 2011**Ahn S, Zhao H, Smith ML, Ory MG, Phillips CD (2011) BMI and lifestyle changes as correlates to changes in self-reported diagnosis of hypertension among older Chinese adults. Journal of the American Society of Hypertension 5: 21-30.

**Ahrens 2011**Ahrens W, Siani A, Bammann K, De H, Iacoviello L, et al. (2011) Introduction to the IDEFICS study - obesity prevalence in European children. International Journal of Obesity 35: s146.

**Ainsworth 2006**Ainsworth BE, Macera CA, Jones DA, Reis JP, Addy CL, et al. (2006) Comparison of the 2001 BRFSS and the IPAQ Physical Activity Questionnaires. Medicine and Science in Sports and Exercise 38: 1584-1592.

**Aitken 2008**Aitken R, King L, Bauman A (2008) A comparison of Australian families' expenditure on active and screen-based recreation using the ABS Household Expenditure Survey 2003/04. Australian & New Zealand Journal of Public Health 32: 238-245.

**Akil 2011**Akil L, Ahmad HA (2011) Effects of socioeconomic factors on obesity rates in four southern states and Colorado. Ethnicity & Disease 21: 58-62.

**Akin 1983**Akin JS, Guilkey DK, Popkin BM (1983) The school lunch program and nutrient intake: A switching regression analysis. American Journal of Agricultural Economics 65: 477-485.

**Akkary 2009**Akkary E, Nerlinger A, Yu S, Dziura J, Duffy AJ, et al. (2009) Socioeconomic predictors of weight loss after laparoscopic Roux-Y gastric bypass. Surgical Endoscopy 23: 1246-1251.

**Alaimo 2001**Alaimo K, Olson CM, Frongillo EAJ (2001) Low family income and food insufficiency in relation to overweight in US children: Is there a paradox? . Archives of Pediatrics & Adolescent Medicine 155: 1161-1167.

**Albert 2006**Albert MA, Glynn RJ, Buring J, Ridker PM (2006) Impact of traditional and novel risk factors on the relationship between socioeconomic status and incident cardiovascular events. Circulation 114: 2619-2626.

**Alberty 2009**Alberty R, Albertyova D, Ahlers I (2009) Distribution and correlations of non-high-density lipoprotein cholesterol in Roma and Caucasian children: The Slovak Lipid Community study. Collegium Antropologicum 33: 1015-1022.

**Alfonzo 2006**
Alfonzo L, Hanawa P, Hikaru (2006) Estimating food demand in paraguay from household survey data. Agricultural Economics 34: 243-257.

**al-Isa 1999**al-Isa AN (1999) Dietary and socio-economic factors associated with obesity among Kuwaiti college men. British Journal of Nutrition 82: 369-374.

**Al-Kloub 2010**Al-Kloub MI, Al-Hassan MA, Froelicher ES (2010) Predictors of obesity in school-aged Jordanian adolescents. International Journal of Nursing Practice 16: 397-405.

**Allais 2010**Allais O, Bertail P, Nichele V (2010) The effects of a fat tax on French households' purchases: A nutritional approach. American Journal of Agricultural Economics 92: 228-245.

**Alley 2006**Alley DE, Seeman TE, Ki KJ, Karlamangla A, Hu P, et al. (2006) Socioeconomic status and C-reactive protein levels in the US population: NHANES IV. Brain, Behavior, & Immunity 20: 498-504.

**Al-Mahroos 2001**Al-Mahroos F, Al-Roomi K (2001) Obesity among adult Bahraini population: Impact of physical activity and educational level. Annals of Saudi Medicine 21: 183-187.

**Al-Nuaim 1997**Al-Nuaim AA, Bamgboye EA, Al-Rubeaan KA, Al-Mazrou Y (1997) Overweight and obesity in Saudi Arabian adult population, role of socio- demographic variables. Journal of Community Health 22: 211-223.

**Alston 2010**Alston JM, Rickard BJ, Okrent AM (2010) Farm Policy and Obesity in the United States. Choices 25.

**Alston 2009**Alston JM, Mullally CC, Sumner DA, Townsend M, Vosti SA (2009) Likely effects on obesity from proposed changes to the US Food Stamp Program. Food Policy 34: 176-184.

**Alston 2008**Alston JM, Sumner DA, Vosti SA (2008) Farm subsidies and obesity in the United States: National evidence and international comparisons. Food Policy 33: 470-479.

**Alves 2009**Alves JGB, Siqueira PP, Figueiroa JN (2009) Excesso de peso e inatividade fisica em criancas moradoras de favelas na regiao metropolitana do Recife, PE [Overweight and physical inactivity in children living in favelas in the metropolitan region of Recife, Brazil]. Jornal de Pediatria 85: 67-71.

**Amarasinghe 2009**Amarasinghe A, D'Souza G, Brown C, Oh H, Borisova T (2009) The influence of socioeconomic and environmental determinants on health and obesity: A West Virginia case study. International Journal of Environmental Research & Public Health 6: 2271-2287.

**Ammouri 2008**Ammouri AA (2008) Demographic differences in health promoting lifestyle of adult Jordanians. Jordan Medical Journal 42: 220-228.

**Ammouri 2007**Ammouri AA, Neuberger G, Nashwan AJ, Al-Haj AM (2007) Determinants of self-reported physical activity among Jordanian adults: Clinical scholarship. Journal of Nursing Scholarship 39: 342-348.

**Anderson 2010**Anderson AD (2010) African-American parents' nutritional habits: Implications for the prevention of early childhood obesity [Doctoral dissertation]. Denton, TX: University of North Texas.

**Anderson 1994**Anderson AS, Hunt K, Ford G, Finnigan F (1994) One apple a day? Fruit and vegetable intake in the West of Scotland. Health Education Research 9: 297-305.

**Anderson 2002**Anderson PM, Butcher KF, Levine PB (2002) Maternal employment and overweight children. Cambridge, MA: National Bureau of Economic Research.

**Andreyeva 2011**Andreyeva T, Chaloupka FJ, Brownell KD (2011) Estimating the potential of taxes on sugar-sweetened beverages to reduce consumption and generate revenue. Preventive Medicine 52: 413-416.

**Andreyeva 2010**Andreyeva T, Long MW, Brownell KD (2010) The impact of food prices on consumption: A systematic review of research on the price elasticity of demand for food. American Journal of Public Health 100: 216-222.

**Arambepola 2007**Arambepola C, Ekanayake R, Fernando D (2007) Gender differentials of abdominal obesity among the adults in the district of Colombo, Sri Lanka. Preventive Medicine 44: 129-134.

**Arcia 1990**Arcia GJ, Crouch LA, Kulka RA (1990) Impact of the WIC Program on Food Expenditures. American Journal of Agricultural Economics 72: 218-226.

**Ardern 2007**Ardern CI, Katzmarzyk PT (2007) Geographic and demographic variation in the prevalence of the metabolic syndrome in Canada. Canadian Journal of Diabetes 31: 34-46.

**Arnoult 2008**Arnoult MH, Tiffin R, Traill WB (2008) Models of nutrient demand, tax policy & public health impact. Reading, UK: University of Reading, Research Councils UK, Rural Economy and Land Use. 03 03.

**Arsenault 2003**Arsenault JE, Brown KH (2003) Zinc intake of US preschool children exceeds new dietary reference intakes. American Journal of Clinical Nutrition 78: 1011-1017.

**Asfaw 2007**Asfaw A (2007) Do government food price policies affect the prevalence of obesity? Empirical evidence from Egypt. World Development 35: 687-701.

**Atlantis 2009**Atlantis E, Lange K, Wittert GA (2009) Chronic disease trends due to excess body weight in Australia. Obesity Reviews 10: 543-553.

**Attanasio 2005**Attanasio O, Mesnard A (2005) The impact of a conditional cash transfer programme on consumption in Colombia. Fiscal Studies 27: 421-442.

**Auld 2009**Auld MC, Powell LM (2009) Economics of food energy density and adolescent body weight. Economica 76: 719-740.

**Ayala 2005**Ayala GX, Mueller K, Lopez-Madurga E, Campbell NR, Elder JP (2005) Restaurant and food shopping selections among Latino women in Southern California. Journal of the American Dietetic Association 105: 38-45.

**Ayala 2004**Ayala GX, Elder JP, Campbell NR, Slymen DJ, Roy N, et al. (2004) Correlates of body mass index and waist-to-hip ratio among Mexican women in the United States: implications for intervention development. Womens Health Issues 14: 155-164.

**Azevedo 2008**Azevedo MR, Horta BL, Gigante DP, Victora CG, Barros FC (2008) Factors associated to leisure-time sedentary lifestyle in adults of 1982 birth cohort, Pelotas, Southern Brazil. Revista de Saude Publica 42 70-77.

**Babey 2010**Babey SH, Hastert TA, Wolstein J, Diamant AL (2010) Income disparities in obesity trends among California adolescents. American Journal of Public Health 100: 2149-2155.

**Babey 2009**Babey SH, Hastert TA, Huang W, Brown RE (2009) Sociodemographic, family, and environmental factors associated with active commuting to school among US adolescents. Journal of Public Health Policy 30 s203-s220.

**Bahonar 2010**Bahonar A, Sarrafzadegan N, Kelishadi R, Ramezani MA, Gharipour M, et al. (2010) Relationship between socioeconomic profiles with cardiovascular risk factors in Iran: The IHHP study. Circulation 122 e186.

**Bai 2010**Bai J, Wahl TI, Lohmar BT, Huang J (2010) Food away from home in Beijing: Effects of wealth, time and 'free' meals. China Economic Review 21: 432-441.

**Balistreri 2009**Balistreri KS, Van Hook J (2009) Socioeconomic status and body mass index among Hispanic children of immigrants and children of natives. American Journal of Public Health 99: 2238-2246.

**Ball 2005**Ball K, Crawford D (2005) Socioeconomic status and weight change in adults: A review. Social Science & Medicine 60: 1987-2010.

**Ballew 2001**Ballew C, Bowman BA, Sowell AL, Gillespie C (2001) Serum retinol distributions in residents of the United States: Third National Health and Nutrition Examination Survey, 1988-1994. American Journal of Clinical Nutrition 73: 586-593.

**Baltrus 2010**Baltrus PT, Shim RS, Ye J, Watson L, Davis SK (2010) Socioeconomic position, stress, and cortisol in relation to waist circumference in African American and white women. Ethnicity & Disease 20: 376-382.

**Baltrus 2007**Baltrus PT, Everson-Rose SA, Lynch JW, Raghunathan TE, Kaplan GA (2007) Socioeconomic position in childhood and adulthood and weight gain over 34 years: The Alameda County Study. Annals of Epidemiology 17: 608-614.

**Banovic 2006**Banovic M, Barreira MM, Fontes MA (2006) Portuguese household food expenditure: 1990, 1995 and 2000. New Medit: Mediterranean Journal of Economics, Agriculture and Environment 5: 25-31.

**Baquero 2010**Baquero BI (2010) Macro, meso and cultural exchange level of influence on obesity among Mexican immigrants and Mexican-Americans living on the US-Mexico border in Southern California [Doctoral dissertation]. San Diego, CA, United States: University of California, San Diego.

**Barnett 2008**Barnett TA, Gauvin L, Craig CL, Katzmarzyk PT (2008) Distinct trajectories of leisure time physical activity and predictors of trajectory class membership: A 22 year cohort study. International Journal of Behavioral Nutrition and Physical Activity 5.

**Barreto 2003**Barreto SM, Passos VM, Lima-Costa MF (2003) Obesidade e baixo peso entre idosos brasileiros. Projeto Bambuí [Obesity and underweight among Brazilian elderly: The Bambui Health and Aging Study]. Cadernos de saude publica / Ministerio da Saude, Fundacao Oswaldo Cruz, Escola Nacional de Saude Publica 19: 605-612.

**Barslund 2007**Barslund M (2007) Regional differences in food consumption in urban Mozambique: A censored demand system approach. Copenhagen: University of Copenhagen.

**Basiotis 1987**Basiotis PP, Johnson SR, Morgan KJ, Chen J-SA (1987) Food stamps, food costs, nutrient availability, and nutrient intake. Journal of Policy Modeling 9: 383-404.

**Baum 2011**Baum CL (2011) The effects of food stamps on obesity. Southern Economic Journal 77: 623-651. [*Primary reference*]

Baum CL (2007) The effects of food stamps on obesity Murfreesboro, TN: Middle Tennessee State University.

**Baum 2008**Baum C (2008) The effects of food stamp benefits on weight gained by expectant mothers. Madison, WI: University of Wisconsin Madison Institute for Research on Poverty.

**Bauman 2011**Bauman A, Ma G, Cuevas F, Omar Z, Waqanivalu T, et al. (2011) Cross-national comparisons of socioeconomic differences in the prevalence of leisure-time and occupational physical activity, and active commuting in six Asia-Pacific countries. Journal of Epidemiology and Community Health 65: 35-43.

**Behrman 1989**Behrman JR, Deolalikar AB (1989) Is variety the spice of life? Implications for calorie intake. Review of Economics and Statistics 71: 666-672.

**Bell 1999**Bell DR, Chiang J, Padmanabhan V (1999) The decomposition of promotional response: An empirical generalization. Marketing Science 18: 504-526.

**Bennett 2006**Bennett GG, Wolin KY, Puleo E, Emmons KM (2006) Pedometer-determined physical activity among multiethnic low-income housing residents. Medicine and Science in Sports and Exercise 38: 768-773.

**Bere 2008**Bere E, van Lenthe F, Klepp K-I, Brug J (2008) Why do parents' education level and income affect the amount of fruits and vegetables adolescents eat? European Journal of Public Health 18: 611-615.

**Bergman 2010**Bergman P, Grjibovski AM, Hagströmer M, Patterson E, Sjöström M (2010) Congestion road tax and physical activity. American Journal of Preventive Medicine 38: 171-177.

**Bergtold 2004**Bergtold JS, Akobundu E, Peterson EB (2004) The FAST method: Estimating unconditional demand elasticities for processed foods in the presence of fixed effects. Journal of Agricultural and Resource Economics 29: 276-295. [*Primary reference*]

Bergtold JS, Akobundu E, Peterson EB (2003) The FAST method: Estimating unconditional demand elasticities for processed foods in the presence of fixed effects. Annual meeting of the American Agricultural Economics Association. Montreal, Quebec: Virginia Polytechnic Institute and State University. pp. 30.

**Berrigan 2006**Berrigan D, Troiano RP, McNeel T, Disogra C, Ballard-Barbash R (2006) Active transportation increases adherence to activity recommendations. American Journal of Preventive Medicine 31: 210-216.

**Berrigan 2003**Berrigan D, Dodd K, Troiano RP, Krebs-Smith SM, Ballard B (2003) Patterns of health behavior in U.S. adults. Preventive Medicine 36: 615-623.

**Bersamin 2009**Bersamin A, Hanni KD, Winkleby MA (2009) Predictors of trying to lose weight among overweight and obese Mexican-Americans: A signal detection analysis. Public Health Nutrition 12: 64-73.

**Bertail 2008**Bertail P, Caillavet F (2008) Fruit and vegetable consumption patterns: A segmentation approach. American Journal of Agricultural Economics 90: 827-842.

**Beydoun 2008a**
Beydoun MA, Powell LM, Wang Y (2008a) The association of fast food, fruit and vegetable prices with dietary intakes among US adults: Is there modification by family income? Social Science & Medicine 66: 2218-2229. [*Primary reference*]

Beydoun MA, Powell LM, Chen X, Wang Y (2011) Food prices are associated with dietary quality, fast food consumption, and body mass index among U.S. children and adolescents. The Journal of Nutrition 141: 304-311.

**Bezerra 2010**Bezerra IN, Sichieri R (2010) Characteristics and spending on out-of-home eating in Brazil. Revista de Saude Publica 44: 221-229.

**Bhargava 2007**Bhargava A, Amialchuk A (2007) Added sugars displaced the use of vital nutrients in the National Food Stamp Program Survey. Journal of Nutrition 137: 453-460.

**Bhargava 1991**Bhargava A (1991) Estimating short and long run income elasticities of foods and nutrients for rural South India. Journal of the Royal Statistical Society: Series A (Statistics in Society) 154: 157-174.

**Bhattacharyya 2007**Bhattacharyya NG, Ogston SA, Mukherjee DK, Bandopadhyay S, Sabui TK, et al. (2007) Family income influences height and fat-free mass in girls not boys in Kolkata. Acta Paediatrica, International Journal of Paediatrics 96: 774-775.

**Billson 1999**Billson H, Pryer JA, Nichols R (1999) Variation in fruit and vegetable consumption among adults in Britain. An analysis from the dietary and nutritional survey of British adults. European Journal of Clinical Nutrition 53: 946-952.

**Biltoft-Jensen 2009**Biltoft-Jensen A, Groth MV, Matthiessen J, Wachmann H, Christensen T, et al. (2009) Diet quality: Associations with health messages included in the Danish Dietary Guidelines 2005, personal attitudes and social factors. Public Health Nutrition 12: 1165-1173.

**Binkley 2010**Binkley J, Golub A (2010) Household food choice in four food categories: Healthy or unhealthy? West Lafayette, IN: Purdue University. [*Primary reference*]

Binkley J, K., Golub A (2011) Consumer demand for nutrition versus taste in four major food categories. Agricultural Economics 42: 65-74.

**Binkley 2006**Binkley JK (2006) The effect of demographic, economic, and nutrition factors on the frequency of food away from home. Journal of Consumer Affairs 40: 372-391.

**Bitler 2004**Bitler M, Currie J (2004) Medicaid at birth, WIC take up, and children's outcomes: A final report to the IRP/USDA small grants program. Rand Corporation Publications Department.

**Bittencourt 2007**Bittencourt MVL, Teratanavat RP, Chern WS (2007) Food consumption and demographics in Japan: Implications for an aging population. Agribusiness 23: 529-551.

**Black 2010**Black JL, Macinko J (2010) The changing distribution and determinants of obesity in the neighborhoods of New York City, 2003-2007. American Journal of Epidemiology 171: 765-775.

**Black 2004**Black MM, Cutts DB, Frank DA, Geppert J, Skalicky A, et al. (2004) Special supplemental nutrition program for women, infants, and children participation and infants' growth and health: A multisite surveillance study. Pediatrics 114: 169-176.

**Blakely 2011**Blakely T, Ni Mhurchu C, Jiang Y, Matoe L, Funaki-Tahifote M, et al. (2011) Do effects of price discounts and nutrition education on food purchases vary by ethnicity, income and education? Results from a randomised, controlled trial. Journal of Epidemiology and Community Health.

**Blanck 2011**Blanck HM, Thompson OM, Nebeling L, Yaroch AL (2011) Improving fruit and vegetable consumption: Use of farm-to-consumer venues among US adults. Preventing Chronic Disease 8: A49.

**Blaylock 1982**Blaylock JR, Smallwood DM (1982) Analysis of income and food expenditure distributions: A flexible approach. Review of Economics and Statistics 64: 104-109.

**Blijham 2007**Blijham N, De Kan L, Niehof A (2007) Determinants and adequacy of food consumption of children in La Trinidad, the Philippines. International Journal of Consumer Studies 31: 195-203.

**Block 2010**Block JP, Chandra A, McManus Katherine D, Willett Walter C (2010) Point-of-purchase price and education intervention to reduce consumption of sugary soft drinks. American Journal of Public Health 100: 1427-1433.

**Bodea 2009**Bodea TD, Garrow LA, Meyer MD, Ross CL (2009) Socio-demographic and built environment influences on the odds of being overweight or obese: The Atlanta experience. Transportation Research: Part A: Policy and Practice 43: 430-444.

**Bodnar 2002**Bodnar LM, Siega-Riz AM (2002) A diet quality index for pregnancy detects variation in diet and differences by sociodemographic factors. Public Health Nutrition 5: 801-809.

**Bodor 2010**Bodor J, Rice J, Farley T, Swalm C, Rose D (2010) The association between obesity and urban food environments. Journal of Urban Health 87: 771-781.

**Boizot-Szantaï 2005**Boizot-Szantaï C, Etilé F (2005) The food prices/ body mass index relationship: Theory and evidence from a sample of French adults. 11th Congress of the European Association of Agricultural Economists. Copenhagen, Denmark: Institut National de la Recherche Agronomique

**Bonnet 2011a**
Bonnet C, Requillart V (2011a) Does the EU sugar policy reform increase added sugar consumption? An empirical evidence on the soft drink market. Health Economics 20: 1012-1024.

**Bonnet 2011b**
Bonnet C, Requillart V (2011b) Strategic pricing and health price policies. Toulouse, France: Toulouse School of Economics. 35 p.

**Boone-Heinonen 2010**Boone-Heinonen J, Evenson KR, Song Y, Gordon-Larsen P (2010) Built and socioeconomic environments: Patterning and associations with physical activity in U.S. adolescents. International Journal of Behavioral Nutrition and Physical Activity 7.

**Booth 2005**Booth KM, Pinkston MM, Poston WSC (2005) Obesity and the built environment. Journal of the American Dietetic Association 105: 110-117.

**Bopp 2006**Bopp M, Wilcox S, Laken M, Butler K, Carter RE, et al. (2006) Factors associated with physical activity among African-American men and women. American Journal of Preventive Medicine 30: 340-346.

**Borders 2006**Borders TF, Rohrer JE, Cardarelli KM (2006) Gender-specific disparities in obesity. Journal of Community Health 31: 57-68.

**Bose 2007**Bose K, Bisai S, Das P, Dikshit S, Pradhan S (2007) Inter-relationships of income, chronic energy deficiency, morbidity and hospitalization among adult male slum dwellers of Midnapore, West Bengal, India. Journal of Biosocial Science 39: 779-786.

**Boslaugh 2006**Boslaugh SE, Andresen EM (2006) Correlates of physical activity for adults with disability. Preventing Chronic Disease 3: 1-14.

**Bossan 2007**Bossan FM, Dos A, Luiz A, de Vasconcellos MTL, Wahrlich V (2007) Nutritional status of the adult population in Niteroi, Rio de Janeiro, Brazil: The nutrition, physical activity, and health survey. Cadernos de Saude Publica 23: 1867-1876.

**Bouis 1996**Bouis HE (1996) A food demand system based on demand for characteristics: If there is 'curvature' in the Slutsky matrix, what do the curves look like and why? Journal of Development Economics 51: 239-266.

**Boukouvalas 2009**Boukouvalas G, Shankar B, Bruce T (2009) Determinants of fruit and vegetable intake in England: A re-examination based on quantile regression. Public Health Nutrition 12: 2183-2191.

**Boumtje 2005**Boumtje PI, Huang CL, Lee J-Y, Lin B-H (2005) Dietary habits, demographics, and the development of overweight and obesity among children in the United States. Food Policy 30: 115-128.

**Bowman 2007**Bowman S (2007) Low economic status is associated with suboptimal intakes of nutritious foods by adults in the National Health and Nutrition Examination Survey 1999-2002. Nutrition Research 27: 515-523.

**Bowman 2006**Bowman SA (2006) Television-viewing characteristics of adults: Correlations to eating practices and overweight and health status. Preventing Chronic Disease 3: 1-11.

**Bowman 2004**Bowman SA, Gortmaker SL, Ebbeling CB, Pereira MA, Ludwig DS (2004) Effects of fast-food consumption on energy intake and diet quality among children in a national household survey. Pediatrics 113: 112-118.

**Boynton 2008**Boynton A, Neuhouser ML, Sorensen B, McTiernan A, Ulrich CM (2008) Predictors of diet quality among overweight and obese postmenopausal women. Journal of the American Dietetic Association 108: 125-130.

**Braveman 2010**Braveman PA, Cubbin C, Egerter S, Williams DR, Pamuk E (2010) Socioeconomic disparities in health in the United States: What the patterns tell us. American Journal of Public Health 100: s186-s196.

**Breckenkamp 2007**Breckenkamp J, Mielck A, Razum O (2007) Health inequalities in Germany: Do regional-level variables explain differentials in cardiovascular risk? BioMed Central Public Health 7: 132.

**Breuer 2010**
Breuer C, Hallmann K, Wicker P, Feiler S (2010) Socio-economic patterns of sport demand and ageing. European Reviews of Aging & Physical Activity 7: 61-70.

**Briefel 2009**Briefel RR, Wilson A, Gleason PM (2009) Consumption of low-nutrient, energy-dense foods and beverages at school, home, and other locations among school lunch participants and nonparticipants. Journal of the American Dietetic Association 109: s79-s90.

**Brophy 2011**Brophy S, Cooksey R, Lyons RA, Thomas NE, Rodgers SE, et al. (2011) Parental factors associated with walking to school and participation in organised activities at age 5: Analysis of the Millennium Cohort Study. BioMed Central Public Health 11: 14.

**Brophy 2009**Brophy S, Cooksey R, Gravenor MB, Mistry R, Thomas N, et al. (2009) Risk factors for childhood obesity at age 5: Analysis of the millennium cohort study. BioMed Central Public Health 9: 467.

**Brown 2009**Brown DE, Hampson SE, Dubanoski JP, Murai AS, Hillier TA (2009) Effects of ethnicity and socioeconomic status on body composition in an admixed, multiethnic population in Hawaii. American Journal of Human Biology 21: 383-388.

**Brown 1990**Brown DM (1990) The restaurant and fast food race: Who's winning? Southern Economic Journal 56: 984-995.

**Brown 1954**Brown JAC (1954) The consumption of food in relation to household composition and income. Econometrica 22: 444-460.

**Brownson 2006**Brownson RC, Haire-Joshu D, Luke DA (2006) Shaping the context of health: A review of environmental and policy approaches in the prevention of chronic diseases. Annual Review of Public Health 27: 341-370.

**Brug 2008**Brug J (2008) Determinants of healthy eating: Motivation, abilities and environmental opportunities. Family Practice 25: i50-i55.

**Bryan 2011**Bryan SN (2011) The epidemiology of physical activity in Canada [Doctoral dissertation]. Kingston, ON, Canada: Queen's University.

**Bryan 2009**Bryan SN, Katzmarzyk PT (2009) Patterns and trends in walking behaviour among Canadian adults. Canadian Journal of Public Health Revue Canadienne de Sante Publique 100: 294-298.

**Bryant 1981**Bryant W, Gerner JL (1981) Television use by adults and children: A multivariate analysis. Journal of Consumer Research 8: 154-161.

**Burstein 2000**Burstein NR, Fox MK, Hiller JB, Kornfeld R, Lam K, et al. (2000) WIC general analysis project: Profile of WIC children. Cambridge, MA: Abt Associates Inc.

**Butcher 2008**
Butcher K, Sallis JF, Mayer JA, Woodruff S (2008) Correlates of physical activity guideline compliance for adolescents in 100 U.S. Cities. Journal of Adolescent Health 42: 360-368.

**Butler 2007**Butler GP, Orpana HM, Wiens AJ (2007) By your own two feet: Factors associated with active transportation in Canada. Canadian Journal of Public Health Revue Canadienne de Sante Publique 98: 259-264.

**Butler 1985**Butler JS, Ohls JC, Posner B (1985) The effect of the Food Stamp Program on the nutrient intake of the eligible elderly. The Journal of Human Resources 20: 405-420.

**Buttet 2010**Buttet S, Dolar V (2010) A simple model of eating decisions and weight with rational and forward-looking agents. Cleveland, OH: Cleveland State University.

**Capacci 2011**Capacci S, Mazzocchi M (2011) Five-a-day, a price to pay: An evaluation of the UK program impact accounting for market forces. Journal of Health Economics 30: 87-98.

**Caraher 2007**Caraher M, Carr-Hill R (2007) Taxation and population health: "Sin taxes" or structured approaches. In: Galea S, editor. Macrosocial determinants of population health. New York, NY: Springer pp. 211-231.

**Caraher 2005**Caraher M, Cowburn G (2005) Taxing food: Implications for public health nutrition. Public Health Nutrition 8: 1242-1249.

**Casagrande 2007**Casagrande SS, Wang Y, Anderson C, Gary TL (2007) Have Americans increased their fruit and vegetable intake? The trends between 1988 and 2002. American Journal of Preventive Medicine 32: 257-263.

**Casey 2001**Casey PH, Szeto K, Lensing S, Bogle M, Weber J (2001) Children in food-insufficient, low-income families: Prevalence, health, and nutrition status. Archives of Pediatrics and Adolescent Medicine 155: 508-514.

**Cash 2007a**
Cash SB, Goddard E, Lacanilao RD (2007a) Fat taxes and health outcomes: An investigation of economic factors influencing obesity in Canada. Edmonton, AB: University of Alberta. CMD-07-09 CMD-07-09. 29 p.

Cash SB, Lacanilao RD (2007b) Taxing food to improve health: Economic evidence and arguments. Agricultural and Resource Economics Review 36: 174-182.

**Cassani 2009**Cassani RSL, Nobre F, Pazin F, Schmidt A (2009) Prevalencia de fatores de risco cardiovascular em trabalhadores de uma industria Brasileira [Prevalence of cardiovascular risk factors in a population of Brazilian industry workers]. Arquivos Brasileiros de Cardiologia 92: 15-21.

**Cawley 2011**Cawley J, Ruhm C (2011) The economics of risky health behaviors. Bonn, Germany: Forschungsinstitut zur Zukunft der Arbeit (IZA) Institute for the Study of Labor.

**Cawley 2010**Cawley J, Moran J, Simon K (2010) The impact of income on the weight of elderly Americans. Health Economics 19: 979-993. [*Primary reference*]

Cawley J, Moran JR, Simon KI (2008) The impact of income on the weight of elderly Americans. Cambridge, MA: National Bureau of Economic Research

**Cerin 2008**Cerin E, Leslie E (2008) How socio-economic status contributes to participation in leisure-time physical activity. Social Science & Medicine 66: 2596-2609. [*Primary reference*]

Cerin E, Leslie E, Owen N (2009) Explaining socio-economic status differences in walking for transport: An ecological analysis of individual, social and environmental factors. Social Science & Medicine 68: 1013-1020.

**Cervero 2003**Cervero R, Duncan M (2003) Walking, bicycling, and urban landscapes: Evidence from the San Francisco Bay Area. American Journal of Public Health 93: 1478-1483.

**Chad 2005**Chad KE, Reeder BA, Harrison EL, Ashworth NL, Sheppard SM, et al. (2005) Profile of physical activity levels in community-dwelling older adults. Medicine and Science in Sports and Exercise 37: 1774-1784.

**Chadarat 2006**Chadarat M, Tongkumchum P, Tongsinoot S (2006) Factors affecting high school overweight students in Pattani Province of Thailand. Pakistan Journal of Nutrition 5: 125-129.

**Chakraborty 2009**Chakraborty R, Bose K, Bisai S (2009) Relationship of family income and house type to body mass index and chronic energy deficiency among urban Bengalee male slum dwellers of Kolkata, India. Homo 60: 45-57.

**Chaloupka 2011**Chaloupka FJ, Powell LM, Chriqui JF (2011) Sugar-sweetened beverages and obesity: The potential impact of public policies. Journal of Policy Analysis and Management 30: 645-655.

**Chaloupka 2009a**
Chaloupka FJ, Powell LM, Chriqui J (2009a) Sugar-sweetened beverage taxes and public health. Princeton, NJ: Robert Wood Johnson Foundation.

**Chaloupka 2009b**
Chaloupka FJ, Powell LM (2009b) Price, availability, and youth obesity: Evidence from Bridging the Gap. Preventing Chronic Disease 6: A93.

**Chang 2005a**
Chang VW, Lauderdale DS (2005a) Income disparities in body mass index and obesity in the United States, 1971-2002. Archives of Internal Medicine 165: 2122-2128.

**Chang 2005b**
Chang VW, Christakis NA (2005b) Income inequality and weight status in US metropolitan areas. Social Science & Medicine 61: 83-96.

**Chaput 2006**Chaput JP, Brunet M, Tremblay A (2006) Relationship between short sleeping hours and childhood overweight/obesity: Results from the 'Quebec en Forme' project. International Journal of Obesity 30: 1080-1085.

**Chasan-Taber 2008**Chasan-Taber L, Schmidt MD, Pekow P, Sternfeld B, Solomon CG, et al. (2008) Predictors of excessive and inadequate gestational weight gain in Hispanic women. Obesity (Silver Spring) 16: 1657-1666.

**Chasan-Taber 2007**Chasan-Taber L, Schmidt MD, Pekow P, Sternfeld B, Manson J, et al. (2007) Correlates of physical activity in pregnancy among Latino women. Maternal and Child Health Journal 11: 353-363.

**Chavas 1983**Chavas J-P, Keplinger KO (1983) Impact of domestic food programs on nutrient intake of low-income persons in the United States. Southern Journal of Agricultural Economics 15: 155-163.

**Chavas 1982**Chavas J-P, Yeung ML (1982) Effects of the Food Stamp Program on food consumption in the Southern United States. Southern Journal of Agricultural Economics 14: 131-139.

**Chee 2004**Chee H-L, Kandiah M, Khalid M, Shamsuddin K, Jamaluddin J, et al. (2004) Body mass index and factors related to overweight among women workers in electronic factories in Peninsular Malaysia. Asia Pacific Journal of Clinical Nutrition 13: 248-254.

**Chen 2009**Chen J-L (2009) Household income, maternal acculturation, maternal education level and health behaviors of Chinese-American children and mothers. Journal of Immigrant and Minority Health 11: 198-204.

**Chen 2008**Chen Z, Meltzer D (2008) Beefing up with the Chans: Evidence for the effects of relative income and income inequality on health from the China Health and Nutrition Survey. Social Science & Medicine 66: 2206-2217.

**Chen 2005**Chen Z, Yen ST, Eastwood DB (2005) Effects of food stamp participation on body weight and obesity. American Journal of Agricultural Economics 87: 1167-1173.

**Chen 2002a**Chen S-N (2002a) Prices and health: Identifying the effects of nutrition, exercise, and medication choices on blood pressure. American Journal of Agricultural Economics 84: 990-1002. [*Primary reference*]

Chen S-N (2002b) Prices and health: Identifying the effects of nutrition and behavior choices on health in Taiwan. Taipei, Taiwan: Shih Hsin University.

**Chern 2002**Chern WS, Ishibashi K, Taniguchi K, Tokoyama Y (2002) Analysis of food consumption behavior by Japanese households. Rome, Italy: Agricultural Development Economics Division of the Food and Agriculture Organization of the United Nations.

**Choiniere 2000**Choiniere R, Lafontaine P, Edwards AC (2000) Distribution of cardiovascular disease risk factors by socioeconomic status among Canadian adults. Canadian Medical Association Journal 162: s13-s24.

**Chou 2002**Chou SY, Grossman M, Saffer H (2002) An economic analysis of adult obesity: Results from the behavioral risk factor surveillance system. Cambridge, MA: National Bureau of Economic Research.

**Chouinard 2007**Chouinard HH, Davis DE, LaFrance JT, Perloff JM (2007) Fat taxes: Big money for small change. Forum for Health Economics & Policy 10: 2. [*Primary reference*]

Chouinard HH, Davis DE, LaFrance JT, Perloff JM (2005) The effects of a fat tax on dairy products. Berkeley, CA: University of California, Berkeley.

**Chung 1994**Chung C-F (1994) A cross-section demand analysis of Spanish provincial food consumption. American Journal of Agricultural Economics 76: 513-521.

**Clarke 2007**Clarke D, McKenzie T (2007) Legislative interventions to prevent obesity in Pacific Island countries. Wellington, New Zealand: Allen & Clarke Policy and Regulatory Specialists Ltd.

**Claro 2010**Claro RM, Monteiro CA (2010) Family income, food prices, and household purchases of fruits and vegetables in Brazil. Revista de Saude Publica 44: 1014-1020.

**Claro 2007**Claro RM, Esvael do Carmo HC, Machado FMS, Monteiro CA (2007) Renda, preco dos alimentos e participacao de frutas e hortalicas na dieta [Income, food prices, and participation of fruit and vegetables in the diet]. Revista de Saude Publica 41: 557-564.

**Clunk 2001**Clunk JM, Lin CY, Curtis JJ (2001) Variables affecting weight gain in renal transplant recipients. American Journal of Kidney Diseases 38: 349-353.

**Coelho 2010**Coelho AB, de Aguiar DRD, Eales JS (2010) Food demand in Brazil: An application of Shonkwiler and Yen two-step estimation method. Estudos Economicos 40: 185-211.

**Cohen 2008**Cohen BE, Garg SK, Ali S, Harris WS, Whooley MA (2008) Red blood cell docosahexaenoic acid and eicosapentaenoic acid concentrations are positively associated with socioeconomic status in patients with established coronary artery disease: Data from the Heart and Soul Study. Journal of Nutrition 138: 1135-1140.

**Colchero 2008**Colchero MA, Caballero B, Bishai D (2008) The effect of income and occupation on body mass index among women in the Cebu Longitudinal Health and Nutrition Surveys (1983-2002). Social Science & Medicine 66: 1967-1978.

**Cole 2004**Cole N, Fox MK (2004) Nutrition and health characteristics of low-income populations: Volume II, WIC Program participants and nonparticipants. Washington, DC: US Department of Agriculture.

**Conde 2004**Conde WL, Monteiro CA, Moura EC, Popkin BM (2004) Socioeconomic status and obesity in adult populations of developing countries: A review. Bulletin of the World Health Organization 82: 940-947.

**Conen 2009**Conen D, Glynn RJ, Ridker PM, Buring JE, Albert MA (2009) Socioeconomic status, blood pressure progression, and incident hypertension in a prospective cohort of female health professionals. European Heart Journal 30: 1378-1384.

**Cook 2008**Cook A, Daponte B (2008) A demographic analysis of the rise in the prevalence of the US population overweight and/or obese. Population Research and Policy Review 27: 403-426.

**Cordell 2002**Cordell H, Betz CJ, Green GT (2002) Recreation and the environment as cultural dimensions in contemporary American society. Leisure Sciences 24: 13-41.

**Courtemanche 2011**Courtemanche C (2011) A silver lining? The connection between gasoline prices and obesity. Economic Inquiry 49: 935-957.

**Craig 2005**Craig CL, Cameron C, Bauman A (2005) Socio-demographic and lifestyle correlates of obesity Ottawa, ON: Canadian Institute for Health Information.

**Craig 2004**Craig CL, Russell SJ, Cameron C, Bauman A (2004) Twenty-year trends in physical activity among Canadian adults. Canadian Journal of Public Health 95: 59-63.

**Crawford 1995**Crawford PB, Obarzanek E, Schreiber GB, Barrier P, Goldman S, et al. (1995) The effects of race, household income, and parental education on nutrient intakes of 9- and 10-year-old girls NHLBI growth and health study. Annals of Epidemiology 5: 360-368.

**Crespo 2000**Crespo CJ, Smit E, Andersen RE, Carter-Pokras O, Ainsworth BE (2000) Race/ethnicity, social class and their relation to physical inactivity during leisure time: Results from the Third National Health and Nutrition Examination Survey, 1988-1994. American Journal of Preventive Medicine 18: 46-53.

**Cummins 2006**Cummins S, Macintyre S (2006) Food environments and obesity—neighbourhood or nation? International Journal of Epidemiology 35: 100-104.

**Curhan 1974**Curhan RC (1974) The effects of merchandising and temporary promotional activities on the sales of fresh fruits and vegetables in supermarkets. Journal of Marketing Research 11: 286-294.

**Currie 2003**Currie J (2003) U.S. food and nutrition programs. In: Moffitt RA, editor. Means-tested transfer programs in the United States. Chicago, IL: University of Chicago Press.

**da Costa 2007**da Costa JS, Barcellos FC, Sclowitz ML, Sclowitz IK, Castanheira M, et al. (2007) Hypertension prevalence and its associated risk factors in adults: a population-based study in Pelotas. Arquivos Brasileiros de Cardiologia 88: 59-65.

**da Silveira 2010**da Silveira VMF, Horta BL, Gigante DP, Azevedo J, Mario R (2010) Metabolic syndrome in the 1982 Pelotas cohort: effect of contemporary lifestyle and socioeconomic status. Arquivos Brasileiros de Endocrinologia e Metabologia 54: 390-397.

**Dahly 2010**Dahly D, L., Gordon-Larsen P, Popkin BM, Kaufman JS, Adair LS (2010) Associations between multiple indicators of socioeconomic status and obesity in young adult Filipinos vary by gender, urbanicity, and indicator used. Journal of Nutrition 140: 366-370.

**Dallongeville 2005**Dallongeville J, Cottel D, Ferrieres J, Arveiler D, Bingham A, et al. (2005) Household income is associated with the risk of metabolic syndrome in a sex-specific manner. Diabetes Care 28: 409-415.

**Darmon 2008**Darmon N, Drewnowski A (2008) Does social class predict diet quality? The American Journal of Clinical Nutrition 87: 1107-1117.

**Dastgiri 2006**Dastgiri S, Mahdavi R, TuTunchi H, Faramarzi E (2006) Prevalence of obesity, food choices and socio-economic status: A cross-sectional study in the north-west of Iran. Public Health Nutrition 9: 996-1000.

**Davis 1985**Davis MA, Randall E, Forthofer RN, Lee ES, Margen S (1985) Living arrangements and dietary patterns of older adults in the United States. Journal of Gerontology 40: 434-442.

**Davis 1979**Davis CG, Neenan PH (1979) Impact of food stamp and nutrition education programs on food group expenditure and nutrient intake of low income households. Southern Journal of Agricultural Economics 11: 121-129.

**de Andrade 2010**de Andrade RG, Pereira RA, Sichieri R (2010) Ten-year increase in the prevalence of obesity and reduction in fat intake in Brazilian women aged 35 years and older. Journal of Epidemiology and Community Health 64: 252-254.

**de Bem Lignani 2011**de Bem Lignani J, Sichieri R, Burlandy L, Salles-Costa R (2011) Changes in food consumption among the Programa Bolsa Familia participant families in Brazil. Public Health Nutrition 14: 785-792.

**De Maio 2009**De Maio FG, Linetzky B, Virgolini M (2009) An average/deprivation/inequality (ADI) analysis of chronic disease outcomes and risk factors in Argentina. Population Health Metrics 7.

**de Souza 2003**
de Souza LJ, Filho JTDS, de Souza TF, Reis AFF, Neto CG, et al. (2003) Prevalencia de Dislipidemia e Fatores de Risco em Campos dos Goytacazes [Prevalence of dyslipidemia and risk factors in Campos dos Goytacazes, in the Brazilian state of Rio de Janeiro]. Arquivos Brasileiros de Cardiologia 81: 249-264.

**Dean 2009**Dean M, Raats MM, Grunert KG, Lumbers M (2009) Factors influencing eating a varied diet in old age. Public Health Nutrition 12: 2421-2427.

**Deaton 1990**Deaton A (1990) Price elasticities from survey data: Extensions and Indonesian results. Journal of Econometrics 44: 281-309.

**Dellava 2010**Dellava JE, Bulik CM, Popkin BM (2010) Price changes alone are not adequate to produce long-term dietary change. Journal of Nutrition 140: 1887-1891.

**DellaVigna 2006**DellaVigna S, Malmendier U (2006) Paying not to go to the gym. American Economic Review 96: 694-694.

**Der 1999**Der G, MacIntyre S, Ford G, Hunt K, West P (1999) The relationship of household income to a range of health measures in three age cohorts from the West of Scotland. European Journal of Public Health 9: 271-277.

**Deshmukh-Taskar 2007**Deshmukh-Taskar P, Nicklas TA, Yang S-J, Berenson GS (2007) Does food group consumption vary by differences in socioeconomic, demographic, and lifestyle factors in young adults? The Bogalusa Heart study. Journal of the American Dietetic Association 107: 223-234.

**Devaney 1993**Devaney B, Gordon A, Burghardt J (1993) The school nutrition dietary assessment study: Dietary intakes of program participants and nonparticipants. Alexandria, VA: US Department of Agriculture Food and Nutrition Service.

**Devaney 1991**Devaney B, Moffitt R (1991) Dietary effects of the Food Stamp Program. American Journal of Agricultural Economics 73: 202-211.

**DeWalt 1993**DeWalt KM (1993) Nutrition and the commercialization of agriculture: Ten years later. Social Science & Medicine 36: 1407-1416.

**Dhar 2003**Dhar T, Chavas J-P, Gould BW (2003) An empirical assessment of endogeneity issues in demand analysis for differentiated products. American Journal of Agricultural Economics 85: 605-617.

**Dharmasena 2009**Dharmasena S, Capps O (2009) Demand interrelationships of at-home nonalcoholic beverage consumption in the United States. Agricultural & Applied Economics Association’s 2009 AAEA & ACCI Joint Annual Meeting. Milwaukee, Wisconsin: Texas A&M University.

**Dhehibi 2003**Dhehibi B, Gil JM (2003) Forecasting food demand in Tunisia under Alternative Pricing Policies. Food Policy 28: 167-186.

**Dien 2004**Dien LN, Thang NM, Bentley ME (2004) Food consumption patterns in the economic transition in Vietnam. Asia Pacific Journal of Clinical Nutrition 13: 40-47.

**Diez-Roux 2000**Diez-Roux AV, Link BG, Northridge ME (2000) A multilevel analysis of income inequality and cardiovascular disease risk factors. Social Science and Medicine 50: 673-687.

**Diez-Roux 1999**Diez-Roux AV, Northridge ME, Morabia A, Bassett MT, Shea S (1999) Prevalence and social correlates of cardiovascular disease risk factors in Harlem. American Journal of Public Health 89: 302-307.

**Dijkshoorn 2008**Dijkshoorn H, Nierkens V, Nicolaou M (2008) Risk groups for overweight and obesity among Turkish and Moroccan migrants in the Netherlands. Public Health 122: 625-630.

**Dolar 2010**Dolar V (2010) Assessing the effect of changes in relative food prices and icome on obesity prevalence in the United States [Doctoral dissertation]. Minneapolis, MN, United States: University of Minnesota. 72 p.

**Domingues 2007**Domingues MR, Barros AJD (2007) Leisure-time physical activity during pregnancy in the 2004 Pelotas Birth Cohort Study. Revista de Saude Publica 41: 173-180.

**Dong 2009**Dong D, Lin B-H (2009) Fruit and vegetable consumption by low-income Americans: Would a price reduction make a difference? Washington, DC: U.S. Department of Agriculture, Economic Research Service.

**Donkin 1998**Donkin AJM, Johnson AE, Lilley JM, Morgan K, Neale RJ, et al. (1998) Gender and living alone as determinants of fruit and vegetable consumption among the elderly living at home in urban Nottingham. Appetite 30: 39-51.

**Dowda 2001**Dowda M, Ainsworth BE, Addy CL, Saunders R, Riner W (2001) Environmental influences, physical activity, and weight status in 8- to 16-year-olds. Archives of Pediatrics and Adolescent Medicine 155: 711-717.

**Dowler 2001**Dowler E (2001) Inequalities in diet and physical activity in Europe. Public Health Nutrition 4: 701-709.

**Dregval 2009**Dregval L, Petrauskiene A (2009) Associations between physical activity of primary school first-graders during leisure time and family socioeconomic status. Medicina (Kaunas, Lithuania) 45: 549-556.

**Drewnowski 2009**Drewnowski A (2009) Obesity, diets, and social inequalities. Nutrition Reviews 67: s36-s39.

**Drewnowski 2005a**
Drewnowski A, Darmon N (2005a) The economics of obesity: Dietary energy density and energy cost. The American Journal of Clinical Nutrition 82: s265-s273.

**Drewnowski 2005b**
Drewnowski A, Darmon N (2005b) Food choices and diet costs: An economic analysis. The Journal of Nutrition 135: 900-904.

**Drewnowski 2004a**
Drewnowski A (2004a) Obesity and the food environment: Dietary energy density and diet costs. American Journal of Preventive Medicine 27: 154-162.

**Drewnowski 2004b**
Drewnowski A, Specter SE (2004b) Poverty and obesity: The role of energy density and energy costs. American Journal of Clinical Nutrition 79: 6-16.

**Drewnowski 2003**Drewnowski A (2003) Fat and sugar: An economic analysis. Journal of Nutrition 133: s838-s840.

**Drewnowski 1997**Drewnowski A, Popkin BM (1997) The nutrition transition: New trends in the global diet. Nutrition Reviews 55: 31-43.

**Du 2004**Du S, Mroz TA, Zhai F, Popkin BM (2004) Rapid income growth adversely affects diet quality in China--particularly for the poor! Social Science & Medicine 59: 1505-1515.

**Dubois 2007**Dubois L, Farmer A, Girard M, Peterson K, Tatone-Tokuda F (2007) Problem eating behaviors related to social factors and body weight in preschool children: A longitudinal study. International Journal of Behavioral Nutrition and Physical Activity 4.

**Dubois 2006**Dubois L, Girard M, Potvin K, Monique (2006) Breakfast eating and overweight in a pre-school population: Is there a link? Public Health Nutrition 9: 436-442.

**Dubois 2001**Dubois L, Girard M (2001) Social position and nutrition: A gradient relationship in Canada and the USA. European Journal of Clinical Nutrition 55: 366-373.

**Due 2009**Due P, Damsgaard MT, Rasmussen M, Holstein BE, Wardle J, et al. (2009) Socioeconomic position, macroeconomic environment and overweight among adolescents in 35 countries. International Journal of Obesity 33: 1084-1093.

**Duffey 2010**Duffey KJ, Gordon-Larsen P, Shikany JM, Guilkey D, Jacobs DR, Jr, et al. (2010) Food price and diet and health outcomes: 20 years of the CARDIA study. Archive of Internal Medicine 170: 420-426.

**Dumith 2007**Dumith SC, Gigante DP, Domingues MR (2007) Stages of change for physical activity in adults from Southern Brazil: A population-based survey. International Journal of Behavioral Nutrition and Physical Activity 4: 25.

**Dunton 2010**Dunton GF, Berrigan D, Ballard-Barbash R, Perna FM, Graubard BI, et al. (2010) Adolescents' sports and exercise environments in a U.S. time use survey. American Journal of Preventive Medicine 39: 122-129.

**Durham 2010**Durham C, Eales J (2010) Demand elasticities for fresh fruit at the retail level. Applied Economics 42: 1345-1354.

**Ecob 1999**Ecob R, Davey S (1999) Income and health: What is the nature of the relationship? Social Science and Medicine 48: 693-705.

**Edmonds 2001**Edmonds J, Baranowski T, Baranowski J, Cullen KW, Myres D (2001) Ecological and socioeconomic correlates of fruit, juice, and vegetable consumption among African-American boys. Preventive Medicine 32: 476-481.

**El 2011**El R, Nejjari C, Zidouh A, Bakkali R, Berraho M, et al. (2011) Prevalence of obesity and associated sociodemographic and lifestyle factors in Morocco. Public Health Nutrition 14: 160-167.

**Emmons 1987**Emmons L (1987) Relationship of participation in food assistance programs to the nutritional quality of diets. American Journal of Public Health 77: 856-858.

**Ene-Obong 2001**Ene-Obong HN, Enugu GI, Uwaegbute AC (2001) Determinants of health and nutritional status of rural Nigerian women. Journal of Health Population and Nutrition 19: 320-330.

**Engle 1993**
Engle PL (1993) Influences of mothers' and fathers' income on children's nutritional status in Guatemala. Social Science and Medicine 37: 1303-1312.

**Epstein 2010**Epstein LH, Dearing KK, Roba LG, Finkelstein E (2010) The influence of taxes and subsidies on energy purchased in an experimental purchasing study. Psychological Science 21: 406-414.

**Epstein 2007**Epstein LH, Dearing KK, Paluch RA, Roemmich JN, Cho D (2007) Price and maternal obesity influence purchasing of low- and high-energy-dense foods. The American Journal of Clinical Nutrition 86: 914-922.

**Epstein 2006a**
Epstein LH, Handley EA, Dearing KK, Cho DD, Roemmich JN, et al. (2006a) Purchases of food in youth. Influence of price and income. Psychological Science 17: 82-89.

**Epstein 2006b**
Epstein LH, Dearing KK, Handley EA, Roemmich JN, Paluch RA (2006b) Relationship of mother and child food purchases as a function of price: A pilot study. Appetite 47: 115-118.

**Erem 2004**Erem C, Arslan C, Hacihasanoglu A, Deger O, Topbas M, et al. (2004) Prevalence of obesity and associated risk factors in a Turkish population (Trabzon City, Turkey). Obesity Research 12: 1117-1127.

**Eshah 2011**Eshah NF (2011) Lifestyle and health promoting behaviours in Jordanian subjects without prior history of coronary heart disease. International Journal of Nursing Practice 17: 27-35.

**Evers 1987**Evers S (1987) Economic and social factors associated with obesity in adult Canadians. Nutrition Research 7: 3-13.

**Ezzati 2005**Ezzati M, Hoorn SV, Lawes CMM, Leach R, James WPT, et al. (2005) Rethinking the "diseases of affluence" paradigm: Global patterns of nutritional risks in relation to economic development. PLoS Medicine 2: e133.

**Fabiosa 2008**Fabiosa JF (2008) The food-away-from-home consumption expenditure pattern in Egypt. Ames, IA: Iowa State University Center for Agricultural and Rural Development.

**Faeh 2011**Faeh D, Braun J, Bopp M (2011) Prevalence of obesity in Switzerland 1992-2007: The impact of education, income and occupational class. Obesity Reviews 12: 151-166.

**Faith 2007**Faith MS, Fontaine KR, Baskin ML, Allison DB (2007) Toward the reduction of population obesity: Macrolevel environmental approaches to the problems of food, eating, and obesity. Psychological Bulletin 133: 205-226.

**Fan 2010**Fan M (2010) Do food stamps contribute to obesity in low-income women? Evidence from the National Longitudinal Survey of Youth 1979. American Journal of Agricultural Economics 92: 1165-1180.

**Farrell 2002**Farrell L, Shields MA (2002) Investigating the economic and demographic determinants of sporting participation in England. Journal of the Royal Statistical Society: Series A (Statistics in Society) 165: 335-348.

**Faulkner 2010**Faulkner G, Grootendorst P, Nguyen VH, Ferrence R, Mendelson R, et al. (2010) Economic policy, obesity and health: A scoping review. Ottawa, ON: Heart and Stroke Foundation of Canada. [*Primary reference*]

Faulkner G, Grootendorst P, Nguyen V, Andreyeva T, Arbour-Nicitopoulos K, et al. (2011) Economic instruments for obesity prevention: Results of a scoping review and modified delphi survey. International Journal of Behavioral Nutrition and Physical Activity 8: 109.

**Ferguson 2010**Ferguson TS, Younger N, Tulloch-Reid MK, Forrester TE, Cooper RS, et al. (2010) Prevalence of the metabolic syndrome in Jamaican adults and its relationship to income and education levels. The West Indian Medical Journal 59: 265-273.

**Fernald 2007**Fernald LCH (2007) Socio-economic status and body mass index in low-income Mexican adults. Social Science & Medicine 64: 2030-2042.

**Ferrar 2010**Ferrar K, Olds T (2010) Thin adolescents: Who are they? What do they do? Socio-demographic and use-of-time characteristics. Preventive Medicine: An International Journal Devoted to Practice and Theory 51: 253-258.

**Ferreira 2006**Ferreira I, van der Horst K, Wendel-Vos W, Kremers S, van Lenthe FJ, et al. (2006) Environmental correlates of physical activity in youth - A review and update. Obesity Reviews 8: 129-154.

**Fey-Yensan 2003**Fey-Yensan N, English C, Pacheco HE, Belyea M, Schuler D (2003) Elderly food stamp participants are different from eligible nonparticipants by level of nutrition risk but not nutrient intake. Journal of the American Dietetic Association 103: 103-107.

**Figueiredo 2007**Figueiredo FP, Silva AAM, Bettiol H, Barbieri MA, Batista RFL, et al. (2007) Early life, current socioeconomic position and serum lipids in young adulthood of participants in a cohort study initiated in 1978/1979. Brazilian Journal of Medical and Biological Research 40: 1267-1276.

**Finke 2003**Finke MS, Huston SJ (2003) Factors affecting the probability of choosing a risky diet. Journal of Family and Economic Issues 24: 291-303.

**Finkelstein 2010**Finkelstein EA, Zhen C, Nonnemaker J, Todd JE (2010) Impact of targeted beverage taxes on higher- and lower-income households. Archives of Internal Medicine 170: 2028-2034.

**Fischer 1999**Fischer ID, Brown DR, Blanton CJ, Casper ML, Croft JB, et al. (1999) Physical activity patterns of Chippewa and Menominee Indians: The Inter-Tribal Heart Project. American Journal of Preventive Medicine 17: 189-197.

**Flegal 1988**Flegal KM, Harlan WR, Landis JR (1988) Secular trends in body mass index and skinfold thickness with socioeconomic factors in young adult men. American Journal of Clinical Nutrition 48: 544-551.

**Fleischer 2008**Fleischer NL, Diez R, Ana V, Alazraqui M, Spinelli H (2008) Social patterning of chronic disease risk factors in a Latin American city. Journal of Urban Health 85: 923-937.

**Fleming 1994**
Fleming KH, Heimbach JT (1994) Consumption of calcium in the U.S.: Food sources and intake levels. Journal of Nutrition 124: s1426-s1430.

**Fletcher** **2010a**
Fletcher JM, Frisvold D, Tefft N (2010a) Can soft drink taxes reduce population weight? Contemporary Economic Policy 28: 23-35. [*Primary reference*]

Fletcher JM, Frisvold DE, Tefft N (2010b) The effects of soft drink taxes on child and adolescent consumption and weight outcomes. Journal of Public Economics 94: 967-974.

Fletcher J, Frisvold D, Tefft N (2009) The effects of soft drink taxes on child and adolescent consumption and weight outcomes. Atlanta, GA: Emory University.

**Floyd 2008**Floyd MF, Spengler JO, Maddock JE, Gobster PH, Suau L (2008) Environmental and social correlates of physical activity in neighborhood parks: An observational study in Tampa and Chicago. Leisure Sciences 30: 360-375.

**Fox 2004**Fox MK, Cole N (2004a) Nutrition and health characteristics of low-income populations: Volume I, Food Stamp Program participants and nonparticipants. Alexandria, VA: US Department of Agriculture, Economic Research Service. [*Primary reference*]

Fox MK, Hamilton W, Lin B-H (2004b) Effects of food assistance and nutrition programs on nutrition and health: Volume 4, executive summary of the literature review. Alexandria, VA: US Department of Agriculture, Economic Research Service.

**Frank 2008**Frank L, Bradley M, Kavage S, Chapman J, Lawton T (2008) Urban form, travel time, and cost relationships with tour complexity and mode choice. Transportation 35: 37-54.

**Frank 2007**Frank LD, Saelens BE, Powell KE, Chapman JE (2007) Stepping towards causation: Do built environments or neighborhood and travel preferences explain physical activity, driving, and obesity? Social Science & Medicine 65: 1898-1914.

**Frank 2004**Frank LD, Andresen MA, Schmid TL (2004) Obesity relationships with community design, physical activity, and time spent in cars. American Journal of Preventive Medicine 27: 87-96.

**Freedman 2007**Freedman DS, Ogden CL, Flegal KM, Khan LK, Serdula MK, et al. (2007) Childhood overweight and family income. MedGenMed: Medscape General Medicine 9: 26.

**French 2010**French SA, Hannan PJ, Harnack LJ, Mitchell NR, Toomey T, L., et al. (2010) Pricing and availability intervention in vending machines at four bus garages. Journal of Occupational & Environmental Medicine 52 s29-s33.

**French 2004b**
French SA, Wechsler H (2004b) School-based research and initiatives: Fruit and vegetable environment, policy, and pricing workshop. Preventive Medicine: An International Journal Devoted to Practice and Theory 39: s101-s107.

**French 2001a**
French SA, Jeffery RW, Story M, Breitlow KK, Baxter JS, et al. (2001a) Pricing and promotion effects on low-fat vending snack purchases: The CHIPS study. American Journal of Public Health 91: 112-117.

**French 2001b**
French SA, Story M, Jeffery RW (2001b) Environmental influences on eating and physical activity. Annual Review of Public Health 22: 309-335.

**French 2000**French SA, Harnack L, Jeffery RW (2000) Fast food restaurant use among women in the Pound of Prevention study: Dietary, behavioral and demographic correlates. International Journal of Obesity & Related Metabolic Disorders 24: 1353-1359.

**French 1997a**
French SA, Jeffery RW, Story M, Hannan P, Snyder MP (1997a) A pricing strategy to promote low-fat snack choices through vending machines. American Journal of Public Health 87: 849-851. [*Primary reference*]

French SA (2005) Public health strategies for dietary change: Schools and workplaces. The Journal of Nutrition 135: 910-912.

French SA (2003) Pricing effects on food choices. Journal of Nutrition 133: s841-s843.

**French 1997b**
French SA, Story M, Jeffery RW, Snyder P, Eisenberg M, et al. (1997) Pricing strategy to promote fruit and vegetable purchase in high school cafeterias. Journal of the American Dietetic Association 97: 1008-1010.

**Fulwood 1980**Fulwood R (1980) Serum cholesterol levels of persons 4-74 years of age by socioeconomic characteristics, United States 1971-74. Vital and Health Statistics. Hyattsville, MD: National Health Survey, U.S. Department of Health, Education, and Welfare. [*Primary reference*]

Fulwood R (1981) Height and weight of adults, ages 18-74 years, by socioeconomic and geographic variables, United States, 1971-74. Hyattsville, MD: U.S. Dept. of Health and Human Services, Public Health Service Office of Health Research Statistics and Technology, National Center for Health Statistics.

**Gabe 2008**Gabe T (2008) Fiscal and economic impacts of beverage excise taxes imposed by Maine Public Law 629. Orono, ME: University of Maine, School of Economics.

**Garcia Villar 2009**Garcia Villar J, Quintana-Domeque C (2009) Income and body mass index in Europe. Economics and Human Biology 7: 73-83. [*Primary reference*]

Garcia Villar J, Quintana-Domeque C (2006) Income and body mass index in Europe. Barcelona: Universitat Pompeu Fabra.

**Garn 1977**Garn SM, Bailey SM, Cole PE, Higgins IT (1977) Level of education, level of income, and level of fatness in adults. American Journal of Clinical Nutrition 30: 721-725.

**Gary 2004**Gary TL, Baptiste-Roberts K, Gregg EW, Williams DE, Beckles GL, et al. (2004) Fruit, vegetable and fat intake in a population-based sample of African Americans. Journal of the National Medical Association 96: 1599-1605.

**Gaston 2011**Gaston MH, Porter GK, Thomas VG (2011) Paradoxes in obesity with mid-life African American women. Journal of the National Medical Association 103: 17-25.

**Gelbach 2009**Gelbach JB, Klick J, Stratmann T (2009) Cheap donuts and expensive broccoli: The effect of relative prices on obesity. Social Science Research Network eLibrary.

**Giardina 2009**Giardina E-GV, Laudano M, Hurstak E, Saroff A, Fleck E, et al. (2009) Physical activity participation among Caribbean Hispanic women living in New York: Relation to education, income, and age. Journal of Women's Health 18: 187-193.

**Gibson 2006**Gibson D (2006) Long-term Food Stamp Program participation is positively related to simultaneous overweight in young daughters and obesity in mothers. Journal of Nutrition 136: 1081-1085.

**Gibson 2004**Gibson D (2004) Long-term Food Stamp Program participation is differentially related to overweight in young girls and boys. Journal of Nutrition 134: 372-379.

**Gibson 2003**Gibson D (2003) Food Stamp Program participation is positively related to obesity in low income women. Journal of Nutrition 133: 2225-2231.

**Gigante 2008**Gigante DP, Minten GC, Horta BL, Barros FC, Victora CG (2008) Evaluación nutricional de adultos de la cohorte de nacimientos de 1982, Pelotas, Sur de Brasil [Nutritional evaluation follow-up of the 1982 birth cohort, Pelotas, Southern Brazil]. Revista de Saude Publica 42 60-69.

**Giles-Corti 2002**Giles-Corti B, Donovan RJ (2002) The relative influence of individual, social and physical environment determinants of physical activity. Social Science & Medicine 54: 1793-1812.

**Giskes 2011**Giskes K, van Lenthe FJ, Avendano-Pabon M, Brug J (2011) A systematic review of environmental factors and obesogenic dietary intakes among adults: Are we getting closer to understanding obesogenic environments? Obesity Reviews 12: e95-e106.

**Giskes 2010**Giskes K, Avendano M, Brug J, Kunst AE (2010) A systematic review of studies on socioeconomic inequalities in dietary intakes associated with weight gain and overweight/obesity conducted among European adults. Obesity Reviews 11: 413-429.

**Giskes 2007**Giskes K, van Lenthe FJ, Brug J, Mackenbach JP, Turrell G (2007) Socioeconomic inequalities in food purchasing: The contribution of respondent-perceived and actual (objectively measured) price and availability of foods. Preventive Medicine 45: 41-48.

**Giskes 2006**Giskes K, Turrell G, van Lenthe FJ, Brug J, Mackenbach JP (2006) A multilevel study of socio-economic inequalities in food choice behaviour and dietary intake among the Dutch population: The GLOBE study. Public Health Nutrition 9: 75-83.

**Giskes 2002**Giskes K, Turrell G, Patterson C, Newman B (2002) Socio-economic differences in fruit and vegetable consumption among Australian adolescents and adults. Public Health Nutrition 5: 663-669.

**Glanz 2004a**
Glanz K, Yaroch AL (2004a) Strategies for increasing fruit and vegetable intake in grocery stores and communities: Policy, pricing, and environmental change. Preventive Medicine 39: 75-80.

**Glanz 2004b**
Glanz K, Hoelscher D (2004b) Increasing fruit and vegetable intake by changing environments, policy and pricing: Restaurant-based research, strategies, and recommendations. Preventive Medicine 39: 88-93.

**Glanz 1998**Glanz K, Basil M, Maibach E, Goldberg J, Snyder DAN (1998) Why Americans eat what they do: Taste, nutrition, cost, convenience, and weight control concerns as influences on food consumption. Journal of the American Dietetic Association 98: 1118-1126.

**Glanz 1995**Glanz K, Lankenau B, Foerster S, Temple S, Mullis R, et al. (1995) Environmental and policy approaches to cardiovascular disease prevention through nutrition: Opportunities for state and local action. Health Education & Behavior 22: 512-527.

**Gleason 2009a**
Gleason PM, Dodd AH (2009a) School breakfast program but not school lunch program participation is associated with lower body mass index. Journal of the American Dietetic Association 109: s118-s128.

Gleason PM (1995) Participation in the National School Lunch Program and the School Breakfast Program. American Journal of Clinical Nutrition 61: s213-s220.

**Gleason 2009b**
Gleason PM, Briefel RR, Wilson A, Dodd AH (2009b) School meal program participation and its association with dietary patterns and childhood obesity. Princeton, NJ: Mathematica Policy Research Inc.

**Gleason 2003**Gleason PM, Suitor CW (2003) Eating at school: How the National School Lunch Program affects children's diets. American Journal of Agricultural Economics 85: 1047-1061.

**Gleason 2000**Gleason PM, Rangarajan A, Olson C (2000) Dietary intake and dietary attitudes among food stamp participants and other low-income individuals. Alexandria, VA: US Department of Agriculture, Food and Nutrition Service.

**Godley 2010**Godley J, McLaren L (2010) Socioeconomic status and body mass index in Canada: Exploring measures and mechanisms. Canadian Review of Sociology 47: 381-403.

**Godoy 2005**Godoy R, Byron E, Reyes-Garcia V, Vadez V, Leonard WR, et al. (2005) Income inequality and adult nutritional status: Anthropometric evidence from a pre-industrial society in the Bolivian Amazon. Social Science & Medicine 61: 907-919.

**Goldman 2009**Goldman D, Lakdawalla D, Zheng Y (2009) Food prices and the dynamics of body weight. Cambridge, MA: National Bureau of Economic Research.

**Gonzalez 2009**Gonzalez DA, Nazmi A, Yudkin JS, Victora CG (2009) Life-course socio-economic factors, skin colour and abdominal obesity in adulthood in a Brazilian birth cohort. Public Health Nutrition 12: 2225-2235.

**Goodman 2006**Goodman C, Anise A (2006) What is known about the effectiveness of economic instruments to reduce consumption of foods high in saturated fats and other energy-dense foods for preventing and treating obesity? Copenhagen: WHO Regional Office for Europe.

**Goodman 2003**Goodman E, Adler NE, Daniels SR, Morrison JA, Slap GB, et al. (2003) Impact of objective and subjective social status on obesity in a biracial cohort of adolescents. Obesity Research 11: 1018-1026.

**Goodman 1999**Goodman E (1999) The role of socioeconomic status gradients in explaining differences in US adolescents' health. American Journal of Public Health 89: 1522-1528.

**Gordon 2010**Gordon RA, Kaestner R, Korenman S, Abner K (2010) The Child and Adult Care Food Program: Who is served and what are their nutritional outcomes? Cambridge, MA: National Bureau of Economic Reserach.

**Gordon 1995**Gordon AR, McKinney P (1995) Sources of nutrients in students' diets. American Journal of Clinical Nutrition 61: s232-s240.

**Gordon-Larsen 2003**Gordon-Larsen P, Adair LS, Popkin BM (2003) The relationship of ethnicity, socioeconomic factors, and overweight in US adolescents. Obesity Research 11: 121-129.

**Gordon-Larsen 2000**Gordon-Larsen P, McMurray RG, Popkin BM (2000) Determinants of adolescent physical activity and inactivity patterns. Pediatrics 105: E83.

**Gorin 2007**Gorin AA, Raynor HA, Niemeier HM, Wing RR (2007) Home grocery delivery improves the household food environments of behavioral weight loss participants: Results of an 8-week pilot study. International Journal of Behavioral Nutrition and Physical Activity 4: 1-6.

**Grabnerg 2009**Grabnerg MJ (2009) The effect of socio-economic status on obesity and related health outcomes [Doctoral dissertation]. Davis, CA: University of California, Davis.

**Gray 2007**Gray VB, Byrd SH, Cossman JS, Chromiak J, Cheek WK, et al. (2007) Family characteristics have limited ability to predict weight status of young children. Journal of the American Dietetic Association 107: 1204-1209.

**Gregg 2007**Gregg P, Propper C, Washbrook E (2007) Understanding the relationship between parental income and multiple child outcomes: A decomposition analysis. London, UK: Centre for Analysis of Social Exclusion, London School of Economics.

**Grievink 2004**Grievink L, Alberts JF, O'Niel J, Gerstenbluth I (2004) Waist circumference as a measurement of obesity in the Netherlands Antilles; Associations with hypertension and diabetes mellitus. European Journal of Clinical Nutrition 58: 1159-1165.

**Griffith 2009a**
Griffith R, Nesheim R, O'Connell M (2009a) Empirical estimates of the impact of a fat tax. London, UK: Institute for Fiscal Studies, University College London.

**Groth 2001**Groth MV, Fagt S, Brondsted L (2001) Social determinants of dietary habits in Denmark. European Journal of Clinical Nutrition 55: 959-966.

**Grujic 2009**Grujic V, Cvejin MM, Nikolic EA, Dragnic N, Jovanovic VM, et al. (2009) Udruženost gojaznosti sa socioekonomskim faktorima i stilovima života [Association between obesity and socioeconomic factors and lifestyle]. Vojnosanitetski Pregled 66: 705-710.

**Guevremont 2008**Guevremont A, Findlay L, Kohen D (2008) Organized extracurricular activities of Canadian children and youth. Health reports / Statistics Canada, Canadian Centre for Health Information - Rapports sur la sante / Statistique Canada, Centre canadien d'information sur la sante 19: 65-69.

**Gulliford 2004**Gulliford MC, Mahabir D, Rocke B (2004) Socioeconomic inequality in blood pressure and its determinants: Cross-sectional data from Trinidad and Tobago. Journal of Human Hypertension 18: 61-70.

**Gundersen 2008**Gundersen C, Lohman BJ, Eisenmann JC, Garasky S, Stewart SD (2008) Child-specific food insecurity and overweight are not associated in a sample of 10- to 15-year-old low-income youth. Journal of Nutrition 138: 371-378.

**Guo 2009**Guo X, Willows N, Kuhle S, Jhangri G, Veugelers PJ (2009) Use of vitamin and mineral supplements among Canadian adults. Canadian Journal of Public Health 100: 357-360.

**Guo 2004**Guo X, Warden BA, Paeratakul S, Bray GA (2004) Healthy eating index and obesity. European Journal of Clinical Nutrition 58: 1580-1586.

**Guo 1999**Guo X, Popkin BM, Mroz TA, Zhai F (1999) Food price policy can favorably alter macronutrient intake in China. The Journal of Nutrition 129: 994-1001.

**Gustavsen 2011**Gustavsen GW, Rickertsen K (2011) The effects of taxes on purchases of sugar-sweetened carbonated soft drinks: A quantile regression approach. Applied Economics 43: 707-716. [*Primary reference*]

Gustavsen GW, Rickertsen K (2010) Effects of taxes and subsidies on food purchases: A quantile regression approach. Oslo, Norway: Norwegian Agricultural Economics Research Institute.

**Gustavsen 2005**Gustavsen GW (2005) Public policies and the demand for carbonated soft drinks: A censored quantile regression approach. XIth Congress of the European Association of Agricultural Economists, 'The Future of Rural Europe in the Global Agri-Food System'. Copenhagen, Denmark.

**Gustavsen 2004**Gustavsen GW, Rickertsen K (2004) For whom reduced prices count: A censored quantile regression analysis of vegetable demand. American Agricultural Economics Association Annual Meeting. Denver, CO. [*Primary reference*]

Gustavsen GW, Rickertsen K (2006) A censored quantile regression analysis of vegetable demand: The effects of changes in prices and total expenditure. Canadian Journal of Agricultural Economics 54: 631-645.

**Guthrie 2007**Guthrie JF, Lin B-H, Ver Ploeg M, Frazao E (2007) Can food stamps do more to improve food choices? Alexandria, VA: US Department of Agriculture, Economic Research Service.

**Guthrie 2002**Guthrie JF, Lin B-H (2002) Overview of the diets of lower- and higher-income elderly and their food assistance options. Journal of Nutrition Education & Behavior 34: s31-s41.

**Gutierrez 2010**Gutierrez OM, Anderson C, Isakova T, Scialla J, Negrea L, et al. (2010) Low socioeconomic status associates with higher serum phosphate irrespective of race. Journal of the American Society of Nephrology 21: 1953-1960.

**Hakeem 2001**Hakeem R (2001) Socio-economic differences in height and body mass index of children and adults living in urban areas of Karachi, Pakistan. European Journal of Clinical Nutrition 55: 400-406.

**Hall 2009**Hall JN, Moore S, Harper SB, Lynch JW (2009) Global variability in fruit and vegetable consumption. American Journal of Preventive Medicine 36: 402-409.

**Hallal 2006**Hallal PC, Wells JCK, Reichert FF, Anselmi L, Victora CG (2006) Early determinants of physical activity in adolescence: Prospective birth cohort study. British Medical Journal 332: 1002-1005.

**Hallal 2005**Hallal PC, Azevedo MR, Reichert FF, Siqueira FV, Araujo CLP, et al. (2005) Who, when, and how much? Epidemiology of walking in a middle-income country. American Journal of Preventive Medicine 28: 156-161.

**Hamermesh 2009**Hamermesh DS (2009) Grazing, goods and girth: Determinants and effects. Cambridge, MA: National Bureau of Economic Research. [*Primary reference*]

Hamermesh DS (2010) Incentives, time use and BMI: The roles of eating, grazing and goods. Economics and Human Biology 8: 2-15.

**Hamermesh 2006**Hamermesh DS (2006) Time to eat: Household production under increasing income inequality. Cambridge, MA: National Bureau of Economic Research. [*Primary reference*]

Hamermesh DS (2007) Time to eat: Household production under increasing income inequality. American Journal of Agricultural Economics 89: 852-863.

**Hamilton 1985**Hamilton N, Zimmerman R (1985) Weight control: The interaction of marital power and weight loss success. Journal of Social Service Research 8: 51-64.

**Han 2011**Han E, Powell LM (2011) Effect of food prices on the prevalence of obesity among young adults. Public Health 125: 129-135.

**Hann 2001**Hann CS, Rock CL, King I, Drewnowski A (2001) Validation of the Healthy Eating Index with use of plasma biomarkers in a clinical sample of women. American Journal of Clinical Nutrition 74: 479-486.

**Harnack 2008**Harnack LJ, French SA, Michael JM, Story MT, Jeffery RW, et al. (2008) Effects of calorie labeling and value size pricing on fast food meal choices: Results from an experimental trial. International Journal of Behavioral Nutrition and Physical Activity 5.

**Harrell 1998**Harrell JS, Gore SV (1998) Cardiovascular risk factors and socioeconomic status in African American and Caucasian women. Research in Nursing & Health 21: 285-295.

**Harro 2006**Harro M, Oja L, Tekkel M, Aru J, Villa I, et al. (2006) Monitoring physical activity in Baltic countries: The FINBALT study, HBSC and other surveys in young people. Journal of Public Health 14: 103-109.

**Hasab 1999**Hasab AA, Jaffer A, Hallaj Z (1999) Blood pressure patterns among the Omani population. Eastern Mediterranean Health Journal 5: 46-54.

**Haste 1990**Haste FM, Brooke OG, Anderson HR, Bland JM, Peacock JL (1990) Social determinants of nutrient intake in smokers and non-smokers during pregnancy. Journal of Epidemiology and Community Health 44: 205-209.

**Hawkes 2009a**
Hawkes C (2009a) Financial incentives and disincentives to encourage healthy eating. London: Which? Ltd.

**Hawkes 2009b**
Hawkes C (2009b) Sales promotions and food consumption. Nutrition Reviews 67: 333-342.

**Hay 1998**Hay DI (1988) Socioeconomic status and health status: A study of males in the Canada Health Survey. Social Science and Medicine 27: 1317-1325.

**He 2004**He XZ, Baker DW (2004) Changes in weight among a nationally representative cohort of adults aged 51 to 61, 1992 to 2000. American Journal of Preventive Medicine 27: 8-15.

**Heien 1998**Heien DM, Wessells CR (1988) The demand for dairy products: Structure, prediction, and decomposition. American Journal of Agricultural Economics 70: 219-228.

**Helmert 1997**Helmert U, Mielck A, Shea S (1997) Poverty and health in West Germany. Sozial- und Praventivmedizin 42: 276-285.

**Helmert 1990**Helmert U, Shea S, Herman B, Greiser E (1990) Relationship of social class characteristics and risk factors for coronary heart disease in West Germany. Public Health 104: 399-416.

**Herbst 2009**Herbst CM, Tekin E (2009) Child care subsidies and childhood obesity. Bonn, Germany: Forschungsinstitut zur Zukunft der Arbeit (IZA) Institute for the Study of Labor.

**Herman 2008**Herman DR, Harrison GG, Afifi AA, Jenks E (2008) Effect of a targeted subsidy on intake of fruits and vegetables among low-income women in the special supplemental nutrition program for women, infants, and children. American Journal of Public Health 98: 98-105.

**Hernandez 2011**Hernandez DC, Francis LA, Doyle EA (2011) National School Lunch Program participation and sex differences in body mass index trajectories of children from low-income families. Archives of Pediatrics & Adolescent Medicine 165: 346-353.

**Hjartaker 2001**Hjartaker A, Laake P, Lund E (2001) Body mass index and weight change attempts among adult women. The Norwegian Women and Cancer Study. European Journal of Public Health 11: 141-146.

**Ho 1998**Ho TH, Tang C, Bell D (1998) Rational shopping behavior and the option value of variable pricing. Management Science 44: s145-s160.

**Hoddinott 2003**Hoddinott J, Skoufias E (2003) The impact of PROGRESA on food consumption Washington, D.C.: International Food Policy Reserach Institute(IFPRI). 0379-5721 0379-5721.

**Hoderlein 2008**Hoderlein S, Mihaleva S (2008) Increasing the price variation in a repeated cross section. Journal of Econometrics 147: 316-325.

**Hodgkins 1990**Hodgkins BJ, Manring E, Meyers MA (1990) Demographic, social and stress correlates of hypertension among the urban poor. Family Practice 7: 261-266.

**Hodgson 2011**Hodgson C (2011) Obesity in Canada: A joint report from the Public Health Agency of Canada and the Canadian Institute for Health Information. Ottawa, ON: Public Health Agency of Canada. 1499-2671 1499-2671.

**Hofferth 2005**Hofferth SL, Curtin S (2005) Poverty, food programs, and childhood obesity. Journal of Policy Analysis and Management 24: 703-726.

**Holsten 2008**Holsten JE (2008) Obesity and the community food environment: A systematic review. Public Health Nutrition 12: 397-405.

**Horgen 2002**Horgen KB, Brownell KD (2002) Comparison of price change and health message interventions in promoting healthy food choices. Health Psychology 21: 505-512.

**Horta 2008a**
Horta BL, Gigante DP, Victora CG, Barros FC (2008a) Early determinants of blood pressure among adults of the 1982 birth cohort, Pelotas, Southern Brazil. Revista de Saude Publica 42: 86-92.

**Hou 2011**Hou N, Popkin BM, Jacobs JDR, Song Y, Guilkey DK, et al. (2011) Longitudinal trends in gasoline price and physical activity: The CARDIA study. Preventive Medicine 52: 365-369.

**Hoynes 2007**Hoynes H, Schanzenbach D (2007) Consumption reponses to in-kind transfers: Evidence from the introduction of the Food Stamp Program. Cambridge, MA: National Bureau of Economic Research.

**Hu 2009**Hu Y, Block G, Sternfeld B, Sowers M (2009) Dietary glycemic load, glycemic index, and associated factors in a multiethnic cohort of midlife women. Journal of the American College of Nutrition 28: 636-647.

**Hu 2002**Hu G, Pekkarinen H, Hanninen O, Yu Z, Tian H, et al. (2002) Physical activity during leisure and commuting in Tianjin, China. Bulletin of the World Health Organization 80: 933-938.

**Huang 2011**Huang Y, Hannon PA, Williams B, Harris JR (2011) Workers' health risk behaviors by state, demographic characteristics, and health insurance status. Preventing Chronic Disease 8: A12.

**Huang 1988**Huang KS (1988) An inverse demand system for U.S. composite foods. American Journal of Agricultural Economics 70: 902-909.

**Huang 1996**Huang KS (1996) Nutrient elasticities in a complete food demand system. American Journal of Agricultural Economics 78: 21-29. [*Primary reference*]

Huang KS, Lin B-H (2000) Estimation of food demand and nutrient elasticities from household survey data Washington DC: U.S. Department of Agriculture, Economic Research Service, Food and Rural Economics Division.

Huang KS (1999) Effects of food prices and consumer income on nutrient availability. Applied Economics 31: 367-380.

Huang KS (1998) How economic factors influence the nutrient content of diets. Annual Meeting of the American Agricultural Association. Salt Lake City, Utah: US Department of Agriculture, Economic Research Service.

**Huang 1981**Huang CL, Fletcher SM, Raunikar R (1981) Modeling the effects of the Food Stamp Program on participating households' purchases: An empirical application. Southern Journal of Agricultural Economics 13: 21-28.

**Huot 2004**Huot I, Paradis G, Ledoux M (2004) Factors associated with overweight and obesity in Quebec adults. International Journal of Obesity 28: 766-774.

**Inglis 2009**Inglis V, Ball K, Crawford D (2009) Does modifying the household food budget predict changes in the healthfulness of purchasing choices among low- and high-income women? Appetite 52: 273-279.

**Inglis 2008**Inglis V, Ball K, Crawford D (2008) Socioeconomic variations in women's diets: What is the role of perceptions of the local food environment? Journal of Epidemiology & Community Health 62: 191-197.

**Iribarren 1997**Iribarren C, Luepker RV, McGovern PG, Arnett DK, Blackburn H (1997) Twelve-year trends in cardiovascular disease risk factors in the Minnesota Heart Survey. Are socioeconomic differences widening? Archives of Internal Medicine 157: 873-881.

**Ivanova 2008**Ivanova L, Dimitrov P, Dellava J, Hoffman D (2008) Prevalence of obesity and overweight among urban adults in Bulgaria. Public Health Nutrition 11: 1407-1410.

**Ivers 2010**Ivers LC, Chang Y, Gregory J, Freedberg KA (2010) Food assistance is associated with improved body mass index, food security and attendance at clinic in an HIV program in central Haiti: A prospective observational cohort study. AIDS Research and Therapy 7.

**Jahns 2012**Jahns L, Adair L, Mroz T, Popkin BM (2012) The declining prevalence of overweight among Russian children: Income, diet, and physical activity behavior changes. Economics & Human Biology 10: 139-146.

**Jahns 2003**Jahns L, Baturin A, Popkin BM (2003) Obesity, diet, and poverty: Trends in the Russian transition to market economy. European Journal of Clinical Nutrition 57: 1295-1302.

**Jaime 2009**Jaime PC, Lock K (2009) Do school based food and nutrition policies improve diet and reduce obesity? Preventive Medicine 48: 45-53.

**Janicki-Deverts 2009**Janicki-Deverts D, Cohen S, Matthews KA, Gross MD, Jacobs DRJ (2009) Socioeconomic status, antioxidant micronutrients, and correlates of oxidative damage: The Coronary Artery Risk Development in Young Adults (CARDIA) study. Psychosomatic Medicine 71: 541-548.

**Jansen 1977**Jansen GR, Jansen NB, Shigetomi CT, Harper JM (1977) Effect of income and geographic region on the nutritional value of diets in Brazil. American Journal of Clinical Nutrition 30: 955-964.

**Jeffery 1996**Jeffery RW, French SA (1996) Socioeconomic status and weight control practices among 20- to 45-year-old women. American Journal of Public Health 86: 1005-1005.

**Jeffery 1994**Jeffery RW, French SA, Raether C, Baxter JE (1994) An environmental intervention to increase fruit and salad purchases in a cafeteria. Preventive Medicine: An International Journal Devoted to Practice and Theory 23: 788-792.

**Jekanowski 2001**Jekanowski MD, Binkley JK, Eales JS (2001) Convenience, accessibility, and the demand for fast food. Journal of Agricultural and Resource Economics 26: 58-74.

**Jensen 2010**Jensen RT, Miller NH (2010) Do consumer price subsidies really improve nutrition? Cambridge, MA: National Bureau of Economic Research.

**Jensen 2007**Jensen JD, Smed S (2007) Cost-effective design of economic instruments in nutrition policy. International Journal of Behavioral Nutrition and Physical Activity 4: 10.

**Jensen 1998**Jensen HH, Manrique J (1998) Demand for food commodities by income groups in Indonesia. Applied Economics 30: 491-501. [*Primary reference*]

Jensen HH, Manrique J (1996) Demand for food commodities by income groups in Indonesia. Ames, IA: Center for Agricultural and Rural Development, Iowa State University.

**Jenum 2001**Jenum AK, Stensvold I, Thelle DS (2001) Differences in cardiovascular disease mortality and major risk factors between districts in Oslo. An ecological analysis. International Journal of Epidemiology 30: s59-s65.

**Jha 2006**Jha R, Gaiha R, Sharma A (2006) On modelling variety in consumption expenditure on food. Canberra, Australia: Australia South Asia Research Centre, Australian National University. [*Primary reference*]

Jha R, Gaiha R, Sharma A (2009) Modelling variety in consumption expenditure on food in India. International Review of Applied Economics 23: 503-519.

**Jiang 2007a**
Jiang B, Davis J (2007a) Household food demand in rural China. Applied Economics 39: 373-380.

**Jiang 2007b**
Jiang Y, Chen Y, Manuel D, Morrison H, Mao Y (2007b) Quantifying the impact of obesity category on major chronic diseases in Canada. The Scientific World Journal 7: 1211-1221.

**Jilcott 2011b**
Jilcott SB, Liu H, Dubose KD, Chen S, Kranz S (2011b) Food stamp participation is associated with fewer meals away from home, yet higher body mass index and waist circumference in a nationally representative sample. Journal of Nutrition Education & Behavior 43: 110-115.

**Jo 2009**Jo C, Lim J-Y (2009) The effect of the Food Stamp Program and the minimum wage on obesity: An empirical investigation using Behavioral Risk Factor Surveillance System (BRFSS) Data. Journal of Economic Research 14: 17-48.

**Johnson 1994a**Johnson RK, Guthrie H, Smiciklas-Wright H, Min QW (1994a) Characterizing nutrient intakes of children by sociodemographic factors. Public Health Reports 109: 414-420. [*Primary reference*]

Johnson RK, Johnson DG, Min QW, Smiciklas-Wright H, Guthrie HA (1994b) Characterizing nutrient intakes of adolescents by sociodemographic factors. Journal of Adolescent Health 15: 149-154.

**Johnston 2007**Johnston DW, Propper C, Shields MA (2007) Comparing subjective and objective measures of health: Evidence from hypertension for the income/health gradient. Bonn, Germany: Forschungsinstitut zur Zukunft der Arbeit (IZA) Institute for the Study of Labor. [*Primary reference*]

Johnston DW, Propper C, Shields MA (2009) Comparing subjective and objective measures of health: Evidence from hypertension for the income/health gradient. Journal of Health Economics 28: 540-552.

**Joliffe 2007**Joliffe D (2007) The income gradient and distribution-sensitive measures of overweight in the U.S. International Association of Agricultural Economists Conference. Gold Coast, Australia: Economic Research Service, U.S. Department of Agriculture.

**Jones 2006**Jones SJ, Frongillo EA (2006) The modifying effects of Food Stamp Program participation on the relation between food insecurity and weight change in women. Journal of Nutrition 136: 1091-1094.

**Jones 2003a**
Jones E, Akbay C, Roe B, Chern WS (2003a) Analyses of consumers' dietary behavior: An application of the AIDS model to supermarket scanner data. Agribusiness 19: 203-221.

**Jones 2003b**
Jones SJ, Jahns L, Laraia BA, Haughton B (2003b) Lower risk of overweight in school-aged food insecure girls who participate in food assistance: Results from the panel study of income dynamics child development supplement. Archives of Pediatrics & Adolescent Medicine 157: 780-784.

**Jones 1997**Jones E (1997) An analysis of consumer food shopping behavior using supermarket scanner data: Differences by income and location. American Journal of Agricultural Economics 79: 1437-1443.

**Jonnalagadda 2000**Jonnalagadda SS, Earnest SG, Baxter DH, Cody MM, Mullis RM (2000) Dietary intake, socioeconomic status and cardiovascular disease risk in African-American women. Nutrition Research 20: 491-503.

**Jurakic 2009**Jurakic D, Pedisic Z, Andrijasevic M (2009) Physical activity of Croatian population: Cross-sectional study using International Physical Activity Questionnaire. Croatian Medical Journal 50: 165-173.

**Kahn 1998**Kahn HS, Tatham LM, Pamuk ER, Heath CW, Jr. (1998) Are geographic regions with high income inequality associated with risk of abdominal weight gain? Social Science and Medicine 47: 1-6.

**Kahn 1991**Kahn HS, Williamson DF, Stevens JA (1991) Race and weight change in US women: The roles of socioeconomic and marital status. American Journal of Public Health 81: 319-323.

**Kaleta 2007**Kaleta D, Jegier A (2007) Predictors of inactivity in the working-age population. International Journal of Occupational Medicine and Environmental Health 20: 175-182.

**Kamphuis 2009**Kamphuis CBM, van Lenthe FJ, Giskes K, Huisman M, Brug J, et al. (2009) Socioeconomic differences in lack of recreational walking among older adults: The role of neighbourhood and individual factors. International Journal of Behavioral Nutrition and Physical Activity 6.

**Kanao 2009**Kanao BJ, Abu-Nada OS, Zabut BM (2009) Nutritional status correlated with sociodemographic and economic factors among preparatory school-aged children in the Gaza Strip. Journal of Public Health 17: 113-119.

**Kanjilal 2006**Kanjilal S, Gregg EW, Cheng YJ, Zhang P, Nelson DE, et al. (2006) Socioeconomic status and trends in disparities in 4 major risk factors for cardiovascular disease among US adults, 1971-2002. Archives of Internal Medicine 166: 2348-2355.

**Kant 2008**Kant AK, Graubard BI (2008) Ethnic and socioeconomic differences in variability in nutritional biomarkers. American Journal of Clinical Nutrition 87: 1464-1471.

**Kant 2007a**
Kant AK, Graubard BI (2007a) Secular trends in the association of socio-economic position with self-reported dietary attributes and biomarkers in the US population: National Health and Nutrition Examination Survey (NHANES) 1971-1975 to NHANES 1999-2002. Public Health Nutrition 10: 158-167.

**Kant 2007b**
Kant AK, Graubard BI (2007b) Ethnicity is an independent correlate of biomarkers of micronutrient intake and status in American adults. Journal of Nutrition 137: 2456-2463.

**Kant 2004**Kant AK (2004) Dietary patterns and health outcomes. Journal of the American Dietetic Association 104: 615-635.

**Kantomaa 2007**Kantomaa MT, Tammelin TH, Nayha S, Taanila AM (2007) Adolescents' physical activity in relation to family income and parents' education. Preventive Medicine 44: 410-415.

**Kaplan 2010**Kaplan MS, Huguet N, Feeny DH, McFarland BH (2010) Self-reported hypertension prevalence and income among older adults in Canada and the United States. Social Science & Medicine 70: 844-849.

**Karaye 2009**Karaye KM, Okeahialam BN, Wali SS (2009) Impact of income on the profile of cardiovascular risk factors among hypertensives in a Nigerian tertiary health centre: A cross-sectional study. Cardiovascular Journal of Africa 20: 251-255.

**Karvinen 2007**Karvinen KH, Courneya KS, Campbell KL, Pearcey RG, Dundas G, et al. (2007) Correlates of exercise motivation and behavior in a population-based sample of endometrial cancer survivors: An application of the Theory of Planned Behavior. International Journal of Behavioral Nutrition and Physical Activity 4.

**Katariina 2010**Katariina K, Arja H, Hannu K, Markku P, Pekka M, et al. (2010) Leisure-time physical activity and metabolic syndrome plus depressive symptoms in the FIN-D2D survey. Preventive Medicine 51: 466-470.

**Katulanda 2010**Katulanda P, Jayawardena MAR, Sheriff MHR, Constantine GR, Matthews DR (2010) Prevalence of overweight and obesity in Sri Lankan adults. Obesity Reviews 11: 751-756.

**Kaufman 1997**Kaufman PR, MacDonald JM, Lutz SM, Smallwood DM (1997) Do the poor pay more for food? Item selection and price differences affect low-income household food costs. Washington, D.C.: U.S. Department of Agriculture, Economic Research Service, Food and Rural Economics Division.

**Kaushal 2009**Kaushal N, Gao Q (2009) Food Stamp Program and consumption choices. Cambridge, MA: National Bureau of Economic Reserach.

**Kavanagh 2010**Kavanagh A, Bentley RJ, Turrell G, Shaw J, Dunstan D, et al. (2010) Socioeconomic position, gender, health behaviours and biomarkers of cardiovascular disease and diabetes. Social Science & Medicine 71: 1150-1160.

**Kelishadi 2003**Kelishadi R, Pour NH, Sarraf-Zadegan N, Sadry Gholam H, Ansari R, et al. (2003) Obesity and associated modifiable environmental factors in Iranian adolescents: Isfahan Healthy Heart Program - Heart Health Promotion from Childhood. Pediatrics International 45: 435-442.

**Keller 1997**Keller HH, Ostbye T, Bright-See E (1997) Predictors of dietary intake in Ontario seniors. Canadian Journal of Public Health Revue Canadienne de Sante Publique 88: 305-309.

**Keng 2005**Keng S-H, Lin C-H (2005) Wives' value of time and food consumed away from home in Taiwan. Asian Economic Journal 19: 319-334.

**Kennedy 1993**Kennedy ET, Oniang'o R (1993) Household and preschooler vitamin A consumption in southwestern Kenya. Journal of Nutrition 123: 841-846.

**Khan 1997**Khan LK, Sobal J, Martorell R (1997) Acculturation, socioeconomic status, and obesity in Mexican Americans, Cuban Americans, and Puerto Ricans. International Journal of Obesity & Related Metabolic Disorders: Journal of the International Association for the Study of Obesity 21: 91-96.

**Khongsdier 2002**Khongsdier R (2002) Body mass index and morbidity in adult males of the War Khasi in northeast India. European Journal of Clinical Nutrition 56: 484-489.

**Kim 2010**Kim J, Lee JS, Shin A, Kang MH, Shin DS, et al. (2010) Sociodemographic and lifestyle factors are associated with the use of dietary supplements in a Korean population. Journal of epidemiology / Japan Epidemiological Association 20: 197-203.

**Kim 2008**Kim D, Kawachi I, Hoorn SV, Ezzati M (2008) Is inequality at the heart of it? Cross-country associations of income inequality with cardiovascular diseases and risk factors. Social Science & Medicine 66: 1719-1732.

**Kim 2006**Kim D, Kawachi I (2006) Food taxation and pricing strategies to "thin out" the obesity epidemic. American Journal of Preventive Medicine 30: 430-437.

**Kimm 1996**Kimm SY, Obarzanek E, Barton BA, Aston CE, Similo SL, et al. (1996) Race, socioeconomic status, and obesity in 9- to 10-year-old girls: the NHLBI Growth and Health Study. Annals of Epidemiology 6: 266-275.

**King 2006**King T, Kavanagh AM, Jolley D, Turrell G, Crawford D (2006) Weight and place: A multilevel cross-sectional survey of area-level social disadvantage and overweight/obesity in Australia. International Journal of Obesity 30: 281-287.

**Kirkpatrick 2007**Kirkpatrick SI, Tarasuk V (2007) Adequacy of food spending is related to housing expenditures among lower-income Canadian households. Public Health Nutrition 10: 1464-1473.

**Kirkpatrick 2003**Kirkpatrick S, Tarasuk V (2003) The relationship between low income and household food expenditure patterns in Canada. Public Health Nutrition 6: 589-597.

**Kjolhede 1995**Kjolhede CL, Stallings RY, Dibley MJ, Sadjimin T, Dawiesah S, et al. (1995) Serum retinol levels among preschool children in Central Java: Demographic and socioeconomic determinants. International Journal of Epidemiology 24: 399-403.

**Klein-Platat 2003**Klein-Platat C, Wagner A, Haan MC, Arveiler D, Schlienger JL, et al. (2003) Prevalence and sociodemographic determinants of overweight in young French adolescents. Diabetes/Metabolism Research and Reviews 19: 153-158.

**Klonaris 2003**Klonaris S, Hallam D (2003) Conditional and unconditional food demand elasticities in a dynamic multistage demand system. Applied Economics 35: 503-514.

**Koch 2010**Koch E, Romero T, Romero CX, Akel C, Manriquez L, et al. (2010) Impact of education, income and chronic disease risk factors on mortality of adults: Does 'a pauper-rich paradox' exist in Latin American societies? Public Health 124: 39-48.

**Kohrs 1979**Kohrs MB, Wang LL, Eklund D, Paulsen B, O'Neal R (1979) The association of obesity with socioeconomic factors in Missouri. The American Journal of Clinical Nutrition 32: 2120-2128.

**Koplan 1986**Koplan JP, Annest JL, Layde PM, Rubin GL (1986) Nutrient intake and supplementation in the United States (NHANES II). American Journal of Public Health 76: 287-289.

**Kranz 2008**Kranz S, Findeis JL, Shrestha SS (2008) Uso do Indice de Qualidade da Dieta Infantil Revisado para avaliar a dieta alimentar de pre-escolares, seus preditores sociodemogra ficos e sua associacao com peso corporal [Use of the Revised Children's Diet Quality Index to assess preschooler's diet quality, its sociodemographic predictors, and its association with body weight status]. Jornal de Pediatria 84: 26-34.

**Kranz 2002**Kranz S, Siega-Riz Anna M (2002) Sociodemographic determinants of added sugar intake in preschoolers 2 to 5 years old. Journal of Pediatrics 140: 667-672.

**Krishna 1996**Krishna A, Johar GV (1996) Consumer perceptions of deals: Biasing effects of varying deal prices. Journal of Experimental Psychology: Applied 2: 187-206.

**Krosnick 2003**Krosnick JA, Anand SN, Hartl SP (2003) Psychosocial predictors of heavy television viewing among preadolescents and adolescents. Basic and Applied Social Psychology 25: 87-110.

**Kruger 2006**Kruger R, Kruger HS, MacIntyre UE (2006) The determinants of overweight and obesity among 10- to 15-year-old schoolchildren in the North West Province, South Africa - The THUSA BANA (Transition and Health during Urbanisation of South Africans; BANA, children) study. Public health nutrition 9: 351-358.

**Kruger 2002**Kruger HS, Venter CS, Vorster HH, Margetts BM (2002) Physical inactivity is the major determinant of obesity in black women in the North West Province, South Africa: The THUSA study (Transition and Health During Urbanisation of South Africa). Nutrition 18: 422-427.

**Kuchler 2005**Kuchler F, Tegene A, Harris JM (2005) Taxing snack foods: Manipulating diet quality or financing information programs? Applied Economic Perspectives and Policy 27: 4-20.

**Kuchler 2004**Kuchler F, Tegene A, Harris JM (2004) Taxing snack foods: What to expect for diet and tax revenues. Agricultural Information Bulletins 747: 1-11.

**Kuchler 2002**Kuchler F, Lin B-H (2002) The influence of individual choices and attitudes on adiposity. International Journal of Obesity 26: 1017-1022.

**Kuhle 2008**Kuhle S, Veugelers PJ (2008) Why does the social gradient in health not apply to overweight? Health reports / Statistics Canada, Canadian Centre for Health Information - Rapports sur la sante / Statistique Canada, Centre canadien d'information sur la sante 19: 7-15.

**Kulaga 2010**Kulaga Z, Litwin M, Pan H, Feber J, Wojcik P, et al. (2010) Lifestyle and socioeconomic determinants of blood pressure in school-aged children and adolescents. Journal of Hypertension 28: e399.

**Kumanyika 2006**Kumanyika S, Grier S (2006) Targeting interventions for ethnic minority and low-income populations. The Future of Children 16: 187-207.

**Kuntz 2010**Kuntz B, Lampert T (2010) Soziookonomische faktoren und verbreitung von adipositas. [Socioeconomic factors and the distribution of obesity]. Deutsches Arzteblatt 107: 517-522.

**Kwon 2010**Kwon Y, Oh S, Park S, Park Y (2010) Association between household income and overweight of Korean and American children: Trends and differences. Nutrition Research 30: 470-476.

**Laaksonen 2003**
Laaksonen, M., Prattala Laaksonen M, Prattala R, Helasoja V, Uutela A, Lahelma E (2003) Income and health behaviours: Evidence from monitoring surveys among Finnish adults. Journal of Epidemiology and Community Health 57: 711-717.

**Lacanilao 2011**Lacanilao RD, Cash SB, Adamowicz WL (2011) Heterogeneous consumer responses to snack food taxes and warning labels. Journal of Consumer Affairs 45: 108-122. [*Primary reference*]

Cash SB, Lacanilao RD (2008) An experimental investigation of the impact of fat taxes: Prices effects, food stigma, and information effects on economics instruments to improve dietary health. Edmonton, AB, Canada: Department of Rural Economy, University of Alberta.

**Lachapelle 2009**Lachapelle U, Frank LD (2009) Transit and health: Mode of transport, employer-sponsored public transit pass programs, and physical activity. Journal of Public Health Policy 30: s73-s94.

**Lachat 2009**Lachat C, Khanh LNB, Khan NC, Dung NQ, Do Van A, et al. (2009) Eating out of home in Vietnamese adolescents: Socioeconomic factors and dietary associations. American Journal of Clinical Nutrition 90: 1648-1655.

**LaFrance 2008**LaFrance JT (2008) The structure of US food demand. Journal of Econometrics 147: 336-349.

**LaFrance 1999**LaFrance J (1999) An econometric model of the demand for food and nutrition. Berkeley, CA: Department of Agricultural and Resource Economics, University of California, Berkeley

**Lakdawalla 2009**Lakdawalla D, Philipson T (2009) The growth of obesity and technological change. Economics & Human Biology 7: 283-293. [*Primary reference*]

Lakdawalla D, Philipson T (2002) The growth of obesity and technological change: A theoretical and empirical examination. Cambridge, MA: National Bureau of Economic Research.

**Lakdawalla 2005**Lakdawalla D, Philipson T, Bhattacharya J (2005) Welfare-enhancing technological change and the growth of obesity. The American Economic Review 95: 253-257.

**Lake Snell Perry Associates 2003**Lake Snell Perry Associates (2003) Obesity as a public health issue: A look at solutions. Oakland, CA: Lake Snell Perry & Associates.

**Lakka 1996**Lakka TA, Kauhanen J, Salonen JT (1996) Conditioning leisure time physical activity and cardiorespiratory fitness in sociodemographic groups of middle-ages men in eastern Finland. International Journal of Epidemiology 25: 86-93.

**Lallukka 2010**Lallukka T, Pitkaniemi J, Rahkonen O, Roos E, Laaksonen M, et al. (2010) The association of income with fresh fruit and vegetable consumption at different levels of education. European Journal of Clinical Nutrition 64: 324-327.

**Lamerz 2005**Lamerz A, Kuepper-Nybelen J, Wehle C, Bruning N, Trost-Brinkhues G, et al. (2005) Social class, parental education, and obesity prevalence in a study of six-year-old children in Germany. International Journal of Obesity 29: 373-380.

**Lanfranco 2002**Lanfranco BA, Ames GCW, Huang CL (2002) Food expenditure patterns of the Hispanic population in the United States. Agribusiness 18: 197-211.

**Larsen 2009**Larsen K, Gilliland J, Hess P, Tucker P, Irwin J, et al. (2009) The influence of the physical environment and sociodemographic characteristics on children's mode of travel to and from school. American Journal of Public Health 99: 520-526.

**Larson 2011**Larson NI, Story MT (2011) Food insecurity and weight status among U.S. children and families: A review of the literature. American Journal of Preventive Medicine 40: 166-173.

**Lauderdale 2000**Lauderdale DS, Rathouz PJ (2000) Body mass index in a US national sample of Asian Americans: Effects of nativity, years since immigration and socioeconomic status. International Journal of Obesity 24: 1188-1194.

**Lawlor 2005**Lawlor DA, O'Callaghan MJ, Mamun AA, Williams GM, Bor W, et al. (2005) Socioeconomic position, cognitive function, and clustering of cardiovascular risk factors in adolescence: Findings from the Mater University study of pregnancy and its outcomes. Psychosomatic Medicine 67: 862-868.

**Lazaridis 2000**Lazaridis P (2000) Decomposition of food expenditure inequality: An application of the extended Gini coefficient to Greek micro-data. Social Indicators Research 52: 179-193.

**Le 2007**Le C, Chongsuvivatwong V, Geater A (2007) Contextual socioeconomic determinants of cardiovascular risk factors in rural south-west China: A multilevel analysis. BioMed Central Public Health 7: 72.

**Le Grand 2009**
Le Grand J, Titmuss R (2009) Incentives for prevention. London: Health England. 34 p.

**Lee 2010**Lee K, Lim HT, Hwang SS, Chae DW, Park SM (2010) Socio-economic disparities in behavioural risk factors for cancer and use of cancer screening services in Korean adults aged 30 years and older: The Third Korean National Health and Nutrition Examination Survey, 2005 (KNHANES III). Public Health 124: 698-704.

**Lee 2009**Lee DS, Chiu M, Manuel DG, Tu K, Wang X, et al. (2009) Trends in risk factors for cardiovascular disease in Canada: Temporal, socio-demographic and geographic factors. Canadian Medical Association Journal 181: e55-e66.

**Leis 2010**Leis KS, Reeder BA, Chad KE, Spink KS, Fisher KL, et al. (2010) The relationship of chronic disease and demographic variables to physical activity in a sample of women aged 65 to 79 years. Women & Health 50: 459-474.

**Leon 2007**Leon M, Younger SD (2007) Transfer payments, mothers' income and child health in Ecuador. Journal of Development Studies 43: 1126-1143.

**Lera-López 2005**Lera-López F, Rapún-Gárate M (2005) Sports participation versus consumer expenditure on sport: Different determinants and strategies in sports management. European Sport Management Quarterly 5: 167-186.

**Leroy 2010**Leroy JL, Gadsden P, Rodriguez-Ramierz S, González de Cossío T (2010) Cash and in-kind transfers in poor rural communities in Mexico increase household fruit, vegetable, and micronutrient consumption but also lead to excess energy Consumption. The Journal of Nutrition.

**Leung 2010**Leung CW, Villamor E (2011) Is participation in food and income assistance programmes associated with obesity in California adults? Results from a state-wide survey. Public Health Nutrition 14: 645-652.

**Levi 2010**Levi J, Vinter S, St. Laurent R, Segal LM (2010) F as in fat: How obesity threatens America's future. Washington, DC: Trust for America's Health, Robert Wood Johnson Foundation. 121 p.

**Levy 2011a**
Levy DT, Mabry PL, Wang YC, Gortmaker S, Huang TTK, et al. (2011a) Simulation models of obesity: A review of the literature and implications for research and policy. Obesity Reviews 12: 378-394.

**Levy 2011b**
Levy DT, Friend KB, Wang YC (2011b) A review of the literature on policies directed at the youth consumption of sugar sweetened beverages. Advances in Nutrition: An International Review Journal 2: s182-s200.

**Li 2010**Li J, Hooker NH (2010) Childhood obesity and schools: Evidence from the National Survey of Children's Health. Journal of School Health 80: 96-103.

**Lima-Costa 2003**Lima-Costa MF, Barreto SM, Firmo JOA, Uchoa E (2003) Socioeconomic position and health in a population of Brazilian elderly: The Bambui Health and Aging Study (BHAS). Pan American Journal of Public Health 13: 387-394.

**Lin 2010a**
Lin B-H, Yen ST, Dong D, Smallwood DM (2010a) Economic incentives for dietary improvement among food stamp recipients. Contemporary Economic Policy 28: 524-536.

**Lin 2010b**
Lin BH, Smith TA (2010b) The effects of a sugar-sweetened beverage tax: Consumption, calorie intake, obesity, and tax burden by income. Agricultural & Applied Economics Association 2010 AAEA, CAES, & WAEA Joint Annual Meeting. Denver, Colorado: United States Department of Agriculture, Economic Research Service.

**Lin 2007**Lin CC, Liu CS, Lai MM, Li CI, Chen CC, et al. (2007) Metabolic syndrome in a Taiwanese metropolitan adult population. BioMed Central Public Health 7.

**Lin 2004**Lin BH, Huang CL, French SA (2004) Factors associated with women's and children's body mass indices by income status. International Journal of Obesity and Related Metabolic Disorders: Journal for the International Association for the Study of Obesity 28: 536-542.

**Lindsey 2006**Lindsey G, Han Y, Wilson J, Yang J (2006) Neighborhood correlates of urban trail use. Journal of Physical Activity & Health 3: s139-s157.

**Linn 1989**Linn S, Fulwood R, Rifkind B, Carroll M, Muesing R, et al. (1989) High density lipoprotein cholesterol levels among us adults by selected demographic and socioeconomic variables: The Second National Health and Nutrition Examination Survey 1976-1980. American Journal of Epidemiology 129: 281-294.

**Lino 2002**Lino M (2002) The quality of young children's diets. Family Economics and Nutrition Review 14: 52-60.

**Linz 2005**Linz P, Lee M, Bell L (2005) Obesity, poverty, and participation in Nutrition Assistance Programs. Alexandria, VA: United States Department of Agriculture, Food and Nutrition Service.

**Liu 2007a**
Liu Y, Shankar B (2007a) Will rising household incomes solve China's micronutrient deficiency problems? Economics Bulletin 15: 1-14.

**Liu 2006**Liu Y, Zhai F, Popkin BM (2006) Trends in eating behaviours among Chinese children (1991 -1997). Asia Pacific Journal of Clinical Nutrition 15: 72-80.

**Lo 2009**Lo Y-T, Chang Y-H, Lee M-S, Wahlqvist ML (2009) Health and nutrition economics: Diet costs are associated with diet quality. Asia Pacific Journal of Clinical Nutrition 18: 598-604.

**Logan 2006**Logan TD (2006) Food, nutrition, and substitution in the late nineteenth century. Explorations in Economic History 43: 527-545.

**Longmuir 2011**Longmuir PE, Russell JL, Corey M, Faulkner G, McCrindle BW (2011) Factors associated with the physical activity level of children who have the Fontan procedure. American Heart Journal 161: 411-417.

**Longo 2009**Longo GZ, Neves J, Luciano VM, Peres MA (2009) Prevalence of high blood pressure levels and associated factors among adults in Southern Brazil. Arquivos Brasileiros de Cardiologia 93: 387-394, 380.

**Lopez 2012**Lopez RA, Fantuzzi KL (2012) Demand for carbonated soft drinks: Implications for obesity policy. Applied Economics 44: 2859-2865.

**Lopez 2007**Lopez RP (2007) Neighborhood risk factors for obesity. Obesity 15: 2111-2119.

**Lordan 2011**Lordan G, Quiggin J (2011) Should we put a thin subsidy on the policy table in the fight against obesity? Forum for Health Economics & Policy 14: Article 1.

**Lorson 2009**Lorson BA, Melgar-Quinonez HR, Taylor CA (2009) Correlates of fruit and vegetable intakes in US children. Journal of the American Dietetic Association 109: 474-478.

**Loucks 2007a**Loucks EB, Magnusson KT, Cook S, Rehkopf DH, Ford ES, et al. (2007a) Socioeconomic position and the metabolic syndrome in early, middle, and late life: Evidence from NHANES 1999-2002. Annals of Epidemiology 17: 782-790. [Primary reference]

Loucks EB, Rehkopf DH, Thurston RC, Kawachi I (2007b) Socioeconomic disparities in metabolic syndrome differ by gender: Evidence from NHANES III. Annals of Epidemiology 17: 19-26.

**Loureiro 2005**Loureiro ML, Nayga RM (2005) International dimensions of obesity and overweight related problems: An economics perspective. American Journal of Agricultural Economics 87: 1147-1153.

**Lovasi 2009**Lovasi GS, Hutson MA, Guerra M, Neckerman KM (2009) Built environments and obesity in disadvantaged populations. Epidemiologic Reviews 31: 7-20.

**Lowry 1996**Lowry R, Kann L, Collins Janet L, Kolbe Lloyd J (1996) The effect of socioeconomic status on chronic disease risk behaviors among us adolescents. Journal of the American Medical Association 276: 792-797.

**Lu 2002**Lu N, Samuels ME, Huang K-C (2002) Dietary behavior in relation to socioeconomic characteristics and self-perceived health status. Journal of Health Care for the Poor and Underserved 13: 241-257.

**Lucove 2007**Lucove JC, Huston SL, Evenson KR (2007) Workers' perceptions about worksite policies and environments and their association with leisure-time physical activity. American Journal of Health Promotion 21: 196-200.

**Luepker 1993**Luepker RV, Rosamond WD, Murphy R, Sprafka JM, Folsom AR, et al. (1993) Socioeconomic status and coronary heart disease risk factor trends: The Minnesota Heart survey. Circulation 88: 2172-2179.

**Ma 2006**Ma H, Huang J, Fuller F, Rozelle S (2006) Getting rich and eating out: Consumption of food away from home in urban China. Canadian Journal of Agricultural Economics 54: 101-119.

**Ma 2004a**Ma H, Rae A, Huang J, Rozelle S (2004a) Chinese animal product consumption in the 1990s. Australian Journal of Agricultural and Resource Economics 48: 569-590.

**MacFarlane 2009**MacFarlane AM, Abbott GR, Crawford DA, Ball K (2009) Sociodemographic and behavioural correlates of weight status among women with children living in socioeconomically disadvantaged neighbourhoods. International Journal of Obesity 33: 1289-1298.

**Mack 2008**Mack F, Abeygunawardhana N, Mundt T, Schwahn C, Proff P, et al. (2008) The factors associated with body mass index in adults from the study of health in Pomerania (SHIP-0), Germany. Journal of Physiology and Pharmacology 59: 5-16.

**MacLeod 2008**MacLeod KE, Gee GC, Crawford P, Wang MC (2008) Neighbourhood environment as a predictor of television watching among girls. Journal of Epidemiology and Community Health 62: 288-292.

**Maddock 2004**Maddock J (2004) The relationship between obesity and the prevalence of fast food eestaurants: State-level analysis. American Journal of Health Promotion 19: 137-143.

**Madore 2007**Madore O (2007) The impact of economic instruments that promote healthy eating, encourage physical activity and combat obesity: Literature review. Ottawa, ON: Library of Parliament.

**Makinen 2009**Makinen T, Borodulin K, Laatikainen T, Fogelholm M, Prattala R (2009) Twenty-five year socioeconomic trends in leisure-time and commuting physical activity among employed Finns. Scandinavian Journal of Medicine & Science in Sports 19: 188-197.

**Mancino 2007**Mancino L, Newman C (2007) Who has time to cook? How family resources influence food preparation Alexandria, VA: US Depatrment of Agriculture, Economic Research Service.

**Manrique 1998**Manrique J, Jensen HH (1998) Working women and expenditures on food away-from-home and at-home in Spain. Journal of Agricultural Economics 49: 321-333.

**Martin 2008**Martin AR, Nieto JMM, Ruiz JPN, Jimenez LE (2008) Overweight and obesity: The role of education, employment and income in Spanish adults. Appetite 51: 266-272.

**Martin 2007**Martin KS, Ferris AM (2007) Food insecurity and gender are risk factors for obesity. Journal of Nutrition Education and Behavior 39: 31-36.

**Matson-Koffman 2005**Matson-Koffman DM, Brownstein JN, Neiner JA, Greaney ML (2005) A site-specific literature review of policy and environmental interventions that promote physical activity and nutrition for cardiovascular health: What works? American Journal of Health Promotion 19: 167-193.

**Matthews 2002**Matthews KA, Kiefe CI, Lewis CE, Liu K, Sidney S, et al. (2002) Socioeconomic trajectories and incident hypertension in a biracial cohort of young adults. Hypertension 39: 772-776.

**Maurer 1984**Maurer KM (1984) The National Evaluation of School Nutrition Programs: Program impact on family food expenditures. American Journal of Clinical Nutrition 40: 448-453.

**Mazur 2003**Mazur RE, Marquis GS, Jensen HH (2003) Diet and food insufficiency among Hispanic youths: Acculturation and socioeconomic factors in the third National Health and Nutrition Examination Survey. American Journal of Clinical Nutrition 78: 1120-1127.

**McCracken 1987**McCracken VA, Brandt JA (1987) Household consumption of food-away-from-home: Total expenditure and by type of food facility. American Journal of Agricultural Economics 69: 274-284.

**McDonald 2008**McDonald NC (2008) Critical factors for active transportation to school among low-income and minority students: Evidence from the 2001 National Household Travel Survey. American Journal of Preventive Medicine 34: 341-344.

**McGrath 2006**McGrath JJ, Matthews KA, Brady SS (2006) Individual versus neighborhood socioeconomic status and race as predictors of adolescent ambulatory blood pressure and heart rate. Social Science & Medicine 63: 1442-1453.

**McInnes 2009**McInnes MM, Shinogle JJ (2009) Physical activity: Economic and policy factors. Cambridge, MA: National Bureau of Economic Research.

**McNutt 1997**McNutt SW, Yuanreng H, Schreiber GB, Crawford PB, Obarzanek E, et al. (1997) A longitudinal study of the dietary practices of black and white girls 9 and 10 years old at enrollment: The NHLBI Growth and Health Study. Journal of Adolescent Health 20: 27-37.

**Meeks 1990**Meeks CB, Mauldin T (1990) Children's time in structured and unstructured leisure activities. Lifestyles: Family and Economic Issues 11: 257-281.

**Mehta 2008**Mehta NK, Chang VW (2008) Weight status and restaurant availability: A multilevel analysis. American Journal of Preventive Medicine 34: 127-133.

**Melgar-Quinonez 2004**Melgar-Quinonez HR, Kaiser LL (2004) Relationship of child-feeding practices to overweight in low-income Mexican-American preschool-aged children. Journal of the American Dietetic Association 104: 1110-1119.

**Mellor 2011**Mellor JM (2011) Do cigarette taxes affect children's body mass index? The effect of household environment on health. Health Economics 20: 417-431.

**Meltzer 2010**Meltzer DO, Jena AB (2010) The economics of intense exercise. Journal of Health Economics 29: 347-352.

**Meltzer 2009**Meltzer DO, Chen Z (2009) The impact of minimum wage rates on body weight in the United States. Cambridge, MA: National Bureau of Economic Research

**Mendez 2005**Mendez MA, Monteiro CA, Popkin BM (2005) Overweight exceeds underweight among women in most developing countries. American Journal of Clinical Nutrition 81: 714-721.

**Mendez 2004**Mendez MA, Cooper RS, Luke A, Wilks R, Bennett F, et al. (2004) Higher income is more strongly associated with obesity than with obesity-related metabolic disorders in Jamaican adults. International Journal of Obesity & Related Metabolic Disorders: Journal of the International Association for the Study of Obesity 28: 543-550.

**Menezes 2008**
Menezes TA, Azzoni CR, Silveira FG (2008) Demand elasticities for food products in Brazil: A two-stage budgeting system. Applied Economics 40: 2557-2572.

**Mergenthaler 2009**Mergenthaler M, Weinberger K, Qaim M (2009) The food system transformation in developing countries: A disaggregate demand analysis for fruits and vegetables in Vietnam. Food Policy 34: 426-436.

**Metcalf 2008**Metcalf PA, Scragg RR, Schaaf D, Dyall L, Black PN, et al. (2008) Comparison of different markers of socioeconomic status with cardiovascular disease and diabetes risk factors in the Diabetes, Heart and Health Survey. New Zealand Medical Journal 121: 45-56.

**Metcalf 2007**Metcalf P, Scragg R, Davis P (2007) Relationship of different measures of socioeconomic status with cardiovascular disease risk factors and lifestyle in a New Zealand workforce survey. New Zealand Medical Journal 120.

**Meyerhoefer 2006**Meyerhoefer CD, Pylypchuk Y (2006) Does participation in the Food Stamp Program increase the prevalence of obesity and health care spending? Rockville, MD: Center for Financing, Access and Cost Trends and Agency for Healthcare Research and Quality. [*Primary reference*]

Meyerhoefer CD, Pylypchuk Y (2008) Does participation in the Food Stamp Program increase the prevalence of obesity and health care spending? American Journal of Agricultural Economics 90: 287-305.

**Meyers 1995**Meyers A, Frank DA, Roos N, Peterson KE, Casey VA, et al. (1995) Housing subsidies and pediatric undernutrition. Archives of Pediatrics and Adolescent Medicine 149: 1079-1084.

**Mfenyana 2006**Mfenyana K, Griffin M, Yogeswaran P, Modell B, Modell M, et al. (2006) Socio-economic inequalities as a predictor of health in South Africa: The Yenza cross-sectional study. South African Medical Journal Suid-Afrikaanse Tydskrif Vir Geneeskunde 96: 323-330.

**Michalek 1992**Michalek J, Keyzer MA (1992) Estimation of a two-stage LES-AIDS consumer demand system for eight EC countries. European Review of Agricultural Economics 19: 137-163.

**Michaud 2007**Michaud P-C, van Soest A, Andreyeva T (2007) Cross-country variation in obesity patterns among older Americans and Europeans. Santa Monica, CA: RAND Centre for the Study of Aging.

**Michels 2008**Michels KB, Bloom BR, Riccardi P, Rosner BA, Willett WC (2008) A study of the importance of education and cost incentives on individual food choices at the Harvard School of Public Health cafeteria. Journal of the American College of Nutrition 27: 6-11.

**Michimi 2010**Michimi A, Wimberly MC (2010) Associations of supermarket accessibility with obesity and fruit and vegetable consumption in the conterminous United States. International Journal of Health Geographics 9: 1-14.

**Mihalopoulos 2001**Mihalopoulos VG, Demoussis MP (2001) Greek household consumption of food away from home: A microeconometric approach. European Review of Agricultural Economics 28: 421-432.

**Miljkovic 2008a**
Miljkovic D, Nganje W (2008a) Regional obesity determinants in the United States: A model of myopic addictive behavior in food consumption. Agricultural Economics 38: 375-384.

**Miljkovic 2008b**
Miljkovic D, Nganje W, de Chastenet H (2008b) Economic factors affecting the increase in obesity in the United States: Differential response to price. Food Policy 33: 48-60.

**Miller 2007**Miller JC, Coble KH (2007) Cheap food policy: Fact or rhetoric? Food Policy 32: 98-111.

**Miller 2005**Miller RR, Sales AE, Kopjar B, Fihn SD, Bryson CL (2005) Adherence to heart-healthy behaviors in a sample of the U.S. population. Preventing Chronic Disease 2: A18.

**Millstone 2007**Millstone E, Lobstein T (2007a) The PorGrow project: Overall cross-national results, comparisons and implications. Obesity Reviews 8: 29-36.

Borg P, Fogelholm M (2007) Stakeholder appraisal of policy options for responding to obesity in Finland. Obesity Reviews 8: 47-52.

Codrington C, Sarri K, Kafatos A (2007) Stakeholder appraisal of policy options for tackling obesity in Greece. Obesity Reviews 8: 63-73.

De Marchi B, Casati S, Tarlao G (2007) Stakeholder appraisal of policy options for responding to obesity in Italy. Obesity Reviews 8: 83-89.

González-Zapata LI, Alvarez-Dardet C, Millstone E, Clemente-Gómez V, Holdsworth M, et al. (2010) The potential role of taxes and subsidies on food in the prevention of obesity in Europe. Journal of Epidemiology and Community Health 64: 696-704.

González-Zapata LI, Ortiz-Moncada R, Alvarez-Dardet C (2007) Mapping public policy options responding to obesity: The case of Spain. Obesity Reviews 8: 99-108.

Holdsworth M, Kameli Y, Delpeuch F (2007) Stakeholder views on policy options for responding to the growing challenge from obesity in France: Findings from the PorGrow project. Obesity Reviews 8: 53-61.

Horváth Z, Pankotai MG, Szabolcs I (2007) Stakeholder appraisal of policy options for responding to obesity in Hungary. Obesity Reviews 8: 75-81.

Lobstein T, Millstone E, PorGrow Research Team (2007) Context for the PorGrow study: Europe’s obesity crisis. Obesity Reviews 8: 7-16.

Millstone E, Lobstein T, PorGrow National T (2007b) The PorGrow project – an introduction and overview. Obesity Reviews 8: 5-6.

Mohebati L, Lobstein T, Millstone E, Jacobs M (2007) Policy options for responding to the growing challenge from obesity in the United Kingdom. Obesity Reviews 8: 109-115.

Savva SC, Chadjioannou M, Tornaritis MJ (2007) Policy options for responding to the growing challenge from obesity: Cyprus national findings. Obesity Reviews 8: 37-45.

Stirling A, Lobstein T, Millstone E (2007) Methodology for obtaining stakeholder assessments of obesity policy options in the PorGrow project. Obesity Reviews 8: 17-27.

Szponar L, Ciok J, Dolna A, Oltarzewski M (2007) Policy options for responding to the growing challenge from obesity (PorGrow) in Poland. Obesity Reviews 8: 91-98.

**Minaker 2006**Minaker LM, McCargar L, Lambriki I, Jessup L, Driezen P, et al. (2006) School region socio-economic status and geographic locale is associated with food behaviour of Ontario and Alberta adolescents. Canadian Journal of Public Health 97: 357-361.

**Mion 2004**Mion DJ, Pierin AM, Bambirra AP, Assuncao JH, Monteiro JM, et al. (2004) Hypertension in employees of a University General Hospital. Revista do Hospital das Clinicas 59: 329-336.

**Mishra 2011**Mishra A, Mishra H (2011) The influence of price discount versus bonus pack on the preference for virtue and vice foods. Journal of Marketing Research 48: 196-206.

**Mohr 2007**Mohr P, Wilson C, Dunn K, Brindal E, Wittert G (2007) Personal and lifestyle characteristics predictive of the consumption of fast foods in Australia. Public Health Nutrition 10: 1456-1463.

**Monsivais 2009**Monsivais P, Drewnowski A (2009) Lower-energy-density diets are associated with higher monetary costs per kilocalorie and are consumed by women of higher socioeconomic status. Journal of the American Dietetic Association 109: 814-822.

**Monteiro 2004**Monteiro CA, Moura EC, Conde WL, Popkin BM (2004) Socioeconomic status and obesity in adult populations of developing countries: A review. Bulletin of the World Health Organization 82: 940-946.

**Monteiro 2003**Monteiro CA, Conde WL, Matsudo SM, Matsudo VR, Bonsenor IM, et al. (2003) A descriptive epidemiology of leisure-time physical activity in Brazil, 1996-1997. Revista Panamericana de Salud Publica/Pan American Journal of Public Health 14: 246-254.

**Monteiro 2002**Monteiro CA, Conde WL, Popkin BM (2002) Is obesity replacing or adding to undernutrition? Evidence from different social classes in Brazil. Public Health Nutrition 5: 105-112.

**Moore 2008**Moore LV, Diez Roux AV, Nettleton JA, Jacobs DR (2008) Associations of the local food environment with diet quality: A comparison of assessments based on surveys and geographic information systems. American Journal of Epidemiology 167: 917-924. [*Primary reference*]

Moore LV, Diez Roux AV, Nettleton JA, Jacobs DR, Franco M (2009) Fast-food consumption, diet quality, and neighborhood exposure to fast food. American Journal of Epidemiology 170: 29-36.

**Moreira 2006**Moreira P, Padrao P (2006) Educational, economic and dietary determinants of obesity in Portuguese adults: A cross-sectional study. Eating Behaviors 7: 220-228.

**Moschonis 2010**Moschonis G, Tanagra S, Vandorou A, Kyriakou AE, Dede V, et al. (2010) Social, economic and demographic correlates of overweight and obesity in primary-school children: Preliminary data from the Healthy Growth Study. Public Health Nutrition 13: 1693-1700.

**Moshfegh 1999**Moshfegh AJ, Friday JE, Goldman JP, Chug A, J K (1999) Presence of inulin and oligofructose in the diets of Americans. Journal of Nutrition 129: s1407-s1411.

**Muennig 2007**Muennig P, Sohler N, Mahato B (2007) Socioeconomic status as an independent predictor of physiological biomarkers of cardiovascular disease: Evidence from NHANES. Preventive Medicine 45: 35-40.

**Mullie 2010**Mullie P, Clarys P, Hulens M, Vansant G (2010) Dietary patterns and socioeconomic position. European Journal of Clinical Nutrition 64: 231-238.

**Mungreiphy 2010**Mungreiphy NK, Kapoor S (2010) Socioeconomic changes as covariates of overweight and obesity among Tangkhul Naga tribal women of Manipur, north-east India. Journal of Biosocial Science 42: 289-305.

**Murasko 2011**Murasko JE (2011) Trends in the associations between family income, height and body mass index in US children and adolescents: 1971--1980 and 1999--2008. Annals of Human Biology 38: 290-306.

**Murasko 2008**Murasko JE (2008) Male-female differences in the association between socioeconomic status and atherosclerotic risk in adolescents. Social Science & Medicine 67: 1889-1897.

**Musgrove 1985**Musgrove P (1985) Household food consumption in the Dominican Republic: Effects of income, price, and family size. Economic Development and Cultural Change 34: 83-101.

**Mushi-Brunt 2007**Mushi-Brunt C, Haire-Joshu D, Elliott M (2007) Food spending behaviors and perceptions are associated with fruit and vegetable intake among parents and their preadolescent children. Journal of Nutrition Education and Behavior 39: 26-30.

**Myllykangas 1995**Myllykangas M, Pekkanen J, Rasi V, Haukkala A, Vahtera E, et al. (1995) Haemostatic and other cardiovascular risk factors, and socioeconomic status among middle-aged Finnish men and women. International Journal of Epidemiology 24: 1110-1116.

**Mytton 2007**Mytton O, Gray A, Rayner M, Rutter H (2007) Could targeted food taxes improve health? Journal of Epidemiology and Community Health 61: 689-694.

**Nagata 2009**Nagata JM, Valeggia CR, Barg FK, Bream KDW (2009) Body mass index, socio-economic status and socio-behavioral practices among Tz'utujil Maya women. Economics and Human Biology 7: 96-106.

**Najdi 2011**Najdi A, El Achhab Y, Nejjari C, Norat T, Zidouh A, et al. (2011) Correlates of physical activity in Morocco. Preventive Medicine 52: 355-357.

**Nayga 1996a**
Nayga RMJ (1996a) Wife's labor force participation and family expenditures for prepared food, food prepared at home, and food away from home. Agricultural and Resource Economics Review 25: 179-186.

**Nayga 1996b**
Nayga RMJ (1996b) Dietary fiber intake away-from-home and at-home in the United States. Food Policy 21: 279-290.

**Nayga 1994a**Nayga RM, Capps O (1994a) Analysis of away-from-home and at-home intake of saturated fat and cholesterol. Review of Agricultural Economics 16: 387-398.

**Nazmi 2008**Nazmi A, Oliveira IO, Victora CG (2008) Correlates of C-reactive protein levels in young adults: A population-based cohort study of 3827 subjects in Brazil. Brazilian Journal of Medical and Biological Research 41: 357-367. [*Primary reference*]

Nazmi A, Oliveira IO, Horta BL, Gigante DP, Victora CG (2010) Lifecourse socioeconomic trajectories and C-reactive protein levels in young adults: Findings from a Brazilian birth cohort. Social Science & Medicine 70: 1229-1236.

**Nebeling 1997**Nebeling LC, Forman MR, Graubard BI, Snyder RA (1997) Changes in carotenoid intake in the United States: The 1987 and 1992 National Health Interview Surveys. Journal of the American Dietetic Association 97: 991-996.

**Nederkoorn 2011**Nederkoorn C, Havermans RC, Giesen JCAH, Jansen A (2011) High tax on high energy dense foods and its effects on the purchase of calories in a supermarket. An experiment. Appetite 56: 760-765.

**Nelson 2002**Nelson KM, Reiber G, Boyko EJ (2002) Diet and exercise among adults with type 2 diabetes: Findings from the third national health and nutrition examination survey (NHANES III). Diabetes Care 25: 1722-1728.

**Nelson 2000**Nelson M (2000) Childhood nutrition and poverty. Proceedings of the Nutrition Society 59: 307-315.

**Ng 2010a**
Ng SW, Norton EC, Guilkey DK, Popkin BM (2010a) Estimation of a dynamic model of weight. Cambridge, MA: National Bureau of Economic.

**Ng 2010b**
Ng C, Young TK, Corey PN (2010b) Associations of television viewing, physical activity and dietary behaviours with obesity in aboriginal and non-aboriginal Canadian youth. Public Health Nutrition 13: 1430-1437.

**Ni Mhurchu 2011**Ni Mhurchu C, Eyles H, Dixon R, Matoe L, Teevale T, et al. (2011) Economic incentives to promote healthier food purchases: Exploring acceptability and key factors for success. Health Promotion International.

**Ni Mhurchu 2010**Ni Mhurchu C, Blakely T, Jiang Y, Eyles HC, Rodgers A (2010) Effects of price discounts and tailored nutrition education on supermarket purchases: A randomized controlled trial. American Journal of Clinical Nutrition 91: 736-747.

**Nies 2002**Nies MA, Kershaw TC (2002) Psychosocial and environmental influences on physical activity and health outcomes in sedentary women. Journal of Nursing Scholarship 34: 243-249.

**Nikolaou 2008**Nikolaou A, Nikolaou D (2008) Income-related inequality in the distribution of obesity among Europeans. Journal of Public Health 16: 403-411.

**Niskar 2003**Niskar AS, Paschal DC, Kieszak SM, Flegal KM, Bowman B, et al. (2003) Serum selenium levels in the US population: Third National Health and Nutrition Examination Survey, 1988-1994. Biological Trace Element Research 91: 1-10.

**Nnoaham 2009**Nnoaham KE, Sacks G, Rayner M, Mytton O, Gray A (2009) Modelling income group differences in the health and economic impacts of targeted food taxes and subsidies. International Journal of Epidemiology 38: 1324-1333.

**Nocon 2007**Nocon M, Keil T, Willich SN (2007) Education, income, occupational status and health risk behaviour. Journal of Public Health 15: 401-405.

**Noppa 1980**Noppa H, Bengtsson C (1980) Obesity in relation to socioeconomic status: A population study of women in Goteborg, Sweden. Journal of Epidemiology and Community Health 34: 139-142. [*Primary reference*]

Noppa H, Hallstrom T (1981) Weight gain in adulthood in relation to socioeconomic factors, mental illness and personality traits: A prospective study of middle-aged women. Journal of Psychosomatic Research 25: 83-89.

**Nordström 2011**
Nordström J, Thunström L (2011) Economic policies for healthier food intake: The impact on different household categories. The European Journal of Health Economics 12: 127-140.

**Nordström 2009**Nordström J, Thunström L (2009) The impact of tax reforms designed to encourage healthier grain consumption. Journal of Health Economics 28: 622-634.

**Nube 1998**Nube M, Asenso-Okyere WK, van den Boom GJM (1998) Body mass index as indicator of standard of living in developing countries. European Journal of Clinical Nutrition 52: 136-144.

**Oaks 2005**Oaks B (2005) An evaluation of the snack tax on the obesity rate of Maine [Masters of Public Administration thesis]. San Marcos, TX: Texas State University.

**O'Dea 2008**O'Dea JA (2008) Gender, ethnicity, culture and social class influences on childhood obesity among Australian schoolchildren: Implications for treatment, prevention and community education. Health & Social Care in the Community 16: 282-290.

**O'Dea 2006**O'Dea JA, Wilson R (2006) Socio-cognitive and nutritional factors associated with body mass index in children and adolescents: Possibilities for childhood obesity prevention. Health Education Research 21: 796-805.

**Offord 1998**Offord D, Lipman E, Duku E (1998) Sports, the arts and community programs: Rates and correlates of participation. Hull, Quebec: Applied Research Branch, Strategic Policy, and Human Resources Development Canada.

**Oksuz 2008**Oksuz E (2008) Unhealthy body perception among Turkish youths: Socioeconomic status and social comparisons. Collegium Antropologicum 32: 5-13.

**Oliveira 2000**Oliveira V, Gundersen C (2000) WIC and the nutrient intake of children. Washington, DC: US Department of Agriculture, Economic Research Service, Food and Rural Economics Division.

**Oliver 2008**Oliver LN, Hayes MV (2008) Effects of neighbourhood income on reported body mass index: An eight year longitudinal study of Canadian children. BioMed Central Public Health 8.

**Olsen 2000**Olsen WK, Warde A, Martens L (2000) Social differentiation and the market for eating out in the UK. International Journal of Hospitality Management 19: 173-190.

**Oreffice 2010**Oreffice S, Quintana-Domeque C (2010) Anthropometry and socioeconomics among couples: Evidence in the United States. Economics and Human Biology 8: 373-384.

**Oyewole 2007**Oyewole P (2007) Fast food marketing and the African American consumers: The impact of socio-economic and demographic characteristics. Journal of International Consumer Marketing 19: 75-108.

**Paeratakul 2002**Paeratakul S, Lovejoy JC, Ryan DH, Bray GA (2002) The relation of gender, race and socioeconomic status to obesity and obesity comorbidities in a sample of US adults. International Journal of Obesity & Related Metabolic Disorders 26: 1205-1210.

**Pan 2009**Pan S, Jensen HH (2008) Does the Food Stamp Program affect food security status and the composition of food expenditures? Journal of Agricultural and Applied Economics 30: 21–35.

**Pan 2008**Pan SY, Cameron C, Desmeules M, Morrison H, Craig CL, et al. (2009) Individual, social, environmental, and physical environmental correlates with physical activity among Canadians: A cross-sectional study. BioMed Central Public Health 9.

**Papandreou 2008**Papandreou C, Mourad TA, Jildeh C, Abdeen Z, Philalithis A, et al. (2008) Obesity in Mediterranean region (1997-2007): A systematic review. Obesity Reviews 9: 389-399.

**Park 2009**Park J (2009) Obesity on the job. Perspectives on Labour and Income 21: 37-45.

**Park 1997**Park JL, Capps O (1997) Demand for prepared meals by U.S. households. American Journal of Agricultural Economics 79: 814-824.

**Park 1996**Park JL, Holcomb RB, Raper KC, Capps O (1996) A demand systems analysis of food commodities by U.S. households segmented by income. American Journal of Agricultural Economics 78: 290-300.

**Parks 2011**Parks JC, Smith AD, Alston JM (2011) The effects of the Food Stamp Program on energy balance and obesity. Agricultural & Applied Economics Association’s 2011 AAEA & NAREA Joint Annual Meeting. Pittsburgh, Pennsylvania: Department of Agriculture and Resource Economics, University of California, Davis.

**Parks 2003**Parks SE, Housemann RA, Brownson RC (2003) Differential correlates of physical activity in urban and rural adults of various socioeconomic backgrounds in the United States. Journal of Epidemiology and Community Health 57: 29-35.

**Pascual 2007**Pascual C, Regidor E, Astasio P, Ortega P, Navarro P, et al. (2007) The association of current and sustained area-based adverse socioeconomic environment with physical inactivity. Social Science & Medicine 65: 454-466.

**Patt 2004**
Patt MR, Yanek LR, Moy TF, Becker DM (2004) Sociodemographic, behavioral, and psychological correlates of current overweight and obesity in older, urban African American women. Health Education & Behavior 31: s57-s68.

**Patterson 1988**Patterson BH, Block G (1988) Food choices and the cancer guidelines. American Journal of Public Health 78: 282-286.

**Paulin 2000**Paulin GD (2000) Let's do lunch: Expenditures on meals away from home. Monthly Labor Review 123: 36-45.

**Pearson 2005**Pearson T, Russell J, Campbell MJ, Barker ME (2005) Do 'food deserts' influence fruit and vegetable consumption? A cross-sectional study. Appetite 45: 195-197.

**Peixoto 2007**Peixoto MDRG, Benicio MHD, Jardim PCBV (2007) The relationship between body mass index and lifestyle in a Brazilian adult population: A cross-sectional survey. Cadernos de Saude Publica 23: 2694-2704.

**Perez-Escamilla** **2000**Perez-Escamilla R, Ferris AM, Drake L, Haldeman L, Peranick J, et al. (2000) Food stamps are associated with food security and dietary intake of inner-city preschoolers from Hartford, Connecticut. Journal of Nutrition 130: 2711-2717.

**Perrin 2005**Perrin A-E, Dallongeville J, Ducimetiere P, Ruidavets J-B, Schlienger J-L, et al. (2005) Interactions between traditional regional determinants and socio-economic status on dietary patterns in a sample of French men. British Journal of Nutrition 93: 109-114.

**Phillips 2010**Phillips AC, Carroll D, Thomas GN, Gale CR, Deary I, et al. (2010) The influence of multiple indices of socioeconomic disadvantage across the adult life course on the metabolic syndrome: The Vietnam Experience study. Metabolism: Clinical and Experimental 59: 1164-1171.

**Pickett 2005**Pickett KE, Kelly S, Brunner E, Lobstein T, Wilkinson RG (2005) Wider income gaps, wider waistbands? An ecological study of obesity and income inequality. Journal of Epidemiology and Community Health 59: 670-674.

**Piggott 2003**Piggott NE (2003) The nested PIGLOG model: An application to U.S. food demand. American Journal of Agricultural Economics 85: 1-15.

**Pittman 2004**Pittman GF (2004) Drivers of demand, interrelationships, and nutritional impacts within the nonalcoholic beverage complex [Doctoral dissertation]. College Station, TX: Texas A&M University.

**Platat 2006**Platat C, Perrin A-E, Oujaa M, Wagner A, Haan M-C, et al. (2006) Diet and physical activity profiles in French preadolescents. British Journal of Nutrition 96: 501-507.

**Pleis 2003**Pleis JR, Schiller JS, Benson V (2003) Summary health statistics for U.S. adults: National Health Interview Survey, 2000. Vital & Health Statistics - Series 10: Data From the National Health Survey: 1-132.

**Pomerleau 2000**Pomerleau J, McKee M, Robertson A, Vaasc S, Kadziauskiene K, et al. (2000) Physical inactivity in the Baltic countries. Preventive Medicine 31: 665-672. [*Primary reference*]

Pomerleau J, Pudule I, Grinberga D, Kadziauskiene K, Abaravicius A, et al. (2000) Patterns of body weight in the Baltic Republics. Public Health Nutrition 3: 3-10.

**Pomerleau 1997**Pomerleau J, Pederson LL, Ostbye T, Speechley M, Speechley KN (1997) Health behaviours and socio-economic status in Ontario, Canada. European Journal of Epidemiology 13: 613-622.

**Pont 2009**Pont K, Ziviani J, Wadley D, Bennett S, Abbott R (2009) Environmental correlates of children's active transportation: A systematic literature review. Health & Place 15: 827-840.

**Popham 2006**Popham F, Mitchell R (2006) Leisure time exercise and personal circumstances in the working age population: Longitudinal analysis of the British household panel survey. Journal of Epidemiology and Community Health 60: 270-274.

**Popkin 2003**Popkin BM, Zizza C, Siega-Riz Anna M (2003) Who is leading the change? U.S. dietary quality comparison between 1965 and 1996. American Journal of Preventive Medicine 25: 1-8.

**Posner 1987**Posner BM, Ohls JC, Morgan JC (1987) The impact of food stamps and other variables on nutrient intake in the elderly. Journal of Nutrition for the Elderly 6: 3-16.

**Pouyanne 2005**Pouyanne G (2005) Urban form and travel patterns: An application to the metropolitan area of Bordeaux. Canadian Journal of Regional Science 28: 19-47.

**Powell 2011**Powell LM, Han E (2011) The costs of food at home and away from home and consumption patterns among U.S. adolescents. Journal of Adolescent Health 48: 20-26.

**Powell 2010**Powell LM, Han E, Chaloupka FJ (2010) Economic contextual factors, food consumption, and obesity among U.S. adolescents. Journal of Nutrition 140: 1175-1780.

**Powell 2009a**
Powell LM (2009a) Fast food costs and adolescent body mass index: Evidence from panel data. Journal of Health Economics 28: 963-970.

**Powell 2009b**
Powell LM, Bao Y (2009b) Food prices, access to food outlets and child weight. Economics & Human Biology 7: 64-72.

**Powell 2009c**
Powell LM, Chriqui J, Chaloupka FJ (2009c) Associations between state-level soda taxes and adolescent body mass index. Journal of Adolescent Health 45: s57-s63.

**Powell 2009d**
Powell LM, Chaloupka FJ (2009d) Economic contextual factors and child body mass index. Cambridge, MA: National Bureau of Economic Research.

**Powell 2009e**
Powell LM, Chaloupka FJ (2009e) Food prices and obesity: Evidence and policy implications for taxes and subsidies. Milbank Quarterly 87: 229-257.

**Powell 2009f**
Powell LM, Zhao Z, Wang Y (2009f) Food prices and fruit and vegetable consumption among young American adults. Health & Place 15: 1064-1070.

**Powell 2007**Powell LM, Auld C, Chaloupka FJ, O’Malley PM, Johnston LD (2007) Access to fast food and food prices: Relationship with fruit and vegetable consumption and overweight among adolescents. Advances in Health Economics and Health Services Research 17: 23-48.

**Power 2005**Power EM (2005) Determinants of healthy eating among low-income Canadians. Canadian Journal of Public Health Revue Canadienne de Sante Publique 96 s37-42, s42.

**Pradeepa 2003**Pradeepa R, Deepa R, Rani SS, Premalatha G, Saroja R, et al. (2003) Socioeconomic status and dyslipidaemia in a South Indian population: The Chennai Urban Population Study (CUPS 11). National Medical Journal of India 16: 73-78.

**Prasad 2008**Prasad A, Strijnev A, Zhang Q (2008) What can grocery basket data tell us about health consciousness? International Journal of Research in Marketing 25: 301-309.

**Pratt 1999**Pratt M, Macera CA, Blanton C (1999) Levels of physical activity and inactivity in children and adults in the United States: Current evidence and research issues. Medicine and Science in Sports and Exercise 31: s526-s533.

**Proper 2007**Proper KI, Cerin E, Brown WJ, Owen N (2007) Sitting time and socio-economic differences in overweight and obesity. International Journal of Obesity 31: 169-176.

**Pudaric 2000**Pudaric S, Sundquist J, Johansson SE (2000) Major risk factors for cardiovascular disease in elderly migrants in Sweden. Ethnicity and Health 5: 137-350.

**Raberg 2010**Raberg KMK, Holmboe-Ottesen G, Wandel M (2010) Associations between food patterns, socioeconomic position and working situation among adult, working women and men in Oslo. European Journal of Clinical Nutrition 64: 1150-1157.

**Rabin 2006**Rabin BA, Boehmer TK, Brownson RC (2006) Cross-national comparison of environmental and policy correlates of obesity in Europe. The European Journal of Public Health 17: 53-61.

**Radimer 2000**Radimer KL, Subar AF, Thompson FE (2000) Nonvitamin, nonmineral dietary supplements: Issues and findings from NHANES III. Journal of the American Dietetic Association 100: 447-454.

**Raffensperger 2010**Raffensperger S, Kuczmarski MF, Hotchkiss L, Cotugna N, Evans MK, et al. (2010) Effect of race and predictors of socioeconomic status on diet quality in the HANDLS Study sample. Journal of the National Medical Association 102: 923-930.

**Raine 2005**Raine KD (2005) Determinants of healthy eating in Canada: An overview and synthesis. Canadian Journal of Public Health Revue Canadienne de Sante Publique 96 s8-14, s18.

**Ramachandran 2002**Ramachandran A, Snehalatha C, Vijay V, King H (2002) Impact of the poverty on the prevalence of diabetes and its complications in urban southern India. Diabetic Medicine 19: 130-135.

**Ramezani 1995**Ramezani CA (1995) Determinants of nutrient demand: A nonparametric analysis. Journal of Agricultural and Resource Economics 20: 165-177.

**Ranjit 2007**Ranjit N, Diez-Roux AV, Shea S, Cushman M, Ni H, et al. (2007) Socioeconomic position, race/ethnicity, and inflammation in the multi-ethnic study of atherosclerosis. Circulation 116: 2383-2390.

**Ransdell 1998**Ransdell LB, Wells CL (1998) Physical activity in urban white, African American, and Mexican-American women. Medicine and Science in Sports and Exercise 30: 1608-1615.

**Rashad 2009**Rashad I (2009) Associations of cycling with urban sprawl and the gasoline price. American Journal of Health Promotion 24: 27-36. [*Primary reference*]

Rashad I (2007) Cycling: An increasingly untouched source of physical and mental health. Cambridge, MA: National Bureau of Economic Research.

**Rashad 2006a**
Rashad I, Grossman M, Chou S-Y (2006a) The super size of America: An economic estimation of body mass index and obesity in adults. Eastern Economic Journal 32: 133-148. [*Primary reference*]

Rashad I, Grossman M, Chou S-Y (2005) The super size of America: An economice estimation of body mass index and obesity in adults. Cambridge, MA: Natonal Bureau of Economic Reserach.

**Rashad 2006b**
Rashad I (2006b) Structural estimation of caloric intake, exercise, smoking, and obesity. The Quarterly Review of Economics and Finance 46: 268-283.

**Rasmussen 2006**Rasmussen M, Krolner R, Klepp KI, Lytle L, Brug J, et al. (2006) Determinants of fruit and vegetable consumption among children and adolescents: A review of the literature. Part I: Quantitative studies. International Journal of Behavioral Nutrition and Physical Activity 3.

**Ree 2008**Ree M, Riediger N, Moghadasian MH (2008) Factors affecting food selection in Canadian population. European Journal of Clinical Nutrition 62: 1255-1262.

**Reed 2010**Reed AJ, Levedahl JW (2010) Food stamps and the market demand for food. American Journal of Agricultural Economics 92: 1392-1400.

**Reedy 2010**Reedy J, Krebs-Smith SM (2010) Dietary sources of energy, solid fats, and added sugars among children and adolescents in the United States. Journal of the American Dietetic Association 110: 1477-1484.

**Rehkopf 2010**Rehkopf DH, Krieger N, Coull B, Berkman LF (2010) Biologic risk markers for coronary heart disease: Nonlinear associations with income. Epidemiology 21: 38-46.

**Rehm 2008**Rehm CD, Matte TD, Van Wye G, Young C, Frieden TR (2008) Demographic and behavioral factors associated with daily sugar-sweetened soda consumption in New York City adults. Journal of Urban Health 85: 375-385.

**Resnicow 2001**Resnicow K, Wang T, Dudley WN, Jackson A, Ahluwalia JS, et al. (2001) Risk factor distribution among sociodemographically diverse African American adults. Journal of Urban Health 78: 125-140.

**Ricciuto 2006**Ricciuto L, Tarasuk V, Yatchew A (2006) Socio-demographic influences on food purchasing among Canadian households. European Journal of Clinical Nutrition 60: 778-790. [*Primary reference*]

Ricciuto LE, Tarasuk VS (2007) An examination of income-related disparities in the nutritional quality of food selections among Canadian households from 1986-2001. Social Science & Medicine 64: 186-198.

**Richards 2009**Richards TJ, Padilla L (2009) Promotion and fast food demand. American Journal of Agricultural Economics 91: 168-183.

**Riediger 2008**Riediger ND, Moghadasian MH (2008) Patterns of fruit and vegetable consumption and the influence of sex, age and socio-demographic factors among Canadian elderly. Journal of the American College of Nutrition 27: 306-313.

**Riediger 2007**Riediger ND, Shooshtari S, Moghadasian MH (2007) The influence of sociodemographic factors on patterns of fruit and vegetable consumption in Canadian adolescents. Journal of the American Dietetic Association 107: 1511-1518.

**Risica 2009**Risica PM, Kerr S, Lawson E, Belhumeur R, Ankoma A (2009) Self-report of sugar-sweetened beverage and fast food consumption by annual household income. Medicine & Health, Rhode Island 92: 63-64.

**Robert 2004**Robert SA, Reither EN (2004) A multilevel analysis of race, community disadvantage, and body mass index among adults in the US. Social Science & Medicine 59: 2421-2434.

**Robinson 2011**Robinson CA, Zheng X (2011) Household Food Stamp Program participation and childhood pbesity. Journal of Agricultural and Resource Economics 36: 1-13.

**Robinson 2009**Robinson CA (2009) Three essays in health economics [Doctoral dissertation]. Raliegh, NC, United States: North Caroline State University. 1-55 p.

**Robinson 2007**Robinson KT (2007) The applicability and usage of the International Classification of Functioning, Disability and Health (ICF) to address obesity among United States women [Doctoral dissertation]. Pittsburgh, PA, United States: University of Pittsburgh.

**Rodrigues 2008**Rodrigues PL, Lacerda EMdA, Schlussel MM, Spyrides MHC, Kac G (2008) Determinants of weight gain in pregnant women attending a public prenatal care facility in Rio de Janeiro, Brazil: A prospective study, 2005-2007. Cadernos de Saude Publica 24 s272-s284.

**Roos 2008**
Roos E, Talala K, Laaksonen M, Helakorpi S, Rahkonen O, et al. (2008) Trends of socioeconomic differences in daily vegetable consumption, 1979-2002. European Journal of Clinical Nutrition 62: 823-833.

**Rose 2004**Rose D, Richards R (2004) Food store access and household fruit and vegetable use among participants in the US Food Stamp Program. Public Health Nutrition 7: 1081-1088.

**Rose 1998**Rose D, Habicht JP, Devaney B (1998) Household participation in the Food Stamp and WIC Programs increases the nutrient intakes of preschool children. Journal of Nutrition 128: 548-555.

**Rose 1995**Rose D, Smallwood D, Blaylock J (1995) Socio-economic factors associated with the iron intake of preschoolers in the United States. Nutrition Research 15: 1297-1309.

**Roskam 2008**Roskam A-JR, Kunst AE (2008) The predictive value of different socio-economic indicators for overweight in nine European countries. Public Health Nutrition 11: 1256-1266.

**Roy 2001**Roy N (2001) A semiparametric analysis of calorie response to income change across income groups and gender. Journal of International Trade and Economic Development 10: 93-109.

**Ruhm 2005**Ruhm CJ (2005) Healthy living in hard times. Journal of Health Economics 24: 341-363. [*Primary reference*]

Ruhm CJ (2003) Healthy living in hard times. Cambridge, MA: National Bureau of Economic Research.

**Ruiz-Arranz 2006**Ruiz-Arranz M, Davis B, Handa S, Stampini M, Winters P (2006) Program conditionality and food security: The impact of PROGRESA and PROCAMPO transfers in rural Mexico. Economia, Associação Nacional dos Centros de Pósgraduação em Economia [Brazilian Association of Graduate Programs in Economics] 7: 249-278. [*Primary reference*]

Ruiz-Arranz M, Davis B, Stampini M, Winters P, Handa S (2002) More calories or more diversity? An econometric evaluation of the impact of the PROGRESA and PROCAMPO transfer programmes on food security in rural Mexico. Rome, Italy: Agricultural and Development Economics Division, The Food and Agriculture Origanization of the United Nations.

**Rundle 2008**Rundle A, Field S, Park Y, Freeman L, Weiss CC, et al. (2008) Personal and neighborhood socioeconomic status and indices of neighborhood walk-ability predict body mass index in New York City. Social Science & Medicine 67: 1951-1958.

**Rush 1988**Rush D, Sloan NL, Leighton J, Alvir JM, Horvitz DG, et al. (1988) The National WIC Evaluation: Evaluation of the Special Supplemental Food Program for women, infants, and children. V. Longitudinal study of pregnant women. The American Journal of Clinical Nutrition 48: 439-483.

**Sabanayagam 2009**Sabanayagam C, Shankar A, Saw SM, Tai ES, Wong TY (2009) The association between socioeconomic status and overweight/obesity in a Malay population in Singapore. Asia-Pacific Journal of Public Health 21: 487-496.

**Sabanayagam 2007**Sabanayagam C, Shankar A, Wong TY, Saw SM, Foster PJ (2007) Socioeconomic status and overweight/obesity in an adult Chinese population in Singapore. Journal of epidemiology / Japan Epidemiological Association 17: 161-168.

**Sabri 2005**Sabri S, Bener A, Eapen V, Azhar AA, Abdishakure A, et al. (2005) Correlation between hypertension and income distribution among United Arab Emirates population. Medical Journal of Malaysia 60: 416-425.

**Sabri 2004**Sabri S, Bener A, Eapen V, Abu Zeid MSO, Al-Mazrouei AM, et al. (2004) Some risk factors for hypertension in the United Arab Emirates. Eastern Mediterranean Health Journal 10: 610-619.

**Sabzghabaee 2010**Sabzghabaee AM, Mirmoghtadaee P, Mohammadi M (2010) Fruit and vegetable consumption among community dwelling elderly in an Iranian population. International Journal of Preventive Medicine 1: 98-102.

**Sacks 2011**Sacks G, Veerman JL, Moodie M, Swinburn B (2011) `Traffic-light' nutrition labelling and `junk-food' tax: A modelled comparison of cost-effectiveness for obesity prevention. International Journal of Obesity 35: 1001-1009. [*Primary reference*]

Sacks G, Veerman JL, Moodie M, Swinburn B (2010a) 'Traffic-light' nutrition labelling and 'junk-food' tax: A modelled comparison of cost-effectiveness. Obesity Reviews 11: 63-64.

Sacks G (2010b) Nutrient profiling interventions targeting obesity prevention: Role and potential impact [Doctoral dissertation]. Melbourne, Australia: Deaking University. 296 p.

**Sahn 1988**Sahn DE (1988) The effect of price and income changes on food-energy intake in Sri Lanka. Economic Development and Cultural Change 36: 315-340.

**Sakamoto 2001**Sakamoto N, Wansorn S, Tontisirin K, Marui E (2001) A social epidemiologic study of obesity among preschool children in Thailand. International Journal of Obesity & Related Metabolic Disorders: Journal of the International Association for the Study of Obesity 25: 389-394.

**Sallis 2009a**
Sallis JF, Saelens BE, Frank LD, Conway TL, Slymen DJ, et al. (2009a) Neighborhood built environment and income: Examining multiple health outcomes. Social Science & Medicine 68: 1285-1293.

**Salois 2011**Salois M, Blacombe K (2011) Do food stamps cause obesity? A generalised Bayesian instrumental variable approach in the presence of heteroscedasticity. Munich, Germany: Munich Personal RePEc Archive.

**Salonen 2009**Salonen MK, Kajantie E, Osmond C, Forsen T, Ylihrsil H, et al. (2009) Role of socioeconomic indicators on development of obesity from a life course perspective. Journal of Environmental and Public Health 2009.

**Salsberry 2007**Salsberry PJ, Corwin E, Reagan PB (2007) A complex web of risks for Metabolic Syndrome: Race/ethnicity, economics, and gender. American Journal of Preventive Medicine 33: 114-120.

**Samuel-Hodge 2010**Samuel-Hodge CD, Gizlice Z, Cai J, Brantley PJ, Ard JD, et al. (2010) Family functioning and weight loss in a sample of African Americans and whites. Annals of Behavioral Medicine 40: 294-301.

**Sanchez-Vaznaugh 2009**Sanchez-Vaznaugh EV, Kawachi I, Subramanian SV, Sanchez Brisa N, Acevedo-Garcia D (2009) Do socioeconomic gradients in body mass index vary by race/ethnicity, gender, and birthplace? American Journal of Epidemiology 169: 1102-1112.

**Sarlio-Lahteenkorva 2006**Sarlio-Lahteenkorva S, Silventoinen K, Lahti-Koski M, Laatikainen T, Jousilahti P (2006) Socio-economic status and abdominal obesity among Finnish adults from 1992 to 2002. International Journal of Obesity 30: 1653-1660.

**Sarno 2009**Sarno F, Claro RM, Levy RB, Bandoni DH, Ferreira SR, et al. (2009) Estimativa de consumo de sodio pela populacao brasileira, 2002-2003 [Estimated sodium intake by the Brazilian population, 2002-2003]. Revista de Saude Publica 43: 219-225.

**Satheannoppakao 2009**Satheannoppakao W, Aekplakorn W, Pradipasen M (2009) Fruit and vegetable consumption and its recommended intake associated with sociodemographic factors: Thailand National Health Examination Survey III. Public Health Nutrition 12: 2192-2198.

**Sausenthaler 2007**
Sausenthaler S, Kompauer I, Mielck A, Borte M, Herbarth O, et al. (2007) Impact of parental education and income inequality on children's food intake. Public Health Nutrition 10: 24-33.

**Savio 2008**Savio KE, Oliveira, da Costa THM, Schmitz BdAS, da Silva EF (2008) Sex, income and level of education associated with physical activity level among workers. Revista de Saude Publica 42: 457-463.

**Scearce 1979**Scearce WK, Jensen RB (1979) Food Stamp Program effects on availability of food nutrients for low income families in the southern region of the United States. Southern Journal of Agricultural Economics 11: 113-120.

**Schanzenbach 2005**Schanzenbach D (2005) Do school lunches contribute to childhood obesity? Chicago, IL: Harris School of Public Policy, University of Chicago.

**Schindler 1992**Schindler RM (1992) A coupon is more than a low price: Evidence from a shopping-simulation study. Psychology & Marketing 9: 431-451.

**Schmeer 2010**Schmeer KK (2010) Household income during childhood and young adult weight status: Evidence from a nutrition transition setting. Journal of Health and Social Behavior 51: 79-91.

**Schmeiser 2012**Schmeiser MD (2012) The impact of long-term participation in the supplemental nutrition assistance program on child obesity. Health Economics, 21: 386-404.

**Schmeiser 2009**Schmeiser MD (2009) Expanding wallets and waistlines: The impact of family income on the BMI of women and men eligible for the Earned Income Tax Credit. Health Economics 18: 1277-1294.

**Schnoover 2006**Schnoover H, Muller M (2006) Food without thought: How U.S. Farm Policy contributes to obesity. Minneapolis, MN: Institute for Agriculture and Trade Policy. 14 p.

**Schoenborn 2002**Schoenborn CA, Adams PF, Barnes PM (2002) Body weight status of adults: United States, 1997-98. Advance Data: 1-15.

**Schroeter 2008**Schroeter C, Lusk J, Tyner W (2008) Determining the impact of food price and income changes on body weight. Journal of Health Economics 27: 45-68.

**Sear 1982**Sear AM, Weinrich M, Hersh JE, Lam JJ (1982) The relationship between income, education and hypertension. Journal of Biosocial Science 14: 213-221.

**Seeman 2008**Seeman T, Merkin SS, Crimmins E, Koretz B, Charette S, et al. (2008) Education, income and ethnic differences in cumulative biological risk profiles in a national sample of US adults: NHANES III (1988-1994). Social Science & Medicine 66: 72-87.

**Seo 2007**Seo D-C, Torabi M (2007) Differences in vigorous and moderate physical activity by gender, race/ethnicity, age, education, and income among U.S. Adults. American Journal of Health Education 38: 122-128.

**Seubsman 2010**Seubsman S-a, Lim LLY, Banwell C, Sripaiboonkit N, Kelly M, et al. (2010) Socioeconomic status, sex, and obesity in a large national cohort of 15-87-year-old open university students in Thailand. Journal of Epidemiology 20: 13-20.

**Seymour 2004b**
Seymour JD, Yaroch AL, Serdula M, Blanck HM, Khan LK (2004) Impact of nutrition environmental interventions on point-of-purchase behavior in adults: A review. Preventive Medicine 39: s108-s136.

**Shankar 2010**Shankar B (2010) Socio-economic drivers of overnutrition in China. Journal of Human Nutrition & Dietetics 23: 471-479.

**Shapo 2003**Shapo L, Pomerleau J, McKee M, Coker R, Ylli A (2003) Body weight patterns in a country in transition: A population-based survey in Tirana City, Albania. Public Health Nutrition 6: 471-477. [*Primary reference*]

Shapo L, Pomerleau J, McKee M (2004) Physical inactivity in a country in transition: A population-based survey in Tirana City, Albania. Scandinavian Journal of Public Health 32: 60-67.

**Sharkey 2011**Sharkey JR, Johnson CM, Dean WR, Horel SA (2011) Association between proximity to and coverage of traditional fast-food restaurants and nontraditional fast-food outlets and fast-food consumption among rural adults. International Journal of Health Geographics 10.

**Sharpe 2003**Sharpe DL, Huston SJ, Finke MS (2003) Factors affecting nutritional adequacy among single elderly women. Family Economics and Nutrition Review 15: 74-82.

**Shimokawa 2008**Shimokawa S, Chang H-H, Pinstrup-Andersen P (2008) Understanding the differences in obesity among working adults between Taiwan and China. Asia Pacific Journal of Clinical Nutrition 18: 88-95.

**Shrewsbury 2008**Shrewsbury V, Wardle J (2008) Socioeconomic status and adiposity in childhood: A systematic review of cross-sectional studies 1990-2005. Obesity 16: 275-284.

**Sichieri 1994**Sichieri R, Coitinho DC, Leão MM, Recine E, Everhart JE (1994) High temporal, geographic, and income variation in body mass index among adults in Brazil. American Journal of Public Health 84: 793-798.

**Sidney 1996**Sidney S, Sternfeld B, Haskell WL, Jacobs DRJ, Chesney MA, et al. (1996) Television viewing and cardiovascular risk factors in young adults: The CARDIA study. Annals of Epidemiology 6: 154-159.

**Siega-Riz 2004**Siega-Riz AM, Kranz S, Blanchette D, Haines PS, Guilkey DK, et al. (2004) The effect of participation in the WIC program on preschoolers' diets. Journal of Pediatrics 144: 229-234.

**Siegel 1995**Siegel PZ, Brackbill RM, Heath GW (1995) The epidemiology of walking for exercise: Implications for promoting activity among sedentary groups. American Journal of Public Health 85: 706-710.

**Simon 1993**Simon JA, Schreiber GB, Crawford PB, Frederick MM, Sabry ZI (1993) Income and racial patterns of dietary vitamin C intake among black and white girls. Public Health Reports 108: 760-764.

**Simons-Morton 2000**Simons-Morton DG, Hogan P, Dunn AL, Pruitt L, King AC, et al. (2000) Characteristics of inactive primary care patients: Baseline data from the activity counseling trial. For the Activity Counseling Trial Research Group. Preventive Medicine 31: 513-521.

**Singh 2011**Singh GK, Siahpush M, Hiatt RA, Timsina LR (2011) Dramatic increases in obesity and overweight prevalence and body mass index among ethnic-immigrant and social class groups in the United States, 1976-2008. Journal of Community Health 36: 94-110.

**Singh 2010**Singh GK, Siahpush M, Kogan MD (2010) Rising social inequalities in US Childhood obesity, 2003-2007. Annals of Epidemiology 20: 40-52. [*Primary reference*]

Singh GK, Kogan MD, van Dyck PC (2008) A multilevel analysis of state and regional disparities in childhood and adolescent obesity in the United States. Journal of Community Health: The Publication for Health Promotion and Disease Prevention 33: 90-102.

**Sisson 2009**Sisson SB, Church TS, Martin CK, Tudor-Locke C, Smith SR, et al. (2009) Profiles of sedentary behavior in children and adolescents: The US National Health and Nutrition Examination Survey, 2001-2006. International Journal of Pediatric Obesity 4: 353-359.

**Siu 2010**Siu J, Giskes K, Turrell G (2010) Socioeconomic differences in weight-control behaviors among a population-representative group of mid-aged adults. Public Health Nutrition 14: 1768-1778.

**Skoufias 2009**Skoufias E, Di Maro V, Gonzalez-Cossio T, Rodriguez SR (2009) Nutrient consumption and household income in rural Mexico. Agricultural Economics 40: 657-675.

**Smed 2007**Smed S, Jensen JD, Denver S (2007) Socio-economic characteristics and the effect of taxation as a health policy instrument. Food Policy 32: 624-639. [*Primary reference*]

Smed S, Jensen JD, Denver S (2005) Differentiated food taxes as a tool in health and nutrition policy. XIth Congress of the European Association of Agricultural Economists 'The Future of Rural Europe in a Global Agri-Food System'. Copenhagen, Denmark: Food and Resource Economics Insititute.

**Smeets 2007**Smeets P (2007) Following tobacco and alcoholics, it is time to tax fat. Maastricht: Maastricht University, Netherlands.

**Smith 2010**Smith TA, Lin BH, Lee JY (2010) Taxing caloric sweetened beverages: Potential effects on beverage consumption, calorie intake, and obesity Alexandria, VA: US Department of Agriculture, Economic Research Service.

**Smith 2007**Smith KV, Goldman N (2007) Socioeconomic differences in health among older adults in Mexico. Social Science & Medicine 65: 1372-1385.

**Smith 1992**Smith AM, Baghurst KI (1992) Public health implications of dietary differences between social status and occupational category groups. Journal of Epidemiology and Community Health 46: 409-416.

**Spence 2010**Spence J, Holt N, Dutove J, Carson V (2010) Uptake and effectiveness of the Children's Fitness Tax Credit in Canada: The rich get richer. BioMed Central Public Health 10: 356.

**Spinney 2010**Spinney J, Millward H (2010) Time and money: A new look at poverty and the barriers to physical activity in Canada. Social Indicators Research 99: 341-356.

**St. John 2008**St. John M, Durant M, Campagna PD, Rehman LA, Thompson AM, et al. (2008) Overweight Nova Scotia children and youth: The roles of household income and adherence to Canada's Food Guide to Healthy Eating. Canadian Journal of Public Health Revue Canadienne de Sante Publique 99: 301-306.

**Stamatakis 2009**Stamatakis E, Hillsdon M, Mishra G, Hamer M, Marmot M (2009) Television viewing and other screen-based entertainment in relation to multiple socioeconomic status indicators and area deprivation: The Scottish Health Survey 2003. Journal of Epidemiology and Community Health 63: 734-740.

**Stamatakis 2005**Stamatakis E, Primatesta P, Chinn S, Rona R, Falascheti E (2005) Overweight and obesity trends from 1974 to 2003 in English children: What is the role of socioeconomic factors? Archives of Disease in Childhood 90: 999-1004.

**Staudigel 2011**
Staudigel M (2011) How (much) do food prices contribute to obesity in Russia? Economics and Human Biology 9: 133-147.

**Steenhuis 2009**Steenhuis IHM, Nooy SBC, Moes MJG, Schuit AJ (2009) Financial barriers and pricing strategies related to participation in sports activities: The perceptions of people of low income. Journal of Physical Activity and Health 6: 716-721.

**Stelmach 2004a**Stelmach W, Kaczmarczyk-Chalas K, Bielecki W, Stelmach I, Drygas W (2004a) How income and education contribute to risk factors for cardiovascular disease in the elderly in a former Communist country. Public Health 118: 439-449.

**Stewart 2008**Stewart H, Blisard N (2008) Who pays more for food? Journal of Agricultural Economics 59: 150-168.

**Stewart 2004**Stewart H, Yen ST (2004) Changing household characteristics and the away-from-home food market: A censored equation system approach. Food Policy 29: 643-658.

**Steyn 2011**Steyn NP, Nel JH, Parker W-A, Ayah R, Mbithe D (2011) Dietary, social, and environmental determinants of obesity in Kenyan women. Scandinavian Journal of Public Health 39: 88-97.

**Stookey 2001**Stookey JD, Adair L, Stevens J, Popkin BM (2001) Patterns of long-term change in body composition are associated with diet, activity, income and urban residence among older adults in China. Journal of Nutrition 131: s2433-s2440.

**Stookey 2000**Stookey JD, Wang Y, Ge K, Lin H, Popkin BM (2000) Measuring diet quality in China: The INFH-UNC-CH diet quality index. European Journal of Clinical Nutrition 54: 811-821.

**Storey 2003**Storey ML, Forshee RA, Waever AR, Sansalone WR (2003) Demographic and lifestyle factors associated with body mass index among children and adolescents. International Journal of Food Sciences & Nutrition 54: 491.

**Story 2008**Story M, Kaphingst KM, Robinson-O'Brien R, Glanz K (2008) Creating healthy food and eating environments: Policy and environmental approaches. Annual Review of Public Health 29: 253-272.

**Strauss 1999**Strauss RS, Knight J (1999) Influence of the home environment on the development of obesity in children. Pediatrics 103: e85.

**Stunkard 2004**Stunkard AJ, Berkowitz RI, Schoeller D, Maislin G, Stallings VA (2004) Predictors of body size in the first 2 y of life: A high-risk study of human obesity. International Journal of Obesity & Related Metabolic Disorders 28: 503-513.

**Sturm 2011**Sturm R, Datar A (2011) Regional price differences and food consumption frequency among elementary school children. Public Health 125: 136-141.

**Sturm 2010**Sturm R, Powell LM, Chriqui JF, Chaloupka FJ (2010a) Soda taxes, soft drink consumption, and children's body mass index. Health Affairs (Millwood) 29: 1052-1058.

**Sturm 2005**Sturm R, Datar A (2005) Body mass index in elementary school children, metropolitan area food prices and food outlet density. Public Health 119: 1059-1068. [*Primary reference*]

Sturm R, Datar A (2008) Food prices and weight gain during elementary school: 5-year update. Public Health 122: 1140-1143.

**Suchday 2005**Suchday S, Krantz DS, Gottdiener JS (2005) Relationship of socioeconomic markers to daily life ischemia and blood pressure reactivity in coronary artery disease patients. Annals of Behavioral Medicine 30: 74-84.

**Sweet 2007**Sweet E, McDade TW, Kiefe CI, Liu K (2007) Relationships between skin color, income, and blood pressure among African Americans in the CARDIA study. American Journal of Public Health 97: 2253-2259.

**Tefft 2008**Tefft N (2008) The effects of a soft drink tax on household expenditures. Lewiston, ME: Bates College.

**Temple 2006**Temple JB (2006) Household factors associated with older Australian's purchasing a varied diet: Results from household expenditure data. Nutrition & Dietetics 63: 28-35.

**Thiele 2004**Thiele S, Mensink GBM, Beitz R (2004) Determinants of diet quality. Public Health Nutrition 7: 29-37.

**Thiele 2003**Thiele S, Weiss C (2003) Consumer demand for food diversity: Evidence for Germany. Food Policy 28: 99-115.

**Thompson 2009**Thompson FE, McNeel TS, Dowling EC, Midthune D, Morrissette M, et al. (2009) Interrelationships of added sugars intake, socioeconomic status, and race/ethnicity in adults in the United States: National Health Interview Survey, 2005. Journal of the American Dietetic Association 109: 1376-1383.

**Thow 2010**Thow AM, Jan S, Leeder S, Swinburn B (2010) Efecto de la politica fiscal en la dieta, la obesidad y las enfermedades cronicas: Revision sistematica [The effect of fiscal policy on diet, obesity and chronic disease: A systematic review]. Bulletin of the World Health Organization 88: 609-614.

**Tiffin 2011**Tiffin R, Arnoult M (2011) The public health impacts of a Fat Tax. European Journal of Clinical Nutrition 65: 427-433.

**Tiffin 2010**Tiffin R, Arnoult M (2010) The demand for a healthy diet: Estimating the almost ideal demand system with infrequency of purchase. European Review of Agricultural Economics 37: 501-521.

**Tiffin 1999**Tiffin A, Tiffin R (1999) Estimates of food demand elasticities for Great Britain: 1972-1994. Journal of Agricultural Economics 50: 140-147.

**Todd 2010**Todd JE, Zhen C (2010) Can taxes on calorically sweetened beverages reduce obesity? Choices 25.

**Tomey 2005**Tomey KM, Chen DM, Wang X, Braunschweig CL (2005) Dietary intake and nutritional status of urban community-dwelling men with paraplegia. Archives of Physical Medicine & Rehabilitation 86: 664-671.

**TfL 2008**
Transport for London (2008) Central London congestion charging: Impacts monitoring. Sixth Annual Report, July 2008. London: Transport for London. [*Primary reference*]

Transport for London (2007a) Central London congestion charging: Impacts monitoring. Fifth Annual Report, July, 2007. London: Transport for London.

Transport for London (2007b) Central London Congestion Charging Scheme: Ex-post evaluation of the quantified impacts of the original scheme. London: Transport for London.

Transport for London (2006) Central London congestion charging: Impacts monitoring. Fourth Annual Report, June 2006. London: Transport for London.

Santos G, Shaffer B (2004) Preliminary results of the London Congestion Charging Scheme. Public Works Management Policy 9: 164-181.

**Trinh 2008**Trinh OTH, Nguyen ND, Dibley MJ, Phongsavan P, Bauman AE (2008) The prevalence and correlates of physical inactivity among adults in Ho Chi Minh City. BioMed Central Public Health 8.

**Trost 2002**Trost SG, Owen N, Bauman AE, Sallis JF, Brown W (2002) Correlates of adults' participation in physical activity: Review and update. Medicine and Science in Sports and Exercise 34: 1996-2001.

**Truong 2005**Truong KD, Sturm R (2005) Weight gain trends across sociodemographic groups in the United States. American Journal of Public Health 95: 1602-1606.

**Tucker 1995**Tucker K, Spiro IA, Weiss ST (1995) Variation in food and nutrient intakes among older men: Age, and other socio-demographic factors. Nutrition Research 15: 161-176.

**Tucker-Seeley 2009**Tucker-Seeley RD, Subramanian SV, Li Y, Sorensen G (2009) Neighborhood safety, socioeconomic status, and physical activity in older adults. American Journal of Preventive Medicine 37: 207-213.

**Turan 2007**Turan S, Bereket A, Furman A, Omar A, Berber M, et al. (2007) The effect of economic status on height, insulin-like growth factor (IGF)-I and IGF binding protein-3 concentrations in healthy Turkish children. European Journal of Clinical Nutrition 61: 752-758.

**Turrell 2009**Turrell G, Bentley R, Thomas LR, Jolley D, Subramanian S, et al. (2009) A multilevel study of area socio-economic status and food purchasing behaviour. Public Health Nutrition 12: 2074-2083.

**Turrell** **2003**Turrell G, Hewitt B, Patterson C, Oldenburg B (2003) Measuring socio-economic position in dietary research: Is choice of socio-economic indicator important? Public Health Nutrition 6: 191-200. [*Primary reference*]

Turrell G, Kavanagh AM (2006) Socio-economic pathways to diet: Modelling the association between socio-economic position and food purchasing behaviour. Public Health Nutrition 9: 375-383.

**Turrell 1996**Turrell G (1996) Structural, material and economic influences on the food-purchasing choices of socioeconomic groups. Australian and New Zealand Journal of Public Health 20: 611-617.

**Tzormpatzakis 2007**Tzormpatzakis N, Sleap M (2007) Participation in physical activity and exercise in Greece: A systematic literature review. International Journal of Public Health 52: 360-371.

**van Lenthe 2000**van Lenthe FJ, Droomers M, Schrijvers CT, Mackenbach JP (2000) Socio-demographic variables and 6 year change in body mass index: Longitudinal results from the GLOBE study. International Journal of Obesity & Related Metabolic Disorders 24: 1077-1084.

**van Rossem 2010**van Rossem L, Silva LM, Hokken-Koelega A, Arends LR, Moll HA, et al. (2010) Socioeconomic status is not inversely associated with overweight in preschool children. Journal of Pediatrics 157: 929-935.e921.

**Vandegrift 2004**Vandegrift D, Yoked T (2004) Obesity rates, income, and suburban sprawl: An analysis of US states. Health and Place 10: 221-229.

**Variyam 2002**Variyam JN, Blaylock J, Smallwood D (2002) Characterizing the distribution of macronutrient intake among U.S. Adults: A quantile regression approach. American Journal of Agricultural Economics 84: 454-466.

**Vatanparast 2010**Vatanparast H, Adolphe JL, Whiting SJ (2010) Socio-economic status and vitamin/ mineral supplement use in Canada. Health Reports 21: 19-25.

**Ver Ploeg 2009**Ver Ploeg M (2009) WIC and the battle against childhood overweight. Washington, DC: US Department of Agriculture. 4 p.

**Ver Ploeg 2008**Ver Ploeg M, Ralston K (2008a) Food stamps and obesity: What do we know? Alexandria, VA: US Department of Agriculture, Economic Research Service.

**Ver Ploeg 2007**Ver Ploeg M, Mancino L, Biing-Hwan L (2007) Food and Nutrition Assistance Programs and Obesity: 1976-2002. Washington, DC: US Department of Agriculture.

**Vermeersch 1984**Vermeersch J, Hanes S, Gale S (1984) The national evaluation of school nutrition programs: Program impact on anthropometric measures. American Journal of Clinical Nutrition 40: 414-424.

**Veugelersn 2005**Veugelersn PJ, Fitzgeraldn AL (2005) Prevalence of and risk factors for childhood overweight and obesity. Canadian Medical Association Journal 173: 607-613.

**Viebig 2009**Viebig RF, Pastor-Valero M, Scazufca M, Menezes PR (2009) Consumo de frutas y hortalizas por ancianos de baja renta en la ciudad de São Paulo, Sureste de Brasil [Fruit and vegetable intake among low income elderly in the city of Sao Paulo, Southeastern Brazil]. Revista de Saude Publica 43: 806-813.

**Vieweg 2007**Vieweg VR, Johnston CH, Lanier JO, Fernandez A, Pandurangi AK (2007) Correlation between high risk obesity groups and low socioeconomic status in school children. Southern Medical Journal 100: 8-13.

**Villa-Caballero 2006**Villa-Caballero L, Caballero-Solano V, Chavarria-Gamboa M, Linares-Lomeli P, Torres-Valencia E, et al. (2006) Obesity and socioeconomic status in children of Tijuana. American Journal of Preventive Medicine 30: 197-203.

**Viner 2005**Viner RM, Cole TJ (2005) Adult socioeconomic, educational, social, and psychological outcomes of childhood obesity: A National Birth Cohort study. British Medical Journal 330: 1354-1357.

**von Laer Tschudin 2009**von Laer Tschudin L, Chattopadhyay C, Pandit S, Schramm-Garaj K, Seth U, et al. (2009) Risk factors for under- and overweight in school children of a low income area in Kolkata, India. Clinical Nutrition 28: 538-542.

**von Tigerstrom 2011**von Tigerstrom B, Larre T, Sauder J (2011) Using the tax system to promote physical activity: Critical analysis of Canadian initiatives. American Journal of Public Health 101: e10-e16.

**Wamala 1997**Wamala SP, Wolk A, Schenck-Gustafsson K, Orth-Gomer K (1997) Lipid profile and socioeconomic status in healthy middle aged women in Sweden. Journal of Epidemiology & Community Health 51: 400-407.

**Wang 2011**Wang S, Xu L, Jonas JB, You QS, Wang YX, et al. (2011) Prevalence and associated factors of dyslipidemia in the adult Chinese population. PLoS ONE 6: e17326.

**Wang 2010**Wang YC (2010) The potential impact of sugar-sweetened beverage taxes in New York state. New York, NY: Mailman School of Public Health, Columbia University.

**Wang 2009**Wang Y, Li J, Caballero B (2009) Resemblance in dietary intakes between urban low-income African-American adolescents and their mothers: The healthy eating and active lifestyles from school to home for kids study. Journal of the American Dietetic Association 109: 52-63.

**Wang 2008**Wang Z, Zhai F, Du S, Popkin B (2008) Dynamic shifts in Chinese eating behaviors. Asia Pacific Journal of Clinical Nutrition 17: 123-130.

**Wang 2006**Wang Y, Zhang Q (2006) Are American children and adolescents of low socioeconomic status at increased risk of obesity? Changes in the association between overweight and family income between 1971 and 2002. American Journal of Clinical Nutrition 84: 707-716.

**Wang 2002a**
Wang Y, Bentley ME, Zhai F, Popkin BM (2002a) Tracking of dietary intake patterns of Chinese from childhood to adolescence over a six-year follow-up period. Journal of Nutrition 132: 430-438.

**Wang 2002b**
Wang Z, Patterson CM, Hills AP (2002b) Association between overweight or obesity and household income and parental body mass index in Australian youth: Analysis of the Australian National Nutrition Survey, 1995. Asia Pacific Journal of Clinical Nutrition 11: 200-205.

**Washburn 1997**Washburn R, Hedrick BN (1997) Descriptive epidemiology of physical activity in university graduates with locomotor disabilities. International Journal of Rehabilitation Research 20: 275-287.

**Washburn 1992**Washburn RA, Kline G, Lackland DT, Wheeler FC (1992) Leisure time physical activity: Are there black/white differences? Preventive Medicine 21: 127-135.

**Waterlander 2010a**
Waterlander WE, de Mul A, Schuit AJ, Seidell JC, Steenhuis IHM (2010a) Perceptions on the use of pricing strategies to stimulate healthy eating among residents of deprived neighbourhoods: A focus group study. International Journal of Behavioral Nutrition and Physical Activity 7. [*Primary reference*]

Waterlander WE, Steenhuis IHM, de Vet E, Schuit AJ, Seidell JC (2010b) Expert views on most suitable monetary incentives on food to stimulate healthy eating. The European Journal of Public Health 20: 325-331.

**Waterlander 2010c**
Waterlander WE, de Haas WE, van Amstel I, Schuit AJ, Twisk JW, et al. (2010c) Energy density, energy costs and income – how are they related? Public Health Nutrition 13: 1599-1608.

**Webb 2008**Webb AL, Schiff A, Currivan D, Villamor E (2008) Food Stamp Program participation but not food insecurity is associated with higher adult BMI in Massachusetts residents living in low-income neighbourhoods. Public Health Nutrition 11: 1248-1255.

**Wendt 2010**Wendt M, Todd J (2010) Do low prices for sugar-sweetened beverages increase children’s weights? (Poster). Agricultural & Applied Economics Association 2010 AAEA, CAES, & WAEA Joint Annual Meeting. Denver, Colorado: US Department of Agriculture.

**Wendt 2009**Wendt MHD (2009) Economic, environmental, and endowment effects on childhood obesity and school performance [Doctoral dissertation]. Minneapolis, MN, United States: University of Minnesota.

**Whitaker 1994**Whitaker RC, Wright JA, Koepsell TD, Finch AJ, Psaty BM (1994) Characteristics of children selecting low-fat foods in an elementary school lunch program. Archives of Pediatrics and Adolescent Medicine 148: 1085-1091.

**Whitfield 1982**Whitfield RA (1982) A nutritional analysis of the Food Stamp Program. American Journal of Public Health 72: 793-799.

**Whitt-Glover 2007**Whitt-Glover MC, Taylor WC, Heath GW, Macera CA (2007) Self-reported physical activity among Blacks: Estimates from national surveys. American Journal of Preventive Medicine 33: 412-417.

**Wilde 2000**Wilde PE, Ranney CK (2000) The monthly food stamp cycle: Shopping frequency and food intake decisions in an endogenous switching regression framework. American Journal of Agricultural Economics 82: 200-213.

**Wilde 1999**Wilde PE, McNamara PE, Ranney CK (1999) The effect of income and food programs on dietary quality: A seemingly unrelated regression analysis with error components. American Journal of Agricultural Economics 81: 959-971. [*Primary reference*]

Wilde PE, McNamara PE, Ranney CK (2000) The effect on dietary quality of participation in the Food Stamp and WIC Programs. Washington, DC: US Department of Agriculture, Economic Research Service, Food and Rural Economics Division.

**Willms 2003**Willms JD, Tremblay MS, Katzmarzyk PT (2003) Geographic and demographic variation in the prevalence of overweight Canadian children. Obesity Research 11: 668-673.

**Wilson 2000**Wilson DK, Kliewer W, Plybon L, Sica DA (2000) Socioeconomic status and blood pressure reactivity in healthy black adolescents. Hypertension 35: 496-500.

**Wilson 1993**Wilson TW, Kaplan GA, Kauhanen J, Cohen RD, Wu M, et al. (1993) Association between plasma fibrinogen concentration and five socioeconomic indices in the Kuopio Ischemic Heart Disease Risk Factor Study. American Journal of Epidemiology 137: 292-300.

**Winkleby 1992**Winkleby MA, Jatulis DE, Frank E, Fortmann SP (1992) Socioeconomic status and health: How education, income, and occupation contribute to risk factors for cardiovascular disease. American Journal of Public Health 82: 816-820.

**Winters 2007**Winters M, Friesen MC, Koehoorn M, Teschke K (2007) Utilitarian bicycling: A multilevel analysis of climate and personal influences. American Journal of Preventive Medicine 32: 52-58.

**Wolfe 1997**Wolfe WS, Sobal J, Olson CM, Frongillo EAJ, Williamson DF (1997) Parity-associated weight gain and its modification by sociodemographic and behavioral factors: A prospective analysis in US women. International Journal of Obesity & Related Metabolic Disorders: Journal of the International Association for the Study of Obesity 21: 802-810.

**Wolfe 1983**Wolfe BL, Behrman JR (1983) Is income overrated in determining adequate nutrition? Economic Development and Cultural Change 31: 525-549.

**Wolin 2008**Wolin KY, Bennett GG, McNeill LH, Sorensen G, Emmons KM (2008) Low discretionary time as a barrier to physical activity and intervention uptake. American Journal of Health Behavior 32: 563-569.

**Wong 2002**Wong J, Wong S (2002) Trends in lifestyle cardiovascular risk factors in women: Analysis from the Canadian National Population Health Survey. International Journal of Nursing Studies 39: 229-242.

**Worsley 2003**Worsley A, Blasche R, Ball K, Crawford D (2003) Income differences in food consumption in the 1995 Australian National Nutrition Survey. European Journal of Clinical Nutrition 57: 1198-1211.

**Xie 2003**Xie B, Gilliland FD, Li Y-F, Rockett HR (2003) Effects of ethnicity, family income, and education on dietary intake among adolescents. Preventive Medicine: An International Journal Devoted to Practice and Theory 36: 30-40.

**Xu 2005**Xu F, Yin X-M, Zhang M, Leslie E, Ware R, et al. (2005) Family average income and body mass index above the healthy weight range among urban and rural residents in regional Mainland China. Public Health Nutrition 8: 47-51.

**Yang 2010**Yang C-C, Chiou W-B (2010) Substitution of healthy for unhealthy beverages among college students: A health-concerns and behavioral-economics perspective. Appetite 54: 512-516.

**Yarnoff 2010**Yarnoff B (2010) The effectiveness and efficiency of a soft drink tax for reducing calorie consumption. Chicago: University of Illinois at Chicago.

**Yen 2010**Yen ST (2010) The effects of SNAP and WIC programs on nutrient intakes of children. Food Policy 35: 576-583.

**Yen 2004a**Yen ST, Lin BH, Smallwood DM, Andrews M (2004a) Demand for nonalcoholic beverages: The case of low-income households. Agribusiness 20: 309-321.

**Yen 2003**Yen ST, Lin B-H, Smallwood DM (2003) Quasi- and simulated-likelihood approaches to censored demand systems: Food consumption by food stamp recipients in the United States. American Journal of Agricultural Economics 85: 458-478.

**Yen 2002**Yen ST, Lin B-H (2002) Beverage consumption among US children and adolescents: Full-information and quasi maximum-likelihood estimation of a censored system. European Review of Agricultural Economics 29: 85-103.

**Yen 1992**Yen ST, Chern WS (1992) Flexible demand systems with serially correlated errors: Fat and oil consumption in the United States. American Journal of Agricultural Economics 74: 689-697.

**Yip 1993**Yip R, Scanlon K, Trowbridge F (1993) Trends and patterns in height and weight status of low-income U.S. children. Critical Reviews in Food Science and Nutrition 33: 409-421.

**Yoon 2006**Yoon YS, Oh SW, Park HS (2006) Socioeconomic status in relation to obesity and abdominal obesity in Korean adults: A focus on sex differences. Obesity 14: 909-919.

**Yu 2000**Yu Z, Nissinen A, Vartiainen E, Song G, Guo Z, et al. (2000) Associations between socioeconomic status and cardiovascular risk factors in an urban population in China. Bulletin of The World Health Organization 78: 1296-1305.

**Yu 1997**Yu SM, Kogan MD, Gergen P (1997) Vitamin-mineral supplement use among preschool children in the United States. Pediatrics 100: E4.

**Zagorsky 2009**Zagorsky JL, Smith PK (2009) Does the U.S. Food Stamp Program contribute to adult weight gain? Economics & Human Biology 7: 246-258.

**Zeithaml 1985**Zeithaml VA (1985) The new demographics and market fragmentation. Journal of Marketing 49: 64-75.

**Zenk 2005**Zenk SN, Schulz AJ, Hollis-Neely T, Campbell RT, Holmes N, et al. (2005) Fruit and vegetable intake in African Americans: Income and store characteristics. American Journal of Preventive Medicine 29: 1-9.

**Zepeda 2009**Zepeda L (2009) Which little piggy goes to market? Characteristics of US farmers' market shoppers. International Journal of Consumer Studies 33: 250-257.

**Zhang 2004**Zhang Q, Wang Y (2004) Socioeconomic inequality of obesity in the United States: Do gender, age, and ethnicity matter? Social Science & Medicine 58: 1171–1180.

**Zick 2011**Zick CD, Stevens RB (2011) Time spent eating and its implications for Americans' energy balance. Social Indicators Research 101: 267-273.

**Zick 2009**Zick CD, Smith KR, Fan JX, Brown BB, Yamada I, et al. (2009) Running to the Store? The relationship between neighborhood environments and the risk of obesity. Social Science & Medicine 69: 1493-1500.

**Zimmermann 2010**Zimmermann-Sloutskis D, Wanner M, Zimmermann E, Martin BW (2010) Physical activity levels and determinants of change in young adults: A longitudinal panel study. International Journal of Behavioral Nutrition and Physical Activity 7.

**Ziol-Guest 2009**Ziol-Guest KM, Duncan GJ, Kalil A (2009) Early childhood poverty and adult body mass index. American Journal of Public Health 99: 527-532.

**Study reports excluded based on full-text screening**

Adams J, White M (2009) Time perspective in socioeconomic inequalities in smoking and body mass index. Health Psychology 28: 83-90.

Adams RH, Cuecuecha A (2010) Remittances, household expenditure and investment in Guatemala. World Development 38: 1626-1641.

Adams RH (1999) Self-targeted subsidies: The distributional impact of the Egyptian Food Subsidy System. Washington, DC: The World Bank.

Adler N, Singh-Manoux A, Schwartz J, Stewart J, Matthews K, et al. (2008) Social status and health: A comparison of British civil servants in Whitehall-II with European- and African-Americans in CARDIA. Social Science & Medicine 66: 1034-1045.

Alston JM, Sumner DA, Vosti SA (2008) Farm subsidies and obesity in the United States: National evidence and international comparisons. Food Policy 33: 470-479.

Alviola PAIV, Capps O (2010) Household demand analysis of organic and conventional fluid milk in the United States based on the 2004 Nielsen Homescan panel. Agribusiness 26: 369-388.

Amireault S, Godin G, Vohl MC, Perusse L (2008) Moderators of the intention-behaviour and perceived behavioural control-behaviour relationships for leisure-time physical activity. International Journal of Behavioral Nutrition and Physical Activity 5.

Ammerman AS, Lindquist CH, Lohr KN, Hersey J (2002) The efficacy of behavioral interventions to modify dietary fat and fruit and vegetable intake: A review of the evidence. Preventive Medicine: An International Journal Devoted to Practice and Theory 35: 25-41.

Ampofo-Boateng K, Yen M, Barnabas V (2003) The influence of demographic variables on factors that constrain recreational sport participation in Malaysia. Annals of Leisure Research 6: 362-375.

Anderson R, Grossman M (2009) Health and the household. Review of Economics of the Household 7: 219-226.

Angulo AM, Gil JM, Mur J (2007) Spanish demand for food away from home: Analysis of panel data. Journal of Agricultural Economics 58: 289-307.

Angulo AM, Gil JM (2006) Incorporating nutrients into meat demand analysis using household budgets data. Agricultural Economics 35: 131-144.

Anon (2003) Informe sobre la actividad fisica en horas libres y en horas de trabajo en la poblacion estadounidense [Report looks at physical activity in both work and leisure time in the United States]. Revista Panamericana de Salud Publica/Pan American Journal of Public Health 14: 289-292.

Ard JD, Fitzpatrick S, Desmond RA, Sutton BS, Pisu M, et al. (2007) The impact of cost on the availability of fruits and vegetables in the homes of schoolchildren in Birmingham, Alabama. American Journal of Public Health 97: 367-372.

Attanasio O, Lechene V (2010) Conditional cash transfers, women and the demand for food. London, UK: Institute for Fiscal Studies and University College London

Baalwa J, Byarugaba BB, Kabagambe KE, Otim AM (2010) Prevalence of overweight and obesity in young adults in Uganda. African Health Sciences 10: 367-373.

Ball K, Crawford D (2006) Socio-economic factors in obesity: A case of slim chance in a fat world? Asia Pacific Journal of Clinical Nutrition 15 15-20.

Banks E, Jorm L, Rogers K, Clements M, Bauman A (2011) Screen-time, obesity, ageing and disability: Findings from 91 266 participants in the 45 and Up Study. Public Health Nutrition 14: 34-43.

Barros FC, Victora CG, Scherpbier R, Gwatkin D (2010) Socioeconomic inequities in the health and nutrition of children in low/middle income countries. Revista de Saude Publica 44: 1-16.

Bassett DR, Wyatt HR, Thompson H, Peters JC, Hill JO (2010) Pedometer-measured physical activity and health behaviors in U.S. adults. Medicine and Science in Sports and Exercise 42: 1819-1825.

Batten S, Hirschman J, Thomas D (1990) Impact of the special supplemental food program on infants. Journal of Pediatrics 117: s101-s109.

Batty GD, Leon DA (2002) Socio-economic position and coronary heart disease risk factors in children and young people: Evidence from UK epidemiological studies. European Journal of Public Health 12: 263-272.

Baum EL, Corbridge IL (1954) Household income, food expenditures and desires in Seattle, Washington. American Journal of Agricultural Economics 36: 135-139.

Bava CM, Jaeger SR, Park J (2008) Constraints upon food provisioning practices in 'busy' women's lives: Trade-offs which demand convenience. Appetite 50: 486-498.

Beets MW, Cardinal BJ, Alderman BL (2010) Parental social support and the physical activity-related behaviors of youth: A review. Health Education & Behavior 37: 621-644.

Behrman JR, Foster AD, Rosenzweig MR (1997) The dynamics of agricultural production and the calorie-income relationship: Evidence from Pakistan. Journal of Econometrics 77: 187-207.

Behrman JR, Wolfe BL (1984) More evidence on nutrition demand: Income seems overrated and women's schooling underemphasized. Journal of Development Economics 14: 105-128.

Bellisle F, Rolland-Cachera MF, Barthelemy L, Boucher B, Dartois AM, et al. (2007) Three consecutive (1993, 1995, 1997) surveys of food intake, nutritional attitudes and knowledge, and lifestyle in 1000 French children, aged 9-11 years. Journal of Human Nutrition and Dietetics 20: 241-251.

Benge M (1999) How to tax food and make the tax system more progressive at the same time. Agenda 6: 91-94.

Bertoni AG, Clark JM, Feeney P, Yanovski SZ, Bantle J, et al. (2008) Suboptimal control of glycemia, blood pressure, and LDL cholesterol in overweight adults with diabetes: The Look AHEAD Study. Journal of Diabetes & its Complications 22: 1-9.

Besley TJ, Kanbur SMR (1988) Food subsidies and poverty alleviation. Economic Journal 98: 701-719.

Bethell C, Read D, Goodman E, Johnson J, Besl J, et al. (2009) Consistently inconsistent: A snapshot of across- and within-state disparities in the prevalence of childhood overweight and obesity. Pediatrics 123: s277-s286.

Beydoun MA, Wang Y (2008b) How do socio-economic status, perceived economic barriers and nutritional benefits affect quality of dietary intake among US adults? European Journal of Clinical Nutrition 62: 303-313.

Bhattacharya J, Sood N (2005) Health insurance and the obesity externality. Cambridge, MA: National Bureau of Economic Research

Bittman M (2002) Social participation and family welfare: The money and time costs of leisure in Australia. Social Policy and Administration 36: 408-425.

Blaylock J, Smallwood DM, Kassel K, Variyam JN, Aldrich LM (1999) Economics, food choices, and nutrition. Food Policy 24: 269-286.

Boatwright P, Dhar S, Rossi PE (2004) The role of retail competition, demographics and account retail strategy as drivers of promotional sensitivity. Quantitative Marketing and Economics 2: 169-190.

Boylan S, Lallukka T, Lahelma E, Pikhart H, Malyutina S, et al. (2011) Socio-economic circumstances and food habits in Eastern, Central and Western European populations. Public Health Nutrition 14: 678-687.

Brenes H, Mata L (1978) Consumo de alimentos en ninos menores de 5 anos en comunidades rurales de Costa Rica, 1977 [Food consumption of children under 5 in rural communities of Costa Rica, 1977]. Revista de Biologia Tropical 26: 467-483.

Brown DE, Gotshalk LA, Katzmarzyk PT, Allen L (2011) Measures of adiposity in two cohorts of Hawaiian school children. Annals of Human Biology 38: 492-499.

Brownell KD, Farley T, Willett WC, Popkin BM, Chaloupka FJ, et al. (2009) The public health and economic benefits of taxing sugar-sweetened beverages. New England Journal of Medicine 361: 1599-1605.

Cash SB, Sunding D, Ziberman D (2005) Fat taxes and thin subsidies: Prices, diet, and health outcomes. Acta Agriculturae Scandinavica, Section C - Economy 2: 167-174.

Cecil JE, Watt P, Murrie ISL, Wrieden W, Wallis DJ, et al. (2005) Childhood obesity and socioeconomic status: A novel role for height growth limitation. International Journal of Obesity 29: 1199-1203.

Cerin E, Leslie E, Toit Ld, Owen N, Frank LD (2007) Destinations that matter: Associations with walking for transport. Health and Place 13: 713-724.

Ceschini FL, Andrade DR, Oliveira LC, Araujo J, Jorge F, et al. (2009) Prevalence of physical inactivity and associated factors among high school students from state's public schools. Jornal de Pediatria 85: 301-306.

Chen Y, Factor-Litvak P, Howe GR, Parvez F, Ahsan H (2006) Nutritional influence on risk of high blood pressure in Bangladesh: A population-based cross-sectional study. American Journal of Clinical Nutrition 84: 1224-1232.

Chung C-F, Lopez E (1988) A regional analysis of food consumption in Spain. Economics Letters 26: 209-213.

Connolly-Schoonen J (2007) Food availability and pediatric overweight: Fields of influence and bounded rationality [Doctoral dissertation]. Stony Brook, NY, United States: Stony Brook University.

Costanzi CB, Halpern R, Rech RR, Bergmann MLDA, Alli LR, et al. (2009) Associated factors in high blood pressure among schoolchildren in a middle size city, southern Brazil. Jornal de Pediatria 85: 335-340.

Cubbin C, Sundquist K, Ahlen H, Johansson S-E, Winkleby MA, et al. (2006) Neighborhood deprivation and cardiovascular disease risk factors: Protective and harmful effects. Scandinavian Journal of Public Health 34: 228-237.

Cutler D, Glaeser E, Shapiro J (2003) Why have Americans become more obese? Cambridge, MA: National Bureau of Economic Research.

Dargay J (2007) The effect of prices and income on car travel in the UK. Transportation Research: Part A: Policy and Practice 41: 949-960.

Darling H, Reeder AI, McGee R, Williams S (2006) Brief report: Disposable income, and spending on fast food, alcohol, cigarettes, and gambling by New Zealand secondary school students. Journal of Adolescence 29: 837-843.

Dekimpe MG, Hanssens DM, Silva-Risso JM (1999) Long-run effects of price promotions in scanner markets. Journal of Econometrics 89: 269-291.

del Ninno C, Dorosh PA (2003) Impacts of in-kind transfers on household food consumption: Evidence from targeted food programmes in Bangladesh. Journal of Development Studies 40: 48-78.

Delva J, Johnston LD, O'Malley PM (2007) The epidemiology of overweight and related lifestyle behaviors: Racial/ethnic and socioeconomic status differences among American youth. American Journal of Preventive Medicine 33: s178-s186.

Dhehibi B, Laajimi A (2009) Effects of food prices and consumer income on nutrient availability: An application of the demand for dairy products in Tunisia. Economia Agraria y Recursos Naturales 9: 25-36.

Dhehibi B, Laajimi A (2004) How economic factors influence the nutrient content of diets: An application of animal products demand system in Tunisia. Agricultural Economics Review 5: 67-79.

Dimenstein R, Simplicio JL, Ribeiro KDS, Melo ILP (2003) Influencia de variaveis socioeconomicas e de saude materno-infantil sobre os niveis de retinol no colostro humano [Retinol levels in human colostrum: Influence of child, maternal and socioeconomic variables]. Jornal de Pediatria 79: 513-518.

Doljanin K, Olaris K (2004) Subsidised cafe meals program: More than "just a cheap meal". Australian Journal of Primary Health 10: 54-60.

Drewett R, Emond A, Blair P, Emmett P (2005) The importance of slow weight gain in the first 2 months in identifying children who fail to thrive. Journal of Reproductive and Infant Psychology 23: 309-317.

Drewnowski A, Bellisle F (2007) Liquid calories, sugar, and body weight. American Journal of Clinical Nutrition 85: 651-661.

Dumith SC (2009) Physical activity in Brazil: A systematic review. Cadernos de Saude Publica 25: s415-s426.

Fabiosa JF (2006) Westernization of the Asian diet: The case of rising wheat consumption in Indonesia. Ames, IA: Center for Agricultural and Rural Development, Iowa State University.

Ferrand A, Robinson L, Valette-Florence P (2010) The intention-to-repurchase paradox: A case of the health and fitness industry. Journal of Sport Management 24: 83-105.

Fox MK, Dodd AH, Wilson A, Gleason PM (2009) Association between school food environment and practices and body mass index of US public school children. Journal of the American Dietetic Association 109: s108-s117.

French SA, Story M, Fulkerson JA, Hannan P (2004a) An environmental intervention to promote lower-fat food choices in secondary schools: Outcomes of the TACOS study. American Journal of Public Health 94: 1507-1512.

Frenn M, Malin S, Villarruel AM, Slaikeu K, McCarthy S, et al. (2005) Determinants of physical activity and low-fat diet among low income African American and Hispanic middle school students. Public Health Nursing 22: 89-97.

Fulkerson JA, French SA, Story M, Nelson H, Hannan PJ (2004) Promotions to increase lower-fat food choices among students in secondary schools: description and outcomes of TACOS (Trying Alternative Cafeteria Options in Schools). Public Health Nutrition 7: 665-674.

Gagnon L (2003) "Sin" tax proposed for junk food. Canadian Medical Association Journal 168: 1697-1697.

Galdeano E (2005) An inverse demand analysis with introduction of quality effects: An application to Spanish consumption of fruit and vegetables. Agricultural Economics 33: 163-177.

Gandal N, Shabelansky A (2009) Obesity and price sensitivity at the supermarket. London, UK: Centre for Economic Policy Research.

Gittelsohn J, Vijayadeva V, Davison N, Ramirez V, Cheung LWK, et al. (2010) A food store intervention trial improves caregiver psychosocial factors and children's dietary intake in Hawaii. Obesity 18: s84-s90.

Gourley L, Duffy SW, Lee HP, Walker AM, Day NE (1988) A survey of household food purchases and dietary habits in relation to affluence among Singapore Chinese. European Journal of Clinical Nutrition 42: 333-343.

Grier SA, Kumanyika SK (2008) The context for choice: Health implications of targeted food and beverage marketing to African Americans. American Journal of Public Health 98: 1616-1629.

Griffith R, O'Connell M (2009b) The use of scanner data for research into nutrition. Fiscal Studies 30: 339-365.

Gulliford MC, Mahabir D, Rocke B (2003) Food insecurity, food choices, and body mass index in adults: Nutrition transition in Trinidad and Tobago. International Journal of Epidemiology 32: 508-516.

Hamilton S, Ni Mhurchu C, Priest P (2007) Food and nutrient availability in New Zealand: An analysis of supermarket sales data. Public Health Nutrition 10: 1448-1455.

Harrell JS, Gansky SA, Bradley CB, McMurray RG (1997) Leisure time activities of elementary school children. Nursing Research 46: 246-253.

Hassan ZA, Johnson SR, Finley RM (1977) An intertemporal comparison of price and income elasticities for food: Reply. Canadian Journal of Agricultural Economics 25: 104-105.

Horswill LJ, Yap C (1999) Consumption of foods from the WIC food packages of Chinese prenatal patients on the US west coast. Journal of the American Dietetic Association 99: 1549-1553.

Houston S, Mitchell S, Evans S (2011) Prevalence of cardiovascular disease risk factors among UK commercial pilots. European Journal of Cardiovascular Prevention and Rehabilitation 18: 510-517.

Ikeda S, Kang M-I, Ohtake F (2010) Hyperbolic discounting, the Sign Effect, and the body mass index. Journal of Health Economics 29: 268-284.

Ikeda S (2009) Time discounting and obesity. The Japanese Journal of Psychonomic Science 28: 156-159.

Jacoby E, Goldstein J, Lopez A, Nunez E, Lopez T (2003) Social class, family, and life-style factors associated with overweight and obesity among adults in Peruvian cities. Preventive Medicine 37: 396-405.

Jaeger SR, Meiselman HL (2004) Perceptions of meal convenience: The case of at-home evening meals. Appetite 42: 317-325.

Jaime PC, Monteiro CA (2005) Fruit and vegetable intake by Brazilian adults, 2003. Cadernos de saude publica / Ministerio da Saude, Fundacao Oswaldo Cruz, Escola Nacional de Saude Publica 21 s19-s24.

Johnson RF (1987) Adult leisure participation and preference differentials at denominationally sponsored retreat/assembly areas [Thesis]. Athens, GA, United States: University of Georgia.

Just DR, Wansink B (2011) The flat-rate pricing paradox: Conflicting effects of 'all-you-can-eat' buffet pricing. Review of Economics and Statistics 93: 193-200.

Karp R, Fairorth J, Kanofsky P, Matthews W, Nelson M, et al. (1978) Effects of rise in food costs on hemoglobin concentrations of early school-age children, 1972-75. Public Health Reports 93: 456-459.

Katsarou A, Tyrovolas S, Psaltopoulou T, Zeimbekis A, Tsakountakis N, et al. (2010) Socio-economic status, place of residence and dietary habits among the elderly: The Mediterranean islands study. Public Health Nutrition 13: 1614-1621.

Katz MH, Bhatia R (2010) Food surcharges and subsidies: Putting your money where your mouth is. Archives of Internal Medicine 170: 405-406.

Kaur S, Sachdev HPS, Dwivedi SN, Lakshmy R, Kapil U (2008) Prevalence of overweight and obesity amongst school children in Delhi, India. Asia Pacific Journal of Clinical Nutrition 17: 592-596.

Kaushal N (2007) Do food stamps cause obesity?: Evidence from immigrant experience. Journal of Health Economics 26: 968-991.

Kehoe TJ, Serra-Puche J (1986) A general equilibrium analysis of price controls and subsidies on food in Mexico. Journal of Development Economics 21: 65-87.

Kelly IR, Markowitz S (2009) Incentives in obesity and health insurance. Inquiry: A journal of medical care organization, provision and financing 46: 418-432.

Kennedy E, Bouis H, Von B (1992) Health and nutrition effects of cash crop production in developing countries: A comparative analysis. Social Science and Medicine 35: 689-697.

Kim S, Symons M, Popkin BM (2004) Contrasting socioeconomic profiles related to healthier lifestyles in China and the United States. American Journal of Epidemiology 159: 184-191.

Kinsey JD (1997) Income and food consumption: A variety of answers: Discussion. American Journal of Agricultural Economics 79: 1461-1464.

Kirkpatrick SI, Tarasuk V (2010) Assessing the relevance of neighbourhood characteristics to the household food security of low-income Toronto families. Public Health Nutrition 13: 1139-1148.

Kitamura R (2009) Life-style and travel demand (1988). Transportation 36: 679-710.

Klonoff DC (2009) A sweetened beverage tax is needed to combat the obesity epidemicas well as related absenteeism and presenteeism. Journal of Diabetes Science & Technology 3: 408-410.

Kockelman KM (2001) A model for time- and budget-constrained activity demand analysis. Transportation Research: Part B: Methodological 35: 255-269.

Kohls RL (1955) The place of merchandising and promotion in expanding the demand for food [with discussion by R. M. Walsh]. Journal of Farm Economics 37: 1380-1386.

Kuczmarski RJ (1992) Prevalence of overweight and weight gain in the United States. American Journal of Clinical Nutrition 55: s495-s502.

Kvamme J-M, Wilsgaard T, Florholmen J, Jacobsen BK (2010) Body mass index and disease burden in elderly men and women: The Tromso Study. European Journal of Epidemiology 25: 183-193.

LaFrance JT (2002) Information theoretic measures of the income distribution in food demand. Journal of Econometrics 107: 235-257.

Lagiou P, Trichopoulou A, Data Food Networking Data contributors (2001) The DAFNE initiative: The methodology for assessing dietary patterns across Europe using household budget survey data. Public Health Nutrition 4: 1135-1141.

Lahelma E, Sarlio-Lahteenkorva S, Silventoinen K (2004) Relative weight and income at different levels of socioeconomic status. American Journal of Public Health 94: 468-472.

Lane S (1978) Food distribution and Food Stamp Program effects on food consumption and nutritional 'achievement' of low income persons in Kern County, California. American Journal of Agricultural Economics 60: 108-116.

Lee JB, MacKey-Bilaver L (2007) Effects of WIC and Food Stamp Program participation on child outcomes. Children and Youth Services Review 29: 501-517.

Lee K, Song Y-M (2007) Parent-reported appetite of a child and the child's weight status over a 2-year period in Korean children. Journal of the American Dietetic Association 107: 678-680.

Liaskos G, Lazaridis P (2003) The Demand for Selected Food Nutrients in Greece: The Role of Socioeconomic Factors. Agricultural Economics Review 4: 93-106.

Lichtenstein DR, Bloch PH, Black WC (1988) Correlates of price acceptability. Journal of Consumer Research 15: 243-252.

Lindstom M, Hanson BS, Wirfalt E, Ostergren P-O (2001) Socioeconomic differences in the consumption of vegetables, fruit and fruit juices. European Journal of Public Health 11: 51-59.

Liu L, Rettenmaier AJ, Saving TR (2007b) Endogenous food quality and bodyweight trend. Advances in Health Economics & Health Services Research 17: 3-21.

Luoto R, Pekkanen J, Uutela A, Tuomilehto J (1994) Cardiovascular risks and socioeconomic status: Differences between men and women in Finland. Journal of Epidemiology and Community Health 48: 348-354.

Mackenbach JP, Stirbu I, Roskam A-JR, Schaap MM, Menvielle G, et al. (2008) Socioeconomic inequalities in health in 22 European countries. New England Journal of Medicine 358: 2468-2481.

Maddah M, Nikooyeh B (2010) Obesity among Iranian adolescent girls: Location of residence and parental obesity. Journal of Health, Population & Nutrition 28: 61-66.

Manuck SB, Phillips JE, Gianaros PJ, Flory JD, Muldoon MF (2010) Subjective socioeconomic status and presence of the metabolic syndrome in midlife community volunteers. Psychosomatic Medicine 72: 35-45.

Marette S, Roosen J, Blanchemanche S (2007) Tax, subsidy, and/or information for health: An example from fish consumption. Ames, IA: Iowa State University.

Marette S, Roosen J, Blanchemanche S (2008) Taxes and subsidies to change eating habits when information is not enough: An application to fish consumption. Journal of Regulatory Economics 34: 119-143.

Marinda PA (2006) Child-mother nutrition and health status in rural Kenya: The role of intra-household resource allocation and education. International Journal of Consumer Studies 30: 327-336.

Marins VMR, Almeida RMVR, Pereira RA, Sichieri R (2007) The association between socioeconomic indicators and cardiovascular disease risk factors in Rio de Janeiro, Brazil. Journal of Biosocial Science 39: 221-229.

Marshall T (2000) Exploring a fiscal food policy: The case of diet and ischaemic heart disease. British Medical Journal 320: 301-304.

Martinez-Garmendia J (2010) Application of hedonic price modeling to consumer packaged goods using store scanner data. Journal of Business Research 63: 690-696.

Mbuya MNN, Habicht J-P, Monteiro CA (2005) Revisiting the independent effects of income on the risk of obesity. Journal of Nutrition 135: 2496.

McGrail KM, van Doorslaer E, Ross NA, Sanmartin C (2009) Income-related health inequalities in Canada and the United States: A decomposition analysis. American Journal of Public Health 99: 1856-1863.

Merlo J, Chaix B, Yang M, Lynch J, Rastam L (2005) A brief conceptual tutorial on multilevel analysis in social epidemiology: Interpreting neighbourhood differences and the effect of neighbourhood characteristics on individual health. Journal of Epidemiology and Community Health 59: 1022-1028.

Miller JE, Korenman S (1994) Poverty and children's nutritional status in the United States. American Journal of Epidemiology 140: 233-243.

Monsivais P, Kirkpatrick S, Johnson DB (2011) More nutritious food is served in child-care homes receiving higher federal food subsidies. Journal of the American Dietetic Association 111: 721-726.

Morgenstern M, Sargent JD, Hanewinkel R (2009) Relation between socioeconomic status and body mass index: Evidence of an indirect path via television use. Archives of Pediatrics & Adolescent Medicine 163: 731-738.

Murakami K, Sasaki S, Okubo H, Takahashi Y (2009) Neighborhood socioeconomic status in relation to dietary intake and body mass index in female Japanese dietetic students. Nutrition 25: 745-752.

Murakami K, Sasaki S, Takahashi Y, Uenishi K, Japan Dietetic Students' Study For Nutrition, et al. (2009) Neighborhood socioeconomic disadvantage is associated with higher ratio of 24-hour urinary sodium to potassium in young Japanese women. Journal of the American Dietetic Association 109: 1606-1611.

Naska A, Bountziouka V, Trichopoulou A, Participants D (2010) Soft drinks: Time trends and correlates in twenty-four European countries. A cross-national study using the DAFNE (Data Food Networking) databank. Public Health Nutrition 13: 1346-1355.

Nelson JA (1991) Quality variation and quantity aggregation in consumer demand for food. American Journal of Agricultural Economics 73: 1204-1212.

Nelson JP (2001) Hard at play! The growth of recreation in consumer budgets, 1959-1998. Eastern Economic Journal 27: 35-53.

Newman C, Henchion M, Matthews A (2003) A double-hurdle model of Irish household expenditure on prepared meals. Applied Economics 35: 1053-1061.

Nguyen MD, Beresford SAA, Drewnowski A (2007) Trends in overweight by socio-economic status in Vietnam: 1992 to 2002. Public Health Nutrition 10: 115-121.

Ni Mhurchu C, Blakely T, Wall J, Rodgers A, Jiang Y, et al. (2007) Strategies to promote healthier food purchases: A pilot supermarket intervention study. Public Health Nutrition 10: 608-615.

Niclasen B, Schnohr CW (2010) Greenlandic schoolchildren's compliance with national dietary guidelines. Public Health Nutrition 13: 1162-1169.

Nugent R (2004) Food and agriculture policy: Issues related to prevention of noncommunicable diseases. Food & Nutrition Bulletin 25: 200-207.

O&P Business News (2005) Low income children not fated to be overweight. O&P Business News 14: 68-68.

Olinto MT, Willett WC, Gigante DP, Victora CG (2011) Sociodemographic and lifestyle characteristics in relation to dietary patterns among young Brazilian adults. Public Health Nutrition 14: 150-159.

Olson CM, Bove CF, Miller EO (2007) Growing up poor: Long-term implications for eating patterns and body weight. Appetite 49: 198-207.

Panagiotakos DB, Pitsavos C, Manios Y, Polychronopoulos E, Chrysohoou CA, et al. (2005) Socio-economic status in relation to risk factors associated with cardiovascular disease, in healthy individuals from the ATTICA study. European Journal of Cardiovascular Prevention & Rehabilitation 12: 68-74.

Park Y-H, de Groot L, C. P. G. M., van Staveren WA (2003) Dietary intake and anthropometry of Korean elderly people: A literature review. Asia Pacific Journal of Clinical Nutrition 12: 234-242.

Parsons TJ, Manor O, Power C (2005) Changes in diet and physical activity in the 1990s in a large British sample (1958 birth cohort). European Journal of Clinical Nutrition 59: 49-56.

Patterson PM, Olofsson H, Richards TJ, Sass S (1999) An empirical analysis of state agricultural product promotions: A case study on Arizona grown. Agribusiness 15: 179-196.

Pattillo R (2010) Is tax on soft drinks in our future? Nurse Educator 35: 1-9.

Philipson TJ, Posner RA (1999) The long-run growth in obesity as a function of technological change. Cambridge, MA: National Bureau of Economic Research.

Plotnikoff RC, Hugo K, Cousineau N (2001) Heart disease risk factor prevalence and profiles in a randomized community sample of Canadian women. Canadian Journal of Public Health Revue Canadienne de Sante Publique 92: 121-126.

Pratt M, Macera CA, Sallis JF, O'Donnell M, Frank LD (2004) Economic interventions to promote physical activity: Application of the SLOTH model. American Journal of Preventive Medicine 27: 136-145.

Raju JS (1992) The effect of price promotions on variability in product category sales. Marketing Science 11: 207-220.

Ramachandran A, Snehalatha C, Vinitha R, Thayyil M, Kumar CKS, et al. (2002) Prevalence of overweight in urban Indian adolescent school children. Diabetes Research & Clinical Practice 57: 185-190.

Rehm J, Sempos C, Kohlmeier L, Myers G, Thefeld W, et al. (2000) A comparison of serum total cholesterol levels and their determinants between the Federal Republic of Germany and the United States. European Journal of Epidemiology 16: 669-675.

Reynolds A (1990) Analyzing fresh vegetable consumption from household survey data. Southern Journal of Agricultural Economics 22: 31-38.

Robertson A, Tirado C, Lobstein T, Jermini M, Knai C, et al. (2004) Food and health in Europe: A new basis for action. World Health Organization Regional Publications European Series.: i-xvi, 1.

Rose D (2011) Growing our kids in "healthy soil": New research on environmental influences on children's food intake. Journal of Adolescent Health 48: 3-4.

Ruhm CJ (2010) Understanding Overeating and Obesity. Cambridge, MA: National Bureau of Economic Research.

Salehi L, Eftekhar H, Mohammad K, Tavafian SS, Jazayery A, et al. (2010) Consumption of fruit and vegetables among elderly people: A cross sectional study from Iran. Nutrition Journal 9: 2.

Samani-Radia D, McCarthy HD (2011) Comparison of children's body fatness between two contrasting income groups: Contribution of height difference. International Journal of Obesity 35: 128-133.

Sanwalka NJ, Khadilkar AV, Mughal MZ, Sayyad MG, Khadilkar VV, et al. (2010) A study of calcium intake and sources of calcium in adolescent boys and girls from two socioeconomic strata, in Pune, India. Asia Pacific Journal of Clinical Nutrition 19: 324-329.

Sarlio-Lahteenkorva S, Lahelma E (1999) The association of body mass index with social and economic disadvantage in women and men. International Journal of Epidemiology 28: 445-449.

Schneider RR (1985) Food subsidies: A multiple price model. International Monetary Fund Staff Papers 32: 289-316.

Selmer RM, Kristiansen IS, Haglerod A, Graff-Iversen S, Larsen HK, et al. (2000) Cost and health consequences of reducing the population intake of salt. Journal of Epidemiology & Community Health 54: 697-702.

Serra-Majem L, Ribas L, Ngo J, Ortega RM, Garcia A, et al. (2004) Food, youth and the Mediterranean diet in Spain: Development of KIDMED, Mediterranean Diet Quality Index in children and adolescents. Public Health Nutrition 7: 931-935.

Seymour JD, Ann F, Lazarus Y, Kettel K, Serdula M (2004a) Fruit and vegetable environment, policy, and pricing workshop: Introduction to the conference proceedings. Preventive Medicine 39: s71-s74.

Shape (2006) Uncle Sam wants you to work out. Shape 26: 107-107.

Sharkey JR, Schoenberg NE (2002) Variations in nutritional risk among black and white women who receive home-delivered meals. Journal of Women & Aging 14: 99-119.

Shelton NJ (2005) What not to eat: Inequalities in healthy eating behaviour, evidence from the 1998 Scottish Health Survey. Journal of Public Health 27: 36-44.

Sherwood NE, Jeffery RW (2000) The behavioral determinants of exercise: Implications for physical activity interventions. Annual Review of Nutrition 20: 21-44.

Sichieri R, Silva CVC, Moura AS (2003) Combined effect of short stature and socioeconomic status on body mass index and weight gain during reproductive age in Brazilian women. Brazilian Journal of Medical and Biological Research 36: 1319-1325.

Singh RB, Beegom R, Mehta AS, Niaz MA, De A, et al. (1999) Social class, coronary risk factors and undernutrition, a double burden of diseases, in women during transition, in five Indian cities. International Journal of Cardiology 69: 139-147.

Singh RB, Sharma JP, Rastogi V, Niaz MA, Singh NK (1997) Prevalence and determinants of hypertension in the Indian social class and heart survey. Journal of Human Hypertension 11: 51-56.

Soori H (2001) Pattern of dietary behaviour and obesity in Ahwaz, Islamic Republic of Iran. Eastern Mediterranean Health Journal 7: 163-170.

Sugarman SD, Sandman N (2008) Using performance-based regulation to reduce childhood obesity. Australia and New Zealand Health Policy 5.

Tarasuk V, McIntyre L, Li J (2007) Low-income women's dietary intakes are sensitive to the depletion of household resources in one month. Journal of Nutrition 137: 1980-1987.

Thow AM (2009) Trade liberalisation and the nutrition transition: Mapping the pathways for public health nutritionists. Public Health Nutrition 12: 2150-2158.

Turrell G, Blakely T, Patterson C, Oldenburg B (2004) A multilevel analysis of socioeconomic (small area) differences in household food purchasing behaviour. Journal of Epidemiology and Community Health 58: 208-215.

Turrell G, Haynes M, Burton NW, Giles-Corti B, Oldenburg B, et al. (2010) Neighborhood disadvantage and physical activity: Baseline results from the HABITAT multilevel longitudinal study. Annals of Epidemiology 20: 171-181.

Ulijaszek SJ (2003) Socio-economic factors associated with physique of adults of the Purari Delta of the Gulf Province, Papua New Guinea. Annals of Human Biology 30: 316-328.

Ulijaszek SJ, Koziel S, Hermanussen M (2005) Village distance from urban centre as the prime modernization variable in differences in blood pressure and body mass index of adults of the Purari |Delta of the Gulf Province, Papua New Guinea. Annals of Human Biology 32: 326-338.

Utter J, Scragg R, Ni Mhurchu C, Schaaf D (2007) At-home breakfast consumption among New Zealand children: Associations with body mass index and related nutrition behaviors. Journal of the American Dietetic Association 107: 570-576.

Van Zyl MK, Steyn NP, Marais ML (2010) Characteristics and factors influencing fast food intake of young adult consumers in Johannesburg, South Africa. South African Journal of Clinical Nutrition 23: 124-130.

Wang MC, Kim S, Gonzalez AA, MacLeod KE, Winkleby MA (2007) Socioeconomic and food-related physical characteristics of the neighborhood environment are associated with body mass index. Journal of Epidemiology and Community Health 61: 491-498.

Wessman C, Betterley C, Jensen H (2001) An evaluation of the costs and benefits of Iowa's Expanded Food and Nutrition Education Program (EFNEP). Final Report Ames, IA, United States: Center for Agricultural and Rural Development, Iowa State University.

West DA, Price DW (1976) The effects of income, assets, food programs, and household size on food consumption. American Journal of Agricultural Economics 58: 725-730.

Weststrate JA, van het Hof KH, van den Berg H, Velthuis-te-Wierik EJ, de Graaf C, et al. (1998) A comparison of the effect of free access to reduced fat products or their full fat equivalents on food intake, body weight, blood lipids and fat-soluble antioxidants levels and haemostasis variables. European Journal of Clinical Nutrition 52: 389-395.

Yaniv G, Rosin O, Tobol Y (2009) Junk-food, home cooking, physical activity and obesity: The effect of the fat tax and the thin subsidy. Journal of Public Economics 93: 823-830.

Yarcheski A, Mahon NE, Yarcheski TJ, Cannella BL (2004) A meta-analysis of predictors of positive health practices. Journal of Nursing Scholarship 36: 102-108.

Yen IH (1997) The influence of neighborhood socioenvironmental characteristics on health behaviors and health status: Evidence from the Alameda County study [Doctoral dissertation]. Berkeley, CA, United States: University California at Berkeley.

Yen IH, Kaplan GA (1998) Poverty area residence and changes in physical activity level: Evidence from the Alameda County Study. American Journal of Public Health 88: 1709-1712.

Yitzhaki S (1990) On the effect of subsidies to basic food commodities in Egypt. Oxford Economic Papers, N S 42: 772-792.

Zhang L, Rashad I (2008) Obesity and time preference: The health consequences of discounting the future. Journal of Biosocial Science 40: 97-113.

**Study reports for which the full-text could not be assessed**

Abdus S (2007) Obesity: The role of economic incentive in an individual's body weight choice [Doctoral dissertation]. Minneapolis, MN, United States: University of Minnesota.

Adeniyi AF, Chedi H (2010) Levels and predictors of physical activity in a sample of pre-retirement and retired civil servants in Nigeria. East African Journal of Public Health 7: 140-143.

Agbola FW (2005) Estimating demand elasticities with the Linear Approximate Almost Ideal Demand System (LA/AIDS): Some empirical evidence from India. Empirical Economics Letters 4: 1-12.

Ahmed AU, Bouis HE, Gutner T, Lofgren H (2002) The Egyptian food subsidy system: Structure, performance, and options for reform. Food & Nutrition Bulletin 23: 423-424.

Ahmed AU, Gutner T, Lofgren H, Bouis HE (2001) The Egyptian food subsidy system: Structure, performance, and options for reform. Washington, D.C: International Food Policy Research Institute.

Akpan JO, Gingerich RL (1991) Association of hyperglycemia with dietary cyanogen and socio-economic level: The study of rural communities in south-east Nigeria. Acta Diabetologica Latina 28: 29-37.

Akpinar MG, Ozkan B, Sayin C, Ceylan RF (2010) Consumer risk perceptions towards food supply chain preferences: The case of the supermarket. Journal of Food, Agriculture and Environment 8: 256-260.

Akpinar MG, Aykin SM, Sayin C, Ozkan B (2009) The role of demographic variables in purchasing decisions on fresh fruit and vegetables. Journal of Food, Agriculture and Environment 7: 106-110.

Alarcon JA, Immink MD (1990) Elasticidad ingreso de la demanda de alimentos y otros bienes en grupos de poblacion marginal urbana de la ciudad de Guatemala [Stretching the income in the demand for food and other goods in marginal urban populations in the city of Guatemala]. Archivos Latinoamericanos de Nutricion 40: 518-532.

al-Awadi F, Amine EK (1989) Overweight and obesity in Kuwait. Journal of the Royal Society of Health 109: 175-177.

Albu A, Cucu AI, Malaimare A, Indrei LL (2010) Relatia venitul familiei--alimentatie la un lot de prescolari din orasul Negresti judetul vaslui [Family income--nutrition correlation in a group of Negresti Vaslui preschool children]. Revista medico-chirurgicala a Societatii de Medici si Naturalisti din Iasi 114: 841-844.

Alderman H (1988) Food subsidies in Egypt: Benefit distribution and nutritional effects. In: Pinstrup-Andersen P, editor. Food subsidies in developing countries: Costs, benefits, and policy options. Baltimore and London: Johns Hopkins University Press for the International Food Policy Research Institute. pp. 171-182.

al-Isa AN (1999a) Obesity among Kuwait University students: An explorative study. Journal of the Royal Society for the Promotion of Health 119: 223-227.

al-Isa AN, Moussa MA (1999b) Factors associated with overweight and obesity among Kuwaiti kindergarten children aged 3-5 years. Nutrition and health (Berkhamsted, Hertfordshire) 13: 125-139.

al-Isa AN (1998) Factors associated with overweight and obesity among Kuwaiti college women. Nutrition and health (Berkhamsted, Hertfordshire) 12: 227-233.

Al-Mannai A, Dickerson JWT, Morgan JB, Khalfan H (1996) Obesity in Bahraini adults. Journal of the Royal Society of Health 116: 30-32,37.

Aloia CR, Gasevic D, Yusuf S, Teo K, Chockalingam A, et al. (2011) Differences in fast food consumption between individuals of high and low socio-economic status in Chandigarh, India. Canadian Journal of Diabetes 35 156.

Alvarez CF, Diaz MJJ, Riano G, Perez S, Venta O, et al. (2011) Factores de riesgo cardiovascular clasicos y emergentes en escolares asturianos [Classic and emergent cardiovascular risk factors in schoolchildren in Asturias]. Anales de Pediatria 74: 388-395.

Ambrosius WT, Newman SA, Pratt JH (2001) Rates of change in measures of body size vary by ethnicity and gender. Ethnicity & Disease 11: 303-310.

Angulo AM, Mtimet N, Gil JM (2008) Analisis de la demanda de alimentos en Espana considerando el impacto de la dieta sobre la salud [Spanish food demand analysis taking into account the impact of diet on health. With English summary]. Economia Agraria y Recursos Naturales 8: 3-30.

Antonio MA, Ribeiro JD, Toro AA, Piedrabuena AE, Morcillo AM (2003) Avaliacao do estado nutricional de criancas e adolescentes com asma [Evaluation of the nutritional status of children and adolescents with asthma]. Revista da Associacao Medica Brasileira 49: 367-371.

Arabi M (2010) Linking tortilla price policies to household food consumption and child nutritional intake: Potential outcomes of globalization in rural Mexico [Doctoral dissertation]. Ithica, NY, United States: Cornell University.

Aro A (2003) Laskiverolla ja sensuurillako lihavuuden kimppuun? [Should obesity be attacked by tax for overweight and censure?]. Duodecim: Laaketieteellinen aikakauskirja 119: 1202-1203.

Arroyo P, Mendez O (2007) [Energetic density, diversity of diets and familial income in rural and urban households of Mexico]. Gaceta Medica de Mexico 143: 301-307.

Asfaw A (2006) The role of food price policy in determining the prevalence of obesity: Evidence from Egypt. Review of Agricultural Economics 28: 305-312.

Ashe MC, Miller WC, Eng JJ, Noreau L, Physical Activity and Chronic Conditions Research Team (2009) Older adults, chronic disease and leisure-time physical activity. Gerontology 55: 64-72.

Assarsson B, Edgerton D (1996) The consumption of food in the Nordic countries. In: Edgerton DL, Assarsson B, Hummelmose A, Laurila IP, Rickertsen K et al., editors. The econometrics of demand systems: With applications to food demand in the Nordic countries. Dordrecht, Boston and London: Kluwer Academic Publishers. pp. 7-53.

Bacchieri G, Gigante DP, Assuncao MC (2005) Determinantes e padrões de utilização da bicicleta e acidentes de trânsito sofridos por ciclistas trabalhadores da cidade de Pelotas, Rio Grande do Sul, Brasil [Determinants and patterns of bicycle use and traffic accidents among bicycling workers in Pelotas, Rio Grande do Sul, Brazil]. Cadernos de Saude Publica 21: 1499-1508.

Bahl R, Bird R, Walker MB (2003) The uneasy case against discriminatory excise taxation: Soft drink taxes in Ireland. Public Finance Review 31: 510-533.

Baretta E, Baretta M, Peres KG (2007) Nivel de atividade fisica e fatores associados em adultos no Municipio de Joacaba, Santa Catarina, Brasil [Physical activity and associated factors among adults in Joacaba, Santa Catarina, Brazil]. Cadernos de Saude Publica 23: 1595-1602.

Barrett JE, Plotnikoff RC, Courneya KS, Raine KD (2007) Physical activity and type 2 diabetes: Exploring the role of gender and income. The Diabetes Educator 33: 128-143.

Barretto SA, Cyrillo DC (2001) Análise da composição dos gastos com alimentação no Município de São Paulo (Brasil) na década de 1990 [Analysis of household expenditures with food in the city of S. Paulo in the 1990's]. Revista de Saude Publica 35: 52-59.

Bauman A, Owen N, Rushworth RL (1990) Recent trends and socio-demographic determinants of exercise participation in Australia. Community Health Studies 14: 19-26.

Benedetti TR, Goncalves LHT, Petroski EL, Nassar SM, Schwingel A, et al. (2008) Aging in Brazil: Physical activity, socioeconomic conditions and diseases among older adults in Southern Brazil. Journal of Applied Gerontology 27: 631-640.

Bergstrom JC, Cordell HK (1991) An analysis of the demand for and value of outdoor recreation in the United States. Journal of Leisure Research 23: 67-86.

Bhargava A (2006a) Estimating short and long run income elasticities of foods and nutrients for rural south India. In: Bhargava A, editor. Econometrics, statistics and computational approaches in food and health sciences. Hackensack, N.J. and Singapore: World Scientific. pp. 81-98.

Bhargava A (2006b) Socio-economic and behavioural factors are predictors of food use in the National Food Stamp Program Survey. In: Bhargava A, editor. Econometrics, Statistics and Computational Approaches in Food and Health Sciences. Hackensack, N.J. and Singapore: World Scientific. pp. 363-372.

Blaine TW, Mohammad G (1991) An empirical assessment of U.S. consumer expenditures for recreation-related goods and services: 1946-1988. Leisure Sciences 13: 111-122.

Boizot-Szantai C, Etile F (2009) Le prix des aliments et la distribution de l'indice de masse corporelle des Francais [Food prices and the distribution of body mass index in France]. Revue Economique 60: 413-440.

Bowley AL (1945) Family income and expenditure on food. Proceedings of the Nutrition Society 3: 32-39.

Brail RK, Chapin F (1973) Activity patterns of urban residents. Environment and Behavior 5: 163-190.

Bridges E, Briesch RA (2006) The 'nag factor' and children's product categories. International Journal of Advertising: The Quarterly Review of Marketing Communications 25: 157-187.

Brooks C (1988) A causal modeling analysis of sociodemographics and moderate to vigorous physical activity behavior of American adults. Research quarterly for exercise and sport 59: 328-338.

Brown A, Siahpush M (2006) Socioeconomic predictors of a sedentary lifestyle: Results from the 2001 National Health Survey. Journal of Physical Activity & Health 3: 90-101.

Brown DR, Yore MM, Ham SA, Macera CA (2005) Physical activity among adults > or = 50 yr with and without disabilities, BRFSS 2001. Medicine and Science in Sports and Exercise 37: 620-629.

Browning C, Sims J, Kendig H, Teshuva K (2009) Predictors of physical activity behavior in older community-dwelling adults. Journal of Allied Health 38: 8-17.

Bryan SN, Katzmarzyk PT (2009) Are Canadians meeting the guidelines for moderate and vigorous leisure-time physical activity? Applied Physiology, Nutrition, & Metabolism- Physiologie Appliquee, Nutrition et Metabolisme 34: 707-715.

Burk MC (1994) A study of recent relationships between income and food expenditures. Journal of Agricultural Economics Research 45: 21-31.

Butler JS, Raymond JE (1996) The effect of the Food Stamp Program on nutrient intake. Economic Inquiry 34: 781-798.

Campos LDA, Leite AJM, De Almeida PC (2006a) Nivel socioeconomico e sua influencia sobre a prevalencia de sobrepeso e obesidade em escolares adolescentes do municipio de Fortaleza [Socioeconomic status and its influence on the prevalence of overweight and obesity among adolescent school children in the city of Fortaleza, Brazil]. Revista de Nutricao 19: 531-538.

Campos MAG, Pedroso ERP, Lamounier JA, Colosimo EA, Abrantes MM (2006b) Estado nutricional e fatores associados em idosos [Nutritional status and related factors among elderly Brazilians]. Revista Da Associacao Medica Brasileira 52: 214-221.

Cantarero Prieto D, Pascual Saez M (2006) El Problema De La Obesidad: El Caso De Las Regiones Espanolas [The Obesity Problem: The Case of Spanish Regions. With English summary]. Estudios de Economia Aplicada 24: 837-859.

Carter KSF (1997) Correlates of health behaviors of community living older adults [Doctoral dissertation]. Charlottesville, VA, United States: University of Virginia.

Casper J (2004) Explaining adult tennis participants' participation frequency and purchase intention with the sport commitment model [Doctoral dissertation]. Greeley, CO, United States: University of Northern Colorado.

Castanheira M, Olinto MT, Gigante DP (2003) Associacao de variaveis socio-demograficas e comportamentais com a gordura abdominal em adultos: estudo de base populacional no Sul do Brasil [Socio-demographic and lifestyle factors associated with abdominal fat distribution in adults: a population-based survey in Southern Brazi]. Cadernos de Saude Publica 19 s55-s65.

Cavalcante JW, Daza CM, Pessoa C, Pacheco WS, de Menezes MG, et al. (1995) [Prevalence and sociocultural and economic aspects of hypertension in a health center of the northern area of Manaus]. Arquivos Brasileiros de Cardiologia 65: 493-496.

Chan MWL (1980) A principal component analysis of the income elasticities of demand for food commodities. Canadian Journal of Agricultural Economics 28: 38-45.

Chand R, Kumar P (2002) Long-term changes in coarse cereal consumption in India: Causes and implications. Indian Journal of Agricultural Economics 57: 316-325.

Chen J-L, Kennedy C, Yeh C-H, Kools S (2005) Risk factors for childhood obesity in elementary school-age Taiwanese children. Progress in Cardiovascular Nursing 20: 96-103.

Chetty VK, Haliburn C (1986) Estimation of price and income elasticities of demand for food grains in an economy with public distribution schemes. Indian Economic Review 21: 95-114.

Chriqui JF, Eidson SS, Bates H, Kowalczyk S, Chaloupka FJ (2008) State sales tax rates for soft drinks and snacks sold through grocery stores and vending machines, 2007. Journal of Public Health Policy 29: 226-249.

Chyun D, Lacey KO, Katten DM, Talley S, Price WJ, et al. (2006) Glucose and cardiac risk factor control in individuals with type 2 diabetes: Implications for patients and providers. Diabetes Educator 32: 925-939.

Cicek B, Ozturk A, Mazicioglu MM, Elmali F, Turp N, et al. (2009) The risk analysis of arm fat area in Turkish children and adolescents. Annals of Human Biology 36: 28-37.

Claro RM, Levy RB, Bandoni DH (2009) Influencia da renda sobre as despesas com alimentacao fora do domicilio, no Brasil, 2002-2003 [Influence of income on food expenditures away from home among Brazilian families, 2002-2003]. Cadernos de Saude Publica 25: 2489-2496.

Clausen J, Jensen G (1990) Are blood pressure levels increasing in Denmark? Journal of Internal Medicine 228: 443-450.

Coalter F (1993) Sports participation: Price or priorities? Leisure Studies 12: 171-182.

Colchero MA (2007) The economics of obesity in developing countries: The case of Cebu, Philippines 1983-2002 [Doctoral Dissertation]. Baltimore, MD, United States: John Hopkins University.

Collins AE, Pakiz B, Rock CL (2008) Factors associated with obesity in Indonesian adolescents. International Journal of Pediatric Obesity 3: 58-64.

Crespo CJ, Ainsworth BE, Keteyian SJ, Heath GW, Smit E (1999) Prevalence of physical inactivity and its relation to social class in U.S. adults: Results from the Third National Health and Nutrition Examination Survey, 1988-1994. Medicine and Science in Sports and Exercise 31: 1821-1827.

Cui Z, Huxley R, Wu Y, Dibley MJ (2010) Temporal trends in overweight and obesity of children and adolescents from nine Provinces in China from 1991-2006. International Journal of Pediatric Obesity 5: 365-374.

Dalla Costa MC, Cordoni LJ, Matsuo T (2007) Habito alimentar de escolares adolescentes de um municipio do oeste do Parana [Food habits of adolescent students from a municipality in western Parana, Brazil]. Revista de Nutricao 20: 461-471.

Dardis R, Soberon-Ferrer H, Patro D (1994) Analysis of leisure expenditures in the United States. Journal of Leisure Research 26: 309-321.

de Aquino RdC, Philippi ST (2002) Consumo infantil de alimentos industrializados e renda familiar na cidade de São Paulo [Association of children's consumption of processed foods and family income in the city of Sao Paulo, Brazil]. Revista de Saude Publica 36: 655-660.

Dhehibi B, Gil JM, Khaldi R (2002) Relation entre developpement economique, niveau des prix et ingestion de calories: Cas de la Tunisie [Relationship between economic development, price level and calorie intake: The case of Tunisia]. New Medit: Mediterranean Journal of Economics, Agriculture and Environment 1: 3-11.

Dhurandhar NV, Kulkarni PR (1992) Prevalence of obesity in Bombay. International Journal of Obesity 16: 367-375.

Dinkins JM, Edlow MD (1992) Expenditures for food away from home. Family Economics Review 5: 9-17.

Doo M, Seo JY, Kim Y (2009) Consumption patterns of snacks according to characteristics of family environment among Korean adolescents. The FASEB Journal: The Journal of the Federation of American Societies for Experimental Biology 23: 912.

Drachler ML, Macluf SP, Leite JC, Aerts DR, Giugliani ER, et al. (2003) Fatores de risco para sobrepeso em criancas no Sul do Brasil [Risk factors for overweight in children from Southern Brazil]. Cadernos de saude publica / Ministerio da Saude, Fundacao Oswaldo Cruz, Escola Nacional de Saude Publica 19: 1073-1081.

Drewnowski A, Hanks AS, Smith TG (2010) International trade, food and diet costs, and the global obesity epidemic. In: Hawkes C, Blouin C, Henson S, Drager N, Dube L, editors. Trade, food, diet and health: Perspectives and policy options. Ames, Iowa and Oxford: Wiley-Blackwell. pp. 77-90.

Dreyer LI, Dreyer S, Van der Merwe GG (2001) Fisieke aktiwiteit, lewenstyl en enkele sosio-ekonomiese aspekte se verband met die gesondheidstatus van mans in Potchefstroom [The relationship of physical activity, lifestyle and some socio-economic aspects with the health status of men in Potchefstroom]. South African Journal for Research in Sport, Physical Education & Recreation 23: 35-49.

Driskell JA, Clark AJ, Moak SW (1987) Longitudinal assessment of vitamin B-6 status in Southern adolescent girls. Journal of the American Dietetic Association 87: 307-310.

Dryson E, Metcalf P, Baker J, Scragg R (1992) The relationship between body mass index and socioeconomic status in New Zealand: Ethnic and occupational factors. The New Zealand Medical Journal 105: 233-235.

Duncan BB, Schmidt MI, Achutti AC, Polanczyk CA, Benia LR, et al. (1993) Socioeconomic distribution of noncommunicable disease risk factors in urban Brazil: The case of Porto Alegre. Bulletin of the Pan American Health Organization 27: 337-349.

Duncan SC, Duncan TE, Strycker LA, Chaumeton NR (2004) A multilevel analysis of sibling physical activity. Journal of Sport & Exercise Psychology 26: 57-68.

Durant MA (2008) A study of the determinants of body mass index in a population of Nova Scotia youth [Doctoral dissertation]. Halifax, Nova Scotia: Dalhousie University.

Ebashi S, Morino S, Ikeda M (1971) Survey on the leisure behavior and concept of workers in the big industries. Journal of Leisure & Recreation Studies: 27-34.

Edirisinghe N (1987) The Food Stamp Scheme in Sri Lanka: Costs, benefits, and options for modification. Washington, D.C: International Food Policy Research Center. 85-85 p.

Elston JA, Stanton KR, Levy DT, Acs ZJ (2007) Tax solutions to the external costs of obesity. In: Ács ZJ, Lyles A, Stanton KR, editors. Obesity, business and public policy. Northampton, MA: Edward Elgar Publishing. pp. 171-188.

Emmett JL, Havitz ME, McCarville RE (1996) A price subsidy policy for socio-economically disadvantaged recreation participants. Journal of Park & Recreation Administration 14: 63-80.

Enes CC, da Silva MV (2009) Disponibilidade de energia e nutrientes nos domicílios: O contraste entre as regiões Norte e Sul do Brasil [Energy and nutrients disposal in residences: The contrast between North and South regions of Brazil]. Ciencia & Saude Coletiva 14: 1267-1276.

Erem C, Hacihasanoglu A, Deger O, Kocak M, Topbas M (2008) Prevalence of dyslipidemia and associated risk factors among Turkish adults: Trabzon lipid study. Endocrine 34: 36-51.

Eyler AA, Haire-Joshu D, Brownson RC, Nanney MS (2004) Correlates of fat intake among urban, low income African Americans. American Journal of Health Behavior 28: 410-417.

Farvid M, Rabiee S, Homayoni F, Rashidkhani B, Arian V (2010) Determinants of fruit and vegetable consumption in type 2 diabetics in Tehran. [Arabic]. Iranian Journal of Endocrinology and Metabolism 12: 89-98,193.

Feng Q, Hu DY, Yang JG, Sun YH, Lu CL, et al. (2008) [Effects of socioeconomic status on the distribution of cardiovascular risk factors and clinical treatments of patients with acute myocardial infarction in Beijing]. Zhonghua liu xing bing xue za zhi [Chinese Journal of Epidemiology] 29: 430-433.

Fernald LC, Adler NE (2008) Blood pressure and socioeconomic status in low-income women in Mexico: A reverse gradient? Journal of Epidemiology and Community Health 62: e8.

Findlay LC, Garner RE, Kohen DE (2009) Children's organized physical activity patterns from childhood into adolescence. Journal of Physical Activity & Health 6: 708-715.

Fisher KL, von Tigerstrom B, Cameron C, Chad KE, Larre T, et al. (2011) The children's fitness tax credit: Does it influence parents' decisions regarding physical activity? Canadian Journal of Diabetes 35 194.

Fitness Business Canada (2008) Canadian Diabetes Association endorses FIC's adult fitness tax credit campaign Fitness Business Canada 9: 9-10.

Fletcher PC, Hirdes JP (1996) A longitudinal study of physical activity and self-rated health in Canadians over 55 years of age. Journal of Aging & Physical Activity 4: 136-150.

Fonseca MdJM, Faerstein E, Chor D, Lopes CS, Andreozzi VL (2006) Associacoes entre escolaridade, renda e indice de massa corporal em funcionarios de uma universidade no Rio de Janeiro, Brasil: Estudo Pro-Saude [Associations between schooling, income, and body mass index among public employees at an university in Rio de Janeiro, Brazil: The Pro- Saude Study]. Cadernos de Saude Publica 22: 2359-2367.

Forshee RA, Storey ML (2006) Demographics, not beverage consumption, is associated with diet quality. International Journal of Food Sciences & Nutrition 57: 494-511.

Forshee RA, Anderson PA, Storey ML (2004) The role of beverage consumption, physical activity, sedentary behavior, and demographics on body mass index of adolescents. International Journal of Food Sciences & Nutrition 55: 463-478.

Francois P, Perisse J, Kamoun A (1982) A Tunisian case study: The effects of household size and income on the probability of energy inadequacy. Food and Nutrition 8: 32-39.

Furman J (2005) Individual, social and physical environment determinants of physical activity among adolescents grades 10--12 in a suburban region [Doctoral dissertation]. New York, NY, United States: City University of New York.

Futrell MF, Kilgore LT, Windham F (1975) Nutritional status of black preschool children in Mississippi: Influence of income, mother's education, and food programs. Journal of the American Dietetic Association 66: 22-27.

Futrell MF, Kilgore LT, Windham F (1971) Nutritional status of Negro preschool children in Mississippi: Impact of education and income. Journal of the American Dietetic Association 59: 224-227.

Garcia M (1988) Food subsidies in the Philippines: Preliminary results. In: Pinstrup-Andersen P, editor. Food subsidies in developing countries: Costs, benefits, and policy options. Baltimore and London: Johns Hopkins University Press for the International Food Policy Research Institute. pp. 206-218.

Ge K, Weisell R, Guo X, Cheng L, Ma H, et al. (1994) The body mass index of Chinese adults in the 1980s. European Journal of Clinical Nutrition 48: s148-s154.

Gebre-Yohannes A, Rahlenbeck SI (1998) Coronary heart disease risk factors among blood donors in northwest Ethiopia. East African Medical Journal 75: 495-500.

Gharipour M, Siadat ZD, Maghroun M, Tavasolli AA (2009) Socioeconomic status and metabolic syndrome in Iranian population (IHHP study). Journal of Diabetes 1: A114-A115.

Gigante DP, Dias-da-Costa JS, Olinto MTA, Menezes AMB, Silvia M (2006) Obesidade da população adulta de Pelotas, Rio Grande do Sul, Brasil e associação com nível sócio-econômico [Adult obesity in Pelotas, Rio Grande do Sul, Brazil, and the association with socioeconomic status]. Cadernos de Saude Publica [Journal of Public Health] 22: 1873-1879.

Giray SH, Gunay T, Ucku R (2010) Effects of social inequalities on coronary heart disease risk factors: A population-based, cross-sectional study in Izmir. Anadolu Kardiyoloji Dergisi [Anatolian Journal of Cardiology] 10: 193-201.

Gomes FS, Anjos LA, Vasconcellos MTL (2010) Association between nutritional and socioeconomic status among adolescents in Niteri, Rio de Janeiro State, Brazil. Obesity Reviews 11: 57.

Govil KK, Bhatnager DP, Pant KC (1956) Dietary habits in Uttar Pradesh in relation to income. Journal of the Indian Medical Association 26: 138-141.

Gratton C, Taylor P (1985) Leisure expenditure. Leisure Management 5: 12-13.

Gregson J (2009) How inequality influences individuals through the built food environment [Doctoral dissertation]. Davis, CA, United States: University of California, Davis.

Grossman M, Mocan N, editors (2011) Economic aspects of obesity: A National Bureau of Economic Research Conference Report (Conference held November 10-11, 2008). Chicago and London: University of Chicago Press. xii p.

Grujic V, Dragnic N, Radic I, Harhaji S, Susnjevic S (2010) Overweight and obesity among adults in Serbia: Results from the National Health Survey. Eating & Weight Disorders 15: e34-42.

Guenther PM, Juan W, Lino M, Hiza HA, Fungwe TV, et al. (2009) Diet quality of low-income and higher-income Americans in 2003-2004 as measured by the Healthy Eating Index-2005. The FASEB Journal: The Journal of the Federation of American Societies for Experimental Biology 23.

Guenther PM (1986) Beverages in the diets of American teenagers. Journal of the American Dietetic Association 86: 493-499.

Guimaraes LV, Barros MBDA, Martins MSAS, Duarte EC (2006) Fatores associados ao sobrepeso em escolares [Factors associated with overweight in schoolchildren]. Revista de Nutricao 19: 5-17.

Guize L, Jaffiol C, Gueniot M, Bringer J, Giudicelli C, et al. (2008) [Diabetes and socio-economic deprivation. A study in a large French population]. Bulletin de l'Academie Nationale de Medecine 192: 1707-1723.

Gutierrez S, Marquez C, Colomer R (1994) Social inequities and food: an ecological study of the food sales of a supermarket chain. Gaceta Sanitaria 8: 304-309.

Hall LF (1985) Socio-economic factors and leisure activity participation among Blacks [Master's thesis]. University Park, PA, United States: Pennsylvania State University.

Hallal PC, Bertoldi AD, Goncalves H, Victora CG (2006) Prevalencia de sedentarismo e fatores associados em adolescentes de 10-12 anos de idade [Prevalence of sedentary lifestyle and associated factors in adolescents 10 to 12 years of age]. Cadernos de Saude Publica 22: 1277-1287.

Hannan P, French SA, Story M, Fulkerson JA (2002) AA pricing strategy to promote sales of lower fat foods in high school cafeterias: Acceptability and sensitivity analysis. American Journal of Health Promotion 17: ii1-6.

Hanson ER, Hartoonian N, Lesniak KT (2011) Predictors of adherence to healthcare provider hypertension behavioral treatment recommendations: BRFSS 2009 findings. Psychosomatic Medicine 73 A65.

Hashemi JM (2010) The prevalence of obesity among children aged 7-12 years in Jeddah Saudi Arabia and their parents' awareness of this problem [Doctoral dissertation]. Fayetteville, AR, United States: University of Arkansas.

Hawkes C (2010a) Understanding and changing the retail environment for obesity prevention. Obesity Reviews 11: 20-21.

Hawkes C, Blouin C, Henson S, Drager N, Dubé L (2010b) Trade, food, diet and health: Perspectives and policy options. Ames, Iowa and Oxford: Wiley-Blackwell.

Hawkes C (2009) Sales promotions and food consumption. Nutrition Reviews 67: 333-342.

Hayes DK, Denny CH, Keenan NL, Croft JB, Sundaram AA, et al. (2006) Racial/ethnic and socioeconomic differences in multiple risk factors for heart disease and stroke in women: Behavioral risk factor surveillance system, 2003. Journal of Women's Health 15: 1000-1008.

Haynes AL (2009) Time discounting, market and sociocultural factors associated with body mass index for a combined sample of four United States Hispanic populations [Doctoral dissertation]. Washington, DC, United States: Howard University.

He KS, Yoon KA, Kim WK, Park OJ (1992) Urban nutritional problems of Korea. The Southeast Asian Journal of Tropical Medicine and Public Health 23 69-76.

He Y-N, Yang Z, Xu J, Sha YM, Ren ZY, et al. (2008) [Analysis on influence factors of dietary supplement used in population aged above 45 years in Beijing]. Zhonghua yu fang yi xue za zhi [Chinese Journal of Preventive Medicine] 42: 823-826.

He Y-N, Zhai F-Y (2005) [Relative factors effecting dietary quality of Chinese adults]. Wei Sheng Yen Chiu [Journal of Hygiene Research] 34: 611-612.

Health Care Food & Nutrition Focus (1998) The "twinkie" tax: Opponents are angry, advocates believe it can curb obesity. Health Care Food & Nutrition Focus 15: 1, 3-4.

Hernandez D, Cole B, Dean L, Dove S, Adler S, et al. (2010) Subsidization of fruits and vegetables in two urban supermarkets leads to increases in purchasing and intake of these foods, as well as weight loss. Obesity Reviews 11: 60.

Hinds CD (2007) An economic investigation into the relationship between Food Stamp Program participation and obesity [Doctoral dissertation]. Washington, DC, United States: The American University.

Hodge AM, Dowse GK, Gareeboo H, Tuomilehto J, Alberti KG, et al. (1996) Incidence, increasing prevalence, and predictors of change in obesity and fat distribution over 5 years in the rapidly developing population of Mauritius. International Journal of Obesity & Related Metabolic Disorders: Journal of the International Association for the Study of Obesity 20: 137-146.

Hopper WC (1943) Income and food consumption. Canadian Journal of Economics and Political Science 9: 487-506.

Horgen KB (2001) Promoting healthy food choices: A health message and economic incentive intervention [Doctoral dissertation]. New Haven, CT, United States: Yale University.

Horta BL, Gigante DP, Victora CG, Barros FC, Oliveira I, et al. (2008b) [Early determinants of random blood glucose among adults of the 1982 birth cohort, Pelotas, Southern Brazil]. Revista de Saude Publica 42 93-100.

Howell SC, Loeb MB (1969) Income, age, and food consumption. The Gerontologist 9: 1-122.

Hoynes HW, Schanzenbach DW (2009) Consumption responses to in-kind transfers: Evidence from the introduction of the Food Stamp Program. American Economic Journal: Applied Economics 1: 109-139.

Hu Y (1991) [A case-control study on risk factors of female hypertension in Tianjin City]. Zhonghua liu xing bing xue za zhi [Chinese Journal of Epidemiology] 12: 197-199.

Huang CL, Raunikar R (1985) Effect of consigned income on food expenditures. Canadian Journal of Agricultural Economics 33: 315-329.

Huang YJ (2009) Socio-environmental correlates of physical activity and sedentary behaviors in primary schoolchildren in Hong Kong [Doctoral dissertation]. Hong Kong, The People's Republic of China: The Chinese University of Hong Kong.

Huffman WE, Huffman SK, Rickertsen K, Tegene A (2010) Over-nutrition and changing health status in high income countries. Forum for Health Economics and Policy 13.

Hufstader MA, Sias S, Vaidya V, White-Means S (2009) Metabolic syndrome risk factors for native born and first generation adolescents (12-17) in the United States. Value in Health 12 A18.

Hughes JP, McDowell MA, Brody DJ (2008) Leisure-time physical activity among US adults 60 or more years of age: Results from NHANES 1999-2004. Journal of Physical Activity & Health 5: 347-358.

Huybrechts I, De Bourdeaudhui I, De Henauw S (2010) Environmental opportunities and obstacles for physical activity and a healthy diet. Obesity Reviews 11: 471.

Hydrie MZ, Basit A, Ahmedani MY, Badruddin N, Masood MQ, et al. (2005) Comparison of risk factors for diabetes in children of different socioeconomic status. Journal of the College of Physicians and Surgeons Pakistan 15: 74-77.

Imperatore G, Bullard KM, Beckles GLA, Cheng YJ, Barker L, et al. Changes in socioeconomic disparities in cardiovascular disease (CVD) risk factors among US adults with diagnosed diabetes, 1988-2006; 2009; New Orleans, LA United States.

Inano M, Pringle DJ (1975) Dietary survey of low-income, rural families in Iowa and North Carolina. II. Family distribution of dietary adequacy. Journal of the American Dietetic Association 66: 361-365.

International Clinical Epidemiology Network (1996) Body mass index and cardiovascular disease risk factors in seven Asian and five Latin American centers: Data from the International Clinical Epidemiology Network (INCLEN). Obesity Research 4: 221-228.

International Council on Active Aging (2006) Older adults believe they eat a healthy diet. Research Review (International Council on Active Aging) 6: 4.

Jardim PC, Gondim MR, Monego ET, Moreira HG, Vitorino PV, et al. (2007) Hipertensão arterial e alguns fatores de risco em uma capital brasileira [High blood pressure and some risk factors in a Brazilian capital]. Arquivos Brasileiros de Cardiologia 88: 452-457.

Jeffery RW, French SA, Forster JL, Spry VM (1991) Socioeconomic status differences in health behaviors related to obesity: The healthy worker project. International Journal of Obesity 15: 689-696.

Jeffery RW, Forster JL, Folsom AR, Luepker RV, Jacobs DR, et al. (1989) The relationship between social status and body mass index in the Minnesota Heart Health Program. International Journal of Obesity 13: 59-67.

Jegasothy K, Duval Y (2003) Food demand in urban and rural Samoa. Pacific Economic Bulletin 18: 50-64.

Johansen H, Nargundkar M, Nair C, Taylor G, elSaadany S (1998) At risk of first or recurring heart disease. Health Reports 9: 19-29.

Kafwembe EM, Mwandu D, Sukwa Y (1996) Socio-economic status and serum vitamin A levels in Zambian children. The Central African Journal of Medicine 42: 70-72.

Kahn BE, Schmittlein DC (1992) The relationship between purchases made on promotion and shopping trip behavior. Journal of Retailing 68: 294-315.

Kahn HS, Williamson DF (1990) The contributions of income, education and changing marital status to weight change among US men. International Journal of Obesity 14: 1057-1068.

Kant AK, Block G, Schatzkin A, Ziegler RG, Nestle M (1991) Dietary diversity in the US population, NHANES II, 1976-1980. Journal of the American Dietetic Association 91: 1526-1531.

Kaplan GA, Lazarus NB, Cohen RD, Leu D-j (1991) Psychosocial factors in the natural history of physical activity. American Journal of Preventive Medicine 7: 12-17.

Keller HH, Ostbye T, Bright-See E, Campbell M (1999) Activity limitation and food intake in community-living seniors. Canadian Journal on Aging 18: 47-63.

Kelly SK, Branta CF, Morrison KM, Seefeldt V, Haubenstricker JL (2007) BMI differences between children ages 5 to 9 in California and Michigan. Journal of Sport & Exercise Psychology 29: s34-s34.

Kenney MA, McCoy JH, Kirby AL (1986) Nutrients supplied by food groups in diets of teenaged girls. Journal of the American Dietetic Association 86: 1549-1555.

Kim JA, Kim SM, Choi YS, Yoon D, Lee JS, et al. (2007) The prevalence and risk factors associated with isolated untreated systolic hypertension in Korea: The Korean National Health and Nutrition Survey 2001. Journal of Human Hypertension 21: 107-113.

Kimbro RT, Rigby E (2010) Federal food policy and childhood obesity: A solution or part of the problem? Health Affairs 29: 411-418.

Kling A (1976) Dietary behavior and constraint by cost. Annales de la Nutrition et de l'Alimentation 30: 447-452.

Koh ET, Caples V (1979) Frequency of selection of food groups by low-income families in southwestern Mississippi. Journal of the American Dietetic Association 74: 660-664.

Kohls KJ, Rudge SJ, Chancey C (1993) Calcium supplementation practices among university faculty and staff women. Journal of Applied Nutrition 45: 102-108.

Kolle E, Steene-Johannessen J, Klasson-Heggebo L, Andersen Lars B, Anderssen Sigmund A (2009) A 5-yr change in Norwegian 9-yr-olds' objectively assessed physical activity level. Medicine and Science in Sports and Exercise 41: 1368-1373.

Kozakiewicz K, Michalewska-Wludarczyk A, Korzeniowska B, Broda G, Pajak A, et al. (2010) Inverse correlation between socioeconomic status and risk of cardiovascular death. European Heart Journal 31: 228.

Kull M (1999) Socioeconomic factors affecting women's physical activity in Estonia. In Acta Academiae Olympiquae Estoniae Tartu, Estonia: University of Tartu. pp. 107-119.

Kumar SK, Alderman H (1988) Food consumption and nutritional effects of consumer-oriented food subsidies. In: Pinstrup-Andersen, editor. Food subsidies in developing countries: Costs, benefits, and policy options. Baltimore and London: Johns Hopkins University Press for the International Food Policy Research Institute. pp. 36-48.

La Rosa E, Valensi P, Cohen R, Soufi K, Robache C, et al. (2003) [Socioeconomic determinism of obesity in the Seine-Saint-Denis area]. Presse Medicale 32: 55-60.

Lafay L, Volatier JL, Martin A (2002) La restauration scolaire dans l'enquete INCA: Associations entre facteurs socio-demographiques, mode de vie et frequentation de la restauration scolaire [School catering in the INCA study: Relationships between socio-economic and lifestyle factors and frequency of use of the school cafeteria]. Cahiers de Nutrition et de Dietetique 37: 36-44.

Laitinen S, Rasanen L, Viikari J, Akerblom HK (1995) Diet of Finnish children in relation to the family's socio-economic status. Scandinavian Journal of Social Medicine 23: 88-94.

Laraki K (1988) Food consumption and food subsidies in Morocco: Justification for policy reform [Doctoral dissertation]. Ithica, NY: Cornell University.

Laurier D, Guiguet M, Chau NP, Wells JA, Valleron AJ (1992) Prevalence of obesity: A comparative survey in France, the United Kingdom and the United States. International Journal of Obesity & Related Metabolic Disorders: Journal of the International Association for the Study of Obesity 16: 565-572.

Lazaridis P (2002) Household consumption of food-away-from-home in Greece. RISEC: International Review of Economics and Business 49: 415-430.

Lea SA, Gasevic D, Schuurman N (2011) The relationship between supermarket characteristics and body mass index of shoppers. Canadian Journal of Diabetes 35: 157.

Leal C, Chaix B (2010) Are obesity outcomes associated with neighborhood socioeconomic characteristics? Findings from the French RECORD Cohort study. Obesity Reviews 11: 330.

Lee CY, Hwang SY, Ham OK (2007) Factors associated with physical inactivity among Korean men and women. American Journal of Health Behavior 31: 484-494.

Leino M, Raitakari OT, Porkka KVK, Helenius HYM, Viikari JSA (2000) Cardiovascular risk factors of young adults in relation to parental socioeconomic status: The Cardiovascular Risk in Young Finns Study. Annals of Medicine 32: 142-151.

Levy M (2004) Tax incentives. Fitness Business Canada 5: 50-50.

Li P, Campbell J, Tutor C (2004) Using BRFSS data to estimate prevalence of obesity and physical activities in Oklahoma. The Journal of the Oklahoma State Medical Association 97: 404-408; quiz 409-410.

Lima ES, Euclydes MP, Cruz TA, Casali AD (1989) [Socioeconomic conditions, food and nutrition of the urban population in a locality of the State of Minas Gerais (Brazil)]. Revista de Saude Publica 23: 410-421.

Lizarzaburu JL, Palinkas LA (2002) Immigration, acculturation, and risk factors for obesity and cardiovascular disease: A comparison between Latinos of Peruvian descent in Peru and in the United States. Ethnicity & Disease 12: 342-352.

Lo KH, Pan LY, Lin MC, Chang HY, Shih YT (2003) [The analysis of special physical inactivity, higher energy intakes and body mass index of children in Taiwan: Results of 2001 National Health Interview Survey]. Taiwan Journal of Public Health 22: 474-482.

Loland NW (2004) Exercise, health, and aging. Journal of Aging and Physical Activity 12: 170-184.

Lopes JA, Longo GZ, Peres KG, Boing AF, de Arruda MP (2010) Fatores associados a atividade fisica insuficiente em adultos: Estudo de base populacional no sul do Brasil [Factors associated with insufficient physical activity: A population-based study in southern Brazil]. Revista Brasileira de Epidemiologia 13: 689-698.

Lopez LM, Habicht JP (1987) Food stamps and the iron status of the U.S. elderly poor. Journal of the American Dietetic Association 87: 598-603.

Lopez NA, Viudes dVA (2010) Posibilidades y limitaciones de las politicas fiscales comoinstrumentos de salud: Los impuestos sobre consumos nocivos. Informe SESPAS 2010 [Possibilities and limitations of fiscal policies as health instruments: Taxes on harmful consumption. SESPAS Report 2010.]. Gaceta Sanitaria 24: 85-89.

Lora KR, Lewis N, Eskridge K, Travnicek D (2009) Association of age, socioeconomic status (SES) and acculturation with intake of omega-3 fatty acids in Latinas. The FASEB Journal: The Journal of the Federation of American Societies for Experimental Biology 23: 551.527.

Los-Rycharska E, Nieclawska A (2010a) Analiza wybranych cech diety dzieci w wieku przedszkolnym i poniemowlecym w zaleznosci od sytuacji spoleczno- ekonomicznej rodzin [The analysis of chosen features of young children's diet in dependence on socio-economic situation]. Pediatria Polska 85: 471-480.

Los-Rycharska E, Nieclawska A (2010b) Spozycie pokarmow typu fast-food przez dzieci w wieku poniemowlecym i przedszkolnym [Fast-food consumption by 2-3 years old and preschool children]. Pediatria Polska 85: 345-352.

Ma W-J, Xu Y-J, Fu C-X, Chen M-F, Xu H-F, et al. (2005) [A cross sectional survey on serum lipid level and its influencing factors in children aged 3-14 years in Guangdong province]. Chung-Hua Hsin Hsueh Kuan Ping Tsa Chih [Chinese Journal of Cardiology] 33: 950-955.

Ma W-J, Xu Y-J, Li J-S, Xu H-F, Nie S-P, et al. (2004b) [Study on the epidemiological characteristics of overweight and obesity among population aged eighteen and over in Guangdong Province in 2002]. Zhonghua liu xing bing xue za zhi [Chinese Journal of Epidemiology] 25: 1035-1038.

Machado PA, Sichieri R (2002a) Waist-to-hip ratio and dietary factors in adults. Revista de Saude Publica 36: 198-204.

Machado PAN, Sichieri R (2002b) Relação cintura-quadril e fatores de dieta em adultos [Waist-to-hip ratio and dietary factors in adults]. Revista de Saude Publica 36: 198-204.

Makinen T, Kestila L, Borodulin K, Martelin T, Rahkonen O, et al. (2010) Occupational class differences in leisure-time physical inactivity--contribution of past and current physical workload and other working conditions. Scandinavian Journal of Work, Environment & Health 36: 62-70.

Malone CM (1997) The prevalence of physical activity or inactivity in a rural African American community. Journal of National Black Nurses' Association 9: 58-65.

Malyutina S, Bobak M, Soboleva S, Malyutina D, Peasey A, et al. (2009) Whether income is associated with blood pressure levels in a population sample? European Heart Journal 30: 438.

Manore MM, Vaughan LA, Carroll SS (1989) Iron status in free-living, low income very elderly. Nutrition Reports International 39: 1-12.

Marcus C, Rossner S (2011) [Specific purchase tax on sweetened beverages--are we there soon?]. Lakartidningen 108: 188-189.

Mark S (2010) Vitamin D status and recommendations to improve vitamin D status in Canadian youth. Applied Physiology, Nutrition, & Metabolism- Physiologie Appliquee, Nutrition et Metabolisme 35: 718.

Marshall SJ, Jones DA, Ainsworth BE, Reis JP, Levy SS, et al. (2007) Race/ethnicity, social class, and leisure-time physical inactivity. Medicine and Science in Sports and Exercise 39: 44-51.

Marti B, Salonen JT, Tuomilehto J, Puska P (1988) 10-Year trends in physical activity in the eastern Finnish adult population: Relationship to socioeconomic and lifestyle characteristics. Acta Medica Scandinavica 224: 195-203.

Martinez SM (2010) Individual, social and environmental correlates of physical activity among Latinos in San Diego county [Doctoral dissertation]. San Diego, CA, United States: University of California, San Diego and San Diego State University.

Martin-Mollard ML (2008) Socioeconomic status and childhood overweight in Alameda county: 2003 and 2005 California health interview survey [Doctoral dissertation]. Berkeley, CA, United States: University of California, Berkeley.

Martins IS, Cavalcanti ML, Mazzilli RN (1977) Relação entre consumo alimentar e renda familiar na cidade de Iguape, S. Paulo (Brasil) [Relation of food consumption to family income in the city of Iguape, S. Paulo, Brazil]. Revista de Saude Publica 11: 27-38.

Masson CR, Dias-da-Costa JS, Olinto MT, Meneghel S, Costa CC, et al. (2005) Prevalencia de sedentarismo nas mulheres adultas da cidade de Sao Leopoldo, Rio Grande do Sul, Brasil [Prevalence of physical inactivity in adult women in Sao Leopoldo, Rio Grande do Sul, Brazil.]. Cadernos de saude publica / Ministerio da Saude, Fundacao Oswaldo Cruz, Escola Nacional de Saude Publica 21: 1685-1695.

Mathur N, Srivastava AK, Rastogi SK (2005) The influence of socio-economic status, lifestyle and health outcomes with body mass index in North Indian adult population. Journal of Ecophysiology and Occupational Health 5: 105-111.

Mathus-Vliegen EMH (1998) Overgewicht. II. Determinanten van overgewich en strategieen voor preventie [Overweight. II. Determinants of overweight and strategies for prevention]. Nederlands Tijdschrift voor Geneeskunde [Dutch Journal of Medicine] 142: 1989-1995.

Mauny F, Viel JF, Roubaux F, Ratsimandresy R, Sellin B (2003) Blood pressure, body mass index and socio-economic status in the urban population of Antananarivo (Madagascar). Annals of Tropical Medicine & Parasitology 97: 645-654.

McGuire LC, Ahluwalia IB, Strine TW (2006) Chronic disease-related behaviors in U.S. older women: Behavioral risk factor surveillance system, 2003. Journal of Women's Health 15: 3-7.

McKee M, Pomerleau J, Shapo L (2004) Physical Inactivity in a country in transition: A population-based survey in Tirana City, Albania. Scandinavian Journal of Public Health 32: 60-67.

McPherson BD, Curtis JE (1985) Regional differences in the physical activity patterns of Canadian adults. Unpublished Paper: 29.

Mehta P, Shringarpure B (2000) Diet nutrition and health profile of elderly population of urban Baroda. Indian Journal of Public Health 44: 124-128.

Mei J, Chen Q, Sun J, Xiong R (1998) [The food habit and its affecting factors of preschool children in Guangzhou]. Wei Sheng Yan Jiu [Journal of Hygiene Research] 27: 334-336.

Mei Z, Cogswell ME, Looker AC, Pfeiffer CM, Cusick SE, et al. (2011) Assessment of iron status in US pregnant women from the national health and nutrition examination survey (NHANES), 1999-2006. American Journal of Clinical Nutrition 93: 1312-1320.

Merchant VA (2010) Has the time come for a tax on soda pop? Journal of Michigan Dental Association 92: 12-13.

Mergos GJ, Donatos GS (1989) Demand for food in Greece: An almost ideal demand system analysis. Journal of Agricultural Economics 40: 178-184.

Michaud C, Baudier F, Loundou A, Le B, Janvrin MP, et al. (1998a) Habitudes, consommations et connaissances alimentaires des Francais en situation de precarite financiere [Nutrition habits and food consumption in low income French people]. Sante Publique 10: 333-347.

Michaud C, Baudier F, Loundou A, Le Bihan G, Janvrin MP, et al. (1998b) [Food habits, consumption, and knowledge of a low-income French population]. Sante Publique (Vandoeuvre-Les-Nancey) 10: 333-347.

Milliron B-J (2011) An ecological approach to investigating the influences of obesity [Doctoral dissertation]. Phoenix, AZ, United States: Arizona State University.

Mitchell D, Hoppe M (2006) From Marrakesh to Doha: Effects of removing food subsidies on the poor. In: Newfarmer R, editor. Trade, Doha, and Development: A Window into the Issues. Washington, D.C: World Bank. pp. 341-352.

Mitola AH (2008) Using technology to study dietary lapse and weight loss maintenance among weight-reduced adults [Doctoral dissertation]. Baltimore, MD, United States: John Hopkins University.

Mo F, Turner M, Krewski D, Mo Fan D (2005) Physical inactivity and socioeconomic status in Canadian adolescents. International Journal of Adolescent Medicine and Health 17: 49-57.

Mohr M, Pose G (1971) [Food consumption and calorie and nutrient intake in workers' and employees households in the German Democratic Republic as related to income]. Nahrung 15: 343-352.

Molina JA (2002) Modelling the demand behaviour of Spanish consumers using parametric and non-parametric approaches. Journal for Studies in Economics and Econometrics 26: 19-36.

Mondini L, de Moraes SA, de Freitas ICM, Gimeno SGA (2010) Fruit and vegetable intake by adults in Ribeirao Preto, Southeastern Brazil. Revista de Saude Publica 44: 686-694.

Monteiro CA, de Freitas IC, Baratho RM (1989) [Health, nutrition and the social classes: The empirical link evident in a large urban center, Brazil]. Revista de Saude Publica 23: 422-428.

Monteiro CA, Mondini L, de Souza AL, Popkin BM (1995) The nutrition transition in Brazil. European Journal of Clinical Nutrition 49: 105-113.

Monteiro P, Victora C, Barros F (2004) [Social, familial, and behavioral risk factors for obesity in adolescents]. Pan American Journal of Public Health 16: 250-258.

Morgan KJ (1987) Consumer demand for nutrients in food. In: Raunikar R, Huang C-L, editors. Food demand analysis: Problems, issues, and empirical evidence. Ames: Iowa State University Press. pp. 219-235.

Moro D, Moschini G (2002) Separable almost ideal demand system (A). Ames, IA, United States: Iowa State University Dept. of Economics.

Mullally CC, Alston JM, Sumner DA, Townsend MS, Vosti SA (2008) Proposed modifications to the food stamp program: Likely effects and their policy implications. In: Blass EM, editor. Obesity: Causes, mechanisms, prevention, and treatment. Sunderland, MA: Sinauer Associates; US. pp. 319-369.

Mushi-Brunt CR (2007) Fruit and vegetable intake and weight status among pre-adolescent children: An ecological perspective [Doctoral dissertation]. Saint Louis, MO, United States: Saint Louis University.

Myres AW, Kroetsch D (1978) The influence of family income on food consumption patterns and nutrient intake in Canada. Canadian Journal of Public Health/Revue Canadienne de Sante Publique 69: 208-221.

Naidu AN, Rao NP (1994) Body mass index: A measure of the nutritional status in Indian populations. European Journal of Clinical Nutrition 48: s131-s140.

Narksawat K, Punyaratabundhu P, Podhipak A, Podang J (2003) Atheromatous risk factors among Thai labor forces by socioeconomic status. Journal of the Medical Association of Thailand 86: 655-665.

Nayga RM (1995) Presence of children and household food expenditures at home and away from home. Journal of Consumer Studies and Home Economics 19: 235-245.

Nayga RM (1994b) Impact of sociodemographic and economic factors on sodium intake in the U.S.A. Journal of Consumer Studies and Home Economics 18: 305-314.

Nelson PE (1950) Relation of price to food selection. Journal of the American Dietetic Association 26: 769-770.

Nestrick W (1939) Constructional activities of adult males [Doctoral dissertation]. New York, NY, United States: Columbia University.

Neumann AIDLCP, Shirassu MM, Fisberg RM (2006) Consumo de alimentos de risco e protecao para doencas cardiovasculares entre funcionarios publicos [Consumption of protective and promotive foods in cardiovascular diseases among public employees]. Revista de Nutricao 19: 19-28.

Neutzling MB, Rombaldi AJ, Azevedo MR, Hallal PC (2009) Factors associated with fruit and vegetable intake among adults in a southern Brazilian city. [Portuguese] Fatores associados ao consumo de frutas, legumes e verduras em adultos de uma cidade no sul do Brasil. Cadernos de Saude Publica 25: 2365-2374.

Ng SW (2009) Understanding changes in diet, physical activity and weight among adults in China [Doctoral dissertation]. Chapel Hill, NC, United States: University of North Carolina at Chapel Hill.

Nicholls SG, Williams G, Wickins-Drazilova D, Siani A, De Henauw S, et al. (2011) Money's too tight (to mention): Taxation and subsidisation as obesity intervention measures. International Journal of Obesity 35: s159.

Nicklett EJ, Szanton S, Sun K, Ferrucci L, Fried LP, et al. (2011) Neighborhood socioeconomic status is associated with serum carotenoid concentrations in older, community-dwelling women. Journal of Nutrition 141: 284-289.

Nogueira A, Marcopito L, Lanas F, Galdames D, Jialiang W, et al. (1994) Socio-economic status and risk factors for cardiovascular disease: A multicentre collaborative study in the International Clinical Epidemiology Network (INCLEN). Journal of Clinical Epidemiology 47: 1401-1409.

Nogueira AR, Alves PM, de Miranda RF, Boechat NL (1990) [Cholesterol and other cardiovascular risk factors among employees of the Universidade Federal do Rio de Janeiro. Prevalence and influence of social variables]. Arquivos Brasileiros de Cardiologia 55: 227-232.

Novak NL, Brownell KD (2011) Taxation as prevention and as a treatment for obesity: The case of sugar-sweetened beverages. Current Pharmaceutical Design 17: 1218-1222.

Novotny R (1987) Preschool child feeding, health and nutritional status in Gualaceo, Ecuador. Archivos Latinoamericanos de Nutricion 37: 417-443.

Ohls JC, Beebout H (1993) The food stamp program: Design tradeoffs, policy, and impacts: A mathematica policy reserach study (Mathematica Policy Research Study Series). Washington, DC: Urban Institute Press.

Olivares CS, Bustos ZN, Lera ML, Zelada ME (2007) Estado nutricional, consumo de alimentos y actividad fisica en escolares mujeres de diferente nivel socioeconomico de Santiago de Chile [Nutritional status, food consumption and physical activity in female school children of different socioeconomic levels from Santiago, Chile]. Revista Medica de Chile 135: 71-78.

Oliver LN (2008) Examining the influence of the neighbourhood environment on childhood obesity [Doctoral dissertation]. Surrey, BC, Canada: Simon Fraser University.

Ory FG, Shukla A, Kumar S, Harte JM (1996) Body mass index of tannery workers in Kanpur, India. Indian Journal of Medical Research 103: 232-240.

Paek K-W, Hong Y-M (2006) Health behavior factors affecting waist circumference as an indicator of abdominal obesity. Yebang Uihakhoe Chi [Journal of Preventive Medicine & Public Health] 39: 59-66.

Paredes-Rojas RR, Solomons HC (1982) Food for thought: Impact of a supplemental nutritional program on low-income preschool children. Pediatric Nursing 8: 315-317.

Park H (2007) Longitudinal relationships between physical activity, sedentary behaviors, and obesity in children and adolescents [Doctoral dissertation]. Chapel Hill, NC, United States: University of North Carolina, Chapel Hill.

Pascual C, Regidor E, Gutierrez-Fisac JL, Martinez D, Calle ME, et al. (2005) Material well-being of the province of residence and leisure-time physical inactivity. Gaceta Sanitaria 19: 424-432.

Patel D, Lambert EV, da Silva R, Greyling M, Kolbe-Alexander T, et al. (2011) Participation in fitness-related activities of an incentive-based health promotion program and hospital costs: A retrospective longitudinal study. American Journal of Health Promotion 25: 341-348.

Pearte CA, Gary TL, Brancati FL (2004) Correlates of physical activity levels in a sample of urban African Americans with type 2 diabetes. Ethnicity & Disease 14: 198-205.

Pedersen KM, Worre-Jensen AL (2006) [Obesity and health economics. Review and status]. Ugeskrift for Laeger 168: 196-201.

Pereira A, Martinez T, Vieira L. Cholesterol - A epidemiological study in children and adolescents; 2009; Boston, MA United States.

Pereira RF, Lajolo FM, Hirschbruch MD (2003) Consumo de suplementos por alunos de academias de ginastica em Sao Paulo [Supplement consumption among fitness center users in Sao Paulo, Brasil]. Revista de Nutricao 16: 265-272.

Petersen AM, Leet TL, Brownson RC (2005) Correlates of physical activity among pregnant women in the United States. Medicine and Science in Sports and Exercise 37: 1748-1753.

Peterson DC (1991) The effects of changes in the demographic and income distributions on the demand for food in the United States [Doctoral dissertatoin]. Ithica, NY, United States: Cornell University.

Pinstrup-Andersen P (1993) Integrating political and economic considerations in programs and policies to improve nutrition: Lessons learned. In: Pinstrup-Andersen P, editor. The political economy of food and nutrition policies. Baltimore and London: Johns Hopkins University Press for the International Food Policy Research Institute. pp. 225-235.

Pinstrup-Andersen P, Alderman H (1988) The effectiveness of consumer-oriented food subsidies in reaching rationing and income transfer goals. In: Pinstrup-Andersen P, editor. Food subsidies in developing countries: Costs, benefits, and policy options. Baltimore and London: Johns Hopkins University Press for the International Food Policy Research Institute. pp. 21-35.

Pollitt E (1975) Failure to thrive: Socioeconomic, dietary intake and mother-child interaction data. Federation Proceedings 34: 1593-1597.

Popkin BM, Paeratakul S, Zhai F, Ge K (1995) Dietary and environmental correlates of obesity in a population study in China. Obesity research 3 s135-s143.

Posner BEM, Smigelski CG, Krachenfels MM (1987) Dietary characteristics and nutrient intake in an urban homebound population. Journal of the American Dietetic Association 87: 452-456.

Powell LM, Christopher A, Chaloupka FJ, O'Malley PM, Johnston LD (2006) Access to fast food and food prices: Relationship with fruit and vegetable consumption and overweight among adolescents. In: Bolin K, Cawley J, editors. The Economics of Obesity. Oxford, United Kingdom: Elsevier Ltd. pp. 23-48.

Proper KI, Cerin E, Owen N (2006) Neighborhood and individual socio-economic variations in the contribution of occupational physical activity to total physical activity. Journal of Physical Activity & Health 3: 179-190.

Pruitt JL, Mack N, Murayi T (1994) Patterns of sedentary lifestyle in Missouri. Missouri Medicine 91: 675-679.

Radhakrishna R, Ravi C (1992) Effects of growth, relative price and preferences on food and nutrition. Indian Economic Review 27: 303-323.

Raj KN (1983) Prices, subsidies and access to food. Indian Economic Review 18: 157-167.

Rashad I (2006c) Obesity and diabetes: The roles that prices and policies play. In: Bolin K, Cawley J, editors. The Economics of Obesity. Oxford, United Kingdom: Elsevier Ltd. pp. 113-128.

Raudsepp L, Viira R (2000) Sociocultural correlates of physical activity in adolescents. Pediatric Exercise Science 12: 51-60.

Reaburn JA, Krondl M, Lau D (1979) Social determinants in food selection. Journal of the American Dietetic Association 74: 637-641.

Reddy BN (1998) Body mass index and its association with socioeconomic and behavioral variables among socioeconomically heterogeneous populations of Andhra Pradesh, India. Human Biology 70: 901-917.

Reddy BS (1982) Public intervention in foodgrains distribution: Dual price and income transfers. Economic Affairs 27: 397.

Redwood D, Schumacher MC, Lanier AP, Ferucci ED, Asay E, et al. (2009) Physical activity patterns of American Indian and Alaskan Native people living in Alaska and the Southwestern United States. American Journal of Health Promotion 23: 388-395.

Reich CM (1965) Socioeconomic factors related to household participation in community recreation [Doctoral dissertation]. University Park, PA, United States: Pennsylvania State University.

Rogers BL (1988) Design and implementation considerations for consumer-oriented food subsidies. In: Pinstrup-Andersen P, editor. Food subsidies in developing countries: Costs, benefits, and policy options. Baltimore and London: Johns Hopkins University Press for the International Food Policy Research Institute. pp. 127-146.

Roos E, Prattala R, Lahelma E, Kleemola P, Pietinen P (1996) Modern and healthy? Socioeconomic differences in the quality of diet. European Journal of Clinical Nutrition 50: 753-760.

Saez N, Aleixandre M, Herraiz T (1996) Leisure sports activities. Revista de Psicologia de la Educacion 20: 83-98.

Salles-Costa R, Heilborn ML, Werneck GL, Faerstein E, Lopes CS (2003) Genero e pratica de atividade fisica de lazer [Gender and leisure-time physical activity]. Cadernos de saude publica / Ministerio da Saude, Fundacao Oswaldo Cruz, Escola Nacional de Saude Publica 19: s325-s333.

Salles-Costa R, Werneck GL, Lopes CS, Faerstein E (2003) Associacao entre fatores socio-demograficos e pratica de atividade fisica de lazer no Estudo Pro-Saude [The association between socio-demographic factors and leisure-time physical activity in the Pro-Saude Study]. Cadernos de saude publica / Ministerio da Saude, Fundacao Oswaldo Cruz, Escola Nacional de Saude Publica 19: 1095-1105.

Samardzija MK (2009) Variables that may affect physical activity levels in African American females with type 2 diabetes [Doctoral dissertation]. Chicago, IL, United States: Rush University.

Santos LM, Assis AM, Martins MC, Araujo MP, Morris SS, et al. (1996) Situacao nutricional e alimentar de pre-escolares no semi-arido da Bahia (Brasil): II--hipovitaminose A [Nutritional status of pre-school children of the semi-arid region of Bahia (Brazil): II--Vitamin A deficiency]. Revista de Saude Publica 30: 67-74.

Santos R, Santos MP, Ribeiro JC, Mota J (2009) Physical activity and other lifestyle behaviors in a Portuguese sample of adults: Results from the Azorean Physical Activity and Health Study. Journal of Physical Activity & Health 6: 750-759.

Saxton-Ross A (2009) Physical activity and the urban form: How the built environment, residential density, residential segregation, income inequality, race, gender and education, influence physical activity in Atlanta, Georgia [Doctoral dissertation]. Washington, DC, United States: Howard University.

Schaeffer C (2007) A study of the influences of nutrition, physical activity, income, and family lifestyle choices on the overweight of elementary-age children [Doctoral dissertation]. Milwaukee, WI, United States: Cardinal Stritch University.

Schroeter C (2005) Determining the impact of food price and income changes on body weight [Doctoral dissertation]. Lafayette, IN, United States: Purdue University.

Seclen-Palacin JA, Jacoby ER (2003) [Sociodemographic and environmental factors associated with sports physical activity in the urban population of Peru]. Pan American Journal of Public Health 14: 255-264.

Seeholzer E, Thomas C, Snitily B, Clark M, Brobbey V, et al. (2010) Fruit and vegetable consumption, attitudes and knowledge among enrollees ofan urban local produce program. Journal of General Internal Medicine 25: s282.

Shah M, Jeffery RW, Hannan PJ, Onstad L (1989) Relationship between socio-demographic and behaviour variables, and body mass index in a population with high-normal blood pressure: Hypertension prevention trial. European Journal of Clinical Nutrition 43: 583-596.

Shatenstein B, Ghadirian P (1996) Nutrient patterns and nutritional adequacy among French-Canadian children in Montreal. Journal of the American College of Nutrition 15: 264-272.

Shi Y, De G, Morrison H (2011) Increasing blood pressure and its associated factors in Canadian children and adolescents from Canadian Health Measures Survey. Canadian Journal of Diabetes 35 184.

Sichieri R, Castro JFG, Moura A-bS (2003) [Factors associated with dietary patterns in the urban Brazilian population]. Cadernos de Saude Publica 19 s47-s53.

Simonton SZ (2008) Social inequalities in childhood obesity. Obesity in childhood and adolescence, Vol 1: Medical, biological, and social issues. Westport, CT: Praeger Publishers/Greenwood Publishing Group; US. pp. 61-91.

Sobel R, Pandit A, Wolf M (2010) Race, literacy and hypertension control among patients in safety net clinics. Journal of General Internal Medicine 25: s374-s375.

Son KY, Cho BL, Lim JW (2010) Different association of social determinants with body mass index between age groups in Korea. Obesity Reviews 11: 444.

Song H-J (2008) Development, implementation and evaluation of an urban food store intervention for Korean corner stores in Baltimore city [Doctoral dissertation]. Baltimore, MD, United States: John Hopkins University.

Sonmez K, Pala S, Mutlu B, Izgi A, Bakal RB, et al. (2004) [Distribution of risk factors according to socioeconomic status in male and female cases with coronary artery disease]. Anadolu Kardiyoloji Dergisi 4: 301-305.

Sousa RMRDP, Sobral DP, da Paz SMRS, Martins MDCDC (2007) Prevalencia de sobrepeso e obesidade entre funcionarios plantonistas de unidades de saude de Teresina, Piaui [Overweight and obesity prevalence in employees of healthcare units in Teresina, Piaui, Brazil]. Revista de Nutricao 20: 473-482.

Spallek M, Turner C, Spinks A, Bain C, McClure R (2006) Walking to school: Distribution by age, sex and socio-economic status. Health Promotion Journal of Australia 17: 134-138.

Spana TM, Rodrigues RCM, Gallani MCBJ, Mendez RDR (2010) [Physical activity behavior among coronary outpatients according to socio-demographic and clinical profile]. Revista Brasileira de Enfermagem 63: 741-748.

Stelmach W, Kaczmarczyk-Chalas K, Bielecki W, Drygas W (2004b) The association between income, education, control over life and health in a large urban population of Poland. International Journal of Occupational Medicine and Environmental Health 17: 299-310.

Stelmach W, Kaczmarczyk-Chalas K, Bielecki W, Drygas W (2004c) The impact of income, education and health on lifestyle in a large urban population of Poland (Cindi Programme). International Journal of Occupational Medicine & Environmental Health 17: 393-401.

Stevens J, Lthrop A, Bradish C (2005) Tracking generation Y: A contemporary sport consumer profile. Journal of Sport Management 19: 254-277.

Stuff JE, Casey PH, Connell CL, Champagne CM, Gossett JM, et al. (2007) Household food insecurity and obesity, chronic disease, and chronic disease risk factors. Journal of Hunger and Environmental Nutrition 1: 43-62.

Sturm R (2010b) Economic approaches to prevent obesity: Taxes, subsidies and regulation. Obesity Reviews 11: 18-19.

Subar AF, Heimendinger J, Patterson BH, Krebs-Smith SM, Pivonka E, et al. (1995) Fruit and vegetable intake in the United States: The baseline survey of the Five A Day for Better Health Program. American Journal of Health Promotion 9: 352-360.

Sung MH, Lim YM (2003) [The study on health promoting lifestyle of the elderly women]. Taehan Kanho Hakhoe Chi 33: 762-771.

Sygnowska E, Piwonska A, Waskiewicz A, Drygas W (2010) Factors associated with metabolic syndrome in Polish population. WOBASZ study. European Journal of Cardiovascular Prevention and Rehabilitation 17: s45.

Sygnowska E, Waskiewicz A (2009) Ocena rozpowszechnienia i wielkości suplementacji witaminami i składnikami mineralnymi w populacji Polskiej [Evaluation of prevalence and magnitude of vitamins and minerals supplementation in Polish population]. Roczniki Panstwowego Zakladu Higieny [Annals of the National Institute of Hygiene] 60: 167-170.

Szarfarc SC (1979) [The adequacy of the consumption of food of animal origin and its relation to family income]. Revista de Saude Publica 13: 26-31.

Taks M, Renson R, Vanreusel B (1995) Social stratification in sport: A matter of money or taste? European Journal For Sport Management 2: 4-14.

Tarasuk V, Fitzpatrick S, Ward H (2010) Nutrition inequities in Canada. Applied Physiology, Nutrition, & Metabolism (Physiologie Appliquee, Nutrition et Metabolisme) 35: 172-179.

Taylor OG, Oyediran OA, Bamgboye AE, Afolabi BM, Osuntokun BO (1996) Profile of some risk factors for coronary heart disease in a developing country: Nigeria. African Journal of Medicine and Medical Sciences 25: 341-346.

Thavorn K, Laporte A, Coyte PC (2011) What drives the change in body mass index over a 14-year period? A panel data analysis. Canadian Journal of Diabetes 35 143.

Thompson CS, Tinsley AW (1978) Income expenditure elasticities for recreation: Their estimation and relation to demand for recreation. Journal of Leisure Research 10: 265-270.

Tomar R (2010) Health, fitness and economic status: A comparative study Ovidius University Annals, Series Physical Education & Sport/Science, Movement & Health 10: 126-132.

Tomiak M, Gentleman JF (1993) Risk factors for hypertension as measured by the Canada Health Survey. Health Reports 5: 419-428.

Tosini N (2008) The socioeconomic determinants and consequences of women's body mass [Doctoral dissertation]. Philadelphia, PA, United States: University of Pennsylvania.

Townsend MS (2001) The relationships of income and food insecurity with overweight in women [Doctoral dissertation]. University Park, PA, United States: Pennsylvania State University.

Troiano RP, Flegal KM (1998) Overweight children and adolescents: Description, epidemiology, and demographics. Pediatrics 101: 497-504.

Tsang SN, Pycz LA, Herbold NH (2007) Dietary supplement use among physically active multiethnic adults. Topics in Clinical Nutrition 22: 246-257.

Turrell G (1998) Determinants of healthy food choice in a population-based sample. American Journal of Health Behavior 22: 342-357.

United Nations Food and Agriculture Association (1985) Review of food consumption surveys--1985. Household food consumption by economic groups. FAO Food and Nutrition Paper 35: i-xxiv, 1.

Valero G, Jorge N (2006) Estimacion de elasticidades e impuestos optimos a los bienes mas consumidos en Mexico. (With English summary). Estudios Economicos 21: 127-176.

Vedana EHB, Peres MA, Neves Jd, Rocha GCd, Longo GZ (2008) [Prevalence of obesity and potential causal factors among adults in southern Brazil]. Arquivos Brasileiros de Endocrinologia e Metabologia 52: 1156-1162.

Veloso HJ, da Silva AA (2010) [Prevalence and factors associated with abdominal obesity and excess weight among adults from Maranhao, Brazil]. Revista Brasileira de Epidemiologia 13: 400-412.

Ver Ploeg M, Mancino L, Lin BH, Guthrie J (2008b) US Food assistance programs and trends in children's weight. International Journal of Pediatric Obesity 3: 22-30.

Versztovsek R (1984) The effect of price increases on the average food consumption and on the nutrition standards of social groups in Hungary. Acta Oeconomica 32: 163-174.

Vilar CM (2010) A study of immigration and obesity among recent Mexican immigrants to the New York city area [Doctoral dissertation]. New York, NY, United States: New York University.

Villard LC, Ryden L, Stahle A, Lindberg LC (2007) Predictors of healthy behaviours in Swedish school children. European Journal of Cardiovascular Prevention and Rehabilitation 14: 366-372.

Vira R, Raudsepp L (2002) Noorukite sotsiaalpsuhholoogiliste determinantide seosed kehalise aktiivsusega seitsmendast kuni kaheksanda klassini [Associations between socio-psychological determinants and physical activity in grade 7 through 8]. Kehakultuuriteaduskonna Teadus- Ja Oppemetoodiliste Toode Kogumik 10: 165-178.

von Tigerstrom B, Larre T, Sauder J (2010) Tax incentives to promote physical activity: Policy analysis of initiatives in Canada. Obesity Reviews 11: 62.

Wandel M (1995) Dietary intake of fruits and vegetables in Norway: Influence of life phase and socio-economic factors. International Journal of Food Sciences and Nutrition 46: 291-301.

Wang Y, Chen C, He W (2000) [Food consumption and dietary pattern in China during 1990-1998]. Wei sheng yan jiu [Journal of Hygiene Research] 29: 288-293.

Wang Z, Zhai F, He Y, Wang H (2008) [Influence of family income on dietary nutrients intake and dietary structure in China]. Wei sheng yan jiu [Journal of Hygiene Research] 37: 62-64.

Ward JO, Almeida AT (1977) Nutricao, renda e tamanho da familia: um exame da situacao nutricional em Caninde, Ceara [Nutrition, income, and family size: an examination of the nutritional situation in Caninde, Ceara]. Revista economica do Nordeste 8: 77-94.

Warren N (2002) Sporting tax. Taxation 149: 680-682.

Weninger Q, Just R (1997) Economic evaluation of food programs: The case of the farmers' market nutrition program. Ames, IA, United States: Iowa State University, Dept. of Economics.

Wilkosz ME (2009) Risk factors contributing to overweight, decreased physical activity and body dissatisfaction in California adolescents [Doctoral dissertation]. San Francisco, CA, United States: University of California at San Francisco.

Witkos M, Uttaburanont M, Lang CD, Arora R (2008) Costs of and reasons for obesity. Journal of the CardioMetabolic Syndrome 3: 173-176.

Wong P, Higuera I, Valencia ME (1984) [Relation between familial income, expenditure and food consumption in marginal urban zones of Sonora, Mexico]. Archivos Latinoamericanos de Nutricion 34: 391-403.

Wu M, Guo ZR, Yang J, Zhou H, Wang PH, et al. (2007) [Study on the risk factors of metabolic syndrome in Jiangsu province, China]. Zhonghua liu xing bing xue za zhi [Chinese Journal of Epidemiology] 28: 536-539.

Wyatt CJ, Triana Tejas MA (2000) Nutrient intake and growth of preschool children from different socioeconomic regions in the city of Oaxaca, Mexico. Annals of Nutrition and Metabolism 44: 14-20.

Xie B, Chou C-P, Spruijt-Metz D, Reynolds K, Clark F, et al. (2007) Socio-demographic and economic correlates of overweight status in Chinese adolescents. American Journal of Health Behavior 31: 339-352.

Yen ST, Fang C, Su S-J (2004b) Household food demand in urban China: A censored system approach. Journal of Comparative Economics 32: 564-585.

Young-Shin L, Levy Susan S (2011) Gender and income associations in physical activity and blood pressure among older adults. Journal of Physical Activity & Health 8: 1-9.

Yu J, Mendell R (1979) Outdoor recreation dimensions and their relationships to socioeconomic variables. Unpublished Paper: 9.

Yu X, Abler D (2009) The demand for food quality in rural China. American Journal of Agricultural Economics 91: 57-69.

Yu ZF (2001) Investigation on the current sports consumption of Beijing residents. Sports Science/Tiyu Kexue 21: 9-11, 21.

Zaini MZ, Lim CT, Low WY, Harun F (2005) Factors affecting nutritional status of Malaysian primary school children. Asia-Pacific Journal of Public Health 17: 71-80.

Zhai F, Lu B, Jin S, Popkin BM (1998) [Impact of maternal income on the nutrients intake of preschool children--a case study in 8 provinces of China]. Wei sheng yan jiu [Journal of hygiene research] 27: 269-272.

Zhao Z (2008) Neighborhood effects on obesity [Doctoral dissertation]. Chicago, IL, United States: University of Illinois at Chicago.

Zheng Y (2008) Economic determinants of obesity among old Americans [Doctoral dissertation]. Santa Monica, CA, United States: Pardee RAND Graduate School.

Zlot AI, Librett J, Buchner D, Schmid T (2006) Environmental, transportation, social, and time barriers to physical activity. Journal of Physical Activity & Health 3: 15-21.
