# Supplementary material for: Economic Instruments for Population Diet and Physical Activity Behaviour Change: A Systematic Scoping Review
Source: PLoS One. 2013 Sep 24;8(9):e75070. doi: 10.1371/journal.pone.0075070 (PMC3782495; doi:10.1371/journal.pone.0075070)
Supplement: Interventions S1 — Defintions of interventions. (DOCX) [file pone.0075070.s003.docx]

**Interventions S1. Definitions of interventions.**

*Price promotions* are a subset of consumer sales promotions that involve reducing the unit retail price of one or more final consumer products or services (e.g. simple discounts, multi-buy deals).

*Taxes* are financial charges or levies imposed by governments or other public legislative authorities, paid by producers, retailers or service providers and/or consumers and held as public funds. Taxes may be paid on income, property or goods and services, among other things.

*Supply-side subsidies* are payments or reimbursements made from public funds to either producers of commodities or intermediate goods (i.e. subsidies paid on inputs), or to retailers or providers of related final consumer products or services (i.e. subsidies paid on products or services) with the intention of stimulating an increase in quantities produced and offered for purchase and a corollary decrease in the unit retail prices. As such, supply-side subsidies change the composition of production and consumption, but have little or no effect on the total quantities of goods or services produced within a whole economy.

*Direct unit pricing legislation* is legislation enacted by government or other legislative authorities that imposes either a minimum or a maximum unit retail price that must be paid by consumers for a category or set of final consumer products or services.

*Transfer payments* are transfers of public or private funds to members of defined population sub-groups (e.g. income transfers, welfare benefits or assistance programs, tax credits). The key distinctions between transfer payments and supply-side subsidies is that the former are typically provided to consumers and are not intended to change relative quantities of products of services produced and offered for consumption.
