# Supplementary material for: Economic Instruments for Population Diet and Physical Activity Behaviour Change: A Systematic Scoping Review
Source: PLoS One. 2013 Sep 24;8(9):e75070. doi: 10.1371/journal.pone.0075070 (PMC3782495; doi:10.1371/journal.pone.0075070)
Supplement: Protocol S1 — Protocol for the systematic scoping review. (DOCX) [file pone.0075070.s004.docx]

**Protocol S1**

**Title**Effects of changes in the economic environment on diet- and physical activity-related behaviours and corollary outcomes: a large-scale scoping review.

**Objective**To assemble, configure and describe empirical research studies of the effects of specific interventions to change, or general exposure to changes in, prices or income on diet- and physical activity-related behaviours and corollary health-related outcomes.

**Methods**

***Provisional criteria for considering studies for this review***Provisional eligibility criteria for considering studies for this review are presented below. These eligibility criteria will be refined iteratively in light of the studies and evidence encountered during the course of the review process.

*Types of studies*: Empirical research studies of any design will be eligible for inclusion. Empirical research studies are defined as studies that involve the analysis of data collected by means of direct or indirect observation or experiment for the purpose of addressing one or more specific research questions. This definition encompasses the following categories of research:

- Primary research (e.g. experimental or quasi-experimental study designs; non-randomised study designs; qualitative study designs; full or partial economic evaluations and budget impact analyses; correlational study designs; mixed-methods study designs);
- Secondary research (e.g. systematic reviews or non-systematic literature reviews that include types of primary study design specified above; decision-analytic models); and
- Tertiary research (e.g. overviews of systematic reviews or non-systematic literature reviews that include types of primary study design specified above).

There will be no restriction of eligible studies based on publication language. We will record bibliographic details of studies that are assessed as potentially eligible (based on initial screening of titles and abstract records) and published in a language other than English. If no corresponding English language full-text can be obtained, the study will not qualify for data collection and analysis. There will be no restrictions of eligible studies based on publication date or publication status.

*Types of interventions and exposures*: Changes in remunerative incentives to consume final consumer products or services may in principle be policy or market driven, but in practice are likely to be determined a combination of market and policy factors. Studies of both policy- changes (i.e. intervention) and market-driven changes (i.e. exposures) in remunerative incentives will be eligible for consideration for this review. Policy driven changes refer to the influence of government or organisation initiated intervention in economic markets for final consumer products or services, encompassing use of both: micro-economic instruments (such as taxes, subsidies, direct (minimum or maximum) unit pricing legislation, or provision of transfer payments (e.g. welfare benefits, tax credits, or income transfer programs); and retail practices, such as consumer sales promotions, or direct unit pricing practices (e.g. minimum, maximum or introductory pricing, where the latter involves retailers setting an artificially low or high initial unit retail price for a new or existing product or service in order to attract consumer purchases). Such interventions have the potential to alter prices and/or income, and hence alter remunerative incentives to consume final consumer products or services. A final product or service is defined as one that is (ultimately) consumed, as opposed to one that is used in the production of another good or service (the latter comprises raw materials and intermediate goods). A consumer product or service is one that is sold for direct consumption by the purchaser (or by the purchaser’s family members, friends, colleagues and/or other associates).

Some provisionally eligible types of interventions may in principle be implemented at any stage in production and distribution channels. However, studies of such interventions will only be considered eligible if the intervention is either applied directly to final consumer products or services, or if a study incorporates evidence that changes in prices or income, levels of consumption of final consumer products or services, other diet- and/or physical activity-related behaviours, and/or corollary health-related outcomes, are attributable to intervention at a preceding point in production and distribution channels. Therefore, with the latter exceptions, micro-economic instruments or business practices that have the potential to change remunerative incentives amongst producers, wholesalers, retailers or service providers will be excluded. For example, studies of agricultural subsidies, which may influence the relative prices of certain commodities used to produce energy-dense foods, will be included if there is evidence for a direct impact of such subsidies on the unit retail prices of (and hence remunerative incentives to consume) energy-dense foods, or on levels of purchasing or dietary intake of energy-dense foods, but will otherwise be excluded.

Interventions that involve provision of personal financial incentives – provisionally defined as any form of reward or penalty with a monetary value, provided directly to individuals contingent on performance of specific behaviours or achievement of specific outcomes – will be excluded. Choice architecture interventions – provisionally defined as interventions that have the potential to change health-related behaviour and are implemented within the same micro-level environment as that in which the targeted behaviour is enacted – will also be excluded, unless the intervention involves, or results in, changes in remunerative incentives to consume one or more final consumer products or services associated with healthy or less healthy diet- or physical activity-related behaviours, or changes in target outcomes, that are directly attributable to changes in monetised or non-monetised prices or changes in disposable income.

With the exceptions of legislative or regulatory provision of taxes, subsidies, direct (minimum or maximum) pricing legislation, or provision of transfer payments, interventions that involve the provision of legislation or regulation with the explicit aim of directly influencing diet- or physical activity-related behaviours, and which include a mechanism for enforcing compliance, will be excluded. Interventions that involve major alterations to the existing physical infrastructure or built environment (e.g. a new supermarket or leisure centre in a local area with no such existing services; a new network of cycle-lanes in an urban area) will be excluded. Finally, there will be no restrictions relating to types of comparators or counterfactuals studied (if applicable).

Market driven changes refer to general variations in prices that may be attributed to the unregulated interaction of supply and demand for final consumer products or services. A standard economic framework assumes that individuals seek to maximize their utility (i.e. happiness or welfare) subject to constraints of budget, time and biology. People weigh the opportunity costs and benefits of different courses of action and then choose the set of actions that maximises their net benefits (i.e. in terms of utility, happiness or welfare) subject to the constraints they face. Standard economic theory therefore predicts that, other things being equal (and given a choice between two final consumer products that perform essentially the same function), exposure to an increase in the relative price of the less healthy product, compared with a healthy product, would result in increased levels of consumption of the healthy product and reduced levels of consumption of the unhealthy product.

Price may have a monetised and/or non-monetised component. Monetised unit price can be conceptualised as a measure of the market value of a single, specified unit of a given product or service (where market value may be influenced by various forms of supply- and/or demand-side intervention into markets, alongside the (unregulated) interaction of supply and demand). In this context, unit retail price is defined as the price paid by the purchaser for a single, specified unit of a final consumer product or service. Opportunity cost is defined as the value of foregone benefits associated with the next best alternative use of a specified set of resources. A change in the unit retail price of one or more final consumer products or services reflects a change in the opportunity costs of consumption. Changes in the opportunity costs of consumption will alter remunerative incentives to consume associated with that product or service, and also remunerative incentives to consume substitute or complementary products or services (and vice versa). For example, an increase in the unit retail price of butter relative to the unit retail price margarine (the latter is a close substitute for butter) would in theory alter the opportunity costs of consumption of both products, with a decreased remunerative incentive to consume butter (and a corollary increased remunerative incentive to consume margarine), leading to reduced average levels of consumption of butter (and increased average levels of consumption of margarine).

Alongside (monetised) prices, other prices (or costs) that are paid or (incurred) but not monetised (i.e. ‘non-monetised prices or costs’), such as ‘time’ or ‘effort’, also have the potential to influence the opportunity costs of consumption of final consumer products or services. Determinants of non-monetised prices or costs may include the accessibility or availability of retail outlets (e.g. the distance from an individual’s home or workplace to the nearest retail food outlet) and access to transportation (e.g. whether an individual must walk or cycle to the nearest retail food outlet, or has the option of making this journey by public transport, or using a car). Determinants of non-monetised prices or costs may in turn be influenced by market forces and/or government or organisational policies relating to, for example, public transport infrastructure or other aspects of the built environment.

A standard economic framework assumes that people decide what and how much final consumer products, goods and services to consume, what and how much to eat, and what and how much physical activity to perform subject to budget constraint, in terms of (disposable) income. Changes in individual or household total (disposable) income will shift this budget constraint, with associated changes in (average) levels of consumption of all final consumer products or services. For example, an increase in individual or household (disposable) income loosens the budget constraint faced by that individual or household, with predicted increases in remunerative incentives to consume and hence levels of consumption of all final consumer products and services, other things being equal. Determinants of individual and/or household disposable income include socio-economic status, occupation, hours of paid work and receipt of welfare benefits, which may in turn be influenced by both (unregulated) market forces (e.g. labour market demand and supply) and public policy (e.g. fiscal, social welfare, education and/or child and family policy). Additionally, price increases (or decreases) will reduce (or increase) the purchasing power of income, with associated changes in remunerative incentives to consume and hence (average) levels of consumption.

Therefore, alongside studies of the use of economic instruments to alter prices or income, we will also include studies of general exposure to variations in:

1. unit retail prices of final consumer products or services associated with healthy or less healthy physical activity- or diet-related behaviours;
2. unit retail prices of substitutes for, or complements to, final consumer products or services associated with healthy or less healthy physical activity- or diet-related behaviours;
3. unit prices of raw materials or intermediate products used in the production of final consumer products or services associated with healthy or less healthy physical activity- or diet-related behaviours, or their substitutes or complements;
4. non-monetised prices/costs of consumption of final consumer products or services associated with healthy or less healthy physical activity- or diet-related behaviours;
5. non-monetised prices/costs of consumption of substitutes for, or complements to, final consumer products or services associated with healthy or less healthy physical activity- or diet-related behaviours;
6. levels of individual or household (disposable) income; and/or
7. determinants of individual and/or household (disposable) income.

Studies of exposures ‘b’ and ‘c’ will only be eligible if they provide evidence that unit retail prices of substitutes or complements (‘b’), or unit prices of raw materials or intermediate products or services (‘c’), moderate, or are moderated by, exposure ‘a’ (i.e. variations in the unit retail prices of final consumer products or services associated with healthy or less healthy physical activity- or diet-related behaviours). Similarly, studies of exposure ‘g’ will only be recorded if the study provides evidence that other determinants of individual and/or household (disposable) income moderate, or are moderated by, exposure ‘f’ (i.e. variations in levels of individual or household (disposable) income).

*Types of outcomes*: The objective of this scoping review is concerned with evidence for the effects of policy- and/or market-driven changes in prices or income on diet- and physical activity-related behaviours. Provisional operational definitions and typologies of diet-related and physical activity-related behaviours are as follows:

Diet-related behaviours are defined as (in principle) modifiable actions of human individuals or groups that affect intake of healthy or less healthy foods or non-alcoholic beverages. Dietary intake is defined as the amounts and types of foods or non-alcoholic beverages consumed (i.e. by eating or drinking) and/or their nutritional profiles. A provisional typology of diet-related behaviours is presented below:

- *Purchasing behaviours* (e.g. specific types of food and drink; amounts purchased (volume; portion size))
- *Shopping behaviours* (e.g. frequency; type of retail outlet - corner shop, market, supermarket, online; mode of transport)
- *Selection behaviours* - non-purchased items (e.g. specific types of food and drink; amounts selected (portion size); amounts discarded)
- *Cooking and food preparation behaviours* (e.g. steam or boil vegetables; grill or fry meats; add salt or not)
- *Mealtime behaviours* (e.g. frequency; timing; number of courses within meals; specific types of food and drink; amounts plated (portion size); amounts discarded)
- *Snacking behaviours* (e.g. frequency; timing; specific types of food and drink; amounts selected (portion size); amounts discarded)
- *Social behaviours* (e.g. social setting/context for meals and snacks - home, work, other’s home, event, on the move, alone or with partner/ children/ peers; social setting – home, restaurant, work canteen; dining table, sofa, desk, other work site)
- *Controlling behaviours* (e.g. dieting or not-dieting; type of diet; restricting)
- *Modelling behaviours* - parents or other adults to children (e.g. encouragement; rules; facilitation; education)
- *Sleep behaviours* (e.g. amount; timing in relation to meals and snacks)
- *Work behaviours* (e.g. amount - hours worked, paid or unpaid; shift patterns; timing in relation to meals and snacks)

Physical activity is defined as actions of human individuals or groups that involve bodily movement produced by skeletal muscles requiring energy expenditure. Health-enhancing physical activity (HEPA) is defined as any form of physical activity that benefits health and functional capacity without undue harm or risk. The focus of this scoping review is on physical activity, including (but not limited to) HEPA.

The terms physical activity and exercise are not synonymous. Exercise is a subcategory of HEPA that is planned, structured, repetitive, and purposeful in the sense that the improvement or maintenance of one or more components of physical fitness is the objective. Physical activity includes exercise as well as other activities which involve bodily movement and are done as part of working, transportation, household chores or recreational activities. It may be performed in various spatial-temporal domains, including: leisure-time activity, occupational activity, household activity, and commuting (transport) activity.

Physical activity-related behaviours are defined as behaviours that involve or influence (by enabling, inhibiting or substituting) the performance of HEPA. This definition encompasses performance of ‘active’, ‘light-intensity’ and ‘sedentary’ physical activities, which may be characterised by their intensity, frequency and or duration. From a biological perspective, sedentary physical activities are defined as those in which energy expenditure levels remain close or equivalent to the resting metabolic rate, whilst active physical activities are defined as those performed at a moderate or vigorous intensity: moderate (on an absolute scale, performed at 3.0–5.9 times the intensity of rest; on a scale relative to an individual’s personal capacity, usually a 5 or 6 on a scale of 0–10) or vigorous (on an absolute scale, performed at 6.0 or more times the intensity of rest for adults and typically 7.0 or more times for children and youth; on a scale relative to an individual’s personal capacity usually a 7 or 8 on a scale of 0–10). HEPA usually involves physical activity performed at this intensity. Light-intensity physical activities are defined as those performed at intensity levels between those of sedentary and active physical activities, encompassing activities of daily living such as standing, walking slowly or lifting lightweight objects. HEPA may further be classified by type: aerobic (e.g. walking, running, and swimming, and bicycling); strength (bone-strengthening, e.g. running, jumping rope, lifting weights; muscle-strengthening, e.g. strength training, resistance training, or muscular strength and endurance exercises); flexibility; or balance. A provisional typology of physical activity-related behaviours is presented below:

- Performance behaviours
  - Exercise behaviours
  - Work behaviours
  - Transport behaviours
  - Domestic behaviours
  - Recreational behaviours
- Purchasing behaviours
- Selection behaviours
- Social behaviours
- Modelling behaviours
- Sleep behaviours

By implication, the primary focus of this scoping review is on studies that provide evidence for the effects of such changes in terms of outcomes relating to:

- Levels and patterns of consumption of final consumer products or services (i.e. purchase and shopping behaviours) associated with healthy or less health diet- and/or physical activity-related behaviours (i.e. products or services whose consumption is recognised as a determinant of other healthy or less healthy physical activity- and/or diet-related behaviours and hence proximal outcomes of such behaviours - namely dietary intake and/or energy expenditure); and
- Levels and patterns of performance of other healthy or less healthy diet- and/or physical activity-related behaviours (e.g. mealtime behaviours, snacking behaviours, performance of physical activity).

However, given complex proposed causal pathways towards final health outcomes, we will also include studies that measure the effects of market and/or policy driven changes in prices and/or income in terms of outcomes relating to:

- Proximal consequences of physical activity- and/or diet-related behaviours, namely dietary intake of healthy or less healthy foods or non-alcoholic beverages (primarily the calorific and nutritional profiles of such intake), and/or levels of energy expenditure; and
- Distal (intermediate) consequences of diet- and/or physical activity-related behaviours, namely major, modifiable metabolic or physiological risk factors for NCDs: overweight and obesity, (raised) blood pressure, (raised) blood glucose and/or (raised) cholesterol.

In summary, we will therefore include studies that provide evidence for the effects of market and/or policy driven changes in prices or income in terms of one or more of the following types of outcome measures:

1. Levels and/or patterns of consumption of final consumer products or services associated with diet- and/or physical activity-related behaviours (i.e. purchasing or shopping behaviours);
2. Levels and/or patterns of consumption of substitutes for, or complements to, final consumer products or services associated with diet- and/or physical activity-related behaviours (i.e. purchasing or shopping behaviours);
3. Levels and/or patterns of performance of other diet- and/or physical activity-related behaviours;
4. Levels (types, amounts and/or nutritional characteristics) of intake of healthy or less healthy foods or non-alcoholic beverages;
5. Levels of energy expenditure;
6. Changes in major modifiable risk factors for NCDs: body weight, overweight and obesity, blood pressure, blood glucose and blood cholesterol.
7. Adverse or unintended effects relating to outcome categories 1-6, above.
8. Distributional effects relating to outcome categories 1-6, above.
9. Acceptability of interventions.
10. Feasibility of interventions.
11. Fidelity or adherence in the implementation of interventions.
12. Costs (resource use) associated with the implementation of interventions and/or their (intermediate or final) outcomes (categories 1-6, above).
13. Cost-effectiveness (i.e. relative efficiency) of interventions.
14. Budget impact of interventions.
15. Sustainability of interventions.

Eligible studies will measure one or more of the above types of outcomes, with the following exceptions:

- Studies measuring outcomes within ‘Category 2’ will only be included if they provide evidence that levels of consumption of substitutes or complements moderate, or are moderated by, outcomes within ‘Category 1’ (i.e. levels of consumption of final consumer products or services associated with diet- and/or physical activity-related behaviours).
- With respect to outcomes within ‘Category 3’, studies measuring outcomes relating to ‘sleep behaviours’ and/or ‘work behaviours’, will only be included if they provide evidence for the mediating impact of such behaviours on outcomes within ‘Category 4’ or ‘Category 5’ (i.e. levels of intake of healthy or less healthy foods or non-alcoholic beverages; energy expenditure).

***Search methods for identification of studies***

*Electronic searches*
The following electronic literature databases will be searched with no date or publication language restrictions.

- MEDLINE (Ovid SP)
- EMBASE (Ovid SP)
- PsycINFO (Ovid SP)
- EconLit (EBSCO)
- SPORTDiscus with Full Text (EBSCO)
- Applied Social Sciences Index and Abstracts (CSA Illumina)
- Cochrane Database of Systematic Reviews (Wiley Online Library)
- Database of Abstracts of Reviews of Effects (Wiley Online Library)
- Health Technology Assessment Database (Wiley Online Library)
- NHS Economic Evaluation Database (Wiley Online Library)
- Database of Promoting Health Effectiveness Reviews (EPPI Centre)

We will develop electronic search strategies for each of these electronic literature databases based on provisional eligibility criteria specified above.

*Snowball searches*Snowball search techniques (i.e. searching reference lists of published reports within the emerging corpus of eligible studies and electronic citation tracking from published reports within the emerging corpus of eligible studies, using PubMed and Google Scholar, to identify published reports that have subsequently cited eligible studies) will be used in conjunction with electronic searches to identify further potentially eligible studies. Snowball searches will encompass grey literature and other electronic resources.

***Data collection and analysis***

*Selection of studies*Data collection and analysis will be managed using EPPI-Reviewer 4 systematic review software. Retrieved records sets (titles and abstract records) will be imported into EPPI-Reviewer 4 (via RIS import filters from Endnote X4) and duplicates will be removed using EPPI-Reviewer 4’s integrated de-duplication software based on fuzzy logic.

Initial screening of titles and abstracts will be led by one reviewer, with support from a second reviewer as required. Initial screening will be facilitated by use of EPPI-Reviewer 4’s integrated suite of text mining technologies to prioritise title and abstract records for manual screening: ‘automatic term recognition’ (this identifies key terms from a body of text); ‘automatic document classification’ (this ‘learns’ how to apply provisional ‘include’ and ‘exclude’ (or other user-defined) codes based on training material); and ‘reviewer terms’ (this relies on reviewers’ personal experiences and conceptualisations or impressions of the screening process to identify terms reckoned to be indicative of includes (but not excludes) and those reckoned to be indicative of an exclude (but not an include)). Each of these text mining technologies will be used alone, or in combination (‘hybrids’, in which one text-mining technology is used as a counterpoint to another). The sequence in which text mining technologies will be used, alone or in combination (‘hybrids’), will be determined iteratively, based on prospective monitoring of text mining performance.

It is expected ex ante that the study selection process will both identify and help to clarify ‘boundary issues’ relating to: provisional operational definitions of key concepts, taxonomies of diet- and physical activity-related behaviours; and determinants or consequences of such behaviours (see Figures S1 and S2); and, consequently, to pre-specified, provisional eligibility criteria. Such ‘boundary issues’ will be discussed and resolved in consultation with the BHRU scoping reviews core project team, with reference to specific exemplar (provisionally) included and/or excluded studies. Implications for operational definitions of key concepts, taxonomies of diet- and physical activity-related behaviours, and determinants or consequences of such behaviours will be agreed by consensus and refined. This emergent knowledge of encountered studies and evidence will also be used to iteratively refine provisional eligibility criteria. Provisional eligibility decisions will be reviewed against final eligibility criteria and revised as necessary.

Corresponding full-texts of provisionally eligible study reports will be obtained via electronic library resources. Full-text screening will be performed by one reviewer, with support from a second reviewer as required. Further duplicates will identified manually and removed. The primary reason for exclusion of a study report at the full-text screening stage will be recorded. Full-text reports located using snowball searches will also be assessed against final eligibility criteria. Duplicate reports between those identified using systematic searches with text mining and those located using snowball searches will be identified manually and removed. Multiple reports of the same study will be linked into a single study. Details of the flow of information (i.e. records, study reports and studies) through different stages of the scoping review process will be recorded and reported using a four-phase PRISMA flow diagram.

*Data collection and management*
Data collection will be limited to data extraction from published records and reports of included studies (i.e. there will be no follow-up contacts with study authors to request data that is missing from published reports). Data collection will be led by one reviewer. Data will be extracted from included studies using a pre-specified data extraction form, to be developed. Collected data will include selected study characteristics and verbatim transcription of authors’ relevant principal results and study conclusions.

*Data analysis*
Collected data will be analysed and presented using: frequency and crosstabs reports of categorical, numeric and textual data; tabular summaries and summary descriptive statistics of numeric data; clustered textual descriptions of the characteristics, methods, principal results and conclusions of included studies, drawing on narrative synthesis approaches (i.e. clustered by study characteristics, for example: types of interventions; types of study designs; types of outcomes assessed).
