# Supplementary material for: Economic Instruments for Population Diet and Physical Activity Behaviour Change: A Systematic Scoping Review
Source: PLoS One. 2013 Sep 24;8(9):e75070. doi: 10.1371/journal.pone.0075070 (PMC3782495; doi:10.1371/journal.pone.0075070)
Supplement: Search Strategies S1 — Search strategies used in electronic searches. (DOCX) [file pone.0075070.s005.docx]

**Search Strategies S1**

**MEDLINE (Ovid SP). 1948 to June Wk 5 2011.**

1. exp diet/

2. exp diet therapy/

3. exp food/

4. exp beverages/

5. food habits/

6. food preferences/

7. fasting/

8. adolescent nutritional physiological phenomena/

9. elder nutritional physiological phenomena/

10. exp food industry/

11. exp hunger/

12. exp appetite regulation/

13. exp appetite/

14. exp digestion/

15. exp eating/

16. exp eating disorders/

17. exp child nutrition disorders/

18. exp infant nutrition disorders/

19. nutritional requirements/

20. nutritional status/

21. nutrition assessment/

22. nutrition disorders/

23. exp nutritive value/

24. (nutri$ or calori$ or diet$ or food$ or eat$ or meal$ or snack$ or cook$ or restaurant$ or supermarket$ or cafe$).ti,ab.

25. ((drink$ or beverage$) not alcohol$).ti,ab.

26. or/1-25

27. physical exertion/

28. exp human activities/

29. exp leisure activities/

30. exp locomotion/

31. exp physical education/

32. lifestyle/

33. sedentary lifestyle/

34. yoga/

35. fitness centers/

36. motor activity/

37. (physical$ adj5 (exercis$ or train$ or activit$ or fit$ or endur$)).ti,ab.

38. (aerobic adj5 (exercis$ or train$ or activit$ or fit$ or endur$)).ti,ab.

39. (strength$ adj5 (exercis$ or train$ or activit$ or fit$ or endur$)).ti,ab.

40. (flexib$ adj5 (exercis$ or train$ or activit$ or fit$ or endur$)).ti,ab.

41. (balanc$ adj5 (exercis$ or train$ or activit$ or fit$ or endur$)).ti,ab.

42. (exercise$ adj5 (train$ or activit$ or fit$ or endur$)).ti,ab.

43. ((occupation$ or work$ or recreation$2 or leisure or play or household or home or domestic or commut$3 or transport$) adj5 (energ$ or exercis$ or train$ or activit$ or fit$ or endur$)).ti,ab.

44. ((walk$3 or hike or hiking or climbing or run$3 or jog$3 or swim$1 or swimming or bicycl$3 or cycl$3 or bike$1 or biking or gym$ or rowing or canoe$ or kayak$ or sailing or windsurf$3 or surf$3 or diving or sport$3 or rollerblading or rollerskating or skating or skiing or yoga or pilates or calisthenics or (jump$3 adj rope$1) or (lift$3 adj weight$1) or circuit or resistance or resilience or dance or dancing or fishing or hunting or shooting) adj5 (energ$ or exercis$ or train$ or activit$ or fit$ or endur$)).ti,ab.

45. (led walk$ or health walk$).ti,ab.

46. ((leisure or fitness) adj5 (centre$ or center$ or facilit$)).ti,ab.

47. (fitness adj class$).ti,ab.

48. (fitness adj (regime$ or program$)).ti,ab.

49. cardiorespiratory fitness.ti,ab.

50. aerobic capacity.ti,ab.

51. (intensity adj2 (rest or quiet or light or moderate or vigorous)).ti,ab.

52. ((car or cars or bus or buses or train or trains or transport$) and (energ$ or activit$ or exercis$)).ti,ab.

53. (active adj (travel$4 or transport$ or commut$)).tw.

54. ((promot$ or uptak$ or encourag$ or increas$ or start$ or adher$ or sustain$ or maintain$) adj5 gym$).ti,ab.

55. ((promot$ or uptak$ or encourag$ or increas$ or start$ or adher$ or sustain$ or maintain$) adj5 physical activit$).ti,ab.

56. ((promot$ or uptak$ or encourag$ or increas$ or start$ or adher$ or sustain$ or maintain$) adj5 (circuit$ or aqua$)).ti,ab.

57. ((promot$ or uptak$ or encourag$ or increas$ or start$ or adher$ or sustain$ or maintain$) adj5 (exercis$ or exertion or keep fit or fitness class or yoga or aerobic$)).ti,ab.

58. ((decreas$ or reduc$ or discourag$) adj5 (sedentary or deskbound or inactiv$)).ti,ab.

59. (exercis$ adj aerobic$).tw.

60. (physical$ adj5 (fit$ or train$ or activ$ or endur$)).tw.

61. (exercis$ adj5 (train$ or physical$ or activ$)).tw.

62. ((lifestyle or life-style) adj5 physical$).tw.

63. ((lifestyle or life-style) adj5 activ$).tw.

64. or/27-63

65. 26 or 64

66. (risk$ adj4 (non-communicable or non communicable or chronic)).ti,ab.

67. blood pressure/

68. hypertension/

69. blood glucose/

70. hyperglycemia/

71. cholesterol/

72. cholesterol, dietary/

73. cholesterol, hdl/

74. cholesterol, ldl/

75. cholesterol, vldl/

76. cholesterol esters/

77. hypercholesteremia/

78. exp hyperlipidemias/

79. exp body weight changes/

80. harm reduction/

81. exp overnutrition/

82. exp overweight/

83. exp obesity/

84. (overweight or over weight).ti,ab.

85. adipos$.ti,ab.

86. fat overload syndrome$.ti,ab.

87. (overeat or over eat).ti,ab.

88. weight cycling.ti,ab.

89. weight reduc$.ti,ab.

90. weight losing.ti,ab.

91. weight maint$.ti,ab.

92. weight decreas$.ti,ab.

93. weight watch$.ti,ab.

94. weight control$.ti,ab.

95. weight gain.ti,ab.

96. weight loss.ti,ab.

97. weight chang$.ti,ab.

98. (bmi or obes$ or overweight or (blood adj pressure) or hypertensi$ or (blood adj glucose) or hyperglyc?mi$ or cholester$ or hypercholester$ or hyperlipid?emia$).ti,ab.

99. or/66-98

100. (economic$ or financ$ or cost or costs or costing or pric$ or monetis$ or income$ or wage$ or salar$ or (expenditure$ not energy) or time$).ti,ab.

101. (tax$ or subsid$ or credit$ or (((cash or income) adj2 transfer) or payment) or (welfare adj benefit$) or incentiv$ or disincentiv$ or remunerat$ or retail$ or sale$ or promo$ or consumer$ or consumption$ or purchas$ or shop$ or buy$).ti,ab.

102. ((product or good or service or market) adj (innovat$ or develop$ or efficien$ or quality)).ti,ab.

103. or/100-102

104. 65 and 103

105. 99 and 103

106. 104 or 105

107. animals/

108. humans/ and animals/

109. 107 not 108

110. 106 not 109

**EMBASE (Ovid SP). 1980 to 2011 Wk 27.**

1. exp diet/

2. exp diet therapy/

3. exp food/

4. exp beverage/ not alcoholic beverage/

5. exp feeding behavior/

6. exp dietary intake/ not alcohol consumption/

7. exp food intake/

8. exp child nutrition/

9. exp food handling/

10. exp food processing/

11. exp hunger/

12. exp digestion/

13. exp eating disorder/

14. exp nutritional disorder/

15. nutritional requirement/

16. nutritional status/

17. nutritional assessment/

18. nutritional value/

19. (nutri$ or calori$ or diet$ or food$ or eat$ or meal$ or snack$ or cook$ or restaurant$ or supermarket$ or cafe$).ti,ab.

20. ((drink$ or beverage$) not alcohol$).ti,ab.

21. or/1-20

22. exp exercise/

23. exp physical activity/

24. exp human activities/

25. exp recreation/

26. exp leisure/

27. exp locomotion/

28. exp physical education/

29. exp lifestyle/

30. sedentary lifestyle/

31. exp fitness/

32. health center/

33. yoga/

34. motor activity/

35. (physical$ adj5 (exercis$ or train$ or activit$ or fit$ or endur$)).ti,ab.

36. (aerobic adj5 (exercis$ or train$ or activit$ or fit$ or endur$)).ti,ab.

37. (strength$ adj5 (exercis$ or train$ or activit$ or fit$ or endur$)).ti,ab.

38. (flexib$ adj5 (exercis$ or train$ or activit$ or fit$ or endur$)).ti,ab.

39. (balanc$ adj5 (exercis$ or train$ or activit$ or fit$ or endur$)).ti,ab.

40. (exercise$ adj5 (train$ or activit$ or fit$ or endur$)).ti,ab.

41. ((occupation$ or work$ or recreation$2 or leisure or play or household or home or domestic or commut$3 or transport$) adj5 (energ$ or exercis$ or train$ or activit$ or fit$ or endur$)).ti,ab.

42. ((walk$3 or hike or hiking or climbing or run$3 or jog$3 or swim$1 or swimming or bicycl$3 or cycl$3 or bike$1 or biking or gym$ or rowing or canoe$ or kayak$ or sailing or windsurf$3 or surf$3 or diving or sport$3 or rollerblading or rollerskating or skating or skiing or yoga or pilates or calisthenics or (jump$3 adj rope$1) or (lift$3 adj weight$1) or circuit or resistance or resilience or dance or dancing or fishing or hunting or shooting) adj5 (energ$ or exercis$ or train$ or activit$ or fit$ or endur$)).ti,ab.

43. (led walk$ or health walk$).ti,ab.

44. ((leisure or fitness) adj5 (centre$ or center$ or facilit$)).ti,ab.

45. (fitness adj class$).ti,ab.

46. (fitness adj (regime$ or program$)).ti,ab.

47. cardiorespiratory fitness.ti,ab.

48. aerobic capacity.ti,ab.

49. (intensity adj2 (rest or quiet or light or moderate or vigorous)).ti,ab.

50. ((car or cars or bus or buses or train or trains or transport$) and (energ$ or activit$ or exercis$)).ti,ab.

51. (active adj (travel$4 or transport$ or commut$)).tw.

52. ((promot$ or uptak$ or encourag$ or increas$ or start$ or adher$ or sustain$ or maintain$) adj5 gym$).ti,ab.

53. ((promot$ or uptak$ or encourag$ or increas$ or start$ or adher$ or sustain$ or maintain$) adj5 physical activit$).ti,ab.

54. ((promot$ or uptak$ or encourag$ or increas$ or start$ or adher$ or sustain$ or maintain$) adj5 (circuit$ or aqua$)).ti,ab.

55. ((promot$ or uptak$ or encourag$ or increas$ or start$ or adher$ or sustain$ or maintain$) adj5 (exercis$ or exertion or keep fit or fitness class or yoga or aerobic$)).ti,ab.

56. ((decreas$ or reduc$ or discourag$) adj5 (sedentary or deskbound or inactiv$)).ti,ab.

57. (exercis$ adj aerobic$).tw.

58. (physical$ adj5 (fit$ or train$ or activ$ or endur$)).tw.

59. (exercis$ adj5 (train$ or physical$ or activ$)).tw.

60. ((lifestyle or life-style) adj5 physical$).tw.

61. ((lifestyle or life-style) adj5 activ$).tw.

62. or/22-61

63. 21 or 62

64. (risk$ adj4 (non-communicable or non communicable or chronic)).ti,ab.

65. blood pressure/

66. hypertension/

67. glucose blood level/

68. hyperglycemia/

69. cholesterol/

70. cholesterol intake/

71. high density lipoprotein cholesterol/

72. low density lipoprotein cholesterol/

73. very low density lipoprotein cholesterol/

74. hypercholesterolemia/

75. exp hyperlipidemia/

76. weight change/

77. harm reduction/

78. exp overnutrition/

79. exp obesity/

80. (overweight or over weight).ti,ab.

81. adipos$.ti,ab.

82. fat overload syndrome$.ti,ab.

83. (overeat or over eat).ti,ab.

84. weight cycling.ti,ab.

85. weight reduc$.ti,ab.

86. weight losing.ti,ab.

87. weight maint$.ti,ab.

88. weight decreas$.ti,ab.

89. weight watch$.ti,ab.

90. weight control$.ti,ab.

91. weight gain.ti,ab.

92. weight loss.ti,ab.

93. weight chang$.ti,ab.

94. (bmi or obes$ or overweight or (blood adj pressure) or hypertensi$ or (blood adj glucose) or hyperglyc?mi$ or cholester$ or hypercholester$ or hyperlipid?emia$).ti,ab.

95. or/64-94

96. (economic$ or financ$ or cost or costs or costing or pric$ or monetis$ or income$ or wage$ or salar$ or (expenditure$ not energy) or time$).ti,ab.

97. (tax$ or subsid$ or credit$ or (((cash or income) adj2 transfer) or payment) or (welfare adj benefit$) or incentiv$ or disincentiv$ or remunerat$ or retail$ or sale$ or promo$ or consumer$ or consumption$ or purchas$ or shop$ or buy$).ti,ab.

98. ((product or good or service or market) adj (innovat$ or develop$ or efficien$ or quality)).ti,ab.

99. or/96-98

100. 63 and 99

101. 95 and 99

102. 100 or 101

103. animal/

104. human/ and animal/

105. 103 not 104

106. 102 not 105

**PsycINFO (Ovid SP). 1806 to July WK2 2011.**

1. exp diets/

2. exp food/

3. "beverages (nonalcoholic)"/

4. food preferences/

5. food intake/

6. eating behavior/

7. drinking behavior/

8. nutrition/

9. exp appetite/

10. digestion/

11. dietary restraint/

12. binge eating/

13. eating attitudes/

14. "rumination (eating)"/

15. satiation/

16. exp nutritional deficiencies/

17. exp eating disorders/

18. dietary supplements/

19. (nutri$ or calori$ or diet$ or food$ or eat$ or meal$ or snack$ or cook$ or restaurant$ or supermarket$ or cafe$).ti,ab.

20. ((drink$ or beverage$) not alcohol$).ti,ab.

21. or/1-20

22. exp physical activity/

23. physical health/

24. physical education/

25. physical fitness/

26. physical endurance/

27. physical strength/

28. physical agility/

29. physical dexterity/

30. leisure time/

31. exp recreation/

32. exp lifestyle/

33. locomotion/

34. exp motor processes/

35. "activities of daily living"/

36. daily activities/

37. exp sports/

38. sports medicine/

39. athletic performance/

40. exp athletes/

41. athletic training/

42. energy expenditure/

43. (physical$ adj5 (exercis$ or train$ or activit$ or fit$ or endur$)).ti,ab.

44. (aerobic adj5 (exercis$ or train$ or activit$ or fit$ or endur$)).ti,ab.

45. (strength$ adj5 (exercis$ or train$ or activit$ or fit$ or endur$)).ti,ab.

46. (flexib$ adj5 (exercis$ or train$ or activit$ or fit$ or endur$)).ti,ab.

47. (balanc$ adj5 (exercis$ or train$ or activit$ or fit$ or endur$)).ti,ab.

48. (exercise$ adj5 (train$ or activit$ or fit$ or endur$)).ti,ab.

49. ((occupation$ or work$ or recreation$2 or leisure or play or household or home or domestic or commut$3 or transport$) adj5 (energ$ or exercis$ or train$ or activit$ or fit$ or endur$)).ti,ab.

50. ((walk$3 or hike or hiking or climbing or run$3 or jog$3 or swim$1 or swimming or bicycl$3 or cycl$3 or bike$1 or biking or gym$ or rowing or canoe$ or kayak$ or sailing or windsurf$3 or surf$3 or diving or sport$3 or rollerblading or rollerskating or skating or skiing or yoga or pilates or calisthenics or (jump$3 adj rope$1) or (lift$3 adj weight$1) or circuit or resistance or resilience or dance or dancing or fishing or hunting or shooting) adj5 (energ$ or exercis$ or train$ or activit$ or fit$ or endur$)).ti,ab.

51. (led walk$ or health walk$).ti,ab.

52. ((leisure or fitness) adj5 (centre$ or center$ or facilit$)).ti,ab.

53. (fitness adj class$).ti,ab.

54. (fitness adj (regime$ or program$)).ti,ab.

55. cardiorespiratory fitness.ti,ab.

56. aerobic capacity.ti,ab.

57. (intensity adj2 (rest or quiet or light or moderate or vigorous)).ti,ab.

58. ((car or cars or bus or buses or train or trains or transport$) and (energ$ or activit$ or exercis$)).ti,ab.

59. (active adj (travel$4 or transport$ or commut$)).tw.

60. ((promot$ or uptak$ or encourag$ or increas$ or start$ or adher$ or sustain$ or maintain$) adj5 gym$).ti,ab.

61. ((promot$ or uptak$ or encourag$ or increas$ or start$ or adher$ or sustain$ or maintain$) adj5 physical activit$).ti,ab.

62. ((promot$ or uptak$ or encourag$ or increas$ or start$ or adher$ or sustain$ or maintain$) adj5 (circuit$ or aqua$)).ti,ab.

63. ((promot$ or uptak$ or encourag$ or increas$ or start$ or adher$ or sustain$ or maintain$) adj5 (exercis$ or exertion or keep fit or fitness class or yoga or aerobic$)).ti,ab.

64. ((decreas$ or reduc$ or discourag$) adj5 (sedentary or deskbound or inactiv$)).ti,ab.

65. (exercis$ adj aerobic$).tw.

66. (physical$ adj5 (fit$ or train$ or activ$ or endur$)).tw.

67. (exercis$ adj5 (train$ or physical$ or activ$)).tw.

68. ((lifestyle or life-style) adj5 physical$).tw.

69. ((lifestyle or life-style) adj5 activ$).tw.

70. or/22-69

71. 21 or 70

72. (risk$ adj4 (non-communicable or non communicable or chronic)).ti,ab.

73. exp blood pressure/

74. exp hypertension/

75. blood sugar/

76. hyperglycemia/

77. cholesterol/

78. exp overweight/

79. weight gain/

80. weight loss/

81. "obesity (attitudes toward)"/

82. weight control/

83. body fat/

84. body mass index/

85. lipid metabolism/

86. harm reduction/

87. (overweight or over weight).ti,ab.

88. adipos$.ti,ab.

89. (overeat or over eat).ti,ab.

90. weight cycling.ti,ab.

91. weight reduc$.ti,ab.

92. weight losing.ti,ab.

93. weight maint$.ti,ab.

94. weight decreas$.ti,ab.

95. weight watch$.ti,ab.

96. weight control$.ti,ab.

97. weight gain.ti,ab.

98. weight loss.ti,ab.

99. weight chang$.ti,ab.

100. (bmi or obes$ or overweight or (blood adj pressure) or hypertensi$ or (blood adj glucose) or hyperglyc?mi$ or cholester$ or hypercholester$ or hyperlipid?emia$).ti,ab.

101. or/72-100

102. (economic$ or financ$ or cost or costs or costing or pric$ or monetis$ or income$ or wage$ or salar$ or (expenditure$ not energy) or time$).ti,ab.

103. (tax$ or subsid$ or credit$ or (((cash or income) adj2 transfer) or payment) or (welfare adj benefit$) or incentiv$ or disincentiv$ or remunerat$ or retail$ or sale$ or promo$ or consumer$ or consumption$ or purchas$ or shop$ or buy$).ti,ab.

104. ((product or good or service or market) adj (innovat$ or develop$ or efficien$ or quality)).ti,ab.

105. or/102-104

106. 71 and 105

107. 101 and 105

108. 106 or 107

109. exp animals/

110. 108 not 109

**EconLit (EBSCO). 1886 to July 2011.**

(TI ( (nutri* or calori* or diet* or food* or eat* or meal* or snack* or cook* or restaurant* or supermarket* or cafe*) ) or AB ( (nutri* or calori* or diet* or food* or eat* or meal* or snack* or cook* or restaurant* or supermarket* or cafe*) ) or TI ( ((drink* or beverage*) not alcohol*) ) or AB ( ((drink* or beverage*) not alcohol*) ) or TI ( (exercise* or train* or activit* or fit* or endur* or exertion or aerobic or strength* or flexib* or balance* or recreation* or leisure or play or energ* or walk* or hike or hiking or climbing or run* or jog* or swim* or aqua* or bicycl* or cycl* or bike* or biking or gym* or rowing or canoe* or kayak* or sailing or windsurf* or surf* or diving or sport* or rollerblading or rollerskating or skating or skiing or yoga or pilates or calisthenics or jump* or lift* or circuit or resistance or resilience or dance or dancing or fishing or hunting or shooting or cardiorespiratory or sedentary or deskbound or inactiv*) ) or AB ( (exercise* or train* or activit* or fit* or endur* or exertion or aerobic or strength* or flexib* or balance* or recreation* or leisure or play or energ* or walk* or hike or hiking or climbing or run* or jog* or swim* or aqua* or bicycl* or cycl* or bike* or biking or gym* or rowing or canoe* or kayak* or sailing or windsurf* or surf* or diving or sport* or rollerblading or rollerskating or skating or skiing or yoga or pilates or calisthenics or jump* or lift* or circuit or resistance or resilience or dance or dancing or fishing or hunting or shooting or cardiorespiratory or sedentary or deskbound or inactiv*) ) or TI ( (non-communicable or "non communicable" or chronic or weight* or overweight or "over weight" or overeat or "over eat" or bmi or obes* or "blood pressure" or hypertensi* or "blood glucose" or hyperglyc?mi* or cholester* or hypercholester* or hyperlipid?emia*) ) or AB ( (non-communicable or "non communicable" or chronic or weight* or overweight or "over weight" or overeat or "over eat" or bmi or obes* or "blood pressure" or hypertensi* or "blood glucose" or hyperglyc?mi* or cholester* or hypercholester* or hyperlipid?emia*) )) AND (TI ( (econom* or finance* or cost or costs or costing or pric* or monetis* or income* or wage* or salar* or expenditure* or time* or tax* or subsid* or credit* or cash or transfer or payment or welfare or benefit* or incentiv* or disincentiv* or remunerat* or retail* or sale* or promo* or consumer* or consumption* or purchas* or shop* or buy* or product* or good* or service* or market*) ) or AB ( (econom* or finance* or cost or costs or costing or pric* or monetis* or income* or wage* or salar* or expenditure* or time* or tax* or subsid* or credit* or cash or transfer or payment or welfare or benefit* or incentiv* or disincentiv* or remunerat* or retail* or sale* or promo* or consumer* or consumption* or purchas* or shop* or buy* or product* or good* or service* or market*) ))

**SPORTDiscus with Full Text (EBSCO). 1800 to July 2011.**

(TI ( (nutri* or calori* or diet* or food* or eat* or meal* or snack* or cook* or restaurant* or supermarket* or cafe*) ) or AB ( (nutri* or calori* or diet* or food* or eat* or meal* or snack* or cook* or restaurant* or supermarket* or cafe*) ) or TI ( ((drink* or beverage*) not alcohol*) ) or AB ( ((drink* or beverage*) not alcohol*) ) or TI ( (exercise* or train* or activit* or fit* or endur* or exertion or aerobic or strength* or flexib* or balance* or recreation* or leisure or play or energ* or walk* or hike or hiking or climbing or run* or jog* or swim* or aqua* or bicycl* or cycl* or bike* or biking or gym* or rowing or canoe* or kayak* or sailing or windsurf* or surf* or diving or sport* or rollerblading or rollerskating or skating or skiing or yoga or pilates or calisthenics or jump* or lift* or circuit or resistance or resilience or dance or dancing or fishing or hunting or shooting or cardiorespiratory or sedentary or deskbound or inactiv*) ) or AB ( (exercise* or train* or activit* or fit* or endur* or exertion or aerobic or strength* or flexib* or balance* or recreation* or leisure or play or energ* or walk* or hike or hiking or climbing or run* or jog* or swim* or aqua* or bicycl* or cycl* or bike* or biking or gym* or rowing or canoe* or kayak* or sailing or windsurf* or surf* or diving or sport* or rollerblading or rollerskating or skating or skiing or yoga or pilates or calisthenics or jump* or lift* or circuit or resistance or resilience or dance or dancing or fishing or hunting or shooting or cardiorespiratory or sedentary or deskbound or inactiv*) ) or TI ( (non-communicable or "non communicable" or chronic or weight* or overweight or "over weight" or overeat or "over eat" or bmi or obes* or "blood pressure" or hypertensi* or "blood glucose" or hyperglyc?mi* or cholester* or hypercholester* or hyperlipid?emia*) ) or AB ( (non-communicable or "non communicable" or chronic or weight* or overweight or "over weight" or overeat or "over eat" or bmi or obes* or "blood pressure" or hypertensi* or "blood glucose" or hyperglyc?mi* or cholester* or hypercholester* or hyperlipid?emia*) )) AND (TI ( (econom* or finance* or cost or costs or costing or pric* or monetis* or income* or wage* or salar* or expenditure* or time* or tax* or subsid* or credit* or cash or transfer or payment or welfare or benefit* or incentiv* or disincentiv* or remunerat* or retail* or sale* or promo* or consumer* or consumption* or purchas* or shop* or buy* or product* or good* or service* or market*) ) or AB ( (econom* or finance* or cost or costs or costing or pric* or monetis* or income* or wage* or salar* or expenditure* or time* or tax* or subsid* or credit* or cash or transfer or payment or welfare or benefit* or incentiv* or disincentiv* or remunerat* or retail* or sale* or promo* or consumer* or consumption* or purchas* or shop* or buy* or product* or good* or service* or market*) ))

**Applied Social Sciences Index and Abstracts (CSA Illumina). 1987 to 2 August 2011.**

((KW=(nutri*) or KW=(calori*) or KW=(diet*) or KW=(food*) or KW=(eat*) or KW=(meal*) or KW=(snack*) or KW=(cook*) or KW=(restaurant*) or KW=(supermarket*) or KW=("café*") or KW=(cafe) or ((KW=(drink*) or KW=(beverage*)) not KW=(alcohol*)) or KW=(exercise*) or KW=(train*) or KW=(activit*) or KW=(fit*) or KW=(endur*) or KW=(exertion) or KW=(aerobic) or KW=(strength*) or KW=(flexib*) or KW=(balance*) or KW=(recreation*) or KW=(leisure) or KW=(play) or KW=(energ*) or KW=(walk*) or KW=(hike) or KW=(hiking) or KW=(climbing) or KW=(run*) or KW=(jog*) or KW=(swim*) or KW=(aqua*) or KW=(bicycl*) or KW=(cycl*) or KW=(bike*) or KW=(biking) or KW=(gym*) or KW=(rowing) or KW=(canoe*) or KW=(kayak*) or KW=(sailing) or KW=(windsurf*) or KW=(surf*) or KW=(diving) or KW=(sport*) or KW=(rollerblading) or KW=(rollerskating) or KW=(skating) or KW=(skiing) or KW=(yoga) or KW=(pilates) or KW=(calisthenics) or KW=(jump*) or KW=(lift*) or KW=(gym*) or KW=(circuit) or KW=(resistance) or KW=(resilience) or KW=(dance) or KW=(dancing) or KW=(fishing) or KW=(hunting) or KW=(shooting) or KW=(cardiorespiratory) or KW=(sedentary) or KW=(deskbound) or KW=(inactiv*) or KW=("non-communicable") or KW=("non communicable") or KW=(chronic) or KW=(weight*) or KW=(overweight) or KW=("over weight") or KW=(overeat) or KW=("over eat") or KW=(bmi) or KW=(obes*) or KW=("blood pressure") or KW=(hypertensi*) or KW=("blood glucose") or KW=(hyperglyc?mi*) or KW=(cholester*) or KW=(hypercholester*) or KW=(hyperlipid?emia*)) and (KW=(econom*) or KW=(finance*) or KW=(cost) or KW=(costs) or KW=(costing) or KW=(pric*) or KW=(monetis*) or KW=(income*) or KW=(wage*) or KW=(salar*) or KW=(expenditure*) or KW=(time*) or KW=(tax*) or KW=(subsid*) or KW=(credit*) or KW=(cash) or KW=(transfer) or KW=(payment) or KW=(welfare) or KW=(benefit*) or KW=(incentiv*) or KW=(disincentiv*) or KW=(remunerat*) or KW=(retail*) or KW=(sale*) or KW=(promo*) or KW=(consumer*) or KW=(consumption*) or KW=(purchas*) or KW=(shop*) or KW=(buy*) or KW=(product*) or KW=(good*) or KW=(service*) or KW=(market*))) not (KW=(animal model*) or KW=(animal*) or KW=(animal experiment*) or KW=(animal disease model*) or KW=(laboratory animal*))

**The Cochrane Library (Wiley Online Library).** **03/08/2011.**

- Cochrane Database of Systematic Reviews
- Database of Abstracts of Reviews of Effects
- Health Technology Assessment Database
- NHS Economic Evaluations Database

#1 MeSH descriptor Diet explode all trees
#2 MeSH descriptor Diet Therapy explode all trees
#3 MeSH descriptor Food explode all trees
#4 MeSH descriptor Beverages explode all trees
#5 MeSH descriptor Food Habits, this term only
#6 MeSH descriptor Food Preferences, this term only
#7 MeSH descriptor Fasting, this term only
#8 MeSH descriptor Adolescent Nutritional Physiological Phenomena, this term only
#9 MeSH descriptor Elder Nutritional Physiological Phenomena, this term only
#10 MeSH descriptor Food Industry explode all trees
#11 MeSH descriptor Hunger explode all trees
#12 MeSH descriptor Appetite Regulation explode all trees
#13 MeSH descriptor Appetite explode all trees
#14 MeSH descriptor Digestion explode all trees
#15 MeSH descriptor Eating explode all trees
#16 MeSH descriptor Eating Disorders explode all trees
#17 MeSH descriptor Child Nutrition Disorders explode all trees
#18 MeSH descriptor Infant Nutrition Disorders explode all trees
#19 MeSH descriptor Nutritional Requirements, this term only
#20 MeSH descriptor Nutritional Status, this term only
#21 MeSH descriptor Nutrition Assessment, this term only
#22 MeSH descriptor Nutrition Disorders, this term only
#23 MeSH descriptor Nutritive Value explode all trees
#24 (nutri* or calori* or diet* or food* or eat* or meal* or snack* or cook* or restaurant* or supermarket* or cafe*):ti
#25 (nutri* or calori* or diet* or food* or eat* or meal* or snack* or cook* or restaurant* or supermarket* or cafe*):ab
#26 ((drink* or beverage*) not alcohol*):ti
#27 ((drink* or beverage*) not alcohol*):ab
#28 (#1 OR #2 OR #3 OR #4 OR #5 OR #6 OR #7 OR #8 OR #9 OR #10 OR #11 OR #12 OR #13 OR #14 OR #15 OR #16 OR #17 OR #18 OR #19 OR #20 OR #21 OR #22 OR #23 OR #24 OR #25 OR #26 OR #27)
#29 MeSH descriptor Physical Exertion, this term only
#30 MeSH descriptor Human Activities explode all trees
#31 MeSH descriptor Leisure Activities explode all trees
#32 MeSH descriptor Locomotion explode all trees
#33 MeSH descriptor Physical Education and Training explode all trees
#34 MeSH descriptor Life Style, this term only
#35 MeSH descriptor Sedentary Lifestyle, this term only
#36 MeSH descriptor Yoga, this term only
#37 MeSH descriptor Fitness Centers, this term only
#38 MeSH descriptor Motor Activity, this term only
#39 (exercise* or train* or activit* or fit* or endur* or exertion or aerobic or strength* or flexib* or balance* or recreation* or leisure or play or energ* or walk* or hike or hiking or climbing or run* or jog* or swim* or aqua* or bicycl* or cycl* or bike* or biking or gym* or rowing or canoe* or kayak* or sailing or windsurf* or surf* or diving):ti
#40 (exercise* or train* or activit* or fit* or endur* or exertion or aerobic or strength* or flexib* or balance* or recreation* or leisure or play or energ* or walk* or hike or hiking or climbing or run* or jog* or swim* or aqua* or bicycl* or cycl* or bike* or biking or gym* or rowing or canoe* or kayak* or sailing or windsurf* or surf* or diving):ab
#41 (sport* or rollerblading or rollerskating or skating or skiing or yoga or pilates or calisthenics or jump* or lift* or gym* or circuit or resistance or resilience or dance or dancing or fishing or hunting or shooting or cardiorespiratory or sedentary or deskbound or inactiv*):ti
#42 (sport* or rollerblading or rollerskating or skating or skiing or yoga or pilates or calisthenics or jump* or lift* or gym* or circuit or resistance or resilience or dance or dancing or fishing or hunting or shooting or cardiorespiratory or sedentary or deskbound or inactiv*):ab
#43 (#29 OR #30 OR #31 OR #32 OR #33 OR #34 OR #35 OR #36 OR #37 OR #38 OR #39 OR #40 OR #41 OR #42)
#44 MeSH descriptor Blood Pressure, this term only
#45 MeSH descriptor Hypertension, this term only
#46 MeSH descriptor Blood Glucose, this term only
#47 MeSH descriptor Hyperglycemia, this term only
#48 MeSH descriptor Cholesterol, this term only
#49 MeSH descriptor Cholesterol, Dietary, this term only
#50 MeSH descriptor Cholesterol, HDL, this term only
#51 MeSH descriptor Cholesterol, LDL, this term only
#52 MeSH descriptor Cholesterol, VLDL, this term only
#53 MeSH descriptor Cholesterol Esters, this term only
#54 MeSH descriptor Hypercholesterolemia, this term only
#55 MeSH descriptor Hyperlipidemias explode all trees
#56 MeSH descriptor Body Weight Changes explode all trees
#57 MeSH descriptor Harm Reduction, this term only
#58 MeSH descriptor Overnutrition explode all trees
#59 MeSH descriptor Overweight explode all trees
#60 MeSH descriptor Obesity explode all trees
#61 (non-communicable or "non communicable" or chronic or weight* or overweight or "over weight" or overeat or "over eat" or bmi or obes* or "blood pressure" or hypertensi* or "blood glucose" or hyperglyc?mi* or cholester* or hypercholester* or hyperlipid?emia*):ti
#62 (non-communicable or "non communicable" or chronic or weight* or overweight or "over weight" or overeat or "over eat" or bmi or obes* or "blood pressure" or hypertensi* or "blood glucose" or hyperglyc?mi* or cholester* or hypercholester* or hyperlipid?emia*):ab
#63 (#44 OR #45 OR #46 OR #47 OR #48 OR #49 OR #50 OR #51 OR #52 OR #53 OR #54 OR #55 OR #56 OR #57 OR #58 OR #59 OR #60 OR #61 OR #62)
#64 (econom* or finance* or cost or costs or costing or pric* or monetis* or income* or wage* or salar* or expenditure* or time* or tax* or subsid* or credit* or cash or transfer or payment or welfare or benefit* or incentiv* or disincentiv* or remunerat* or retail* or sale* or promo* or consumer* or consumption* or purchas* or shop* or buy* or product* or good* or service* or market*):ti
#65 (econom* or finance* or cost or costs or costing or pric* or monetis* or income* or wage* or salar* or expenditure* or time* or tax* or subsid* or credit* or cash or transfer or payment or welfare or benefit* or incentiv* or disincentiv* or remunerat* or retail* or sale* or promo* or consumer* or consumption* or purchas* or shop* or buy* or product* or good* or service* or market*):ab
#66 (#64 OR #65)
#67 (#28 AND #66)
#68 (#43 AND #66)
#69 (#63 AND #66)
#70 (#67 OR #68 OR #69)

**Database of Promoting Health Effectiveness Reviews (EPPI-Centre). 11/08/2011.**

"nutri*" or "calori*" or "diet*" or "food*" or "eat*" or "meal*" or "snack*" or "cook*" or "restaurant*" or "supermarket*" or "cafe*" or "drink*" or "beverage*" or "exercise*" or "train*" or "activit*" or "fit*" or "endur*" or exertion or aerobic or "strength*" or "flexib*" or "balance*" or "recreation*" or "leisure" or "play" or "energ*" or "walk*" or hike or hiking or climbing or"run*" or "jog*" or "swim*" or "aqua*" or "bicycl*" or "cycl*" or "bike*" or biking or "gym*" or rowing or "canoe*" or "kayak*" or sailing or "windsurf*" or "surf*" or diving or "sport*" or "roller*" or skating or skiing or yoga or pilates or calisthenics or "jump*" or "lift*" or circuit or resistance or resilience or dance or dancing or fishing or hunting or shooting or cardiorespiratory or sedentary or deskbound or "inactiv*" or "non communicable" or "non-communicable" or chronic or "weight*" or overweight or "over weight" or overeat or "over eat" or bmi or "obes*" or "blood pressure" or "hypertensi*" or "blood glucose" or "hyperglyc*" or "cholester*" or "hypercholester*" or "hyperlipid*" or (Focus of the report: cardiovascular or diabetes or healthy eating or leisure or obesity or physical activity)
