# Supplementary material for: Economic Instruments for Population Diet and Physical Activity Behaviour Change: A Systematic Scoping Review
Source: PLoS One. 2013 Sep 24;8(9):e75070. doi: 10.1371/journal.pone.0075070 (PMC3782495; doi:10.1371/journal.pone.0075070)
Supplement: Table S1 — Electronic search dates and yields, by database. (DOCX) [file pone.0075070.s006.docx]

**Table S1. Electronic search dates and yields, by database**

| **Database** | **Date of Search** | **Yield (Records)** |
| --- | --- | --- |
| MEDLINE (Ovid SP) | 11/07/11 | 432,641 |
| EMBASE (Ovid SP) | 12/07/11 | 619,990 |
| PsycINFO (Ovid SP) | 19/07/11 | 68,659 |
| EconLit (EBSCO) | 21/07/11 | 152,188 |
| SPORTDiscus with Full Text | 01/08/11 | 90,526 |
| Applied Social Sciences Index and Abstracts (CSA Illumina) | 02/08/11 | 56,523 |
| Cochrane Database of Systematic Reviews (Wiley Online Library) | 03/08/11 | 2,427 |
| Database of Abstracts of Reviews of Effects (Wiley Online Library) | 03/08/11 | 171 |
| Health Technology Assessment Database (Wiley Online Library) | 03/08/11 | 139 |
| NHS Economic Evaluation Database (Wiley Online Library) | 03/08/11 | 1,164 |
| Database of Promoting Health Effectiveness Reviews (EPPI-Centre) | 11/08/11 | 1,604 |
| **Total** (including duplicates) | **-** | **1,426,032** |
