# Supplementary material for: Economic Instruments for Population Diet and Physical Activity Behaviour Change: A Systematic Scoping Review
Source: PLoS One. 2013 Sep 24;8(9):e75070. doi: 10.1371/journal.pone.0075070 (PMC3782495; doi:10.1371/journal.pone.0075070)
Supplement: Table S2 — Characteristics of included studies. (DOCX) [file pone.0075070.s007.docx]

**Table S2. Characteristics of included studies**

|  | Study design | | | Target behaviour(s) | | Population(s) | | | Intervention(s) | | | | | | Outcomes | | | |
| --- | --- | --- | --- | --- | --- | --- | --- | --- | --- | --- | --- | --- | --- | --- | --- | --- | --- | --- |
| First author - year | Primary experimental study | Primary quasi-experimental, non-experimental, modelling | Review | Diet | Physical activity | United Kingdom | United States | Other HIC(s) | | Price promotions | Taxes | Supply side subsidies | Direct unit pricing | Transfer payments | Purchasing behaviour(s) | Other behaviour(s) | Proximal consequence(s) | Distal consequence(s) |
| Abdus 2008 |  | ● |  | ● |  |  | ● |  | |  | ● |  |  |  | ● |  |  | ● |
| Akin 1983 |  | ● |  | ● |  |  | ● |  | |  |  | ● |  | ● |  |  | ● |  |
| Allais 2010 |  | ● |  | ● |  |  |  | ● | |  | ● |  |  |  | ● |  |  |  |
| Alston 2008 |  |  | ● | ● |  | ● | ● | ● | |  |  | ● |  |  |  |  |  | ● |
| Alston 2009 |  | ● |  | ● |  |  | ● |  | |  |  |  |  | ● | ● |  |  |  |
| Alston 2010 |  | ● |  | ● |  |  | ● |  | |  |  | ● |  |  | ● |  |  | ● |
| Andreyeva 2011 |  | ● |  | ● |  |  | ● |  | |  | ● |  |  |  | ● |  |  |  |
| Arcia 1990 |  | ● |  | ● |  |  | ● |  | |  |  |  |  | ● | ● |  |  |  |
| Arnoult 2008 |  | ● |  | ● |  | ● |  |  | |  | ● | ● |  |  | ● |  |  | ● |
| Arsenault 2003 |  | ● |  | ● |  |  | ● |  | |  |  | ● |  | ● |  |  | ● |  |
| Basiotis 1987 |  | ● |  | ● |  |  | ● |  | |  |  | ● |  | ● |  |  | ● |  |
| Baum 2008 |  | ● |  | ● |  |  | ● |  | |  |  |  |  | ● |  |  |  | ● |
| Baum 2011 |  | ● |  | ● |  |  | ● |  | |  |  |  |  | ● |  |  |  | ● |
| Bell 1999 |  | ● |  | ● |  |  | ● |  | | ● |  |  |  |  | ● | ● |  |  |
| Bergman 2010 |  | ● |  |  | ● |  |  | ● | |  | ● |  |  |  |  | ● |  |  |
| Billson 1999 |  | ● |  | ● |  | ● |  |  | |  |  |  |  | ● |  |  | ● |  |
| Binkley 2006 |  | ● |  | ● |  |  | ● |  | |  |  |  |  | ● |  | ● |  |  |
| Bitler 2004 |  | ● |  | ● |  |  | ● |  | |  |  |  |  | ● |  |  |  | ● |
| Block 2010 |  | ● |  | ● |  |  | ● |  | | ● |  |  |  |  | ● |  |  |  |
| Bonnet 2011b |  | ● |  | ● |  |  |  | ● | |  | ● |  |  |  | ● |  |  |  |
| Briefel 2009 |  | ● |  | ● |  |  | ● |  | |  |  | ● |  |  |  | ● | ● |  |
| Brownson 2006 |  |  | ● | ● | ● |  | ● |  | | ● | ● |  |  | ● | ● |  | ● |  |
| Burstein 2000 |  | ● |  | ● |  |  | ● |  | |  |  |  |  | ● |  | ● |  |  |
| Butler 1985 |  | ● |  | ● |  |  | ● |  | |  |  |  |  | ● |  |  | ● |  |
| Caraher 2005 |  |  | ● | ● |  |  | ● |  | |  | ● |  |  |  | ● |  | ● | ● |
| Caraher 2007 |  |  | ● | ● |  |  | ● |  | |  | ● |  |  |  | ● |  | ● |  |
| Cash 2007a |  |  | ● | ● |  |  | ● | ● | | ● | ● | ● |  |  | ● |  | ● | ● |
| Cawley 2011 |  |  | ● | ● |  |  | ● |  | |  | ● |  |  |  | ● |  | ● | ● |
| Chaloupka 2009a |  |  | ● | ● |  |  | ● | ● | |  | ● |  |  |  |  |  |  | ● |
| Chaloupka 2011 |  |  | ● | ● |  |  | ● |  | |  | ● |  |  |  | ● |  | ● | ● |
| Chavas 1982 |  | ● |  | ● |  |  | ● |  | |  |  |  |  | ● | ● |  |  |  |
| Chavas 1983 |  | ● |  | ● |  |  | ● |  | |  |  | ● |  | ● |  |  | ● |  |
| Chen 2005 |  | ● |  | ● |  |  | ● |  | |  |  |  |  | ● |  |  |  | ● |
| Chouinard 2007 |  | ● |  | ● |  |  | ● |  | |  | ● |  |  |  | ● |  |  |  |
| Cole 2004 |  | ● |  | ● | ● |  | ● |  | |  |  |  |  | ● |  | ● | ● | ● |
| Curhan 1974 |  | ● |  | ● |  |  | ● |  | | ● |  |  |  |  | ● |  |  |  |
| Currie 2003 |  |  | ● | ● |  |  | ● |  | |  |  | ● |  | ● |  |  | ● | ● |
| Davis 1979 |  | ● |  | ● |  |  | ● |  | |  |  |  |  | ● | ● |  | ● |  |
| Devaney 1991 |  | ● |  | ● |  |  | ● |  | |  |  | ● |  | ● |  |  | ● |  |
| Devaney 1993 |  | ● |  | ● |  |  | ● |  | |  |  | ● |  |  |  |  | ● |  |
| Dong 2009 |  | ● |  | ● |  |  | ● |  | |  |  | ● |  |  | ● |  |  |  |
| Drewnowski 2004b |  |  | ● | ● |  |  | ● |  | |  |  |  |  | ● | ● |  | ● |  |
| Emmons 1987 |  | ● |  | ● |  |  | ● |  | |  |  |  |  | ● |  |  | ● |  |
| Epstein 2010 | ● |  |  | ● |  |  | ● |  | |  | ● | ● |  |  | ● |  |  |  |
| Faith 2007 |  |  | ● | ● |  |  | ● |  | | ● |  |  |  |  | ● |  | ● | ● |
| Fan 2010 |  | ● |  | ● |  |  | ● |  | |  |  |  |  | ● |  |  |  | ● |
| Faulkner 2010 |  |  | ● | ● | ● |  | ● | ● | |  | ● | ● |  | ● | ● | ● | ● | ● |
| Fey-Yensan 2003 |  | ● |  | ● |  |  | ● |  | |  |  |  |  | ● |  |  | ● | ● |
| Finkelstein 2010 |  |  | ● | ● |  |  | ● |  | |  | ● |  |  |  | ● |  |  | ● |
| Fletcher 2010b |  | ● |  | ● |  |  | ● |  | |  | ● |  |  |  |  |  | ● | ● |
| Fox 2004a |  | ● |  | ● | ● |  | ● |  | |  |  |  |  | ● |  | ● | ● | ● |
| French 1997a |  | ● |  | ● |  |  | ● |  | | ● |  |  |  |  | ● |  |  |  |
| French 1997b |  | ● |  | ● |  |  | ● |  | | ● |  |  |  |  | ● |  |  |  |
| French 2001a |  | ● |  | ● |  |  | ● |  | | ● |  |  |  |  | ● |  |  |  |
| French 2001b |  |  | ● | ● |  |  | ● |  | | ● |  |  |  |  | ● |  |  |  |
| French 2004b |  |  | ● | ● |  |  | ● |  | | ● |  |  |  |  | ● |  |  |  |
| French 2010 |  | ● |  | ● |  |  | ● |  | | ● |  |  |  |  | ● |  | ● |  |
| Gabe 2008 |  | ● |  | ● |  |  | ● |  | |  | ● |  |  |  | ● |  |  |  |
| Gibson 2003 |  | ● |  | ● |  |  | ● |  | |  |  |  |  | ● |  |  |  | ● |
| Gibson 2004 |  | ● |  | ● |  |  | ● |  | |  |  |  |  | ● |  |  |  | ● |
| Gibson 2006 |  | ● |  | ● |  |  | ● |  | |  |  |  |  | ● |  |  |  | ● |
| Glanz 2004a |  |  | ● | ● |  |  | ● | ● | |  |  |  |  | ● | ● |  |  |  |
| Gleason 1995 |  | ● |  | ● |  |  | ● |  | |  |  | ● |  |  |  | ● |  |  |
| Gleason 2000 |  | ● |  | ● |  |  | ● |  | |  |  |  |  | ● |  | ● | ● |  |
| Gleason 2003 |  | ● |  | ● |  |  | ● |  | |  |  | ● |  | ● |  |  | ● |  |
| Gleason 2009a |  | ● |  | ● |  |  | ● |  | |  |  | ● |  |  |  |  |  | ● |
| Gleason 2009b |  | ● |  | ● |  |  | ● |  | |  |  | ● |  |  |  | ● | ● | ● |
| Goodman 2006 |  |  | ● | ● |  |  | ● | ● | | ● | ● | ● |  | ● | ● |  | ● | ● |
| Gordon 1995 |  | ● |  | ● |  |  | ● |  | |  |  | ● |  |  |  |  | ● |  |
| Gordon 2010 |  | ● |  | ● |  |  | ● |  | |  |  | ● |  |  |  |  | ● | ● |
| Gorin 2007 | ● |  |  | ● |  |  | ● |  | |  |  | ● |  |  | ● |  |  | ● |
| Griffith 2009a |  | ● |  | ● |  | ● |  |  | |  | ● |  |  |  | ● |  |  |  |
| Gustavsen 2004 |  | ● |  | ● |  |  |  | ● | |  | ● |  |  | ● | ● |  |  |  |
| Gustavsen 2005 |  | ● |  | ● |  |  |  | ● | |  | ● |  |  |  | ● |  |  |  |
| Gustavsen 2011 |  | ● |  | ● |  |  |  | ● | |  | ● |  |  |  | ● |  |  | ● |
| Guthrie 2007 |  |  | ● | ● |  |  | ● |  | |  |  |  |  | ● |  |  | ● |  |
| Harnack 2008 | ● |  |  | ● |  |  | ● |  | | ● |  |  |  |  | ● |  | ● |  |
| Hawkes 2009a |  |  | ● | ● |  | ● | ● | ● | | ● | ● | ● |  | ● | ● |  | ● | ● |
| Hawkes 2009b |  |  | ● | ● |  | ● | ● | ● | | ● |  |  |  |  | ● |  | ● |  |
| Herbst 2009 |  | ● |  | ● | ● |  | ● |  | |  |  |  |  | ● |  |  |  | ● |
| Herman 2008 |  | ● |  | ● |  |  | ● |  | |  |  |  |  | ● |  |  | ● |  |
| Hernandez 2011 |  | ● |  | ● |  |  | ● |  | |  |  | ● |  |  |  |  |  | ● |
| Ho 1998 |  | ● |  | ● |  |  | ● |  | | ● |  |  |  |  | ● | ● |  |  |
| Hofferth 2005 |  | ● |  | ● |  |  | ● |  | |  |  | ● |  | ● | ● |  |  | ● |
| Horgen 2002 |  | ● |  | ● |  |  | ● |  | | ● |  |  |  |  | ● |  |  |  |
| Hoynes 2007 |  | ● |  | ● |  |  | ● |  | |  |  |  |  | ● |  | ● |  |  |
| Huang 1981 |  | ● |  | ● |  |  | ● |  | |  |  |  |  | ● | ● |  |  |  |
| Inglis 2009 |  | ● |  | ● |  |  |  | ● | |  |  |  |  | ● | ● |  |  |  |
| Jaime 2009 |  |  | ● | ● |  |  | ● |  | | ● |  |  |  |  | ● |  |  |  |
| Jeffery 1994 |  | ● |  | ● |  |  | ● |  | | ● |  |  |  |  | ● |  |  |  |
| Jensen 2007 |  | ● |  | ● |  |  |  | ● | |  | ● | ● |  |  | ● |  |  |  |
| Jilcott 2011b |  | ● |  | ● |  |  | ● |  | |  |  |  |  | ● |  | ● |  | ● |
| Jo 2009 |  | ● |  | ● |  |  | ● |  | |  |  |  |  | ● |  |  |  | ● |
| Jones 2003a |  | ● |  | ● |  |  | ● |  | |  |  | ● |  | ● |  |  |  | ● |
| Jones 2006 |  | ● |  | ● |  |  | ● |  | |  |  |  |  | ● |  |  |  | ● |
| Kaushall 2009 |  | ● |  | ● |  |  | ● |  | |  |  |  |  | ● | ● |  |  |  |
| Kim 2006 |  |  | ● | ● |  |  | ● | ● | | ● | ● |  |  |  | ● |  |  | ● |
| Kirkpatrick 2007 |  | ● |  | ● |  |  |  | ● | |  |  |  |  | ● | ● |  |  |  |
| Kuchler 2004 |  | ● |  | ● |  |  | ● |  | |  | ● |  |  |  | ● |  |  | ● |
| Kuchler 2005 |  | ● |  | ● |  |  | ● |  | |  | ● |  |  |  | ● |  |  |  |
| Lachappelle 2009 |  | ● |  |  | ● |  | ● |  | |  |  |  |  | ● |  | ● |  |  |
| Larson 2011 |  |  | ● | ● |  |  | ● |  | |  |  | ● |  | ● |  |  |  | ● |
| Leung 2011 |  | ● |  | ● |  |  | ● |  | |  |  |  |  | ● |  |  | ● | ● |
| Levi 2010 |  |  | ● | ● | ● |  | ● |  | |  | ● |  |  |  | ● |  | ● | ● |
| Levy 2011a |  |  | ● | ● |  |  | ● |  | |  | ● |  |  |  | ● |  |  | ● |
| Levy 2011b |  |  | ● | ● |  |  | ● |  | | ● | ● | ● |  |  | ● |  | ● | ● |
| Li 2010 |  | ● |  | ● |  |  | ● |  | |  |  | ● |  |  |  |  |  | ● |
| Lin 2010a |  | ● |  | ● |  |  | ● |  | |  |  | ● |  | ● | ● |  |  |  |
| Lin 2010b |  | ● |  | ● |  |  | ● |  | |  | ● |  |  |  | ● |  |  | ● |
| Lino 2002 |  | ● |  | ● |  |  | ● |  | |  |  |  |  | ● |  |  | ● |  |
| Linz 2005 |  |  | ● | ● |  |  | ● |  | |  |  | ● |  |  |  |  |  | ● |
| Lopez 2012 |  | ● |  | ● |  |  | ● |  | |  | ● |  |  |  | ● |  |  | ● |
| Lordan 2011 |  |  | ● | ● |  |  | ● |  | |  | ● | ● |  |  | ● |  |  | ● |
| Lucove 2007 |  | ● |  |  | ● |  | ● |  | |  |  |  |  | ● |  | ● |  |  |
| Madore 2007 |  |  | ● | ● | ● |  | ● | ● | | ● | ● | ● |  | ● | ● |  | ● |  |
| Matson-Koffman 2005 |  |  | ● | ● | ● |  | ● | ● | | ● |  |  |  | ● | ● |  | ● |  |
| Maurer 1984 |  | ● |  | ● |  |  | ● |  | |  |  | ● |  |  | ● |  |  |  |
| McInnes 2009 |  | ● |  |  | ● |  | ● |  | |  | ● |  |  |  |  | ● |  |  |
| Melgar-Quinonez 2004 |  | ● |  | ● |  |  | ● |  | |  |  |  |  | ● |  |  |  | ● |
| Mellor 2011 |  | ● |  | ● | ● |  | ● |  | |  | ● |  |  |  |  |  |  | ● |
| Meyerhoefer 2006 |  | ● |  | ● |  |  | ● |  | |  |  |  |  | ● |  |  |  | ● |
| Meyers 1994 |  | ● |  | ● | ● |  | ● |  | |  |  |  |  | ● |  |  |  | ● |
| Michels 2008 |  | ● |  | ● |  |  | ● |  | | ● |  |  |  |  | ● |  |  |  |
| Mishra 2011 | ● |  |  | ● |  |  | ● |  | | ● |  |  |  |  | ● |  |  |  |
| Mytton 2007 |  | ● |  | ● |  | ● |  |  | |  | ● |  |  |  |  |  | ● | ● |
| Nayga 1994 |  | ● |  | ● |  |  | ● |  | |  |  |  |  | ● |  |  | ● |  |
| Nederkoorn 2011 | ● |  |  | ● |  |  |  | ● | |  | ● |  |  |  | ● |  |  |  |
| Ni Mhurchu 2010 | ● |  |  | ● |  |  |  | ● | | ● |  |  |  |  | ● |  | ● |  |
| Nnoaham 2009 |  | ● |  | ● |  | ● |  |  | |  | ● | ● |  |  |  |  | ● |  |
| Nordstrom 2009 |  | ● |  | ● |  |  |  | ● | |  | ● | ● |  |  | ● |  |  |  |
| Oaks 2005 |  | ● |  | ● |  |  | ● |  | |  | ● |  |  |  |  |  |  | ● |
| Oliveira 2000 |  | ● |  | ● |  |  | ● |  | |  |  |  |  | ● |  |  | ● |  |
| Pan 2008 |  | ● |  | ● |  |  | ● |  | |  |  |  |  | ● | ● | ● |  |  |
| Parks 2011 |  | ● |  | ● | ● |  | ● |  | |  |  |  |  | ● |  | ● | ● | ● |
| Perez-Escamilla 2000 |  | ● |  | ● |  |  | ● |  | |  |  |  |  | ● |  |  | ● |  |
| Posner 1987 |  | ● |  | ● |  |  | ● |  | |  |  |  |  | ● |  |  | ● |  |
| Powell 2009c |  | ● |  | ● |  |  | ● |  | |  | ● |  |  |  |  |  |  | ● |
| Powell 2009e |  |  | ● | ● |  |  | ● |  | |  | ● | ● |  |  |  |  |  | ● |
| Raine 2005 |  |  | ● | ● |  |  | ● | ● | | ● | ● |  |  |  | ● |  | ● |  |
| Rashad 2006a |  | ● |  | ● | ● |  | ● |  | |  | ● |  |  |  |  |  |  | ● |
| Reed 2010 |  | ● |  | ● |  |  | ● |  | |  |  |  |  | ● | ● |  |  |  |
| Richards 2009 |  | ● |  | ● |  |  |  | ● | | ● |  |  |  |  | ● |  |  |  |
| Robinson 2009 |  | ● |  | ● |  |  | ● |  | |  |  |  |  | ● |  |  |  | ● |
| Robinson 2011 |  | ● |  | ● |  |  | ● |  | |  |  |  |  | ● |  |  |  | ● |
| Rose 1995 |  | ● |  | ● |  |  | ● |  | |  |  |  |  | ● |  |  | ● |  |
| Rose 1998 |  | ● |  | ● |  |  | ● |  | |  |  |  |  | ● |  |  | ● |  |
| Rush 1988 |  | ● |  | ● |  |  | ● |  | |  |  |  |  | ● |  |  | ● |  |
| Sacks 2011 |  | ● |  | ● |  |  |  | ● | |  | ● |  |  |  |  |  |  | ● |
| Salois 2011 |  | ● |  | ● |  |  | ● |  | |  |  |  |  | ● |  |  |  | ● |
| Scearce 1979 |  | ● |  | ● |  |  | ● |  | |  |  |  |  | ● | ● |  |  |  |
| Schanzenbach 1992 |  | ● |  | ● |  |  | ● |  | |  |  | ● |  |  |  |  |  | ● |
| Schindler 1992 | ● |  |  | ● |  |  | ● |  | | ● |  |  |  |  | ● |  |  |  |
| Schmeiser 2011 |  | ● |  | ● |  |  | ● |  | |  |  |  |  | ● |  |  |  | ● |
| Schnoover 2006 |  |  | ● | ● |  |  | ● |  | | ● |  |  |  |  | ● |  |  |  |
| Schroeter 2008 |  | ● |  | ● | ● |  | ● |  | |  | ● | ● |  |  |  |  |  | ● |
| Seymour 2004b |  |  | ● | ● |  |  | ● |  | | ● |  |  |  |  | ● |  | ● |  |
| Siega-Riz 2004 |  | ● |  | ● |  |  | ● |  | |  |  |  |  | ● |  | ● | ● |  |
| Smed 2007 |  | ● |  | ● |  |  |  | ● | |  | ● | ● |  |  | ● |  |  |  |
| Smeets 2007 |  |  | ● | ● |  |  | ● |  | | ● | ● |  |  |  | ● |  |  |  |
| Smith 2010 |  | ● |  | ● |  |  | ● |  | |  | ● |  |  |  | ● |  |  | ● |
| Spence 2010 |  | ● |  |  | ● |  |  | ● | |  |  |  |  | ● | ● | ● |  |  |
| Story 2008 |  |  | ● | ● |  |  | ● |  | | ● |  |  |  | ● | ● |  |  | ● |
| Sturm 2010 |  | ● |  | ● |  |  | ● |  | |  | ● |  |  |  | ● |  | ● | ● |
| Tefft 2008 |  | ● |  | ● |  |  | ● |  | |  | ● |  |  |  | ● |  |  |  |
| TfL 2008 |  | ● |  |  | ● | ● |  |  | |  | ● |  |  |  |  | ● |  |  |
| Thow 2010 |  |  | ● | ● |  | ● | ● | ● | |  | ● | ● |  |  | ● |  | ● | ● |
| Tiffin 2011 |  | ● |  | ● |  | ● |  |  | |  | ● | ● |  |  | ● |  |  |  |
| Todd 2010 |  |  | ● | ● |  |  | ● |  | |  | ● |  |  |  | ● |  | ● | ● |
| Ver Ploeg 2007 |  | ● |  | ● |  |  | ● |  | |  |  |  |  | ● |  |  |  | ● |
| Ver Ploeg 2008 |  |  | ● | ● |  |  | ● |  | |  |  |  |  | ● |  |  |  | ● |
| Ver Ploeg 2009 |  | ● |  | ● |  |  | ● |  | |  |  |  |  | ● |  |  |  | ● |
| Vermeersch 1984 |  | ● |  | ● |  |  | ● |  | |  |  | ● |  |  |  |  |  | ● |
| von Tigerstrom 2011 |  |  | ● | ● | ● |  | ● | ● | |  | ● | ● |  | ● | ● | ● | ● | ● |
| Wang 2010 |  | ● |  | ● |  |  | ● |  | |  | ● |  |  |  | ● |  |  | ● |
| Webb 2008 |  | ● |  | ● |  |  | ● |  | |  |  | ● |  | ● |  |  |  | ● |
| Whitfield 1992 |  | ● |  | ● |  |  | ● |  | |  |  |  |  | ● |  |  | ● |  |
| Wilde 1999 |  | ● |  | ● |  |  | ● |  | |  |  |  |  | ● |  |  | ● |  |
| Wilde 2000b |  | ● |  | ● |  |  | ● |  | |  |  |  |  | ● |  | ● | ● |  |
| Yarnoff 2010 |  | ● |  | ● |  |  | ● |  | |  | ● |  |  |  | ● |  | ● |  |
| Yen 2010 |  | ● |  | ● |  |  | ● |  | |  |  |  |  | ● |  |  | ● |  |
| Zagorsky 2009 |  | ● |  | ● |  |  | ● |  | |  |  |  |  | ● |  |  |  | ● |
