# Supplementary material for: Economic Instruments for Population Diet and Physical Activity Behaviour Change: A Systematic Scoping Review
Source: PLoS One. 2013 Sep 24;8(9):e75070. doi: 10.1371/journal.pone.0075070 (PMC3782495; doi:10.1371/journal.pone.0075070)
Supplement: Table S3 — Brief summaries of evidence by intervention type. (DOCX) [file pone.0075070.s008.docx]

**Table S3. Brief summaries of evidence by intervention type**

| **Intervention type and included studies** | **Summary** |
| --- | --- |
| **Price promotions**  Category comprises the subset of consumer sales promotions that involve reducing the unit retail price of one or more diet- and/or physical activity-related final consumer products or services  16 primary studies    (See also Table 1 and Bibliographies S5 - included studies: Bell 1999, Block 2010, Curhan 1974, French 1997a, French 1997b, French 2001a, French 2010, Harnack 2008, Ho 1998, Horgen 2002, Jeffery 1994, Michels 2008, Mishra 2011, Ni Mhurchu 2010, Richards 2009, Schindler 1992) | **Range of interventions encountered:**   - Simple discounts (price restructuring) applied to healthier foods (e.g. low-fat snack foods and fruits and vegetables) in discrete settings such as schools, universities, worksites (e.g. their cafeterias or vending machines), supermarkets or restaurants - Simple discounts and/or multi-buy deals applied to fast foods. - Coupons redeemable against the purchase of food and non-alcoholic beverages products.   **Summary of evidence:**  This corpus of primary studies focused exclusively on price promotions applied to foods or non-alcoholic beverages. No primary studies focused on price promotions applied to physical activity-related products or services.  The specific type of price promotion studied most frequently was simple discounts applied to healthier foods in discrete settings (usually accompanied by minimal or no promotional signage), typically using quasi-experimental study designs. In some of these primary studies, simple discounts were implemented concurrently with other intervention components (e.g. calorie labelling, health messages, nutrition education). A finding reported consistently across this relatively homogenous cluster of studies was that reducing the unit retail prices of healthier foods relative to less healthy foods in discrete settings was independently associated with increased levels of purchasing of healthier foods. This finding was consistent across the range of discrete settings studied. It was also consistent with conclusions reported in reviews that included assessment of this cluster of primary studies.  Few primary studies in this category assessed effects on food or nutrient intake alongside purchasing; those that did reported no association between price promotions and intake of healthy foods or nutrients. Few or no studies were located that assessed other specific types of price promotion (multi-buy deals, price-pack deals, price deals, introductory pricing, couponing or rebates). |
| **Taxes**  Category comprises taxes imposed on food commodities, intermediate goods or diet- or physical activity-related final consumer products or services  38 primary studies  (See also Table 1 and Bibliographies S5 - included studies: Abdus 2008, Allais 2010, Andreyeva 2011, Arnoult 2008, Bergman 2010, Bonnet 2011b, Chouinard 2007, Epstein 2010, Fletcher 2010b, Gabe 2008, Griffith 2009a, Gustavsen 2011, Gustavsen 2005, Gustavsen 2004, Jensen 2007, Kuchler 2005, Kuchler 2004, Lin 2010b, Lopez 2012, McInnes 2009, Mellor 2011, Mytton 2007, Nederkoorn 2011, Nordstrom 2009, Nnoaham 2009, Oaks 2005, Powell 2009c, Rashad 2006a, Sacks 2011, Schroeter 2008, Smed 2007, Smith 2010, Sturm 2010, Tefft 2008, TfL 2008, Tiffin 2011, Wang 2010, Yarnoff 2010) | **Range of interventions encountered:**     - Taxes imposed on less healthy foods and non-alcoholic beverages: Calorie taxes, Snack taxes, Soft drinks taxes. - Taxes imposed on nutrient components of foods and non-alcoholic beverages: Fat taxes, Sugar taxes. - Tax exemptions applied to healthier foods. - Congestion taxes imposed on motorised vehicle use in a defined geographical area. - Taxes imposed on gasoline. - Taxes imposed on cigarettes. - Taxes imposed on alcoholic beverages.   **Summary of evidence:**  The large majority of included studies of taxes considered those imposed on less healthy foods, non-alcoholic beverages and/or nutrients. Because relatively few legislatures outside the United States have implemented such policies, primary studies investigating the *observed* effects of implemented food-related taxes were rare. The majority of included primary studies of food-related taxes were modelling studies that estimated the *predicted* impacts of (typically) multiple hypothetical food tax scenarios on purchasing, dietary intake or outcomes relating to body weight in various populations, based on demand elasticities derived from purchasing data.  There was considerable between- and within-study heterogeneity in terms of characteristics of the tax scenarios considered (e.g. rates, form - ad valorem or per unit), data sources, analytic methods and modelling assumptions. Collectively, they assessed effects in terms of a large and diverse set of specific outcome metrics; >800 across included primary studies of food-related taxes, of which only ≈5% were assessed in more than one primary study.  Estimated own-price elasticities of foods, non-alcoholic beverages or nutrients derived from purchasing data were invariably greater than zero; it was therefore inevitable that models based on such data predicted that imposition of taxes would affect reductions in levels of purchasing of targeted less healthy products. In general, the magnitudes of reductions in purchasing were predicted to be small-to-moderate. Moreover, several studies reported concurrent reductions in levels of purchasing of healthy foods.  Although most primary studies of food-related taxes incorporated modelling of compensatory purchasing, whereby consumers substitute within or between taxed and untaxed sets of products, the range of substitute or complementary products incorporated into their analyses was invariably limited relative to the vast array of potential alternative food and beverage products available to consumers in practice. These studies also typically incorporated an assumption that 100% of the tax burden is ‘passed through’ to consumers in the form of commensurate increases in unit retail prices. This assumption is unlikely to be realistic and may therefore have led to overestimation of predicted impacts on levels of purchasing. The few primary studies that incorporated modelling of supply-side responses (e.g. pricing strategies that limit ‘pass through’ or product reformulation to avoid taxes) typically reported that such responses were predicted to attenuate beneficial impacts of taxes on purchasing behaviours.  Several of these studies extrapolated from impacts on food, beverage or nutrient purchasing to impacts on outcomes relating to body weight status and typically predicted small or negligible beneficial impacts. However, they typically incorporated an assumption that 100% of the foods, non-alcoholic beverages or nutrients that are purchased are also consumed. This assumption is unlikely to be realistic and may therefore have led to overestimation of predicted impacts on outcomes relating to body weight status.  Tax rates considered in most simulated scenarios were relatively small in magnitude (e.g. compared with those typically applied to tobacco or alcohol products). Several authors concluded that higher tax or subsidy rates than those considered in their studies might need to be imposed to have a meaningful impact on target outcomes. Several authors highlighted, but had rarely demonstrated, the potentially regressive nature of food-related taxes. Several studies included estimation of predicted impacts on tax revenues and invariably predicted an increase in such revenues.  Few studies of tax exemptions that might promote physical activity or taxes that might discourage sedentary activity were located. This finding is consistent with reviews. |
| **Supply-side subsidies**  Category comprises payments or reimbursements made from public funds to either producers of commodities or intermediate goods (i.e. subsidies paid on inputs), or to retailers or providers of diet- or physical activity-related final consumer products or services (i.e. subsidies paid on products or services) with the intention of stimulating an increase in quantities produced and offered for purchase and a corollary decrease in the unit retail prices  33 primary studies  (See also Table 1 and Bibliographies S5 - included studies: Alston 2010, Akin 1983, Arnoult 2008, Arsenault 2003, Basiotis 1987, Briefel 2009, Chavas 1983, Devaney 1993, Devaney 1991, Dong 2009, Epstein 2010, Gleason 2009a, Gleason 2009b, Gleason 2003, Gleason 1995, Gordon 2010, Gordon 1995, Gorin 2007, Hernandez 2011, Hofferth 2005, Jensen 2007, Jones 2003a, Li 2010, Lin 2010a, Maurer 1984, Nnoaham 2009, Nordstrom 2009, Schanzenbach 2005, Schroeter 2008, Smed 2007, Tiffin 2011, Vermeersch 1984, Webb 2008) | **Range of interventions encountered:**     - Agricultural commodity subsidies - Removal of agricultural commodity subsidies - School meals subsidies - Other meals subsidies - Healthier foods subsidies - Healthier non-alcoholic beverages subsidies - Specific nutrient subsidies - Food transportation or delivery subsidies   **Summary:**  All primary studies within this category considered food-related subsidies. None investigated subsidisation of physical activity-related products or services (this finding is consistent with reviews).  The largest corpus of primary studies within this category investigated participation in the US National School Lunch Program (NSLP) and/or the School Breakfast Program (SBP). Across primary studies, evidence appeared equivocal with respect to associations between NSLP and/or SBP participation and typically large sets of outcomes relating to food or nutrient intake and/or body weight status. For food or nutrient intake, primary studies typically reported mixed patterns of results and results with respect to specific outcomes appeared inconsistent between studies. For body weight status, reported results appeared similarly inconsistent between studies, including many null findings. Reviews that included coverage of such studies typically reported similarly equivocal conclusions. In conjunction with their use of quasi-experimental study designs and observational datasets with different structures (cross-sectional, time series or panel), this corpus of primary studies used a range of analytic methods to control for potential selection bias with respect to programme participation and observable participant characteristics. Sets of observable characteristics included as covariates in the analyses differed between studies. Between-study differences in reported results with respect to specific outcomes are in part likely to reflect heterogeneity in the covariates assessed.  A smaller corpus of primary studies investigated the effects of supply-side subsidies applied to healthier foods, non-alcoholic beverages or nutrients. These studies typically used the same general modelling approach described above with respect to studies of food-related taxes (indeed some studies modelled both tax and subsidy scenarios, or combined tax-subsidy scenarios in which predicted tax revenues were hypothecated to fund hypothetical subsidies). They also incorporated similar assumptions and overall reported similarly equivocal results with respect to a diverse range of specific outcomes. |
| **Direct pricing legislation**  Category comprises legislation enacted by government or other legislative authorities that imposes either a minimum (or maximum) unit retail price that must be paid by consumers for a category or set of less healthy (or healthy) diet- and/or physical activity-related final consumer products or services  0 primary studies | **Range of interventions encountered:**   - None   **Summary:**  No studies of the effects of direct pricing legislation on diet- or physical activity-related outcomes were located. |
| **Transfer payments**  Category comprises transfers of public or private funds to members of defined population sub-groups (e.g. income transfers, welfare benefits or assistance programs, tax credits)  72 primary studies  (See also Table 1 and Bibliographies S5 - included studies: Alston 2009, Akin 1983, Arcia 1990, Arsenault 2003, Basiotis 1987, Baum 2011, Baum 2008, Billson 1999, Binkley 2006, Bitler 2004, Burstein 2000, Butler 1985, Chavas 1983, Chavas 1982, Chen 2005, Cole 2004, Davis 1979, Devaney 1991, Emmons 1987, Fan 2010, Fen-Yensan 2003, Fox 2004a, Gleason 2003, Gleason 2000, Gibson 2006, Gibson 2004, Gibson 2003, Gustavsen 2004, Herbst 2009, Herman 2008, Hofferth 2005, Hoynes 2007, Huang 1981, Inglis 2009, Jilcott 2011b, Jo 2009, Jones 2006, Jones 2003a, Kaushall 2009, Kirkpatrick 2007, Lachappelle 2009, Leung 2011, Lin 2010a, Lucove 2007, Melgar-Quinonez 2004, Meyerhoefer 2006, Meyers 1994, Nayga 1994, Oliveira 2000, Pan 2008, Parks 2011, Perez-Escamilla 2000, Posner 1987, Reed 2010, Robinson 2011, Robinson 2009, Rose 1998, Rose 1995, Rush 1988, Salois 2011, Scearce 1979, Schmeiser 2012, Siega-Riz 2004, Spence 2010, Ver Ploeg 2009, Ver Ploeg 2007, Webb 2008, Whitfield 1982, Wilde 2000, Wilde 1999, Yen 2010, Zagorsky 2009) | **Range of interventions encountered:**   - Restricted income transfers, welfare benefits or welfare assistance programs - Unrestricted income transfers, welfare benefits or welfare assistance programs - Tax credits   **Summary:**  The large majority of studies within this category assessed the effects of (or associations between) transfer payments restricted for use to purchase foods on (and) diet-related behaviours and corollary outcomes. The largest corpus of primary studies of transfer payments investigated participation (and/or the monetary value of benefits received via participation) in one or both of two large US federal welfare assistance programmes with benefits restricted for use to purchase foods and non-alcoholic beverages: the Supplemental Nutrition Assistance Program (SNAP – formerly known as the Food Stamp Program) and/or the Special Supplemental Nutrition Program for Women Infants and Children (WIC). Overall, evidence appears equivocal with respect to associations between SNAP and/or WIC participation or benefit levels and typically large sets of assessed outcomes relating to food purchasing behaviours, shopping, mealtime and snacking behaviours, food or nutrient intake and/or body weight status.  No clear patterns of results could readily be discerned across these studies with respect to these outcomes and individual studies typically reported mixed or equivocal results. Reviews that included coverage of such studies typically reported similarly equivocal conclusions. Differences in reported results across studies with respect to specific outcomes are in part likely to reflect heterogeneity between study design characteristics (which was similar in both nature and extent to that described with respect to studies that assessed participation in the NSLP and/or SBP). |
